# Effectiveness of introducing pulse oximetry and clinical decision support algorithms for the management of sick children in primary care in Kenya and Senegal on referral and antibiotic prescription: the TIMCI quasi-experimental pre-post study

**DOI:** 10.1016/j.eclinm.2025.103196

**Published:** 2025-05-12

**Authors:** Hélène Langet, Papa Moctar Faye, Francis Njiri, Silvia Cicconi, Gillian A. Levine, Tracy R. Glass, Rose J. Kosgei, Kevin Ngari, Fabian Schaer, Aliou Thiongane, Jean Augustin Diégane Tine, Maymouna Ba, Leah F. Bohle, Mira Emmanuel-Fabula, Mouhamadou Mansour Faye, Susan Horton, Andolo Miheso, Mercy Mugo, Mariah Ngutu, Michael Ruffo, Janet Shauri, Ndèye Marème Sougou, Valérie D’Acremont, Kaspar Wyss, Ousmane Ndiaye, Fenella Beynon, the TIMCI Collaborator Group

**Affiliations:** a Swiss Tropical and Public Health Institute, Allschwil, Switzerland; b University of Basel, Basel, Switzerland; c Cheikh Anta Diop University of Dakar, Dakar, Senegal; d College of Health Sciences, University of Nairobi, Nairobi, Kenya; e PATH; f School of Public Health and Health Systems, University of Waterloo, Waterloo, Ontario, Canada; g Centre for Primary Care and Public Health (Unisanté), University of Lausanne, Switzerland

**Keywords:** Pulse oximetry, Hypoxaemia, Clinical decision support, Integrated management of childhood illness, Primary care, Child health, Quasi-experimental study

## Abstract

**Background:**

Acute illnesses are leading causes of death among children under-five, who often receive antibiotics unnecessarily, contributing to antimicrobial resistance. Pulse oximetry and digital Clinical Decision Support Algorithms (CDSAs) can strengthen the detection and management of severe childhood illnesses, and support antibiotic stewardship in primary care, but lack evidence for scale-up. This study sought to understand the real-world impact of these tools on urgent referrals and antibiotic prescription for children under-five.

**Methods:**

A quasi-experimental pre-post study of the implementation of pulse oximetry and CDSAs for healthcare providers (HCPs) managing sick children at primary care level was conducted in Kenya and Senegal. Sick children 0–59 months attending study facilities were eligible. Trained research assistants collected data from caregivers and facility records on Day 0, with a follow-up phone call at Day 7. Providers were advised to use pulse oximetry for all sick children in Kenya, and in Senegal for all 1–59 days, and for 2–59 months with cough or difficulty breathing, or a moderate to severe illness. Urgent referral was recommended for SpO_2_ <90% in Kenya and SpO_2_ <92% in Senegal. Primary outcomes were antibiotic prescription and urgent referral rates at Day 0. They were assessed using generalised estimating equations for logistic regression. Results were estimated in terms of odds ratios and risk differences (RDs), adjusted where computable. The study is registered with clinicaltrials.gov (NCT05065320).

**Findings:**

A total of 50,580 sick children (1–59 days: 979 pre, 1748 post; 2–59 months: 16,782 pre, 31,071 post) were enrolled from September 13, 2021 to February 8, 2023 in Kenya and August 16, 2021 to March 31, 2023 in Senegal. In the pre-intervention period, urgent referrals were rare (0.6% in 1–59 days; 0.4% in 2–59 months), while antibiotic prescriptions were common (53.9% in 1–59 days; 74.9% in 2–59 months). Intervention uptake was 75% in Kenya and 40% in Senegal where a protracted HCP strike affected the intervention. The prevalence of SpO_2_ values prompting an urgent referral recommendation was 1.3% in 1–59 days and 0.8% in 2–59 months, but few of them resulted in actual referrals (26.1% in 1–59 days; 11.4% in 2–59 months). There was no change in overall urgent referrals (RD 0.2% [−0.5%, 0.9%] in 1–59 days; adjusted RD 0.2% [−0.2%, 0.5%] in 2–59 months). Antibiotic prescription rate was reduced by 14.6% [8.7%, 20.6%] in 1–59 days and by 22.6% [18.3%, 26.9%] in 2–59 months in the post-intervention period while caregiver-reported recovery rates at Day 7 remained stable.

**Interpretation:**

When implemented in routine health systems at primary care level in Kenya and Senegal, pulse oximetry and CDSAs were not found to be associated with an increase in urgent referrals but likely mediated antibiotic prescription reductions. The absence of referral increase may stem from limited severe illness detection due to low hypoxaemia prevalence and barriers to referral, also affected in Senegal by a protracted post-intervention HCP strike. Strengthening the referral system and implementing broader antibiotic stewardship strategies are likely to be needed to improve the effectiveness of the intervention and its impact on child health outcomes.

**Funding:**

Unitaid grant n°2019-35-TIMCI: Tools for Integrated Management of Childhood Illness.

**Research in context**

**Evidence before this study**

The Integrated Management of Childhood Illnesses (IMCI) strategy has been widely adopted to improve child health outcomes in low- and middle-income countries (LMICs), but challenges remain in identifying and managing children with severe illnesses, while rationalising antibiotic prescription. Pulse oximetry and digital clinical decision support algorithms (CDSAs) show promise in addressing these challenges but more effectiveness evidence is needed to inform large-scale implementation.

We searched PubMed, without language restrictions, using the following title or abstract terms (or related terms): [*“pulse oximetry”* OR *“hypoxaemia”* OR *“CDSS”*] AND *“child”* AND *“primary care”* AND *“LMICs”* up to June 30th, 2024. A total of 314 publications were identified, of which 21 primary research studies and two systematic reviews were relevant to effectiveness.

Nine primary studies focused on pulse oximetry, five of which were included in a 2021 systematic review. These studies supported improved identification of severely ill children and increased referral eligibility. Evidence on patient outcomes along the care cascade was limited, largely due to the predominance of observational study designs (seven out of nine studies) and generally restricted to respiratory illness. No prior study was powered to assess the impact of pulse oximetry on referral.

Twelve primary studies focused on CDSAs, eleven of which were included in a 2024 systematic review. The meta-analysis in the systematic review revealed overall improvements in quality of care and reduced antibiotic prescription, although definitions and data sources varied, and one IMCI-based study reported lower treatment appropriateness. Evidence on referrals was scarce and heterogeneous. The largest and most recent cluster RCT evaluating an IMCI-expanded algorithms—not included in the review—similarly found a three-fold reduction in antibiotic prescription and no significant difference in referral.

**Added value of this study**

This quasi-experimental pre-post study is one of the largest evaluations of the effectiveness of pulse oximetry with CDSAs in primary care. While it did not demonstrate the hypothesised increase in urgent referrals, it confirmed a statistically significant reduction in antibiotic prescriptions, sourced from paper prescription and medication in-hand. These findings also highlighted the importance of contextual and implementation factors in influencing effectiveness.

**Implications of all the available evidence**

The available evidence highlights the value of pulse oximetry in identifying severe illnesses and of CDSAs in supporting guideline adherence and reducing unnecessary antibiotic prescriptions. This study confirms that both tools can be implemented at scale in primary care settings, although their combined impact on referral and antibiotic prescription varies across epidemiological, implementation and health system contexts. To maximise effectiveness, future implementation efforts should extend beyond primary care to address broader health system challenges, and in particular referral barriers.

## Introduction

Acute illnesses remain the leading causes of death and disability among children under-five.^1^ Compounding this challenge, children receive an estimated 18 to 25 courses of antibiotics during their first five years, many of which are prescribed in primary care.^2^, ^3^, ^4^ High levels of antibiotic use contribute to the growing public health problem of bacterial antimicrobial resistance, responsible for 1.27 million deaths annually, with the highest burden borne by Western and Eastern Sub-Saharan Africa.^5^

Efforts to address these challenges in primary care have been sustained since the introduction of the Integrated Management of Childhood Illness (IMCI) guidelines in the 1990s by the World Health Organization (WHO) and the United Nations Children Funds (UNICEF).^6^ Encompassing both curative and preventive strategies, these guidelines provide a simple syndromic approach, intended to have high sensitivity for common causes of under-five mortality and support healthcare providers (HCPs) in effectively identifying and managing childhood illnesses. Despite being adopted by over 100 countries,^1^ the implementation of IMCI faces several challenges that impact its effectiveness. More specifically, a number of studies report low adherence,^7^^,^^8^ while some studies highlight limitations in detecting hypoxaemia (low blood oxygen saturation, SpO_2_) based on symptoms and signs only, which is strongly associated with morbidity and mortality.^9^

Pulse oximetry combined with clinical decision support algorithms (CDSAs) holds potential to support primary HCPs in addressing this dual challenge. For the past two decades, there have been calls to incorporate pulse oximetry—the standard and simple tool for non-invasive measurement of SpO_2_—to be used as part of the assessment of children with clinical signs of pneumonia.^10^ But despite a number of studies demonstrating feasibility, and potential to improve detection and referral of children with hypoxaemia in primary care, its use remains scarce.^11^, ^12^, ^13^ CDSAs—digital tools providing step-by-step guidance to HCPs on assessment and treatment—have been recommended by WHO to support guideline adherence,^14^ and could facilitate the effective use of pulse oximetry. Several CDSAs have shown promise in supporting improvements in the quality of assessment and management of childhood illness in primary care, including on antibiotic stewardship with the reduction of unnecessary antibiotic prescriptions, though findings are heterogeneous.^15^, ^16^, ^17^, ^18^, ^19^, ^20^

Despite their promise, evidence on implementation approaches, effectiveness and cost-effectiveness of these tools, especially in resource-constrained health systems, remains limited and hampers decision making about scale-up.^21^ The Tools for Integrated Management of Childhood Illness (TIMCI) project sought to address this gap by introducing and generating evidence on pulse oximetry and CDSAs to support HCPs in assessing and managing sick children under-five attending primary care facilities in India, Kenya, Senegal and Tanzania. The large-scale, multi-country, pragmatic mixed methods evaluation protocol is described elsewhere.^22^ This article examines whether the combined introduction of pulse oximetry and CDSAs, relying on established care delivery mechanisms within health systems, results in increased urgent referral and reduced antibiotic prescription for sick children under-five attending primary care in Kenya and Senegal, as markers of improved quality of care that may ultimately contribute to better child health outcomes.

## Methods

### Study design

A quasi-experimental pre-post study of the implementation of pulse oximetry and CDSAs for HCPs managing sick children in primary care was conducted in Kenya and Senegal (NCT05065320). The study aimed to compare care practices, including antibiotic prescriptions and urgent referrals, before (*pre*) and after (*post*) intervention rollout. Ethical approvals were obtained from the Kenyatta National Hospital Ethic Review Committee (P333/06/2020, KNH/ERC/R/235) on October 6, 2020, the Comité National d’Ethique pour la Recherche en Santé (SEN20/50) on September 15, 2020, and the WHO Ethics Review Committee (ERC.0003406) on January 21, 2021 (v2.3; v2.4: January 19, 2022; v2.5: April 5, 2023).

### Settings and participants

**Fig. 1:**
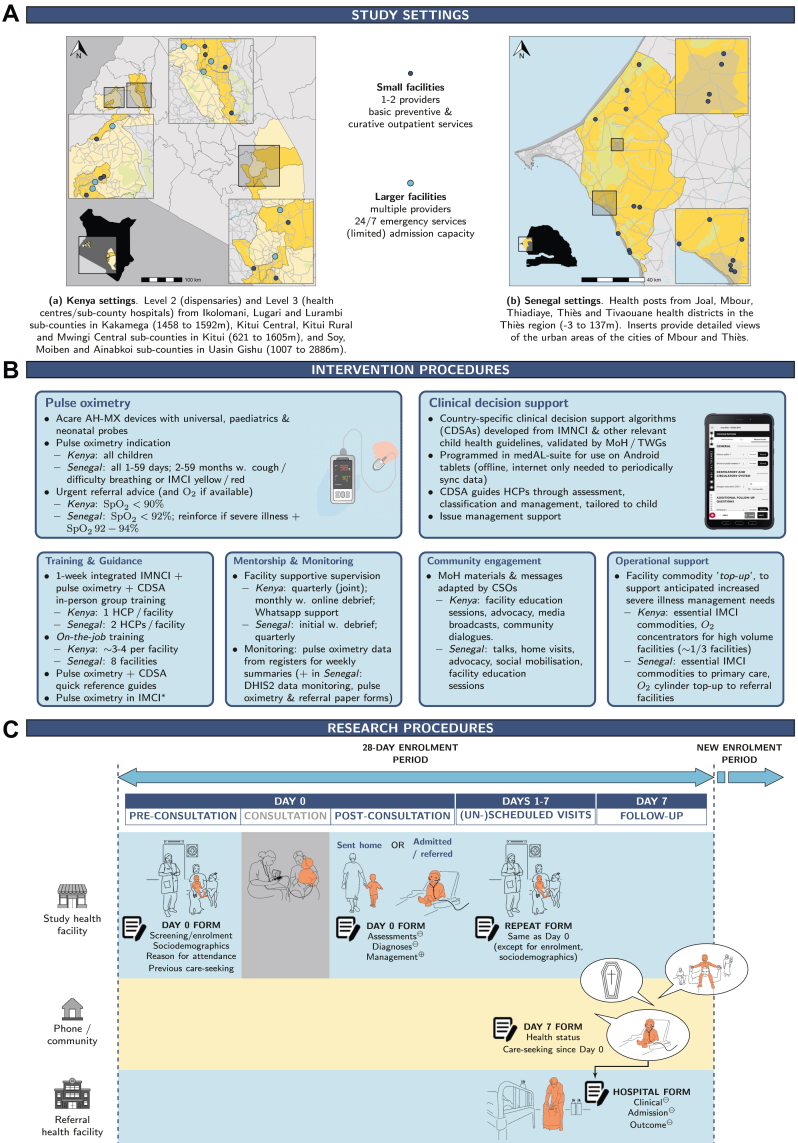
Overview of the study settings (Panel A), implementation (Panel B), and research procedures (Panel C). Panel A: Spatial distribution of facilities by type, with the average altitude of each area provided in metres (m). Panel B: The asterisk (∗) refers to IMCI chart booklet updated to include pulse oximetry according to country specific criteria. Abbreviations: CHMT = Council Health Management Team; CHW = Community Health Workers; CSOs = Civil Society Organisations; TWGs = Technical Working Groups. Panel C: Data sources are indicated as follows: ⊖ = facility records only; ⊕ = both caregivers and facility records; otherwise, from caregivers only.

The study was conducted in government-run primary health care (PHC) facilities. It included level 2 and level 3 facilities in Kakamega, Kitui and Uasin Gishu counties in Kenya, and health posts (i.e., first level in the pyramid of care) in five health districts of the Thiès region in Senegal (Fig. 1A). Facilities were eligible if they provided curative care services for children under-five, had access to oxygen (either onsite or at a referral facility) and electricity. Facilities were excluded if they were inaccessible for significant parts of the year (typically during the rainy season due to seasonal flooding), saw fewer than 20 sick children per month, already systematically used pulse oximetry for sick child consultations, or had another major programmatic or research intervention planned during the study period. Children 0–59 months were eligible if they attended a study facility for an illness or were reported unwell during a routine visit. Children on their first day of life, attending for trauma only, already an inpatient, or enrolled within the preceding 28 days, were excluded.

### Procedures

The TIMCI intervention package was developed collaboratively by the consortium and implemented by PATH in partnership with Ministries of Health (MoHs) in 60 eligible facilities per country, selected through joint discussions and direct assessments. This package included: devices (Acare AH-MX pulse oximeters and tablet-based CDSAs); guidance and training on pulse oximetry, CDSA and IMCI; routine data monitoring; mentorship; community engagement; and operational support (Fig. 1B). All components were adapted to the Senegal and Kenya contexts with review and approval by national clinical expert panels and MoHs. In Kenya, pulse oximetry was indicated for all sick children; urgent referral was recommended for children with SpO_2_ <90%. In Senegal, pulse oximetry was indicated for all 1–59 days and for 2–59 months with cough or difficulty breathing, or a moderate to severe illness (yellow/red IMCI classification); urgent referral was recommended for children with SpO_2_ <92% (to ensure timely referral and optimal care, given the unavailability of oxygen and admission capacity at health posts, while also minimizing unnecessary referrals that could strain the system), and its importance reinforced for children with severe illness and SpO_2_ <95%, who may require oxygen. CDSAs were developed by integrating national IMCI (0–59 day and 2–59 month modules) with other child health guidelines relevant at PHC level.

Study facilities were randomly selected from implementation facilities using proportional sampling to reflect their urban/rural distribution within the study settings. Research data were collected on encrypted Android handheld devices using ODK Collect and stored on ODK Central servers in each country. Children were screened at presentation, and eligible children were enrolled following written informed consent from their caregivers (parents or guardians). Day 0 (D0) data were collected from caregivers before and after the consultation and from facility records (Fig. 1C). Data collected from caregivers included sociodemographics, reason for attendance, previous care-seeking, and management received. No data on ethnicity were collected. Data collected from facility records included assessments performed, diagnoses made, and management provided. Caregiver-reported recovery and care-seeking behaviour were collected by phone on Day 7 (D7). Opportunistic data were recorded for (un-)scheduled follow-up visits at any study facility between Days 1–7. In Senegal, additional data were collected, when available, from referral facility records, for children reported to have attended this level of care.

### Outcomes

Two primary outcomes were assessed at D0: 1) urgent referrals to a higher level of care and 2) antibiotic prescriptions. A child was considered urgently referred (to a hospital or a PHC inpatient ward) if, at D0, advice for urgent referral was documented in facility records. If urgency was unknown, referral was assumed urgent. Antibiotics were defined according to the WHO antibiotic point prevalence survey methodology,^23^ thus only considering antibacterials for systemic use, sourced from caregivers (prescription or medication in-hand) or facility records. Secondary outcomes detailed elsewhere (NCT05065320), included: prevalence of SpO_2_ levels (<90%, 90–91%, and 92–93%); (in-)appropriate antibiotic prescriptions; malaria testing and prescription practices; attendance to a higher level of care by D7; hospitalisations ≤24 h of the D0 consultation, with urgent referral; oxygen administration; severe complications by D7 (death, delayed hospitalisation (>24 h from the D0 consultation) or hospitalisation without urgent referral); caregiver-reported recovery at D7; (un-)scheduled follow-up visits. Pulse oximetry uptake was assessed as the proportion of children with documented SpO_2_ among those for whom it was indicated, i.e., all 1–59 days (both countries), all 2–59 months (Kenya) and 2–59 months with cough, difficulty breathing or moderate/severe illness based on caregiver report or recorded diagnoses (Senegal)^22^; CDSA uptake as the ratio of consultations recorded in the CDSA to the number of enrolled children.

### Statistical analysis

Sample size was calculated separately for Kenya and Senegal, as detailed in Supplement S1. Calculations were based on detecting a ≥50% increase in D0 urgent referrals with 80% power under a type I error of 0.05%, assuming a referral rate of 3.0% in the pre-intervention period (derived from DHIS2, Service Provision Assessment surveys, and facility estimates)^22^ and an intra-cluster correlation (ICC) coefficient of 0.005. The same statistical assumptions allowed for the detection of a clinically meaningful reduction of at least 18.0% in D0 antibiotic prescriptions, assuming a prescription rate of 60.0% in the pre-intervention period and an ICC of 0.05. Due to lower-than-anticipated recruitment in the pre-intervention period, the initial sample size was revised by extending the pre-intervention period from 3 to 6 months in Kenya, and to 8 months in Senegal, and by adding two clusters in each country. The revised sample size required 19 facilities in Kenya and 20 facilities in Senegal, recruiting in each period a minimum of 7429 and 6760 children respectively. Recruitment was continued after meeting the minimum sample size to allow for evaluation of changes over time, particularly those related with the stabilisation of the intervention in the post-intervention period, and overlapping seasonal periods.

Individual country and pooled cross-country intention-to-treat (ITT) analyses were conducted for each period and stratified by age (1–59 days, 2–59 months). Baseline characteristics and outcomes were calculated using R4.1.2 and described with summary statistics. Primary outcomes were assessed using generalised estimating equations for logistic regression (gee R package), with facilities as clusters. Results were estimated in terms of odds ratios (ORs) and risk differences (RDs) derived using a marginal standardization approach (emmeans R package), with a 95% confidence interval (95%CI). Models were adjusted for the following pre-specified potential confounders: age, sex, travel time to facility, illness duration, previous care and treatment, cough, fever, diarrhoea and any danger sign at presentation. Country-level effects were not adjusted for in the modelling of pooled cross-country outcomes as clustering by facilities captured the primary source of variability in the analysis, while also partially accounting for within country correlation, given that each facility was nested within a single country. Modelling for sensitivity analyses and secondary outcomes were performed in a similar way, when numbers allowed. Primary outcomes subgroup analyses were conducted to assess effect modification by age, sex, clinical presentation, and facility location. Pre-specified sensitivity analyses included: considering only the first disease episode per child during the study and using alternative definitions for pre- and post-intervention periods (same annual periods; late post-intervention period), urgent referrals and delayed hospitalisations. These were complemented by a post-hoc per-protocol sensitivity analysis restricted to children who were consulted with CDSA, and, where indicated, received pulse oximetry, in the post-intervention period, as well as a post-hoc analysis of the primary outcomes to account for multiple comparisons using a Bonferroni correction.

### Role of funding source

The funder had no role in study design, data collection, data analysis, data interpretation, or writing of the manuscript.

## Results

Enrolment was conducted from September 13, 2021 to February 8, 2023 in Kenya and August 16, 2021 to March 31, 2023 in Senegal. The intervention package was rolled-out in March 2022 in each country, so that the pre-intervention period lasted approximately seven months, while the post-intervention period lasted approximately nine months. In Senegal, a protracted HCP strike started two weeks after rollout and continued until the end of the study.

### Population

**Fig. 2:**
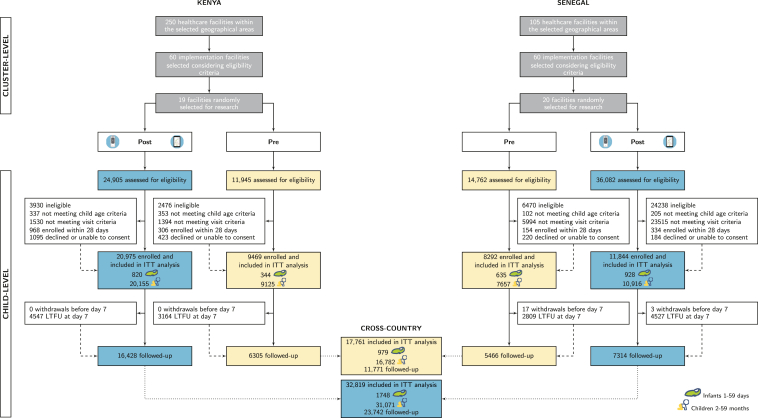
Study flowchart by intervention period, cross- and by country. Numbers are disaggregated by age groups (1–59 days, 2–59 months). Yellow boxes corresponds to the pre-intervention period; blue boxes to the post-intervention period. ITT = intention-to-treat. LTFU = lost-to-follow-up.

The flowchart for the study is detailed in Fig. 2. A total of 50,580 children were enrolled: 17,761 (9469 in Kenya, 8292 in Senegal) in the pre-intervention period, and 32,819 (20,975 in Kenya, 11,844 in Senegal) in the post-intervention period. The characteristics of facilities and recruitment details by type of facilities are available in Supplement S2. Primary outcomes for ITT analyses were available for all enrolled children. Secondary outcomes derived from D7 follow-ups were available for 70.2% of children.

The demographic and clinical characteristics of the study population are summarized in Table 1. Infants 1–59 days represented a larger group in Senegal (7.8%) than in Kenya (3.8%). Characteristics were broadly similar across periods, although the prevalence of previous care-seeking and severe symptoms reported by caregivers was higher pre-than post-intervention (5.7% vs. 2.3% children with previous care at a health facility; 5.9% vs. 3.0% with previous treatment; 12.0% vs. 7.0% with a least one danger sign; 9.5% vs. 5.7% with difficulty breathing). In addition, the prevalence of comorbidities—where data were available, including sickle cell disease in both countries, HIV, and tuberculosis in Kenya—was low (<0.1%).

**Table 1:** Demographic and clinical characteristics of children 1 day to 59 months enrolled in the TIMCI study by intervention period, cross- and by country. Yellow columns correspond to the pre-intervention period; blue to the post-intervention period.

### Intervention uptake

Overall, 58.3% of 1–59 days and 73.4% of 2–59 months with pulse oximetry indication had SpO_2_ values documented in facility records. A marked contrast was observed between countries, with pulse oximetry uptake in Kenya (78.4% in 1–59 days; 82.9% in 2–59 months) nearly double that in Senegal (40.5% in 1–59 days; 44.7% in 2–59 month with pulse oximetry indication). In Senegal, uptake was higher in 2–59 months with a yellow or red IMCI classification based on facility records (58.2%) than with cough/difficulty breathing (44.5%). CDSA uptake followed a similar trend to pulse oximetry: 62.4% of all children; 76.6% in Kenya; 40.5% in Senegal.

### Prevalence of SpO_2_ levels

A small proportion of children had documented SpO_2_ <90%, with a higher prevalence in 1–59 days (1.2%) than in 2–59 months (0.7%). The prevalence of SpO_2_ 90–91% and 92–93% was three to eight-fold higher than prevalence of SpO_2_ <90% and comparable across age groups (3.9% and 5.4% in 1–59 days; 3.3% and 5.8% in 2–59 months). Low SpO_2_ levels were more common in Kenya than in Senegal for all ranges <94% in both age groups (Table 2).

**Table 2:** Pulse oximetry uptake, prevalence of SpO_2_ levels, associated severe complications and cascade of care in children with SpO_2_ values prompting an urgent referral recommendation.

Characteristics	1–59 days			2–59 months		
	Cross-country (n = 1748)	Kenya (n = 820)	Senegal (n = 928)	Cross-country (n = 31,071)	Kenya (n = 20,155)	Senegal (n = 10,916)
Eligible for pulse oximetry, % (n)	100.0% (1748)	100.0% (820)	100.0% (928)	86.4% (26,834)	100.0% (20,155)	61.2% (6679)^f^
Pulse oximetry uptake, % (n/N)^a^	58.3% (1019/1748)	78.4% (643/820)	40.5% (376/928)	73.4% (19,703/26,834)	82.9% (16,717/20,155)	44.7% (2986/6679)
**SpO_2_ levels, % (n)**						
<90%	1.2% (21)	1.7% (14)	0.8% (7)	0.7% (216)	1.0% (203)	0.1% (13)
90–91%	3.9% (68)	8.0% (66)	0.2% (2)	3.3% (1016)	4.9% (986)	0.3% (30)
92–93%	5.4% (95)	10.2% (84)	1.2% (11)	5.8% (1810)	8.5% (1721)	0.8% (89)
SpO_2_ prompting urgent referral^b^	1.3% (23)	1.7% (14)	0.9% (9)	0.8% (246)	1.0% (203)	0.4% (43)
**Severe complications by SpO_2_ levels, % (n/N)**^c^						
≥94%	0.5% (4/820)	0.2% (1/474)	0.9% (3/346)	0.1% (23/16,887)	0.1% (15/13,638)	0.2% (8/3249)
<90%	0.0% (0/21)	0.0% (0/14)	0.0% (0/7)	0.9% (2/216)	1.0% (2/203)	0.0% (0/13)
90–91%	0.0% (0/68)	0.0% (0/66)	0.0% (0/2)	0.4% (4/1016)	0.3% (3/986)	3.3% (1/30)
92–93%	3.2% (3/95)	2.4% (2/84)	9.1% (1/11)	0.2% (4/1810)	0.2% (4/1721)	0.0% (0/89)
<40% (spurious)	–	–	–	0.0% (0/48)	0.0% (0/38)	0.0% (0/10)
Unknown SpO_2_	0.3% (2/744)	0.0% (0/182)	0.4% (2/562)	0.2% (27/11,094)	0.2% (8/3569)	0.3% (19/7525)
**Cascade of care in children with SpO_2_ values prompting urgent referral, % (n/N)**						
Urgent referrals^d^	26.1% (6/23)	35.7% (5/14)	11.1% (1/9)	11.4% (28/246)	12.8% (26/203)	4.7% (2/43)
Hospital attendances among referred^e^	33.3% (2/6)	20.0% (1/5)	100.0% (1/1)	21.4% (6/28)	23.1% (6/26)	0.0% (0/2)^g^
Hospital admissions among referred^e^	33.3% (2/6)	20.0% (1/5)	100.0% (1/1)	17.9% (5/28)	19.2% (5/26)	0.0% (0/2)^g^
Oxygen administration among referred^e^	–	–	0.0% (0/1)^h^	–	–	0.0% (0/2)^g^

a The denominator of each proportion is the population identified in the row immediately above.

b Urgent referral was recommended for SpO_2_ <90% in Kenya and SpO_2_ <92% in Senegal.

c The denominator of each proportion is the corresponding SpO_2_ group.

d The denominator of each proportion is the population with SpO_2_ values prompting an urgent referral recommendation.

e The denominator of each proportion is the population with SpO_2_ values prompting an urgent referral recommendation who was referred at the D0 consultation.

f Estimated, based on caregiver pre-consultation report of cough/difficulty breathing, or documented yellow/red IMCI classification post-consultation.

g In Senegal, the two children 2–59 months with SpO_2_ <92% who were referred (one to Mbour Health Centre and the other to Mbour Hospital) were lost to follow-up at D7. However, a subsequent admission (occurring after the D7 follow-up and thus not included in this analysis) was found for the child referred to Mbour Hospital, which documented the administration of oxygen via nasal cannula.

h In Senegal, the hospital record for the infant aged 1–59 days with SpO_2_ <92%, who was referred to Tivaouane Health Centre and subsequently referred and admitted to Mame Abdou Aziz Sy Dabakh Hospital in Tivaouane, could not be located.

### Primary outcomes

**Fig. 3:**
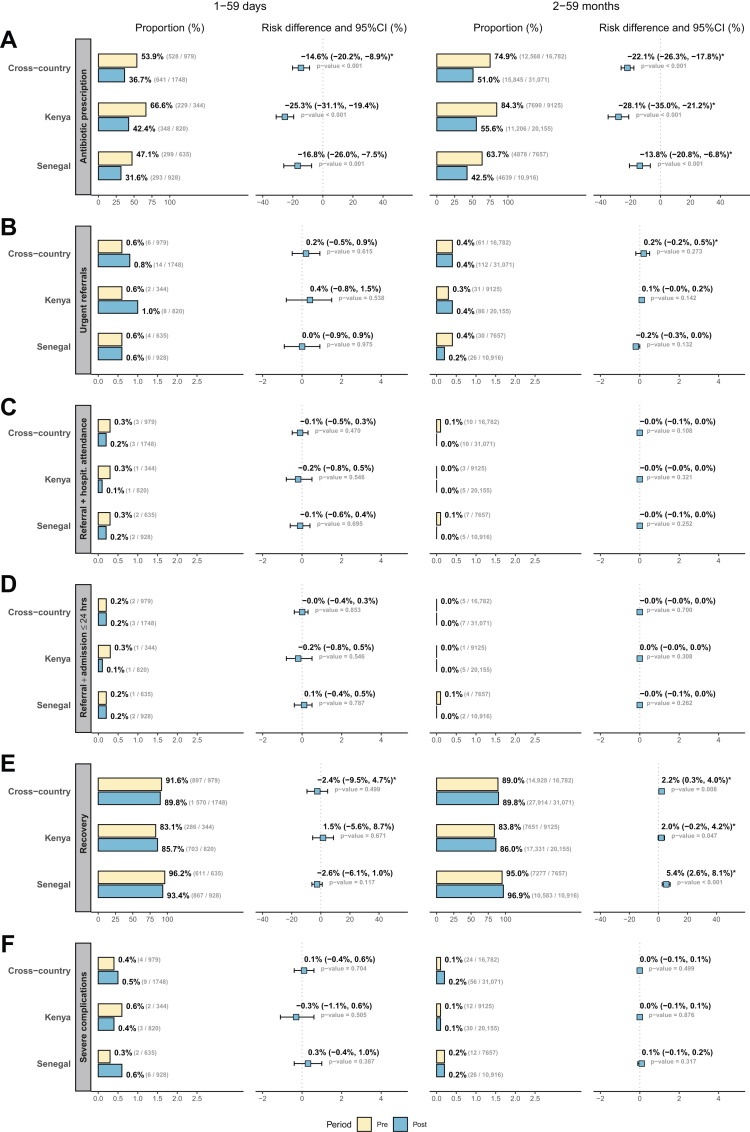
Primary and key secondary outcome proportions and forest plot comparisons between the pre- and post-intervention periods. Yellow corresponds to pre; blue to post. Asterisks (∗) indicate risk differences and 95% confidence intervals resulting from adjusted models (unadjusted models otherwise). A: Antibiotic prescription at the D0 consultation. B: Urgent referral at the D0 consultation. C: Referral on the D0 consultation followed by attendance of a higher level of care. D: Referral on the D0 consultation followed by hospital admission ≤24 h. E: Caregiver-reported recovery by D7. F: Severe complications by D7.

Fewer children than expected were urgently referred to a higher level of care in the pre-intervention period (Fig. 3B), with slightly higher rates in 1–59 days (0.6%) than in 2–59 months (0.4%). Overall referral rates, including both urgent and non-urgent referrals, were still substantially lower (1.5% in 1–59 days and 0.5% in 2–59 months) than the hypothesised 3.0% for urgent referrals alone, which was used to power the study. These rates were consistent with, though lower than, the proportion of children with severe illness recorded in facility registries (2.7% in 1–59 days and 1.5% in 2–59 months). Urgent referral rates remained largely unchanged in the post-intervention period (0.6%, RD 0.2% [−0.5%, 0.9%] in 1–59 days; 0.4%, adjusted RD 0.2% [−0.2%, 0.5%] in 2–59 months). In Kenya, a statistically non-significant increase was observed in both age groups (0.6% vs. 1.0%, RD 0.4% [−0.8%, 1.5%] in 1–59 days; 0.3% vs. 0.4%, RD 0.1% [−0.0%, 0.2%] in 2–59 months). In contrast, in Senegal, urgent referral rates remained stable in 1–59 days (0.6%, RD 0.0% [−0.9%, 0.9%]), while a statistically non-significant reduction was observed in 2–59 months (0.4% vs. 0.2%, RD −0.2% [−0.3%, 0.0%]).

A majority of children were prescribed antibiotics in the pre-intervention period (Fig. 3A), with higher rates in 2–59 months (74.9%) than in 1–59 days (53.9%). Prescription rates were significantly decreased in the post-intervention period, by 14.6 [8.9, 20.2] percentage points in 1–59 days and 22.1 [17.8, 26.3] percentage points in 2–59 months (adjusted models). Although Kenya experienced a greater reduction, its post-intervention prescription rates remained higher (66.6% vs. 42.4%, RD −25.3% [−31.1%, −19.4%] in 1–59 days; 84.3% vs. 55.6%, adjusted RD −28.1% [−35.0%, −21.2%] in 2–59 months) than those in Senegal (47.1% vs. 31.6%, RD −16.8% [−26.0%, −7.5%] in 1–59 days; 63.7% vs. 42.5%, adjusted RD −13.8% [−20.8%, −6.8%] in 2–59 months) for both age groups.

Findings (also including ORs) remained consistent across the different sensitivity analyses (Supplement S3, S7 and S8). The per-protocol sensitivity analysis additionally showed a statistically significant increase for urgent referral in 2–59 months in Kenya (0.3% vs. 0.6%, RD 0.2% [0.0%, 0.4%] in 2–59 months).

### Care cascade and severe complications

Very few children were urgently referred and subsequently reported by caregivers to have attended hospital (3 (0.3%) 1–59 days; 10 (0.1%) 2–59 months; Fig. 3C) or urgently referred and subsequently admitted ≤ 24 h (2 (0.2%) 1–59 days; 6 (0.0%) 2–59 months; Fig. 3D) in the pre-intervention period, with no significant changes in the post-intervention period (RD −0.1% [−0.5%, 0.3%] and −0.0% [−0.4%, 0.3%] in 1–59 days; RD −0.0% [−0.1%, 0.0%] and 0.0% [−0.0%, 0.0%] in 2–59 months).

Only a minority of children with SpO_2_ values prompting an urgent referral recommendation (SpO_2_ <90% in Kenya; SpO_2_ <92% in Senegal) were referred to a higher level of care: 6 out of 23 (26.1%) 1–59 days and 28 out of 246 (11.4%) 2–59 months. Among these children, caregivers reported 2 (33.0%) hospital attendances with admissions in 1–59 days and 6 (21.4%) attendance for 5 (17.9%) admissions in 2–59 months. Incomplete follow-ups in 2–59 months (Supplement S4) and small numbers however limit interpretation. Additionally, it is not possible to comment on whether or not oxygen was administered in Senegal, as no hospital records for these specific children could be found within the D7 follow-up period. However, one of these children was documented to have been admitted after D7, with oxygen administered via nasal cannula.

Few children experienced severe complications in the pre-intervention period as reported by caregivers (0.4% (4) in 1–59 days; 0.1% (24) in 2–59 months; Fig. 3F), with no significant changes in the post-intervention period (RD 0.1% [−0.4%, 0.6%] in 1–59 days; RD 0.0% [−0.1%, 0.1%] in 2–59 months). Most severe complications were attributed to hospitalisations without urgent referral (Supplement S6). Additionally, the proportion of children with severe illness or urgent referrals was comparable between those lost to follow-up and those successfully followed up. No severe complications were observed in 1–59 days with SpO_2_ <90%, though interpretation is limited by incomplete follow-ups (Supplement S4) and small numbers. Severe complications were more common in children with SpO_2_ <94% than in children with SpO_2_ ≥ 94% (Table 2).

### Additional secondary outcomes

Despite antibiotic prescription reduction in the post-intervention period, caregiver-reported recovery rates at D7 remained statistically stable in 1–59 days (89.8% vs. 91.6%, adjusted RD −2.4% [−9.5%, 4.7%]) and increased by 2.2 [0.3; 4.0] percentage points (adjusted RD) in 2–59 months (89.8% vs. 89.0%) compared to the pre-intervention period (Fig. 3E). Further details on the remaining secondary outcomes, which require careful interpretation, are provided in Supplement S5.

## Discussion

This quasi-experimental pre-post study, one of the largest evaluations of pulse oximetry and CDSA effectiveness in primary care, enrolled over 50,000 sick children across 39 facilities in Kenya and Senegal. It did not demonstrate the hypothesised increase in urgent referrals, but confirmed a statistically significant reduction in antibiotic prescriptions. Complementing the TIMCI pragmatic cluster Randomised Controlled Trial (RCT) conducted in Tanzania and India,^24^ these findings can help inform national and global efforts to effectively utilise these interventions.

The lack of a significant increase in urgent referrals, from low pre-intervention proportions (0.6% 1–59 days and 0.4% 2–59 months), may reflect i) limited ‘added value’ of the interventions for severe illness detection, due to low severe illness prevalence, few ‘additional’ severe cases, and/or intervention uptake challenges; ii) barriers to referral; or iii) confounding factors related to the pre-post design. Importantly, cross-country findings mask differing trends in Kenya and Senegal. Though non-significant in either country, there was a trend towards slightly higher post-vs. pre-intervention referral rates in Kenya, similar to the TIMCI RCT^24^ and towards slightly lower rates in Senegal. Though other factors may contribute, it is likely that the protracted post-intervention HCP strike in Senegal influenced referral and other outcomes.

The appropriate actions to take at different levels of care based on SpO_2_ values remain a subject of ongoing debate.^25^ The possibility of low ‘added value’ of pulse oximetry as a single intervention could relate to the relatively low proportion of children documented to meet the SpO_2_ threshold prompting an urgent referral recommendation (SpO_2_ <90% in Kenya; SpO_2_ <92% in Senegal). Cross-country findings (1.3% 1–59 days and 0.8% 2–59 months) were slightly higher than the TIMCI RCT,^24^ and driven by higher prevalence in Kenya, despite Senegal’s higher SpO_2_ threshold. This likely reflects a combination of higher average altitude^26^ and higher pulse oximetry uptake in Kenya, and the inclusion of only lower-level primary care facilities (thus lower illness severity) in Senegal. Aside from the strike in Senegal, uptake differences may relate to the recommendation to use pulse oximetry on all sick children in Kenya, which enabled task-shifting from the consultation to registration. Low CDSA uptake, particularly in Senegal (40.5%, vs. 76.6% in Kenya), may also have resulted in lower than anticipated referral impact.

Several, mostly observational, studies have demonstrated the potential value of pulse oximetry in identifying severe hypoxaemia (SpO_2_ <90%) missed by clinical signs,^27^ supporting better detection of severe pneumonia,^28^ and increasing referral eligibility.^29^^,^^30^ However, these studies included only children with respiratory illness^28^ or suspected pneumonia,^27^^,^^29^ among whom hypoxaemia prevalence is substantially higher (8% non-severe, 41% severe pneumonia).^26^ The lower hypoxaemia prevalence among all sick children attending primary care, seen in this and other studies,^31^ highlights that pulse oximetry may have greater impact on severe illness detection and referral for children with suspected pneumonia. However, when recommending usage criteria, evidence should be considered that restricting pulse oximetry to respiratory illness misses some children with severe hypoxaemia.^31^

Evidence on the impact of CDSAs on referrals are mixed, with significant impact in Nigeria (9.5% CDSA vs. 3.0% paper-based)^15^; Tanzania (1.2% CDSA vs. 1.0% paper-based in one study,^16^ and 6.6% CDSA expanded beyond IMCI, 2.4% IMCI-based CDSA and 0.4% paper-based IMCI in another),^20^ but no impact in Burkina Faso^32^ or South Africa.^33^ This heterogeneity, including with the present study, may be explained by differences in clinical content, technology, implementation approach or wider context.^34^ Further analyses of TIMCI mixed-method studies will explore factors which mediate uptake and effectiveness.

Referral barriers likely also influenced our findings, shaped by contextual, HCP and caregiver factors. Firstly, severe illness exists on a spectrum. For hypoxaemia, mortality is highest in children with both SpO2 <90% and IMCI clinical signs, compared to hypoxaemia or clinical signs alone.^35^^,^^36^ This awareness, whether conscious or subconscious, likely affects decision-making of both HCPs and caregivers. For example, findings from Malawi indicate higher referral rates for children with both hypoxaemia and clinical signs; in Uganda, HCPs reported confidence in managing some children with severe illness without referral,^31^ and caregivers completed referrals more often when danger signs were present.^37^ Beyond or in combination with perceived illness severity, wider barriers including direct and indirect costs, logistical challenges and power dynamics influencing caregiver decision-making act as major influences to referral,^31^^,^^38^^,^^39^ likely contributing to the lack of referral impact seen in this study.

In contrast to referral findings, we observed lower antibiotic prescriptions post-intervention (14.6% for 1–59 days, 22.1% for 2–59 months). This was likely mediated by CDSA-supported guideline adherence, with greater reductions in Kenya where CDSA uptake was higher, though Senegal had lower overall rates both pre- and post-intervention. Similar antibiotic stewardship effects have been reported by other studies,^16^^,^^20^^,^^36^ with no adverse effect on clinical outcomes in those or the present study. However, this impact is not always observed,^15^^,^^40^ including in the TIMCI RCT in Tanzania.^24^ Interestingly, substantial reductions were seen in a parallel cluster RCT in Tanzania^16^ using almost the same CDSA, but with differences in outcome measurement, taking place in different districts and including more extensive HCPs training and mentorship with greater antimicrobial stewardship emphasis, pointing to the importance of study design, contextual and implementation differences.

Despite reductions, post-intervention prescription rates remained above the WHO-benchmarked 30% for primary care, and exceeding estimates suggesting bacterial aetiology for only 10–20% of febrile illness among children.^20^^,^^41^^,^^42^ This underscores the need to address individual, organisational and contextual factors influencing antibiotic prescription decisions.^16^^,^^20^^,^^32^

This study has several limitations. The main limitation relates to the pre-post design, with the potential that outcomes were influenced by the COVID-19 pandemic in the pre-intervention period, the healthcare provider strike in Senegal in the post-intervention period, seasonality, or health system changes beyond the interventions evaluated in this study. Nonetheless, the quasi-experimental design offers advantages over other, largely observational, pulse oximetry studies not powered to evaluate referral. The complex nature of the intervention renders it challenging to attribute causality to single components. The lower-than-expected urgent referral rate suggests that a larger sample size would be required to achieve adequate power for this outcome. As we did not observe consultations, undocumented referrals may have been missed, particularly for conversations in which HCPs recommended referral, but caregivers declined. Similarly, reliance on documentation for SpO_2_ values and severe classification estimation prevents us from fully disentangling whether low referral impact was due to issues of severe illness detection or of referral recommendation. A dedicated analysis on the subpopulation of severely ill children, adjusted for socio-demographic and comorbidity confounders, could provide further insights. However, low follow-up rates at D7 impacted the reporting of severe complications and the search of hospital attendances, therefore limiting our capacity to draw conclusions on severely sick children in this study and in future analyses. Though we used consistent data sources across periods, unlike some CDSA studies,^17^^,^^18^^,^^43^, ^44^, ^45^, ^46^ CDSA introduction may have changed record-keeping practice, potentially affecting outcomes derived from paper-based records, including referrals. Finally, the generalizability of our findings is limited by the study settings in which the data were collected and may not fully reflect the diversity of healthcare contexts in other regions or facilities.

In conclusion, this study makes an important contribution to the literature in demonstrating the intervention’s potential to support antibiotic stewardship. However, the limited impact on referrals, despite the known association of hypoxaemia with mortality, highlights the need to consider where and how to implement pulse oximetry to balance potential impact with feasibility. Importantly, both pulse oximetry and CDSAs need to be implemented with wider consideration of contextual barriers to referral, and strategies to support effective whole systems strengthening to improve child outcomes.

## Contributors

HL, FB, ON, JM, TG, FS, KW, VDA, LFB, MR, MEF and SH were responsible for conceptualization of the study. FB, HL, TG, SC, GL, FN, AM, ON, PMF and JADT were responsible for the methodology of the study. FN, RK, KN, MM, MN and JM were responsible for the implementation of the study in Kenya, and PMF, AT, JADT, NMS and ON in Senegal. MR, MEF, AM, JSMB and MMF were responsible for the intervention implementation, with support from other members of the TIMCI collaborator group. Data were collected by UoN and UCAD research assistants overseen by FJ, KN, PMF and JADT, and hospital data in Senegal was overseen by AT and MC. Data management was led and coordinated by HL globally, and by KN in Kenya and JADT in Senegal. The statistical analysis plan was developed by SC with inputs from TG, FB, HL, GL, PMF, JADT, AT, ON, FN, RK, KN, JM, VDA and KW. The statistical analysis was performed by SC with inputs from TG, HL, GL and FB. Interpretation of findings was conducted collectively through an in-person workshop involving FB, FN, PMF, HL, GL, TG, SC, RK, JADT, NMS, FS, AM, JS, MB, MR, MEF, SH, VDA, KW and discussion with other members of the TIMCI Collaborator Group and external stakeholders. The original draft of the manuscript was prepared by HL, FB, GL, SC, TG and FS, with review and feedback by all other authors. The data reported in the manuscript were accessed and verified by SC, HL, JADT, FN and KN. Visualisations of data presented were prepared by HL, SC and GL with input from TG, FS and FB. Overall oversight was by Principal Investigators KW and VDA globally, ON in Senegal and JM in Kenya, statistical oversight by TG, and clinically by FB globally, PMF in Senegal and RK in Kenya. FS was responsible for research activity planning and execution globally, with PMF responsible in Senegal and FN in Kenya. KW, VDA, MR, ON, JM and other members of the wider TIMCI Collaborator Group were responsible for acquiring funds. The full list of the TIMCI Collaborator Group is available here: https://zenodo.org/communities/timci/about.

## Data sharing statement

De-identified individual children data that underlie the results reported in this manuscript are available through the TIMCI community on Zenodo:

• Kenya: https://doi.org/10.5281/zenodo.12699164.
• Senegal: https://doi.org/10.5281/zenodo.12699143.

These datasets are under restricted access until December 2025. During this period, data will be made available upon reasonable request. Researchers interested in accessing the data should submit a detailed request through the TIMCI community on Zenodo, including a methodologically sound proposal. The study team will review all requests to ensure they meet criteria for data sharing and are scientifically valid. From January 2026, the data will become openly accessible under a Creative Commons Attribution 4.0 International licence (CC-BY 4.0), with no end date.

The study protocol, statistical analysis plan, and analytic code will be available following publication, with no end date, at https://zenodo.org/communities/timci. These materials will be accessible without restriction.

## Declaration of interests

The authors declare no competing interests. All institutes received funding as part of grant n°2019-35-TIMCI, contributing to salaries of co-authors.

## Appendix ASupplementary data

Supplement S8

2024_TIMCI study design

TIMCI_quasi-experimental pre-post study_SAP_v1.1

TIMCI_WHOERC.0003406_v2.3

TIMCI_WHOERC.0003406_v2.4

TIMCI_WHOERC.0003406_v2.5
