# Supplementary material for: Effectiveness of introducing pulse oximetry and clinical decision support algorithms for the management of sick children in primary care in Kenya and Senegal on referral and antibiotic prescription: the TIMCI quasi-experimental pre-post study
Source: eClinicalMedicine. 2025 May 12;83:103196. doi: 10.1016/j.eclinm.2025.103196 (PMC12140026; doi:10.1016/j.eclinm.2025.103196)
Supplement: Supplement S1 [file mmc1.docx]

Supplementary file S1 - Details of the study sample size calculation

Table of contents

[1. Functions for clustered designs 1](#_Toc190641293)

[2. Assumptions 1](#_Toc190641294)

[2.1 Urgent referral assumptions 1](#_Toc190641295)

[2.2 Antibiotic prescription assumptions 1](#_Toc190641296)

[3. Original sample size 1](#_Toc190641297)

[3.1 Kenya 1](#_Toc190641298)

[3.1.1 Urgent referral 1](#_Toc190641299)

[3.1.2 Antibiotic prescription 1](#_Toc190641300)

[3.2 Senegal 1](#_Toc190641301)

[3.2.1 Urgent referral 1](#_Toc190641302)

[3.2.2 Antibiotic prescription 1](#_Toc190641303)

[4. Revised sample size following lower-than-anticipated recruitment rates 1](#_Toc190641304)

[4.1 Kenya 1](#_Toc190641305)

[4.1.1 Urgent referral 1](#_Toc190641306)

[4.1.2 Antibiotic prescription 1](#_Toc190641307)

[4.2 Senegal 1](#_Toc190641308)

[4.2.1 Urgent referral 1](#_Toc190641309)

[4.2.2 Antibiotic prescription 1](#_Toc190641310)

# 1. Functions for clustered designs

These functions calculate the statistical power, minimum detectable difference, and the required number of clusters for a clustered design study

Parameters:

- k = number of clusters
- N = average cluster size (number of subjects per cluster)
- p1 = proportion of the outcome in the pre-intervention phase
- p2 = proportion of the outcome in the post-intervention phase.
- ICC = intra-cluster correlation coefficient (degree of similarity within clusters)
- alpha = significance level, type I error probability (default: 0.05)
- power = statistical power, 1 - type II error probability (default: 0.8)
- m = multiplicity adjustment factor for multiple comparisons (default: 1)

calculate_k <- function(N, p1, p2, ICC, alpha = 0.05, power = 0.8, m = 1) {

 # Adjust alpha for multiple comparisons using Bonferroni correction
 alpha_adj <- alpha / m

 # Calculate the effect size (Cohen's h for proportions)
 # Two proportions from independent populations are compared by considering
 # their difference
 h <- ES.h(p1, p2)

 # Calculate power using the adjusted alpha level
 n <- pwr.2p.test(h = h, sig.level = alpha_adj, power = power)$n

 # Calculate the design effect (DEFF) due to clustering
 # DEFF represents the degree of variance inflation that attributes to cluster
 # sampling
 DEFF <- 1 + (N - 1) * ICC

 # Calculate the number of clusters
 k <- ceiling(n * DEFF / N)

 list(k, paste0("The study requires **", k, "** clusters recruiting ", N, " children each, i.e. ", k * N, " children in total, to detect a statistically significant difference between a proportion of ", sprintf("%.1f", 100*p1), "% at baseline (ICC = ", ICC, ") and a proportion of ", sprintf("%.1f", 100*p2),"% with intervention, with α = ", alpha, " and power = ", sprintf("%.1f", 100*power),"%."))
}

invert_cohen_h <- function(h, p1, increase) {

 if (increase){
 p2 <- sin(asin(sqrt(p1)) + h / 2)^2
 } else{
 p2 <- sin(asin(sqrt(p1)) - h / 2)^2
 }
 p2
}

calculate_diff <- function(k, N, p1, ICC, alpha = 0.05, power = 0.8, m = 1, increase = TRUE) {

 # Adjust alpha for multiple comparisons using Bonferroni correction
 alpha_adj <- alpha / m

 # Calculate the design effect (DEFF) due to clustering
 # DEFF represents the degree of variance inflation that attributes to cluster
 # sampling
 DEFF <- 1 + (N - 1) * ICC

 # Adjust the sample size for clustering
 n_adjusted <- k * N / DEFF

 # Calculate power using the adjusted sample size
 h <- pwr.2p.test(n = n_adjusted, sig.level = alpha_adj, power = power)$h

 # Invert effect size to retrieve the minimal difference
 p2 <- invert_cohen_h(h, p1, increase)
 d <- abs(p2 - p1)/ p1
 list(d, paste0("With ", k, " clusters recruiting ", N, " children each, the minimum detectable difference is **", sprintf("%.1f", 100*d), "%** from a proportion of ", sprintf("%.1f", 100*p1), "% at baseline (ICC = ", ICC, "), with α = ", alpha, " and power = ", sprintf("%.1f", 100*power),"%."))
}

calculate_power <- function(k, N, p1, p2, ICC, alpha = 0.05, m = 1) {

 # Adjust alpha for multiple comparisons using Bonferroni correction
 alpha_adj <- alpha / m

 # Calculate the effect size (Cohen's h for proportions)
 # two proportions from independent populations are compared by considering
 # their difference
 h <- ES.h(p1, p2)

 # Calculate the design effect (DEFF) due to clustering
 # DEFF represents the degree of variance inflation that attributes to cluster
 # sampling
 DEFF <- 1 + (N - 1) * ICC

 # Adjust the sample size for clustering
 n_adjusted <- k * N / DEFF

 # Calculate power using the adjusted sample size
 power <- pwr.2p.test(h = h, n = n_adjusted, sig.level = alpha_adj)$power
 list(power, paste0("Power is ", power))
}

# 2. Assumptions

alpha <- 0.05
pw <- 0.8
m <- 1

## 2.1 Urgent referral assumptions

pre_referral <- 0.03 # 3.0% in the pre-intervention period
post_referral <- pre_referral*(1+0.5) # 50% increase in the post-intervention period (hypothesised)
icc_referral <- 0.005 # ICC = 0.005

## 2.2 Antibiotic prescription assumptions

pre_antibio <- 0.60 # 60% in the pre-intervention period
icc_antibio <- 0.05 # ICC = 0.05

# 3. Original sample size

## 3.1 Kenya

avg_by_cluster_kenya <- 690 # average recruitment rate per facility

### 3.1.1 Urgent referral

k_kenya <- calculate_k(N = avg_by_cluster_kenya,
 p1 = pre_referral,
 p2 = post_referral,
 ICC = icc_referral,
 power = pw,
 alpha = alpha,
 m = m)
cat(k_kenya[[2]])

The study requires **17** clusters recruiting 690 children each, i.e. 11730 children in total, to detect a statistically significant difference between a proportion of 3.0% at baseline (ICC = 0.005) and a proportion of 4.5% with intervention, with α = 0.05 and power = 80.0%.

### 3.1.2 Antibiotic prescription

diff_antibio_kenya <- calculate_diff(k = k_kenya[[1]],
 N = avg_by_cluster_kenya,
 p1 = pre_antibio,
 ICC = icc_antibio,
 power = pw,
 alpha = alpha,
 m = m,
 increase = FALSE)
cat(diff_antibio_kenya[[2]])

With 17 clusters recruiting 690 children each, the minimum detectable difference is **18.0%** from a proportion of 60.0% at baseline (ICC = 0.05), with α = 0.05 and power = 80.0%.

## 3.2 Senegal

avg_by_cluster_senegal <- 510 # average recruitment rate per facility

### 3.2.1 Urgent referral

k_senegal <- calculate_k(N = avg_by_cluster_senegal,
 p1 = pre_referral,
 p2 = post_referral,
 ICC = icc_referral,
 power = pw,
 alpha = alpha,
 m = m)
cat(k_senegal[[2]])

The study requires **18** clusters recruiting 510 children each, i.e. 9180 children in total, to detect a statistically significant difference between a proportion of 3.0% at baseline (ICC = 0.005) and a proportion of 4.5% with intervention, with α = 0.05 and power = 80.0%.

### 3.2.2 Antibiotic prescription

diff_antibio_senegal <- calculate_diff(k = k_senegal[[1]],
 N = avg_by_cluster_senegal,
 p1 = pre_antibio,
 ICC = icc_antibio,
 power = pw,
 alpha = alpha,
 m = m,
 increase = FALSE)
cat(diff_antibio_senegal[[2]])

With 18 clusters recruiting 510 children each, the minimum detectable difference is **17.6%** from a proportion of 60.0% at baseline (ICC = 0.05), with α = 0.05 and power = 80.0%.

# 4. Revised sample size following lower-than-anticipated recruitment rates

## 4.1 Kenya

avg_by_cluster_kenya <- 391 # revised average recruitment rate per facility

### 4.1.1 Urgent referral

k_kenya <- calculate_k(N = avg_by_cluster_kenya,
 p1 = pre_referral,
 p2 = post_referral,
 ICC = icc_referral,
 power = pw,
 m = m)
cat(k_kenya[[2]])

The study requires **19** clusters recruiting 391 children each, i.e. 7429 children in total, to detect a statistically significant difference between a proportion of 3.0% at baseline (ICC = 0.005) and a proportion of 4.5% with intervention, with α = 0.05 and power = 80.0%.

### 4.1.2 Antibiotic prescription

diff_antibio_kenya <- calculate_diff(k = k_kenya[[1]],
 N = avg_by_cluster_kenya,
 p1 = pre_antibio,
 ICC = icc_antibio,
 alpha = alpha,
 power = pw,
 m = m,
 increase = FALSE)
cat(diff_antibio_kenya[[2]])

With 19 clusters recruiting 391 children each, the minimum detectable difference is **17.2%** from a proportion of 60.0% at baseline (ICC = 0.05), with α = 0.05 and power = 80.0%.

## 4.2 Senegal

avg_by_cluster_senegal <- 338 # revised average recruitment rate per facility

### 4.2.1 Urgent referral

k_senegal <- calculate_k(N = avg_by_cluster_senegal,
 p1 = pre_referral,
 p2 = post_referral,
 ICC = icc_referral,
 power = pw,
 m = m)
cat(k_senegal[[2]])

The study requires **20** clusters recruiting 338 children each, i.e. 6760 children in total, to detect a statistically significant difference between a proportion of 3.0% at baseline (ICC = 0.005) and a proportion of 4.5% with intervention, with α = 0.05 and power = 80.0%.

### 4.2.2 Antibiotic prescription

diff_antibio_senegal <- calculate_diff(k = k_senegal[[1]],
 N = avg_by_cluster_senegal,
 p1 = pre_antibio,
 ICC = icc_antibio,
 alpha = alpha,
 power = pw,
 m = m,
 increase = FALSE)
cat(diff_antibio_senegal[[2]])

With 20 clusters recruiting 338 children each, the minimum detectable difference is **16.9%** from a proportion of 60.0% at baseline (ICC = 0.05), with α = 0.05 and power = 80.0%.
