# Supplementary material for: Effectiveness of introducing pulse oximetry and clinical decision support algorithms for the management of sick children in primary care in Kenya and Senegal on referral and antibiotic prescription: the TIMCI quasi-experimental pre-post study
Source: eClinicalMedicine. 2025 May 12;83:103196. doi: 10.1016/j.eclinm.2025.103196 (PMC12140026; doi:10.1016/j.eclinm.2025.103196)
Supplement: Supplement S2 [file mmc2.docx]

## Supplementary file S2 – Characteristics of facilities and recruitment by facilities

### Characteristics of recruiting facilities – Kenya

| Variables | Statistics/Categories | Pre-intervention (n=19) | Post-intervention (n=19) |
| --- | --- | --- | --- |
| District | Kakamega | 36.8% (7) | 36.8% (7) |
|  | Kitui | 26.3% (5) | 26.3% (5) |
|  | Uasin Gishu | 36.8% (7) | 36.8% (7) |
| Location | Rural | 52.6% (10) | 52.6% (10) |
|  | Urban | 47.4% (9) | 47.4% (9) |
| Type | Dispensary | 57.9% (11) | 57.9% (11) |
|  | Health Center | 42.1% (8) | 42.1% (8) |
| Number children per Dispensary | Median, min-max | 408, 245-1169 | 587, 385-2181 |
| Number children per Health center | Median, min-max | 475.5, 228-1026 | 1295.5, 653-2181 |

### Distribution of children by characteristics of recruiting facilities – Kenya

| Variables | Statistics/Categories | Pre-intervention (n=9469) | Post-intervention (n=20975) |
| --- | --- | --- | --- |
| District | Kakamega | 35.5% (3359) | 40.2% (8434) |
|  | Kitui | 17.8% (1688) | 14.2% (2987) |
|  | Uasin Gishu | 46.7% (4422) | 45.5% (9554) |
| Location | Rural | 56.0% (5301) | 52.3% (10964) |
|  | Urban | 44.0% (4168) | 47.7% (10011) |
| Type | Dispensary | 55.4% (5244) | 46.4% (9736) |
|  | Health Center | 44.6% (4225) | 53.6% (11239) |

### Characteristics of recruiting facilities – Senegal

| Variables | Statistics/Categories | Pre-intervention (n=20) | Post-intervention (n=20) |
| --- | --- | --- | --- |
| District | Joal | 10.0% (2) | 10.0% (2) |
|  | Mbour | 40.0% (8) | 40.0% (8) |
|  | Thiadiaye | 10.0% (2) | 10.0% (2) |
|  | Thies | 20.0% (4) | 20.0% (4) |
|  | Tivaouane | 20.0% (4) | 20.0% (4) |
| Location | Rural | 30.0% (6) | 30.0% (6) |
|  | Urban | 70.0% (14) | 70.0% (14) |
| Type | health post | 100.0% (20) | 100.0% (20) |
| Number children per Health Post | Median, min-max | 414.5, 101-809 | 576, 132-1211 |

### Distribution of children by characteristics of recruiting facilities – Senegal

| Variables | Statistics/Categories | Pre-intervention (n=8292) | Post-intervention (n=11844) |
| --- | --- | --- | --- |
| District | Joal | 8.7% (718) | 13.1% (1553) |
|  | Mbour | 35.9% (2980) | 41.8% (4949) |
|  | Thiadiaye | 7.6% (633) | 9.0% (1069) |
|  | Thies | 23.4% (1937) | 12.6% (1491) |
|  | Tivaouane | 24.4% (2024) | 23.5% (2782) |
| Location | Rural | 30.1% (2496) | 31.7% (3749) |
|  | Urban | 69.9% (5796) | 68.3% (8095) |
| Type | health post | 100.0% (8292) | 100.0% (11844) |
