# Supplementary material for: Effectiveness of introducing pulse oximetry and clinical decision support algorithms for the management of sick children in primary care in Kenya and Senegal on referral and antibiotic prescription: the TIMCI quasi-experimental pre-post study
Source: eClinicalMedicine. 2025 May 12;83:103196. doi: 10.1016/j.eclinm.2025.103196 (PMC12140026; doi:10.1016/j.eclinm.2025.103196)
Supplement: Supplement S3 [file mmc3.docx]

## Supplementary file S3 – Summary of primary outcome results

The primary outcomes are assessed using generalised estimating equation for logistic regression, with facilities as clusters. Estimates for the pre-post effect on the outcomes are shown in terms of odds ratio and risk difference (with the pre-intervention period being the reference category) and their associated 95% confidence intervals. Results from both univariate and multivariate models are presented when numbers allowed. Not estimable effects are denoted in the summary table below as “NE”.

Multivariate models are adjusted for age, sex, travel time to facility, illness duration, previous care and treatment, cough, fever, diarrhoea and danger signs.

### Summary of primary outcomes results - infants under 2 months of age

| Outcome | Analysis | N (%) Pre-intervention | N (%) Post-intervention | Unadjusted | p-value | Adjusted | p-value |
| --- | --- | --- | --- | --- | --- | --- | --- |
| Urgent referrals | | | | | | | |
| Primary analysis | Combined | 6 (0.6%) | 14 (0.8%) | 1.309 (0.458, 3.739) 0.2% (-0.5%, 0.9%) | 0.615 | NE | - |
|  | Kenya | 2 (0.6%) | 8 (1.0%) | 1.686 (0.319, 8.899) 0.4% (-0.8%, 1.5%) | 0.538 | NE | - |
|  | Senegal | 4 (0.6%) | 6 (0.6%) | 1.023 (0.255, 4.104) 0.0% (-0.9%, 0.9%) | 0.975 | NE |  |
| Sensitivity analysis-first encounters | Combined | 6 (0.6%) | 13 (0.8%) | 1.233 (0.436, 3.488) 0.1% (-0.6%, 0.8%) | 0.694 | NE | - |
|  | Kenya | 2 (0.6%) | 8 (1.0%) | 1.687 (0.322, 8.847) 0.4% (-0.8%, 1.6%) | 0.536 | NE | - |
|  | Senegal | 4 (0.6%) | 5 (0.5%) | 0.890 (0.214, 3.699) -0.1% (-0.9%, 0.8%) | 0.873 | NE |  |
| Sensitivity analysis-referral caregiver or registry | Combined | 6 (0.6%) | 18 (1.0%) | 1.681 (0.600, 4.712) 0.4% (-0.4%, 1.2%) | 0.324 | NE | - |
|  | Kenya | 2 (0.6%) | 13 (1.6%) | 2.956 (0.540, 16.181) 1.0% (-0.3%, 2.4%) | 0.211 | NE | - |
|  | Senegal | 4 (0.6%) | 5 (0.5%) | 0.852 (0.200, 3.627) -0.1% (-0.9%, 0.8%) | 0.829 | NE |  |
| Sensitivity analysis-referral caregiver and registry | Combined | - | - | - | - | - | - |
|  | Kenya | 2 (0.6%) | 4 (0.5%) | 0.851 (0.130, 5.587) -0.1% (-1.0%, 0.9%) | 0.867 | NE | - |
|  | Senegal | - | - | - | - | - | - |
| Sensitivity analysis-pre-post overlapping period of the year | Combined | 6 (0.7%) | 9 (1.0%) | 1.437 (0.486, 4.245) 0.3% (-0.6%, 1.1%) | 0.512 | NE | - |
|  | Kenya | 2 (0.7%) | 5 (1.5%) | 2.039 (0.346, 12.027) 0.7% (-1.0%, 2.4%) | 0.431 | NE | - |
|  | Senegal | 4 (0.7%) | 4 (0.7%) | 1.040 (0.245, 4.418) 0.0% (-0.9%, 1.0%) | 0.958 | NE |  |
| Sensitivity analysis-pre-post based on conutry-specific key dates | Combined | 6 (0.6%) | 7 (0.9%) | 1.476 (0.486, 4.484) 0.3% (-0.5%, 1.1%) | 0.492 | NE | - |
|  | Kenya | 2 (0.6%) | 5 (2.0%) | 3.673 (0.589, 22.896) 1.4% (-0.6%, 3.3%) | 0.163 | NE | - |
|  | Senegal | 4 (0.6%) | 2 (0.4%) | 0.598 (0.110, 3.265) -0.3% (-1.1%, 0.6%) | 0.553 | NE |  |
| Antibiotics prescription | | | | | | | |
| Primary analysis | Combined | 528 (53.9%) | 641 (36.7%) | 0.434 (0.333, 0.565) -20.2% (-26.4%, -14.1%) | <0.001 | 0.450 (0.358, 0.565) -14.6% (-20.2%, -8.9%) | <0.001 |
|  | Kenya | 229 (66.6%) | 348 (42.4%) | 0.354 (0.274, 0.457) -25.3% (-31.1%, -19.4%) | <0.001 | NE | - |
|  | Senegal | 299 (47.1%) | 293 (31.6%) | 0.481 (0.311, 0.743) -16.8% (-26.0%, -7.5%) | 0.001 | NE | - |
| Sensitivity analysis-first encounters | Combined | 520 (53.7%) | 633 (36.7%) | 0.439 (0.340, 0.566) -20.0% (-26.0%, -14.0%) | <0.001 | 0.455 (0.364, 0.568) -14.4% (-20.0%, -8.8%) | <0.001 |
|  | Kenya | 226 (66.3%) | 346 (42.6%) | 0.360 (0.281, 0.462) -24.9% (-30.6%, -19.2%) | <0.001 | NE | - |
|  | Senegal | 294 (46.8%) | 287 (31.5%) | 0.484 (0.318, 0.736) -16.6% (-25.6%, -7.7%) | 0.001 | NE |  |
| Sensitivity analysis-pre-post overlapping period of the year | Combined | 465 (52.8%) | 329 (35.9%) | 0.450 (0.331, 0.611) -19.3% (-26.4%, -12.2%) | <0.001 | NE | - |
|  | Kenya | 174 (64.7%) | 127 (37.1%) | 0.303 (0.231, 0.398) -29.0% (-35.2%, -22.7%) | <0.001 | NE | - |
|  | Senegal | 291 (47.5%) | 202 (35.1%) | 0.555 (0.369, 0.836) -13.9% (-23.0%, -4.7%) | 0.005 | NE |  |
| Sensitivity analysis-pre-post based on conutry-specific key dates | Combined | 528 (53.9%) | 263 (33.7%) | 0.405 (0.285, 0.575) -21.7% (-29.7%, -13.8%) | <0.001 | NE | - |
|  | Kenya | 229 (66.6%) | 89 (35.2%) | 0.260 (0.177, 0.382) -32.5% (-41.1%, -23.9%) | <0.001 | NE | - |
|  | Senegal | 299 (47.1%) | 174 (33.0%) | 0.513 (0.319, 0.828) -15.5% (-25.8%, -5.2%) | 0.006 | NE |  |
| Subgroup-without cough or difficulty breathing | Combined | 188 (39.5%) | 260 (30.4%) | 0.581 (0.436, 0.773) -12.3% (-18.6%, -6.0%) | <0.001 | 0.595 (0.447, 0.792) -8.8% (-14.0%, -3.6%) | <0.001 |
| Subgroup-with cough or difficulty breathing | Combined | 340 (67.6%) | 381 (42.6%) | 0.336 (0.238, 0.474) -26.5% (-34.5%, -18.6%) | <0.001 | NE |  |

### Summary of primary outcomes results - children 2-59 months of age

| Outcome | Analysis | N (%) Pre-intervention | N (%) Post-intervention | Unadjusted | p-value | Adjusted | p-value |
| --- | --- | --- | --- | --- | --- | --- | --- |
| Urgent referrals | | | | | | | |
| Primary analysis | Combined | 61 (0.4%) | 112 (0.4%) | 0.987 (0.735, 1.325) -0.0% (-0.1%, 0.1%) | 0.929 | 1.202 (0.865, 1.669) 0.2% (-0.2%, 0.5%) | 0.273 |
|  | Kenya | 31 (0.3%) | 86 (0.4%) | 1.262 (0.925, 1.722) 0.1% (-0.0%, 0.2%) | 0.142 | NE | - |
|  | Senegal | 30 (0.4%) | 26 (0.2%) | 0.605 (0.315, 1.164) -0.2% (-0.3%, 0.0%) | 0.132 | NE |  |
| Sensitivity analysis-first encounters | Combined | 57 (0.4%) | 94 (0.4%) | 1.026 (0.760, 1.385) 0.0% (-0.1%, 0.1%) | 0.868 | 1.239 (0.882, 1.741) 0.2% (-0.2%, 0.6%) | 0.217 |
|  | Kenya | 28 (0.3%) | 74 (0.4%) | 1.331 (0.941, 1.884) 0.1% (-0.0%, 0.3%) | 0.106 | NE | - |
|  | Senegal | 29 (0.4%) | 20 (0.2%) | 0.596 (0.322, 1.103) -0.2% (-0.4%, 0.0%) | 0.099 | NE |  |
| Sensitivity analysis-referral caregiver or registry | Combined | 61 (0.4%) | 118 (0.4%) | 1.031 (0.754, 1.409) 0.0% (-0.1%, 0.1%) | 0.850 | 1.269 (0.896, 1.795) 0.2% (-0.2%, 0.7%) | 0.179 |
|  | Kenya | 31 (0.3%) | 93 (0.5%) | 1.346 (0.959, 1.890) 0.1% (-0.0%, 0.3%) | 0.086 | NE | - |
|  | Senegal | 30 (0.4%) | 25 (0.2%) | 0.591 (0.299, 1.171) -0.2% (-0.3%, 0.0%) | 0.132 | NE |  |
| Sensitivity analysis-referral caregiver and registry | Combined | - | - | - | - | - | - |
|  | Kenya | 31 (0.3%) | 45 (0.2%) | 0.679 (0.480, 0.959) -0.1% (-0.2%, -0.0%) | 0.028 | NE | - |
|  | Senegal | - | - | - | - | - | - |
| Sensitivity analysis-pre-post overlapping period of the year | Combined | 52 (0.4%) | 55 (0.4%) | 1.033 (0.650, 1.642) 0.0% (-0.2%, 0.2%) | 0.890 | NE | - |
|  | Kenya | 22 (0.3%) | 39 (0.4%) | 1.403 (0.825, 2.384) 0.1% (-0.1%, 0.3%) | 0.211 | NE | - |
|  | Senegal | 30 (0.4%) | 16 (0.3%) | 0.671 (0.266, 1.694) -0.1% (-0.4%, 0.1%) | 0.398 | NE |  |
| Sensitivity analysis-pre-post based on conutry-specific key dates | Combined | 61 (0.4%) | 36 (0.3%) | 0.834 (0.523, 1.329) -0.1% (-0.2%, 0.1%) | 0.445 | NE | - |
|  | Kenya | 31 (0.3%) | 27 (0.4%) | 1.163 (0.686, 1.972) 0.1% (-0.1%, 0.3%) | 0.574 | NE | - |
|  | Senegal | 30 (0.4%) | 9 (0.2%) | 0.447 (0.184, 1.085) -0.2% (-0.4%, -0.0%) | 0.075 | NE |  |
| Subgroup-location rural | Combined | 22 (0.3%) | 53 (0.4%) | 1.238 (0.775, 1.976) 0.1% (-0.1%, 0.2%) | 0.371 | NE | - |
| Subgroup-location urban | Combined | 39 (0.4%) | 59 (0.3%) | 0.838 (0.594, 1.182) -0.1% (-0.2%, 0.1%) | 0.313 | 0.838 (0.594, 1.182) -0.1% (-0.2%, 0.1%) | 0.313 |
| Antibiotics prescription | | | | | | | |
| Primary analysis | Combined | 12568 (74.9%) | 15845 (51.0%) | 0.327 (0.259, 0.413) -26.1% (-31.4%, -20.8%) | <0.001 | 0.358 (0.285, 0.450) -22.1% (-26.3%, -17.8%) | <0.001 |
|  | Kenya | 7690 (84.3%) | 11206 (55.6%) | 0.235 (0.169, 0.328) -29.5% (-36.7%, -22.3%) | <0.001 | 0.300 (0.219, 0.412) -28.1% (-35.0%, -21.2%) | <0.001 |
|  | Senegal | 4878 (63.7%) | 4639 (42.5%) | 0.431 (0.308, 0.603) -20.7% (-28.8%, -12.5%) | <0.001 | 0.492 (0.353, 0.687) -13.8% (-20.8%, -6.8%) | <0.001 |
| Sensitivity analysis-first encounters | Combined | 11570 (75.0%) | 12795 (51.6%) | 0.328 (0.258, 0.417) -25.9% (-31.3%, -20.5%) | <0.001 | 0.362 (0.286, 0.458) -21.9% (-26.4%, -17.5%) | <0.001 |
|  | Kenya | 7084 (84.5%) | 9453 (56.6%) | 0.238 (0.168, 0.337) - 29.0% (-36.4%, -21.7%) | <0.001 | 0.298 (0.218, 0.409) -28.4% (-35.7%, -21.2%) | <0.001 |
|  | Senegal | 4486 (63.6%) | 3342 (41.3%) | 0.430 (0.307, 0.603) -20.7% (-28.9%, -12.6%) | <0.001 | 0.490 (0.346, 0.694) -14.8% (-21.2%, -8.4%) | <0.001 |
| Sensitivity analysis-pre-post overlapping period of the year | Combined | 10476 (73.1%) | 7680 (51.2%) | 0.362 (0.286, 0.457) -23.8% (-29.2%, -18.4%) | <0.001 | 0.406 (0.316, 0.521) -19.4% (-24.0%, -14.9%) | <0.001 |
|  | Kenya | 5702 (83.2%) | 4837 (54.5%) | 0.244 (0.173, 0.344) - 29.6% (-37.4%, -21.9%) | <0.001 | 0.325 (0.220, 0.480) -25.4% (-32.6%, -18.3%) | <0.001 |
|  | Senegal | 4774 (63.8%) | 2843 (46.4%) | 0.506 (0.384, 0.667) -16.7% (-23.5%, -9.9%) | <0.001 | 0.567 (0.415, 0.776) -11.0% (-18.4%, -3.5%) | <0.001 |
| Sensitivity analysis-pre-post based on conutry-specific key dates | Combined | 12568 (74.9%) | 5788 (49.1%) | 0.324 (0.253, 0.414) -26.2% (-31.9%, -20.6%) | <0.001 | 0.372 (0.283, 0.489) -20.3% (-25.1%, -15.5%) | <0.001 |
|  | Kenya | 7690 (84.3%) | 3487 (53.0%) | 0.216 (0.149, 0.312) -31.3% (-39.3%, -23.3%) | <0.001 | 0.305 (0.199, 0.468) -25.2% (-31.9%, -18.5%) | <0.001 |
|  | Senegal | 4878 (63.7%) | 2301 (44.1%) | 0.459 (0.337, 0.625) -19.1% (-26.7%, -11.5%) | <0.001 | 0.532 (0.370, 0.766) -13.0% (-19.9%, -6.0%) | 0.001 |
| Subgroup-age 2 to 12 months | Combined | 3880 (74.4%) | 4744 (45.7%) | 0.271 (0.208, 0.354) -30.8% (-36.7%, -24.9%) | <0.001 | 0.287 (0.221, 0.372) -26.5% (-32.3%, -20.8%) | <0.001 |
| Subgroup-age 13 to 59 months | Combined | 8688 (75.1%) | 11101 (53.6%) | 0.357 (0.285, 0.449) -23.7% (-28.9%, -18.6%) | <0.001 | 0.399 (0.320, 0.498) -19.3% (-23.3%, -15.3%) | <0.001 |
| Subgroup-sex male | Combined | 6646 (75.2%) | 8358 (51.6%) | 0.330 (0.260, 0.418) -25.8% (-31.1%, -20.4%) | <0.001 | 0.361 (0.287, 0.454) -21.8% (-26.1%, -17.5%) | <0.001 |
| Subgroup-sex female | Combined | 5908 (74.5%) | 7468 (50.3%) | 0.324 (0.256, 0.410) -26.4% (-31.8%, -21.0%) | <0.001 | 0.352 (0.280, 0.443) -22.5% (-26.9%, -18.1%) | <0.001 |
| Subgroup-sex unknown | Combined | 14 (73.7%) | 19 (76.0%) | 1.171 (0.265, 5.183) 2.9% (-25.0%, 30.9%) | 0.835 | NE | - |
| Subgroup-without cough or difficulty breathing | Combined | 4290 (65.5%) | 5695 (44.8%) | 0.414 (0.340, 0.503) -21.6% (-26.3%, -17.0%) | <0.001 | 0.432 (0.354, 0.527) -18.3% (-22.2%, -14.4%) | <0.001 |
| Subgroup-with cough or difficulty breathing | Combined | 8278 (80.9%) | 10150 (55.3%) | 0.269 (0.199, 0.363) -28.5% (-34.8%, -22.3%) | <0.001 | 0.279 (0.205, 0.380) -26.9% (-33.5%, -20.4%) | <0.001 |
