# Supplementary material for: Effectiveness of introducing pulse oximetry and clinical decision support algorithms for the management of sick children in primary care in Kenya and Senegal on referral and antibiotic prescription: the TIMCI quasi-experimental pre-post study
Source: eClinicalMedicine. 2025 May 12;83:103196. doi: 10.1016/j.eclinm.2025.103196 (PMC12140026; doi:10.1016/j.eclinm.2025.103196)
Supplement: Supplement S4 [file mmc4.docx]

## Supplementary file S4 – Follow-up rates and hospital records availability for hypoxaemia-related outcomes

|  | **1-59 days** | | | **2-59 months** | | |
| --- | --- | --- | --- | --- | --- | --- |
| Characteristics | Cross-country (n = 1 748) | Kenya (n = 820) | Senegal (n = 928) | Cross-country (n = 31 071) | Kenya (n = 20 155) | Senegal (n = 10 916) |
| **Follow-up rate by SpO2 group, % (n/N)*^3^*** | | | | | | |
| Day 7 follow-up in ≥94% | 75·0% (615/820) | 77·8% (369/474) | 71·1% (246/346) | 75·0% (12 669/16 887) | 78·4% (10 692/13 638) | 60·8% (1 977/3 249) |
| Day 7 follow-up in <90% | 90·5% (19/21) | 100·0% (14/14) | 71·4% (5/7) | 79·6% (172/216) | 81·3% (165/203) | 53·8% (7/13) |
| Day 7 follow-up in 90-91% | 58·8% (40/68) | 59·1% (39/66) | 50·0% (1/2) | 74·9% (761/1 016) | 75·1% (740/986) | 70·0% (21/30) |
| Day 7 follow-up in 92-93% | 74·7% (71/95) | 76·2% (64/84) | 63·6% (7/11) | 78·4% (1 419/1 810) | 78·8% (1 357/1 721) | 69·7% (62/89) |
| Day 7 follow-up in < 40% (spurious) |  |  |  | 89·6% (43/48) | 97·4% (37/38) | 60·0% (6/10) |
| Day 7 follow-up in unknown SpO2 | 67·4% (502/744) | 75·8% (138/182) | 64·8% (364/562) | 67·0% (7 431/11 084) | 78·8% (2 813/3 569) | 61·4% (4 618/7 525) |
| **Day 7 and hospital follow-up rates in referred children with SpO_2_ values prompting urgent referral, % (n/N)** | | | | | | |
| Day 7 follow-up among referred | 100·0% (6/6) | 100·0% (5/5) | 100·0% (1/1) | 67·9% (19/28) | 73·1% (19/26) | 0·0% (0/2) |
| Hospital record found among those who attended a higher level of care |  |  | 0·0% (0/1) |  |  |  |
