# Supplementary material for: Effectiveness of introducing pulse oximetry and clinical decision support algorithms for the management of sick children in primary care in Kenya and Senegal on referral and antibiotic prescription: the TIMCI quasi-experimental pre-post study
Source: eClinicalMedicine. 2025 May 12;83:103196. doi: 10.1016/j.eclinm.2025.103196 (PMC12140026; doi:10.1016/j.eclinm.2025.103196)
Supplement: Supplement S5 [file mmc5.docx]

## Supplementary file S5 - Summary of secondary outcomes results

Similarly to primary outcomes, the secondary outcomes are assessed using generalised estimating equation for logistic regression, with facilities as clusters. Estimates for the pre-post effect on the outcomes are shown in terms of odds ratios and risk difference (with the pre-intervention period being the reference category) and their associated 95% confidence intervals. Results from both univariate and multivariate models are presented when numbers allowed. Not estimable intervention effects are denoted in the summary table below as “NE”.

Multivariate models are adjusted for age, sex, travel time to facility, illness duration, previous care and treatment, cough, fever, diarrhoea and danger signs.

### Summary of secondary outcomes results - infants under 2 months of age

| Outcome | Analysis | N (%) Pre-intervention | N (%) Post-intervention | Unadjusted | p-value | Adjusted | p-value |
| --- | --- | --- | --- | --- | --- | --- | --- |
| Severe complications by Day7 | Combined | 4 (0.4%) | 9 (0.5%) | 1.262 (0.381, 4.181) 0.1% (-0.4%, 0.6%) | 0.704 | NE | - |
|  | Kenya | 2 (0.6%) | 3 (0.4%) | 0.628 (0.160, 2.466) -0.3% (-1.1%, 0.6%) | 0.505 | NE | - |
|  | Senegal | 2 (0.3%) | 6 (0.6%) | 2.024 (0.409, 10.009) 0.3% (-0.4%, 1.0%) | 0.387 | NE | - |
| Appropriate referrals | Combined | 2 (0.2%) | 3 (0.2%) | 0.842 (0.136, 5.204) -0.0% (-0.4%, 0.3%) | 0.853 | NE | - |
|  | Kenya | 1 (0.3%) | 1 (0.1%) | 0.425 (0.027, 6.820) -0.2% (-0.8%, 0.5%) | 0.546 | NE | - |
|  | Senegal | 1 (0.2%) | 2 (0.2%) | 1.400 (0.122, 16.073) 0.1% (-0.4%, 0.5%) | 0.787 | NE | - |
| Completed referrals by Day7 | Combined | 3 (0.3%) | 3 (0.2%) | 0.553 (0.111, 2.765) -0.1% (-0.5%, 0.3%) | 0.470 | NE | - |
|  | Kenya | 1 (0.3%) | 1 (0.1%) | 0.425 (0.027, 6.820) -0.2% (-0.8%, 0.5%) | 0.546 | NE | - |
|  | Senegal | 2 (0.3%) | 2 (0.2%) | 0.676 (0.095, 4.793) -0.1% (-0.6%, 0.4%) | 0.695 | NE | - |
| Referrals with non-severe disease | Combined | 3 (0.3%) | 7 (0.4%) | 1.326 (0.359, 4.896) 0.1% (-0.4%, 0.6%) | 0.672 | NE | - |
|  | Kenya | 0 (0.0%) | 3 (0.4%) | NE | - | NE | - |
|  | Senegal | 3 (0.5%) | 4 (0.4%) | 0.934 (0.203, 4.286) -0.0% (-0.7%, 0.7%) | 0.930 | NE | - |
| Cure rate by Day7 | Combined | 897 (91.6%) | 1570 (89.8%) | 0.878 (0.562, 1.370) -1.1% (-4.7%, 2.6%) | 0.566 | 0.841 (0.509, 1.390) -2.4% (-9.5%, 4.7%) | 0.499 |
|  | Kenya | 286 (83.1%) | 703 (85.7%) | 1.132 (0.638, 2.011) 1.5% (-5.6%, 8.7%) | 0.671 | NE | - |
|  | Senegal | 611 (96.2%) | 867 (93.4%) | 0.585 (0.299, 1.143) -2.6% (-6.1%, 1.0%) | 0.117 | NE | - |

### Summary of secondary outcomes results - children 2-59 months of age

| Outcome | Analysis | N (%) Pre-intervention | N (%) Post-intervention | Unadjusted | p-value | Adjusted | p-value | |
| --- | --- | --- | --- | --- | --- | --- | --- | --- |
| Severe complications by Day7 | Combined | 24 (0.1%) | 56 (0.2%) | 1.204 (0.703, 2.064) 0.0% (-0.1%, 0.1%) | 0.499 | NE |  |  |
|  | Kenya | 12 (0.1%) | 30 (0.1%) | 1.065 (0.485, 2.336) 0.0% (-0.1%, 0.1%) | 0.876 | NE |  |  |
|  | Senegal | 12 (0.2%) | 26 (0.2%) | 1.430 (0.709, 2.885) 0.1% (-0.1%, 0.2%) | 0.317 | NE |  |  |
| Appropriate referrals | Combined | 5 (0.0%) | 7 (0.0%) | 0.786 (0.231, 2.672) -0.0% (-0.0%, 0.0%) | 0.700 | NE | - |  |
|  | Kenya | 1 (0.0%) | 5 (0.0%) | 2.499 (0.429, 14.561) 0.0% (-0.0%, 0.0%) | 0.308 | NE | - |  |
|  | Senegal | 4 (0.1%) | 2 (0.0%) | 0.346 (0.054, 2.210) -0.0% (-0.1%, 0.0%) | 0.262 | NE | - |  |
| Completed referrals by Day7 | Combined | 10 (0.1%) | 10 (0.0%) | 0.521 (0.235, 1.154) -0.0% (-0.1%, 0.0%) | 0.108 | NE | - |  |
|  | Kenya | 3 (0.0%) | 5 (0.0%) | 0.487 (0.118, 2.014) -0.0% (-0.0%, 0.0%) | 0.321 | NE | - |  |
|  | Senegal | 7 (0.1%) | 5 (0.0%) | 0.577 (0.225, 1.480) -0.0% (-0.1%, 0.0%) | 0.252 | NE | - |  |
| Referrals with non-severe disease | Combined | 41 (0.2%) | 40 (0.1%) | 0.527 (0.337, 0.824) -0.1% (-0.2%, -0.0%) | 0.005 | NE | - |  |
|  | Kenya | 22 (0.2%) | 29 (0.1%) | 0.603 (0.353, 1.029) -0.1% (-0.2%, 0.0%) | 0.063 | NE | - |  |
|  | Senegal | 19 (0.2%) | 11 (0.1%) | 0.406 (0.179, 0.919) -0.1% (-0.3%, -0.0%) | 0.031 | NE | - |  |
| Cure rate by Day7 | Combined | 14928 (89.0%) | 27914 (89.8%) | 1.291 (1.057, 1.576) 2.1% (0.4%, 3.8%) | 0.012 | 1.322 (1.076, 1.625) 2.2% (0.3%, 4.0%) | 0.008 |  |
|  | Kenya | 7651 (83.8%) | 17331 (86.0%) | 1.213 (0.987, 1.492) 2.4% (-0.3%, 5.1%) | 0.067 | 1.231 (1.003, 1.512) 2.0% (-0.2%, 4.2%) | 0.047 |  |
|  | Senegal | 7277 (95.0%) | 10583 (96.9%) | 1.721 (1.193, 2.482) 2.0% (0.6%, 3.3%) | 0.004 | 1.821 (1.325, 2.502) 5.4% (2.6%, 8.1%) | <0.001 |  |
| Febrile children tested for malaria | Combined | 4013 (37.8%) | 6787 (38.4%) | 0.989 (0.769, 1.272) -0.2% (-5.9%, 5.4%) | 0.934 | 1.028 (0.790, 1.338) 0.5% (-4.2%, 5.2%) | 0.837 |  |
|  | Kenya | 1757 (34.5%) | 4541 (41.8%) | 1.301 (0.945, 1.792) 5.6% (-1.3%, 12.5%) | 0.107 | 1.370 (0.993, 1.891) 7.1% (-0.1%, 14.2%) | 0.055 |  |
|  | Senegal | 2256 (40.8%) | 2246 (32.9%) | 0.720 (0.500, 1.038) -7.6% (-15.9%, 0.7%) | 0.079 | 0.755 (0.514, 1.108) -5.4% (-12.6%, 1.8%) | 0.151 |  |
| Antimalarial prescription for children tested positive | Combined | 208 (86.3%) | 626 (95.7%) | 1.451 (1.266, 1.664) 8.9% (5.5%, 12.2%) | <0.001 | NE | - |  |
|  | Kenya | 207 (89.2%) | 623 (97.0%) | 3.958 (2.330, 6.724) 7.8% (4.2%, 11.4%) | <0.001 | NE | - |  |
|  | Senegal | 1 (11.1%) | 3 (25.0%) | NE | - | NE | - |  |
| Antimalarial prescription for children tested negative | Combined | 12 (0.3%) | 37 (0.5%) | 1.710 (0.873, 3.346) 0.1% (-0.1%, 0.3%) | 0.118 | NE | - |  |
|  | Kenya | 10 (0.5%) | 37 (0.8%) | 1.583 (0.937, 2.675) 0.3% (-0.0%, 0.6%) | 0.086 | NE | - |  |
|  | Senegal | 2 (0.1%) | 0 (0.0%) | NE | - | NE | - |  |
| Antimalarial prescription for children untested | Combined | 28 (0.2%) | 19 (0.1%) | 0.361 (0.163, 0.801) -0.1% (-0.3%, 0.0%) | 0.012 | NE | - |  |
|  | Kenya | 18 (0.3%) | 15 (0.1%) | 0.405 (0.179, 0.915) -0.2% (-0.3%, 0.0%) | 0.030 | NE | - |  |
|  | Senegal | 10 (0.2%) | 4 (0.0%) | NE | - | NE | - |  |
