# Supplementary material for: Effectiveness of introducing pulse oximetry and clinical decision support algorithms for the management of sick children in primary care in Kenya and Senegal on referral and antibiotic prescription: the TIMCI quasi-experimental pre-post study
Source: eClinicalMedicine. 2025 May 12;83:103196. doi: 10.1016/j.eclinm.2025.103196 (PMC12140026; doi:10.1016/j.eclinm.2025.103196)
Supplement: Supplement S6 [file mmc6.docx]

## Supplementary file S6 – Summary of the safety evaluation

### Proportion of children who experienced a severe complication at any point during the study duration

The table below summarises severe complications (death or secondary hospitalisation) that occurred throughout the duration of the study.

### Deaths and secondary hospitalisations summary - infants under 2 months of age

|  | Pre-intervention | Post-intervention |
| --- | --- | --- |
| Deaths |  |  |
| Combined | 0.1% (1/979) | 0.2% (4/1748) |
| Kenya | 0.3% (1/344) | 0.1% (1/820) |
| Senegal | 0.0% (0/635) | 0.3% (3/928) |
| Hospitalisations without referral |  |  |
| Combined | 0.5% (5/979) | 0.3% (5/1748) |
| Kenya | 0.3% (1/344) | 0.2% (2/820) |
| Senegal | 0.6% (4/635) | 0.3% (3/928) |
| Hospitalisations with referral but delayed |  |  |
| Combined | 0.0% (0/979) | 0.0% (0/1748) |
| Kenya | 0.0% (0/344) | 0.0% (0/820) |
| Senegal | 0.0% (0/635) | 0.0% (0/928) |
| Severe complications |  |  |
| Combined | 0.6% (6/979) | 0.5% (9/1748) |
| Kenya | 0.6% (2/344) | 0.4% (3/820) |
| Senegal | 0.6% (4/635) | 0.6% (6/928) |

### Deaths and secondary hospitalisations summary - children 2-59 months of age

|  | Pre-intervention | Post-intervention |
| --- | --- | --- |
| Deaths |  |  |
| Combined | 0.0% (4/16782) | 0.0% (10/31071) |
| Kenya | 0.0% (1/9125) | 0.0% (3/20155) |
| Senegal | 0.0% (3/7657) | 0.1% (7/10916) |
| Hospitalisations without referral |  |  |
| Combined | 0.1% (22/16782) | 0.2% (51/31071) |
| Kenya | 0.1% (10/9125) | 0.1% (28/20155) |
| Senegal | 0.2% (12/7657) | 0.2% (23/10916) |
| Hospitalisations with referral but delayed |  |  |
| Combined | 0.0% (1/16782) | 0.0% (2/31071) |
| Kenya | 0.0% (1/9125) | 0.0% (2/20155) |
| Senegal | 0.0% (0/7657) | 0.0% (0/10916) |
| Severe complications |  |  |
| Combined | 0.2% (26/16782) | 0.2% (60/31071) |
| Kenya | 0.1% (12/9125) | 0.2% (32/20155) |
| Senegal | 0.2% (14/7657) | 0.3% (28/10916) |

### Missing data description

Missing values are due predominantly to children lost to follow-up and not reached at Day7.

Among 17744 children recruited in the pre-intervention period and expected to be followed-up at Day7 (i.e. not withdrawn before Day7), 5973 34% were lost to follow-up. Similarly, among 32816 children recruited in the post-intervention period and expected to be followed-up at Day7 (i.e. not withdrawn before Day7), 9074 28% were lost to follow-up.

The tables below describes relevant indicators recorded at Day0 by follow-up status.

|  | Lost to follow-up | | Successful follow-up | |
| --- | --- | --- | --- | --- |
|  | Pre-intervention | Post-intervention | Pre-intervention | Post-intervention |
| Urgent referral |  |  |  |  |
| Combined | 0.3% (16/5990) | 0.4% (38/9077) | 0.4% (51/11771) | 0.4% (88/23742) |
| Kenya | 0.3% (8/3164) | 0.5% (23/4547) | 0.4% (25/6305) | 0.4% (71/16428) |
| Senegal | 0.3% (8/2826) | 0.3% (15/4530) | 0.5% (26/5466) | 0.2% (17/7314) |
| Severe diagnosis without hypoxaemia |  |  |  |  |
| Combined | 3.8% (225/5990) | 4.1% (368/9077) | 3.7% (437/11771) | 3.1% (727/23742) |
| Kenya | 1.1% (35/3164) | 1.8% (83/4547) | 0.9% (57/6305) | 1.7% (286/16428) |
| Senegal | 6.7% (190/2826) | 6.3% (285/4530) | 7.0% (380/5466) | 6.0% (441/7314) |
| Severe diagnosis with hypoxaemia |  |  |  |  |
| Combined | 0.0% (1/5990) | 0.4% (32/9077) | 0.0% (2/11771) | 0.5% (117/23742) |
| Kenya | 0.0% (0/3164) | 0.3% (14/4547) | 0.0% (0/6305) | 0.5% (83/16428) |
| Senegal | 0.0% (1/2826) | 0.4% (18/4530) | 0.0% (2/5466) | 0.5% (34/7314) |
