# Supplementary material for: Effectiveness of introducing pulse oximetry and clinical decision support algorithms for the management of sick children in primary care in Kenya and Senegal on referral and antibiotic prescription: the TIMCI quasi-experimental pre-post study
Source: eClinicalMedicine. 2025 May 12;83:103196. doi: 10.1016/j.eclinm.2025.103196 (PMC12140026; doi:10.1016/j.eclinm.2025.103196)
Supplement: Supplement S7 [file mmc7.docx]

Supplementary file S7 – Summary of primary outcome results as per-protocol sensitivity analysis

The following definition was used for the per-protocol population:

- pre-intervention period: all enrolled children
- post-intervention period:
  - Kenya:
    - all enrolled children who received pulse oximetry and CDSA.
  - Senegal:
    - all enrolled children eligible to pulse oximetry who received pulse oximetry and CDSA, and
    - all enrolled children non-eligible to pulse oximetry who received CDSA.

### Children 2-59 months

| Table 1: Per-Protocol analysis summary (children 2-59 months)   \| **Group** \| **Outcome** \| **Pre**^1^ \| **Post**^1^ \| **Unadjusted** \| **p-value** \| **Adjusted** \| **p-value** \| \| --- \| --- \| --- \| --- \| --- \| --- \| --- \| --- \| \| Combined \| Antibiotic prescription \| 12,568 / 16,782 (74.9%) \| 6,340 / 13,055 (48.6%) \| 0.287 (0.221, 0.373) -0.293 (-0.352, -0.234) \| <0.001 \| 0.317 (0.241, 0.417) -0.247 (-0.294, -0.199) \| <0.001 \| \|  \| Urgent referrals \| 61 / 16,782 (0.4%) \| 66 / 13,055 (0.5%) \| 1.342 (0.987, 1.824) 0.001 (-0.000, 0.003) \| 0.061 \| 1.614 (1.125, 2.315) 0.007 (-0.004, 0.019) \| 0.009 \| \| Kenya \| Antibiotic prescription \| 7,690 / 9,125 (84.3%) \| 5,052 / 10,211 (49.5%) \| 0.196 (0.140, 0.274) -0.339 (-0.412, -0.267) \| <0.001 \| 0.233 (0.169, 0.322) -0.331 (-0.399, -0.263) \| <0.001 \| \|  \| Urgent referrals \| 31 / 9,125 (0.3%) \| 57 / 10,211 (0.6%) \| 1.528 (1.089, 2.144) 0.002 (0.000, 0.004) \| 0.014 \| N.E \| . \| \| Senegal \| Antibiotic prescription \| 4,878 / 7,657 (63.7%) \| 1,288 / 2,844 (45.3%) \| 0.503 (0.377, 0.671) -0.169 (-0.238, -0.099) \| <0.001 \| 0.551 (0.407, 0.746) -0.133 (-0.196, -0.071) \| <0.001 \| \|  \| Urgent referrals \| 30 / 7,657 (0.4%) \| 9 / 2,844 (0.3%) \| 0.826 (0.351, 1.941) -0.001 (-0.004, 0.002) \| 0.661 \| N.E \| . \| \| ^1^n / N (%) \| \| \| \| \| \| \| \| |
| --- | --- | --- | --- | --- | --- | --- | --- | --- | --- | --- | --- | --- | --- | --- | --- | --- | --- | --- | --- | --- | --- | --- | --- | --- | --- | --- | --- | --- | --- | --- | --- | --- | --- | --- | --- | --- | --- | --- | --- | --- | --- | --- | --- | --- | --- | --- | --- | --- | --- | --- | --- | --- | --- | --- | --- | --- | --- | --- | --- | --- | --- | --- | --- | --- |

### Children 1-59 days

| Table 2: Per-Protocol analysis summary (children 1-59 days)   \| **Group** \| **Outcome** \| **Pre**^1^ \| **Post**^1^ \| **Unadjusted** \| **p-value** \| **Adjusted** \| **p-value** \| \| --- \| --- \| --- \| --- \| --- \| --- \| --- \| --- \| \| Combined \| Antibiotic prescription \| 528 / 979 (53.9%) \| 219 / 597 (36.7%) \| 0.413 (0.312, 0.549) -0.214 (-0.280, -0.148) \| <0.001 \| 0.392 (0.300, 0.513) -0.177 (-0.228, -0.126) \| <0.001 \| \|  \| Urgent referrals \| 6 / 979 (0.6%) \| 7 / 597 (1.2%) \| 1.923 (0.623, 5.935) 0.006 (-0.004, 0.016) \| 0.255 \| N.E \| . \| \| Kenya \| Antibiotic prescription \| 229 / 344 (66.6%) \| 151 / 388 (38.9%) \| 0.312 (0.225, 0.432) -0.283 (-0.358, -0.208) \| <0.001 \| N.E \| . \| \|  \| Urgent referrals \| 2 / 344 (0.6%) \| 7 / 388 (1.8%) \| 3.763 (0.536, 26.429) 0.012 (-0.003, 0.028) \| 0.183 \| N.E \| . \| \| Senegal \| Antibiotic prescription \| 299 / 635 (47.1%) \| 68 / 209 (32.5%) \| 0.522 (0.321, 0.851) -0.153 (-0.262, -0.045) \| 0.009 \| N.E \| . \| \|  \| Urgent referrals \| 4 / 635 (0.6%) \| 0 / 209 (0.0%) \| N.E \| . \| N.E \| . \| \| ^1^n / N (%) \| \| \| \| \| \| \| \| |
| --- | --- | --- | --- | --- | --- | --- | --- | --- | --- | --- | --- | --- | --- | --- | --- | --- | --- | --- | --- | --- | --- | --- | --- | --- | --- | --- | --- | --- | --- | --- | --- | --- | --- | --- | --- | --- | --- | --- | --- | --- | --- | --- | --- | --- | --- | --- | --- | --- | --- | --- | --- | --- | --- | --- | --- | --- | --- | --- | --- | --- | --- | --- | --- | --- |
