# Supplementary material for: Effectiveness of introducing pulse oximetry and clinical decision support algorithms for the management of sick children in primary care in Kenya and Senegal on referral and antibiotic prescription: the TIMCI quasi-experimental pre-post study
Source: eClinicalMedicine. 2025 May 12;83:103196. doi: 10.1016/j.eclinm.2025.103196 (PMC12140026; doi:10.1016/j.eclinm.2025.103196)
Supplement: Supplement S8 [file mmc8.docx]

Supplementary file S8 – Summary of primary outcome results applying the Bonferroni correction for multiplicity

The Bonferroni correction adjusts for multiple comparisons by testing each individual primary outcome (urgent referral and antibiotic prescription) at a significance level of $\alpha$ = 0.05 / 2 = 0.025.

### Children 2-59 months

| Table 1: Analysis summary when applying the Bonferroni correction for multiplicity (children 2-59 months)   \| **Group** \| **Outcome** \| **Pre**^1^ \| **Post**^1^ \| **Unadjusted**^2^ \| **p-value** \| **Adjusted**^2^ \| **p-value** \| \| --- \| --- \| --- \| --- \| --- \| --- \| --- \| --- \| \| Combined \| Antibiotic prescription \| 12,568 / 16,782 (74.9%) \| 15,845 / 31,071 (51.0%) \| 0.327 (0.250, 0.427) -0.261 (-0.321, -0.200) \| <0.001 \| 0.357 (0.276, 0.462) -0.219 (-0.267, -0.170) \| <0.001 \| \|  \| Urgent referrals \| 61 / 16,782 (0.4%) \| 112 / 31,071 (0.4%) \| 0.987 (0.704, 1.382) -0.000 (-0.001, 0.001) \| 1.858 \| 1.169 (0.809, 1.689) 0.001 (-0.003, 0.006) \| 0.685 \| \| Kenya \| Antibiotic prescription \| 7,690 / 9,125 (84.3%) \| 11,206 / 20,155 (55.6%) \| 0.235 (0.161, 0.344) -0.295 (-0.377, -0.213) \| <0.001 \| 0.299 (0.209, 0.428) -0.281 (-0.358, -0.203) \| <0.001 \| \|  \| Urgent referrals \| 31 / 9,125 (0.3%) \| 86 / 20,155 (0.4%) \| 1.262 (0.885, 1.801) 0.001 (-0.000, 0.002) \| 0.284 \| N.E \| . \| \| Senegal \| Antibiotic prescription \| 4,878 / 7,657 (63.7%) \| 4,639 / 10,916 (42.5%) \| 0.431 (0.293, 0.633) -0.207 (-0.300, -0.114) \| <0.001 \| 0.492 (0.339, 0.715) -0.134 (-0.218, -0.051) \| <0.001 \| \|  \| Urgent referrals \| 30 / 7,657 (0.4%) \| 26 / 10,916 (0.2%) \| 0.605 (0.287, 1.278) -0.002 (-0.004, 0.000) \| 0.264 \| N.E \| . \| \| ^1^n / N (%)  ^2^95% CI are Bonferroni adjusted \| \| \| \| \| \| \| \| |
| --- | --- | --- | --- | --- | --- | --- | --- | --- | --- | --- | --- | --- | --- | --- | --- | --- | --- | --- | --- | --- | --- | --- | --- | --- | --- | --- | --- | --- | --- | --- | --- | --- | --- | --- | --- | --- | --- | --- | --- | --- | --- | --- | --- | --- | --- | --- | --- | --- | --- | --- | --- | --- | --- | --- | --- | --- | --- | --- | --- | --- | --- | --- | --- | --- |

### Children 1-59 days

| Table 2: Analysis summary when applying the Bonferroni correction for multiplicity (children 1-59 days)   \| **Group** \| **Outcome** \| **Pre**^1^ \| **Post**^1^ \| **Unadjusted**^2^ \| **p-value** \| **Adjusted**^2^ \| **p-value** \| \| --- \| --- \| --- \| --- \| --- \| --- \| --- \| --- \| \| Combined \| Antibiotic prescription \| 528 / 979 (53.9%) \| 641 / 1,748 (36.7%) \| 0.434 (0.321, 0.586) -0.202 (-0.273, -0.132) \| <0.001 \| 0.453 (0.348, 0.590) -0.141 (-0.207, -0.075) \| <0.001 \| \|  \| Urgent referrals \| 6 / 979 (0.6%) \| 14 / 1,748 (0.8%) \| 1.309 (0.394, 4.347) 0.002 (-0.006, 0.010) \| 1.229 \| N.E \| . \| \| Kenya \| Antibiotic prescription \| 229 / 344 (66.6%) \| 348 / 820 (42.4%) \| 0.354 (0.265, 0.474) -0.253 (-0.320, -0.186) \| <0.001 \| N.E \| . \| \|  \| Urgent referrals \| 2 / 344 (0.6%) \| 8 / 820 (1.0%) \| 1.686 (0.252, 11.300) 0.004 (-0.009, 0.017) \| 1.077 \| N.E \| . \| \| Senegal \| Antibiotic prescription \| 299 / 635 (47.1%) \| 293 / 928 (31.6%) \| 0.481 (0.292, 0.791) -0.168 (-0.274, -0.062) \| 0.002 \| N.E \| . \| \|  \| Urgent referrals \| 4 / 635 (0.6%) \| 6 / 928 (0.6%) \| 1.023 (0.209, 5.011) 0.000 (-0.010, 0.010) \| 1.949 \| N.E \| . \| \| ^1^n / N (%)  ^2^95% CI are Bonferroni adjusted \| \| \| \| \| \| \| \| |
| --- | --- | --- | --- | --- | --- | --- | --- | --- | --- | --- | --- | --- | --- | --- | --- | --- | --- | --- | --- | --- | --- | --- | --- | --- | --- | --- | --- | --- | --- | --- | --- | --- | --- | --- | --- | --- | --- | --- | --- | --- | --- | --- | --- | --- | --- | --- | --- | --- | --- | --- | --- | --- | --- | --- | --- | --- | --- | --- | --- | --- | --- | --- | --- | --- |
