# Supplementary material for: Effectiveness of introducing pulse oximetry and clinical decision support algorithms for the management of sick children in primary care in Kenya and Senegal on referral and antibiotic prescription: the TIMCI quasi-experimental pre-post study
Source: eClinicalMedicine. 2025 May 12;83:103196. doi: 10.1016/j.eclinm.2025.103196 (PMC12140026; doi:10.1016/j.eclinm.2025.103196)
Supplement: 2024_TIMCI study design [file mmc9.pdf]

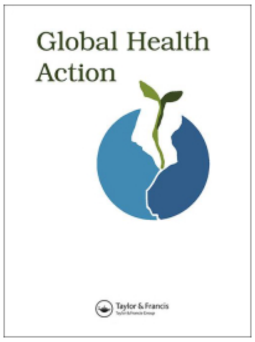


Global Health Action

ISSN: (Print) (Online) Journal homepage: www.tandfonline.com/journals/zgha20

# The Tools for Integrated Management of Childhood Illness (TIMCI) study protocol: a multi-country mixed-method evaluation of pulse oximetry and clinical decision support algorithms

**Fenella Beynon, Hélène Langet, Leah F. Bohle, Shally Awasthi, Ousmane Ndiaye, James Machoki M'Imunya, Honorati Masanja, Susan Horton, Maymouna Ba, Silvia Cicconi, Mira Emmanuel-Fabula, Papa Moctar Faye, Tracy R. Glass, Kristina Keitel, Divas Kumar, Gaurav Kumar, Gillian A. Levine, Lena Matata, Grace Mhalu, Andolo Miheso, Deusdedit Mjungu, Francis Njiri, Elisabeth Reus, Michael Ruffo, Fabian Schär, Kovid Sharma, Helen L. Storey, Irene Masanja, Kaspar Wyss, Valérie D'Acremont & TIMCI Collaborator Group**

**To cite this article:** Fenella Beynon, Hélène Langet, Leah F. Bohle, Shally Awasthi, Ousmane Ndiaye, James Machoki M'Imunya, Honorati Masanja, Susan Horton, Maymouna Ba, Silvia Cicconi, Mira Emmanuel-Fabula, Papa Moctar Faye, Tracy R. Glass, Kristina Keitel, Divas Kumar, Gaurav Kumar, Gillian A. Levine, Lena Matata, Grace Mhalu, Andolo Miheso, Deusdedit Mjungu, Francis Njiri, Elisabeth Reus, Michael Ruffo, Fabian Schär, Kovid Sharma, Helen L. Storey, Irene Masanja, Kaspar Wyss, Valérie D'Acremont & TIMCI Collaborator Group (2024) The Tools for Integrated Management of Childhood Illness (TIMCI) study protocol: a multi-country mixed-method evaluation of pulse oximetry and clinical decision support algorithms, Global Health Action, 17:1, 2326253, DOI: 10.1080/16549716.2024.2326253

**To link to this article:** https://doi.org/10.1080/16549716.2024.2326253

© 2024 The Author(s). Published by Informa UK Limited, trading as Taylor & Francis Group.

View supplementary material

Published online: 29 Apr 2024.

Submit your article to this journal

Article views: 758

View related articles

View Crossmark data

Full Terms & Conditions of access and use can be found at
https://www.tandfonline.com/action/journalInformation?journalCode=zgha20

GLOBAL HEALTH ACTION
2024, VOL. 17, 2326253
https://doi.org/10.1080/16549716.2024.2326253

Taylor & Francis
Taylor & Francis Group

STUDY DESIGN ARTICLE

OPEN ACCESS Check for updates

# The Tools for Integrated Management of Childhood Illness (TIMCI) study protocol: a multi-country mixed-method evaluation of pulse oximetry and clinical decision support algorithms

Fenella Beynon [a,b,*], Hélène Langet [a,b,*], Leah F. Bohle [a,b,*], Shally Awasthi [c,**], Ousmane Ndiaye [d,**], James Machoki M'Imunya [e,**], Honorati Masanja [f,**], Susan Horton [g,**], Maymouna Ba[h], Silvia Cicconi [b,i], Mira Emmanuel-Fabula [h], Papa Moctar Faye [d], Tracy R. Glass [b,i], Kristina Keitel [i,j], Divas Kumar [c], Gaurav Kumar[a,b], Gillian A. Levine [b,i], Lena Matata [a,b,k], Grace Mhalu[k], Andolo Miheso[h], Deusdedit Mjungu [h], Francis Njiri [e], Elisabeth Reus[b,i], Michael Ruffo[h], Fabian Schär [a,b], Kovid Sharma[h], Helen L. Storey [h], Irene Masanja[k,#], Kaspar Wyss [a,b,#], Valérie D'Acremont [i,l,#] and TIMCI Collaborator Group

[a]Swiss Centre for International Health, Swiss Tropical and Public Health Institute, Allschwil, Switzerland; [b]Faculty of Science, University of Basel, Basel, Switzerland; [c]Department of Paediatrics, King George's Medical University, Lucknow, India; [d]Faculté de médecine, Université Cheikh Anta Diop, Dakar, Senegal; [e]College of Health Sciences, University of Nairobi, Nairobi, Kenya; [f]Directorate, Ifakara Health Institute, Dar es Salaam, Tanzania; [g]School of Public Health and Health Systems, University of Waterloo, Waterloo, Canada; [h]PATH; [i]Department of Medicine, Swiss Tropical and Public Health Institute, Allschwil, Switzerland; [j]Division of Pediatric Emergency Medicine, Department of Pediatrics,Inselspital, University of Bern, Bern, Switzerland; [k]Health Systems, Impact Evaluation and Policy, Ifakara Health Institute, Dar es Salaam, Tanzania; [l]Digital Global Health Department, Centre for Primary Care and PublicHealth (Unisanté), University of Lausanne, Lausanne, Switzerland

**ABSTRACT**
Effective and sustainable strategies are needed to address the burden of preventable deaths among children under-five in resource-constrained settings. The Tools for Integrated Management of Childhood Illness (TIMCI) project aims to support healthcare providers to identify and manage severe illness, whilst promoting resource stewardship, by introducing pulse oximetry and clinical decision support algorithms (CDSAs) to primary care facilities in India, Kenya, Senegal and Tanzania. Health impact is assessed through: a pragmatic parallel group, superiority cluster randomised controlled trial (RCT), with primary care facilities randomly allocated (1:1) in India to pulse oximetry or control, and (1:1:1) in Tanzania to pulse oximetry plus CDSA, pulse oximetry, or control; and through a quasi-experimental pre-post study in Kenya and Senegal. Devices are implemented with guidance and training, mentorship, and community engagement. Sociodemographic and clinical data are collected from caregivers and records of enrolled sick children aged 0–59 months at study facilities, with phone follow-up on Day 7 (and Day 28 in the RCT). The primary outcomes assessed for the RCT are severe complications (mortality and secondary hospitalisations) by Day 7 and primary hospitalisations (within 24 hours and with referral); and, for the pre-post study, referrals and antibiotic. Secondary outcomes on other aspects of health status, hypoxaemia, referral, follow-up and antimicrobial prescription are also evaluated. In all countries, embedded mixed-method studies further evaluate the effects of the intervention on care and care processes, implementation, cost and cost-effectiveness. Pilot and baseline studies started mid-2021, RCT and post-intervention mid-2022, with anticipated completion mid-2023 and first results late-2023. Study approval has been granted by all relevant institutional review boards, national and WHO ethical review committees. Findings will be shared with communities, healthcare providers, Ministries of Health and other local, national and international stakeholders to facilitate evidence-based decision-making on scale-up.
**Study registration**: NCT04910750 and NCT05065320

**ARTICLE HISTORY**
Received 3 August 2023
Accepted 25 February 2024

**RESPONSIBLE EDITOR**
Julia Schröders

**KEYWORDS**
Hypoxaemia; IMCI; primary care; quality of care; cluster randomized controlled trial

**PAPER CONTEXT**

- Pulse oximetry and clinical decision support algorithms show potential for supporting healthcare providers to identify and manage severe illness among children under-five attending primary care in resource-constrained settings, whilst promoting resource stewardship but scale-up has been hampered by evidence gaps.

**CONTACT** Fenella Beynon fenella.beynon@swisstph.ch Swiss Centre for International Health, Swiss Tropical and Public Health Institute, Kreuzstrasse 2, Allschwil 4123, Switzerland

*FB, HL, LFB contributed equally to drafting the global protocol, with input and review by KW, VD, KK, TG, ER, GL, HLS, SH. FB drafted the first version of the manuscript with input from HL, LFB, SC and TG. All authors contributed to the review and revision of the draft and approval of the final protocol manuscript.
**As respective country Principal Investigators (PIs), SA, OD, JM, HN contributed to the global protocol and led country-specific adaptions; SH contributed as PI for the economic evaluation, PMF, FN, GK, LM gave input to the global study protocol and country-specific adaptations. In addition to contributors already listed, GM and DK contributed to protocol amendments.
#KW and VDA contributed equally to the overall study design as global PIs; IM was critical to the project and study design and is acknowledged here in memoriam.

Supplemental data for this article can be accessed online at https://doi.org/10.1080/16549716.2024.2326253

© 2024 The Author(s). Published by Informa UK Limited, trading as Taylor & Francis Group.
This is an Open Access article distributed under the terms of the Creative Commons Attribution License (http://creativecommons.org/licenses/by/4.0/), which permits unrestricted use, distribution, and reproduction in any medium, provided the original work is properly cited. The terms on which this article has been published allow the posting of the Accepted Manuscript in a repository by the author(s) or with their consent.

- This study design article describes the largest scale evaluation of these interventions to date, the results of which will inform country- and global-level policy and planning.

## Introduction

### Background and rationale

The vast majority of the 5 million annual deaths of children under-five occur in low- and middle-income countries [1]. With most of these deaths resulting from preventable causes, strategies to improve the early identification and management of sick children, whilst promoting resource stewardship, are needed. The Integrated Management of Childhood Illness (IMCI) strategy, launched by WHO and UNICEF in 1995 and now adopted by over 100 countries, seeks to address this need by providing an evidence-based, simple, structured approach for the integrated assessment, classification and treatment of sick children in resource-constrained settings [2,3].

IMCI was designed to have high sensitivity for severe disease, but a number of studies have demonstrated poor identification of children with hypoxaemia [4–6] who are at a significantly increased risk of mortality [7]. Clinical signs do not reliably predict hypoxaemia and thus cannot effectively be used to identify children who need (or do not need) oxygen [8]. Affordable, robust, easy-to-use pulse oximeters, which provide an accurate, non-invasive method of detecting hypoxaemia, have become increasingly available in recent years, generating calls for their introduction and use in primary care to support the early detection of severe illness in children under five [9–11].

Pulse oximetry has been shown to help identify (and prompt referral for) severe pneumonia among sick children attending primary care which would otherwise have been missed based on IMCI clinical signs alone [5,6,12]. However, scale-up efforts have been hampered by limitations in evidence on health impact and cost-effectiveness, and knowledge gaps on feasible implementation approaches in different contexts; evidence is particularly sparse on the use of pulse oximetry for children with non-pneumonia syndromes, who represent an important proportion of children with hypoxaemia [4,5,13–15].

In addition to the problem of missing hypoxaemia when relying on clinical signs alone, numerous studies have shown poor identification and management of severely ill children among health workers as a result of non-adherence to guidelines [16–19]. Clinical decision support algorithms (CDSAs) – digital tools which can guide health workers through consultations by making recommendations on assessment and management based on individual patient characteristics – have been recommended by WHO to support the implementation of guidelines such as IMCI [20]. Several child health focused CDSAs have demonstrated relevant improvements in quality of care and antimicrobial stewardship, and some have demonstrated improvements in clinical and health outcomes [21–25]. However, evidence on health impact, cost-effectiveness and approaches to sustaining and scaling up these digital tools remains limited [26,27].

By introducing pulse oximetry and CDSAs, the Tools for Integrated Management of Childhood Illness (TIMCI) project aims to contribute to reducing morbidity and mortality for sick children attending primary care facilities in India, Kenya, Senegal and Tanzania, while supporting the rational and efficient use of diagnostics and medicines by healthcare providers. The multi-country, mixed-method evaluation component of the project will generate evidence on the health and quality of care impact, operational priorities, cost and cost-effectiveness of introducing these tools to facilitate national and international decision-making on scale-up.

### Study design

The TIMCI study is a mixed-method evaluation, with pragmatic cluster randomised controlled trials (RCTs) in India and Tanzania (NCT04910750), and quasi-experimental pre-post studies in Kenya and Senegal (NCT05065320), complemented by embedded mixed-method studies in all countries, outlined in Figure 1. The design is informed by quality of care, acceptability and behaviour change frameworks, the project's Theory of Change, and draws on principles of realist evaluation and process evaluation of complex interventions [28–34]. Prior to the RCT and alongside the pre-intervention period, a 3-month pilot was conducted to evaluate and refine the intervention package, and pilot research tools, processes and assumptions – the methods outlined here reflect the final protocols, following adaptations based on this pilot. The protocols, the full versions of which are available on the trial registries, were developed in accordance with SPIRIT guidelines [35].

In the pragmatic, parallel group, superiority cluster RCT, we compare care and outcomes of children attending primary care facilities (clusters) randomly allocated (1:1) in India to pulse oximetry or control, and (1:1:1) in Tanzania to pulse oximetry plus CDSA, pulse oximetry, or control. The CDSA arm was not included in India following pilot findings that indicated a need for substantial adaptation and further piloting before its effectiveness

| | | | |
|---|---|---|---|
| **Pragmatic cluster randomised controlled trial** | Pragmatic, parallel group, superiority cluster RCT<br>• Pulse oximetry + CDSA (Tanzania only)<br>• Pulse oximetry + paper job aid<br>• Routine care (refresher training) | Primary outcomes<br>• Severe complications (mortality & secondary hospitalisations – delayed ≥24hrs or unreferred)<br>• Primary hospitalisations (<24hrs and with referral) | Secondary outcomes (groups)<br>• Hypoxaemia, health status, referral, hospitalisation, follow-up, antimicrobial prescribing and use, device use |
| **Quasi-experimental pre-post study** | Quasi-experimental pre-post study in a sub-set of facilities engaged in the TIMCI project:<br>• Facilities pre-intervention (pulse oximetry + CDSA<br>• Same facilities post-intervention | Primary outcomes<br>• Referral to a higher level of care<br>• Antibiotic prescription | Secondary outcomes (groups)<br>• Hypoxaemia, health status, referral, hospitalisation, follow-up, antimicrobial prescribing and use, device use |
| **Service provision assessments** | Periodic SPAs (facility assessment, healthcare provider interviews, consultation observations, caregiver exit interviews) in a sub-set of RCT/pre-post facilities | • Uptake of interventions<br>• Adherence to pulse oximetry + CDSA use steps<br>• Adherence to key IMCI assessment + management indicators | • Caregiver understanding & satisfaction<br>• Facility and provider factors associated with uptake & impact |
| **Process mapping & timeflow** | Care pathway mapping, observation & timing (& changes with the intervention) through non-participant observation & 'shadowing' of individual sick children & caregivers in a sub-set of RCT/pre-post facilities | • Process maps of care pathways with and without pulse oximetry ± CDSA<br>• Understanding of implementation context, mechanisms, process | • Qualitative insights into quality of care, intervention acceptability and behaviour change related to intervention<br>• Visit, consultation and device use time |
| **Healthcare provider in-depth interviews** | Semi-structured IDIs, incorporating clinical vignettes, with a purposive sample of healthcare providers; focusing on experience with devices & intervention acceptability | • Experiences of the intervention<br>• Acceptability of pulse oximetry ± CDSA use<br>• Reasons for early / late adoption / change of uptake and acceptability over time | • Barriers & facilitators to intervention success<br>• Explanatory insights into quality of care indicators observed in quantitative studies |
| **Caregiver in-depth interviews** | Semi-structured IDIs with a purposive sample of caregivers; focusing on quality of care & acceptability frameworks, behaviour change & incorporating a realist approach | • Experiences of the intervention<br>• Acceptability of pulse oximetry ± CDSA use<br>• Change in acceptability over time<br>• Caregiver experience of care, including referral | • Insights into care-seeking behaviour prior to and after consultation, particularly to referral advice |
| **Stakeholder survey (+ KIIs in Kenya & Senegal)** | Periodic online surveys with key stakeholders at global, national & sub-national level to understand processes, mechanisms & context of implementation, including adaptations made | • Understanding of implementation context, mechanisms, process<br>• Understand stakeholder perceptions of the intervention at different levels of the health system | • Understanding potential facilitators and barriers to scale the interventions to national and international level |
| **Project data review** | Programmatic monitoring data, routine health management information system data, & de-identified CDSA consultation data assessed for all facilities | • Understanding the wider context & impact of the intervention in non-research & non-TIMCI facilities<br>• Understanding the strengths & weaknesses of different data sources for intervention monitoring | • Understanding the potential for CDSA data to be used with HMIS data |
| **Economic evaluation** | Cost and cost-effectiveness studies analysis using direct health system costs, assessing financial & economic costs of the intervention | • Full implementation cost (cost to health system, incl costs paid by households to health system)<br>• Cost per child screened using pulse oximetry / CDSA, compared to cost per child in routine care | • Cost per DALY averted attributed to intervention modelled on hospitalization & outcome data from effectiveness studies, supplemented by literature |

Figure 1. Overview of the multi-country, multi-method TIMCI evaluation – study design and main outcomes. Abbreviations: CDSA: clinical decision support algorithm; RCT: randomised controlled trial; KII: key informant interviews; HMIS: health management information system; DALY: disease-adjusted life years.

could be evaluated. In the quasi-experimental pre-post study in Kenya and Senegal, we compare care and outcomes of children attending primary care facilities before and after implementation of pulse oximetry and CDSA. We chose a cluster design at the facility level: to avoid contamination (that would occur if randomisation were at the individual child or health worker level) and introduce different processes within one facility and enable evaluation of effectiveness in real-world settings.

Embedded mixed-methods studies include a modified Service Provision Assessment (SPA) [36], facility-based process mapping and time-flow studies, in-depth interviews (IDIs) with caregivers and healthcare providers, online key stakeholder surveys (and key informant interviews (KIIs) in Kenya and Senegal only), routine data review, and an economic evaluation.

## Methods and analysis

### Study setting & eligibility criteria

The study is centred on facility-based primary care, from small facilities such as dispensaries and health posts up to outpatient settings at larger health centres, in diverse contexts in India, Kenya, Senegal and Tanzania (Table 1) [37–39].

Government primary care facilities are eligible if they provide curative primary care services for children aged 0–59 months, with access to oxygen (on site or at the designated government referral facility) and electricity (continuous or intermittent). Facilities are excluded if they are inaccessible for significant parts of the year, saw fewer than 20 sick children per month (12-month average prior to eligibility assessment), already systematically used pulse oximetry for sick child consultations, or had another major programmatic or research intervention planned during the study period which could significantly influence the primary outcome.

Children are eligible for the quantitative studies if they are aged 0–59 months, attend a study facility during the relevant study's recruitment period, and are reported by the caregiver to be ill (regardless of whether they are attending for an acute care or routine visit). Children are excluded if they are less than one day old, attending for trauma only, are already admitted in a ward within the facility, or were previously enrolled in the study within the preceding 28 days.

Caregivers are eligible for IDIs if their child is enrolled in an intervention arm (RCT) or post-intervention (pre-post study) facility. Healthcare providers are eligible for IDIs and SPA interviews if they provide care for children aged 0–59 months at a study facility (and for the SPA, if they are present on the day(s) of assessment). Medical and non-medical personnel from study facilities and government hospitals to which the primary care facilities refer are eligible to provide costing data. Stakeholders are eligible for KIIs and surveys if they are involved in policy, implementation or research in child health and/or interventions at international, national, or sub-national level.

### Interventions

The TIMCI intervention package includes devices (pulse oximetry, with or without tablet-based CDSA) and related guidance and training on IMCI, pulse oximetry, CDSA and routine data reporting; monitoring and evaluation with supportive supervision; and community engagement. The 'global' package was developed collaboratively by the consortium, based on institutional experience, formal and informal exchange, review of evidence and stakeholder consultation internationally and within each country. Country-specific adaptations, based on engagement with Ministries of Health (MoHs) and other stakeholders, are outlined in Table 1.

### Pulse oximetry

Handheld pulse oximeters (Acare AH-MX devices [40] procured through the UNICEF catalogue) were selected for their portability, reliability, affordability, and suitability for children and neonates. We expanded use criteria relative to IMCI [3], to explore the relevance and feasibility of pulse oximetry for non-pneumonia syndromes, which may account for significant hypoxaemia burden [4,13,14]. MoHs in each country determined the criteria for pulse oximetry use. In Senegal and Tanzania, this included all young infants under 2 months, all children with cough or difficulty breathing, and all children with IMCI moderate (yellow) and severe (red) classifications (Figure 2) [1,4,13,14,16,41]. In India and Kenya, MoHs opted to recommend pulse oximetry for all sick young infants and children.

Three probes (universal, paediatric and neonatal) are provided, with training on how to use each of them according to the age/size of the child. Providers are advised to use a universal probe fully over the toe of a young infant (or neonatal wrap on a digit if not able to obtain a good waveform with the universal probe) and a paediatric probe on a finger (or a universal probe over the big toe if agitated or unable to obtain a good waveform). In Kenya, providers are advised to use the universal probe in the first instance for all children, and in Tanzania providers are advised to use the age-appropriate probes in the first instance. Healthcare providers are advised to attempt to obtain a reading for no longer than 5 minutes, as most readings are obtainable within this time [12,42,43]: to urgently refer children with $SpO2 < 90\%$ ($< 92\%$ in Senegal) and to reinforce the importance of referral for children with severe illness and $SpO2 < 94\%$, who may require oxygen [44]. Guidance is incorporated into updated IMCI chart booklets and the CDSA and is accompanied by a paper job aid on how to use the oximeter.

**Table 1.** Overview of the study setting and intervention according by country.

| | India (Uttar Pradesh) | Kenya | Senegal | Tanzania |
|---|---|---|---|---|
| U5 mortality | 44–60/1000 live births [36] | 37/1000 live births [37] | 39/1000 live births [37] | 43/1000 live births [38] |
| Geographical areas & altitude [37] | Unnao: 101–139 m<br>Sitapur: 108-158 m<br>Deoria: 55–100 m | Kakamega: 1458–1592 m<br>Kitui: 621–1605 m<br>Uasin Gishu 1007–2886 m | Thiès: −3–137 m | Sengerema: 1229–1328 m<br>Tanga CC: −2–238 m<br>Kaliua: 1052–1655 m |
| Facilities included | Small facilities; 1–2 providers consulting sick children. Basic preventive & curative outpatient services | | | |
| | PHCs | Level 2 (dispensaries) | Health posts | Dispensaries |
| | Larger primary care facilities (or higher level facilities providing outpatient primary care services). Multiple providers consulting sick children; 24/7 emergency services, (limited) admission capacity | | | |
| | CHCs | Level 3 (HCs/SCHs) | N/A | HCs |
| Staff using intervention | Non-specialist (MBBS) doctors; + paediatricians in some CHCs | Clinical officers, nurses, (+doctors in L3) | Nurses and nurse-assistants | Nurses, clinical officers; + doctors in large HCs |
| Referral | Refer to DH or CHC with paediatrician. Access to free ambulances with O2, but subject to availability | L2 refer to L3 or directly to hospital depending on services. Ambulances for emergencies (hospital-based); most patients organize own transport | Most HPs refer to 'referral' HCs; some refer directly to hospital (based on services & proximity); ambulances available | Refer to HCs or DHs. Mostly private transport e.g. boda bodas (motorbikes), bajaji (tricycles) and taxis |
| Oxygen availability | O2 cylinders/concentrators at PHCs & CHCs; piped O2 at hospitals | Some HCs have O2 cylinders/ concentrators; hospitals have O2 (type varies) | Very few HPs have O2 cylinders; O2 at referral HCs and hospitals (type varies) | All HCs have O2 cylinders; hospitals have O2 (type varies) |
| Pulse oximetry use criteria | All sick infants & children | | • All young infants under 2 months of age<br>• All 2–59 months with cough/difficulty breathing<br>• Children 2–59 months with IMCI/CDSA moderate (yellow)/ severe (red) classifications | |
| When to refer (and give O2 if available) | SpO2 < 90%<br>Reinforced if SpO2 < 94% + severe illness | SpO2 < 90% | SpO2 < 92%<br>Reinforced if SpO2 92 to < 95% + severe illness | SpO2 < 90% |
| CDSA | (Pilot only) | IMNCI + additional diagnoses and granularity, including: skin, abdominal/gastrointestinal, urinary, ENT, eye, MSK/injuries/anomalies (variation according to Ministries of Health + national guidelines) | | |
| Training | In-person:<br>1-day pulse oximetry (intervention arm only)<br>Online:<br>1-day IMNCI refresher (intervention + control)<br>'On-the-job' training for additional staff at CHCs | In-person group training for 1 providers/facility:<br>1-week integrated IMNCI + pulse oximetry<br>2-day CDSA + record keeping<br>'On-the-job' training for additional staff (~3–4/facility) | In-person group training for 2 providers/facility: 1-week integrated IMNCI, pulse oximetry, CDSA training<br>'On-the-job' training for 8 facilities | 3-month blended distance/ in-person IMCI refresher (all arms)<br>In person:<br>1-day pulse oximetry (intervention arms)<br>1-day CDSA (CDSA arm only)<br>'On-the-job' training as needed for new staff |
| Mentorship & supportive supervision | Joint, by district officials & PATH every 2 months; debrief with Chief Medical Officer | Joint, by MOH & county DoH, SC child health focal person, PATH, quarterly; monthly by SC child health focal person; debrief with CHMT and facilities (online). Whatsapp support. | 1 supportive supervision visit to all facilities with debrief after training; subsequent quarterly visitsDHIS2 data monitoring & additional paper forms (on pulse oximetry, referral) | In-person & phone supportive supervision; monthly PATH/CHMT joint facility visits. Two one-day joint provider meetings per district |
| M&E | Weekly summary data on pulse oximetry use | Weekly summary data on pulse oximetry + CDSA use | | |
| Community engagement | Engagement with ASHAs to co-develop and deliver communication materials on danger signs and health seeking behaviour; district community engagement workshops | CSOs, using national materials adapted with CHMTs, engaged with CHVs. Health facility education, advocacy, media broadcasts, community dialogues. | MoH materials & messages adapted by CSOs, used through talks, home visits, advocacy, social mobilisation, facility education sessions | CSOs and CHWs engagement with village leaders, community members, community theatre, household visits |

Abbreviations: PHC: primary health centre; CHC: community health centre; HP: health posts (poste de santé); HCs: health centres; DH: district hospital; O2: oxygen; SpO2: oxygen saturation; IM(N)CI: integrated management of (neonatal and) childhood illness; ENT: ear, nose, throat; MSK: musculoskeletal; MoH: ministry of health; DoH: department of health; SC: sub-country; CHMT: community health management team; ASHA: government community health worker; CSO: civil society organisation; CHV: community health volunteer; CHW: community health worker.

Although some communities in Uasin Gishu in Kenya are above the WHO 2500m altitude threshold for lowering the SpO2 referral cut-off [44], all primary care facilities were situated below 2500m altitude and therefore no adjustment to SpO2 cut-off was made.

## Clinical decision support algorithm

The CDSA, comprising the clinical algorithm (ePOCT+) and software platform (medAL-*suite*), described in more detail elsewhere [45,46], uses decision logic to guide healthcare providers through consultations based on demographic and clinical information they enter about an individual child. The algorithms are drafted by country-specific clinical algorithm development groups in consultation with MoH, based on national IMCI (0–2 and 2–59 month modules) and other relevant child health guidelines. The MoH-approved algorithms are

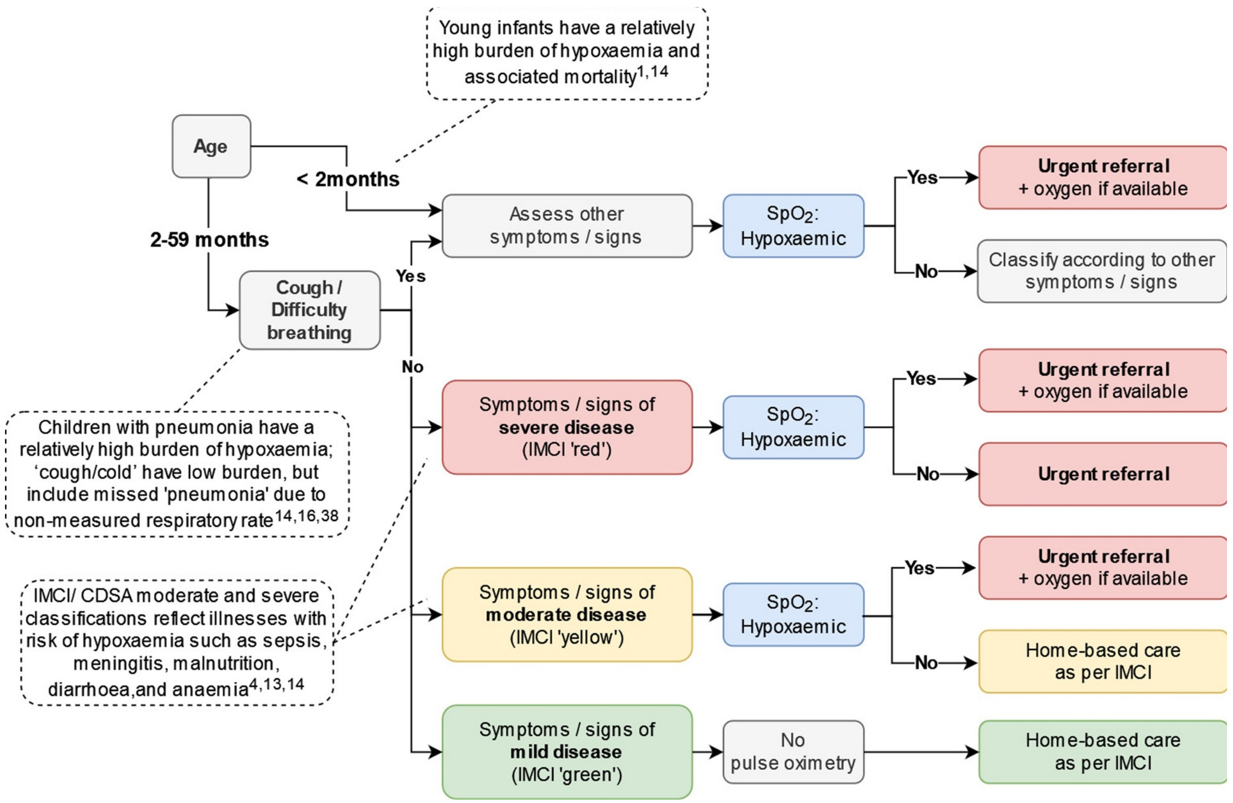


**Figure 2.** Pulse oximetry criteria for Senegal and Tanzania. In India and Kenya, Ministries opted to recommend pulse oximetry for all sick young infants and children.

programmed into the medAL-*creator* algorithm builder and transformed into the end-user tablet-based application, medAL-*reader*. This undergoes extensive desk-based testing using clinical vignettes before testing and piloting with healthcare providers. Feedback is reviewed with MoHs to inform the final algorithms and clinical content, which are programmed and tested before roll-out. Troubleshooting and feedback mechanisms support ongoing implementation.

### Training

Global pulse oximetry and CDSA training materials are adapted through consultation with technical working groups and MoHs in each country. Training for 1–2 providers per facility is conducted in line with adult learning principles, incorporating practical sessions using the devices with clinical vignettes and with patients in health facilities. Post-training tests determine immediate knowledge and skills acquisition, and prompt supportive guidance and reinforcement on correct use if needed. Training for additional staff in facilities is conducted using a cascade approach, with in-person mentorship follow-up; training for new staff is provided either in a group or individually, depending on circumstances.

IMCI refresher training is provided given that many healthcare providers had not been trained recently or at all; it is delivered to providers in all arms of the RCT to prevent possible bias from a training effect and was delivered as part of the integrated training package in Kenya and Senegal.

### Monitoring, evaluation and supportive supervision

An initial mentoring and supervision visit is conducted within the first few weeks following training, with subsequent routine visit(s) at least quarterly according to MoH supervision mechanisms, integrating use of routine registry data, and data on device use for relevant facilities for monitoring and evaluation.

### Community engagement

Community engagement through local civil society organisations and community health initiatives complements the facility-based intervention package (including communities surrounding both pre-intervention and control facilities). Though mechanisms vary by country, this includes both information giving (on childhood illness, including recognising danger signs) and demand generation for health services including project interventions through promotion of health-seeking behaviour, messaging on the importance of adhering to referral advice and engagement on project interventions

### Outcomes

If the intervention is successful, we expect that healthcare providers equipped with pulse oximetry are better able to identify children with hypoxaemia, provide urgent pre-referral treatment and refer them to a higher level of care for further treatment and supportive care, resulting in improved clinical outcomes. Alongside this, we anticipate that healthcare providers equipped with CDSAs better adhere to IMCI and other relevant child health guidelines, thus improving both detection and management of severe and non-severe illness, leading to improved outcomes and antimicrobial and other resource stewardship.

The primary and secondary outcomes for the RCT and pre-post study, and outcomes for sub-studies, were developed through engagement with MoHs, WHO and other key stakeholders. Though the outcomes at the primary care level are more directly attributable to the intervention, stakeholders including WHO and MoHs emphasised the importance of understanding the impact of pulse oximetry and CDSAs on clinical and health outcomes, rather than only on process outcomes, to inform decisions about scale-up. Selecting relevant clinical and health outcomes to address this evidence gap was challenging. Mortality is fortunately an extremely rare event in primary care and therefore was not feasible to assess alone. Overall hospital attendance and hospitalisation are not suitable, because whilst the intervention should increase appropriate hospitalisation, it should decrease inappropriate hospitalisation (for non-severe disease) and late hospitalisation (particularly through earlier appropriate treatment for moderate illness as a result of use of the CDSA).

Through stakeholder engagement and consultation with the project's International Advisory Group, we therefore selected two primary outcomes for the RCT:

- Severe complications by Day 7 (mortality and 'secondary hospitalisations' i.e. delayed ≥24 hours from the Day 0 consultation, or without referral), expected to be reduced as a result of the intervention
- 'Primary' hospitalisations (within 24 h of the Day 0 consultation and with referral), expected to increase as a result of the intervention

The study population is all enrolled children for the primary analysis. Given the pragmatic nature of the intervention, we felt that it was important to minimise the influence of the research on healthcare provider behaviour, so as to get as close as possible to measuring the impact of the intervention package. Identifying the 'highest risk' children would have required an independent clinical assessment or observation of the consultation and potentially resulted in a need to

modify proposed management if incorrect and thus influencing subsequent outcomes. Using the sub-group of severely ill children based on healthcare provider reported information, particularly diagnosis, would likely result in biased results as the intervention itself influences healthcare provider classification, i.e. we may expect to find a greater proportion of children with severe disease in the intervention arm, but this does not imply that there are truly a greater proportion, rather a greater proportion identified (likely to have different characteristics). However, given the importance of the evaluation of these 'highest risk' children, we plan a sub-group analysis. Finally, acknowledging that these outcomes are highly contingent on hospital care quality and many other barriers to referral completion [47] and are relatively rare events requiring large sample sizes to demonstrate effectiveness, they were selected as primary outcomes for the RCT and secondary outcomes for the pre-post study.

Two primary outcomes are assessed for the quasi-experimental pre-post study: referrals to a higher level of care at Day 0 consultation and antibiotic prescriptions on Day 0. These reflect the aim that the intervention increases detection of severe disease, and therefore increasing referral, whilst promoting antimicrobial stewardship, and therefore reducing antibiotic prescription. We chose to assess the overall antibiotic prescription, rather than 'appropriate' prescription for several reasons. Appropriate antibiotic prescription is challenging to accurately assess, particularly in the context of a large-scale pragmatic study. Appropriateness can be considered against a 'gold standard' assessment of the clinical condition of the child, or against healthcare provider recorded diagnosis. The former would require an observation or second assessment of the child (very resource intensive to conduct at large scale), whilst the latter poses a challenge because the intervention itself is anticipated to modify the appropriateness of diagnoses. Antibiotic prescription by healthcare providers is commonly very high (50–80%) for sick children attending primary care. Whilst the proportion of children who truly require antibiotics varies with epidemiological differences across settings, WHO recommends that prescription rates should be lower than 30% (and studies since then indicate that this should be substantially lower still, given the predominance of viral aetiology for childhood infections in primary care in resource-constrained settings). We therefore chose overall prescription as an important indicator of antibiotic use which can be assessed with a relatively high degree of certainty within a large scale pragmatic study, whilst also evaluating appropriate antibiotic prescription as a secondary outcome and within the SPA sub-study. Referral and antibiotic prescription are included as secondary outcomes for the RCT.

Other secondary outcomes for the RCT and pre-post studies, relating to hypoxaemia, referral, antimicrobial prescription, follow-up, and health status, are further detailed in the full protocol and statistical analysis plan available in the trial registries. A high-level summary of these outcomes, and those of the embedded mixed-method studies are described in Figure 1.

## Participant timeline

The study flowchart is presented in Figure 3. For the RCT and pre-post study, research assistants (RAs) enrol participants at study facilities following informed consent, and collect information from the caregiver prior to and after consultation, and extract information from clinical records. If the child is critically unwell, recruitment is attempted only if the child is first stabilised. Follow-up at Day 7 (and Day 28 for the RCT) is conducted by phone with community follow-up mechanisms for caregivers unreachable by phone in Tanzania. Where available, RAs collect information from hospital (or inpatient primary care) records for children reported to have attended hospital (or admitted to a primary care facility) or lost to follow-up at Day 7. During the follow-up period, basic visit information is also recorded for children returning to their enrolment facility (or any other study facility).

During SPA recruitment periods, children and caregivers are recruited simultaneously to the SPA and RCT/pre-post study and, if enrolled, undergo two additional time-points of data collection – during the consultation (observation), and after (exit interview). When relevant, simultaneous recruitment also occurs for the time-flow study and RCT/pre-post study; if enrolled, time data is collected throughout the facility visit. IDIs with participating caregivers take place after the day 7 follow-up phone call.

Healthcare providers may be recruited to one or more of the SPA, qualitative and cost studies depending on sampling considerations, and may be included in one or more rounds of assessment depending on staff rotation, and sampling considerations for the qualitative studies. Key informants are invited to an online survey at different time points and, in Kenya and Senegal, a sub-set are also invited to KIIs.

## Sample size, recruitment & allocation

### Sample size

Sample sizes were calculated separately for each country.

The RCT sample size was estimated based on planned enrolment over 12 months and ability to detect a ≥30% decrease in severe complications (from 1.1% [22]) and ≥30% increase in primary

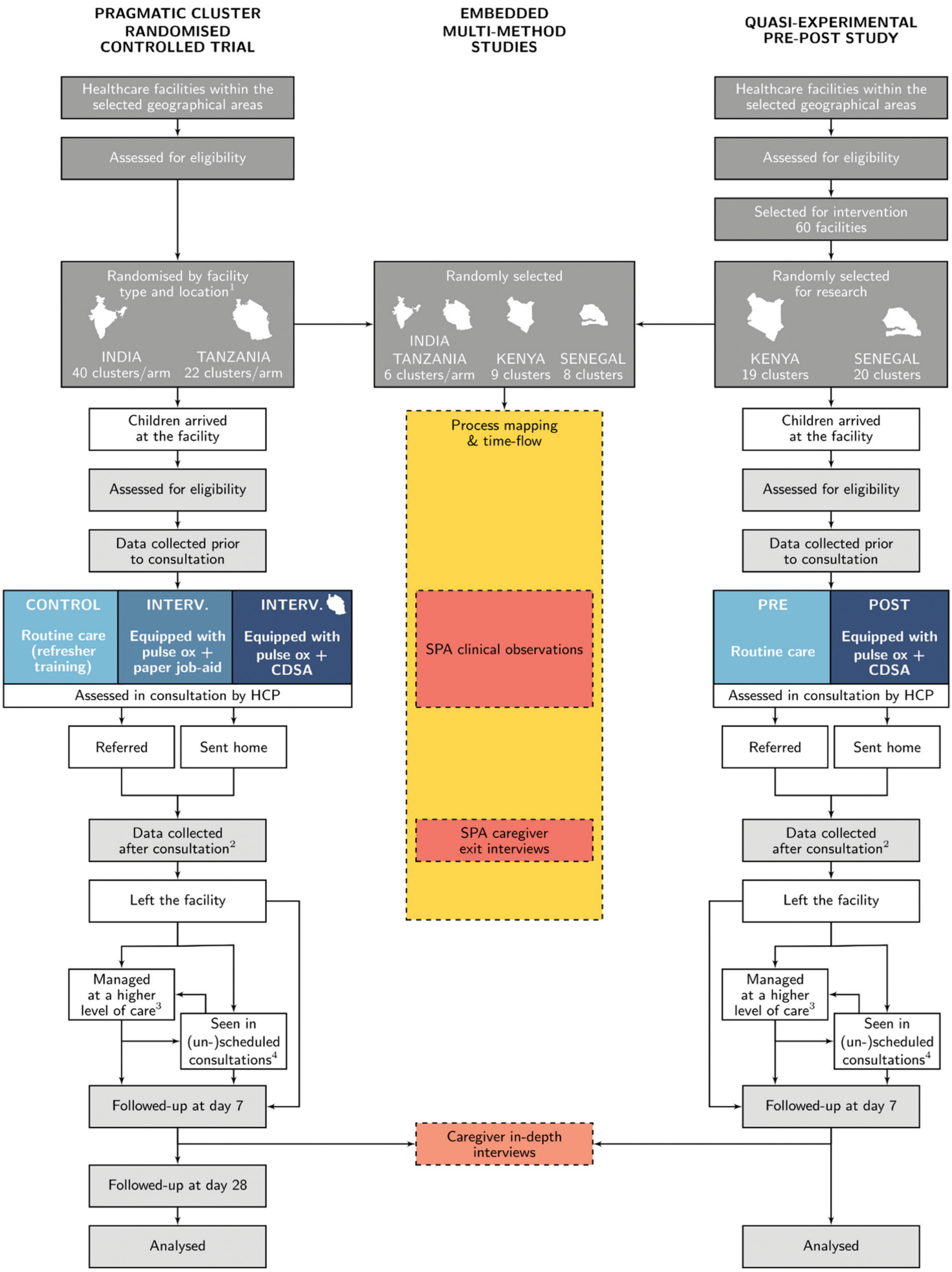


**Figure 3.** Study flowcharts of the pragmatic cluster RCT and quasi-experimental pre-post study, with interconnections with the embedded mixed-methods studies involving caregivers and children. (1) Location refers to urban/rural for Tanzania and to districts in India. (2) Data collected after consultations include caregiver responses at consultation exit and clinical records. (3) In all countries other than Kenya, data are collected from hospital (or primary care admission area) records for all children reported to have attended a hospital/admission facility (4) If children return to their enrolment facility (or attend any other study facility) during the follow-up period, data about the visit is collected, which includes the same information as gathered on Day 0.

hospitalisations (from 1.5%, based on facility estimates) for each arm compared to control with 80% power, 0.05 alpha per arm, and intra-cluster correlation coefficient (ICC) of 0.001 [48]. Anticipated feasible enrolment rates were based on DHIS2 and facility data. In Tanzania, 22 clusters per

arm, each recruiting an average of 1680 children, were estimated to be needed (total 110,880). In India, 40 clusters per arm, each recruiting an average of 510 children, were estimated to be needed (total 40,800).

The pre-post study sample size was originally estimated for a planned 15-month study, with 3 months pre- (Q1) and 12 months post-intervention (Q2–5), with comparison of Q1 and Q5 for the primary outcome. Calculations were based on detecting a ≥50% increase in Day 0 referrals (from 3%, based on DHIS2, SPA and facility estimates), with 80% power, 0.05 alpha and ICC of 0.005 [48]. A relatively large detectable difference was chosen given that a relatively high proportion of referrals may not be completed [5,47]. Day 0 referrals, rather than antibiotic prescriptions (with baseline estimated at 60% with ICC 0.05), drove the sample size calculation. We estimated needing 17 facilities, recruiting an average of 690 children per facility per 3-month period in Kenya, and 18 facilities recruiting 510 children per facility per period in Senegal.

Following a lower than anticipated recruitment in the baseline of the pre-post study, sample size was re-evaluated resulting in a decision to add two facilities per country, extend the baseline (to 6–7 months) and reduce the post-intervention period (to 9–10 months). A minimum of 7429 and 6760 children were estimated to be needed in each period in Kenya and Senegal, respectively, with recruitment continuing after meeting the minimum sample size in order to allow for description of changes over time and with overlapping seasonal periods, in line with the intention of the original design.

Sample sizes for other studies were chosen pragmatically to explore differences between non-intervention and intervention facilities and over time within the intervention period. Sample sizes for qualitative studies were based on reaching thematic saturation, as outlined in Table 2.

### Recruitment

Strategies to ensure adequate enrolment following the pilot include weekly reviews of automated monitoring reports and review of assumptions during RCT 545 interim analysis. Potential interventions include actions to increase recruitment at study facilities, increasing facility numbers or study duration.

### Allocation

Clusters are allocated 1:1:1 in Tanzania and 1:1 in India from the list of eligible facilities for the RCT by an independent statistician, stratified by facility type (Primary Health Centre/Community Health Centre for India, and dispensary/health centre for Tanzania) and location (district in India, and urban/rural in Tanzania). Unallocated eligible facilities are retained as back-ups for later allocation if needed. Given the cluster design, concealment only occurs at facility allocation, conducted centrally and distributed to study sites.

**Table 2.** Estimated sample sizes for each of the TIMCI sub-studies.

| | RCT & embedded studies | | Pre-post study & embedded studies | |
|---|---|---|---|---|
| Study | India | Tanzania | Kenya | Senegal |
| RCT/pre-post study | 80 clusters (40/arm)<br>510 children/cluster<br>Target = 40800 children | 66 clusters (22/arm)<br>1680 children/cluster<br>Target = 110,880 children | 19 clusters<br>391 children/cluster/period<br>Target = min. 14858* | 20 clusters<br>339 children/cluster/period<br>Target = min. 13558* |
| Service Provision Assessments | 12 clusters (6/arm)<br>5 rounds (pre, quarterly in RCT) | 18 clusters (6/arm)<br>5 rounds (pre, quarterly in RCT | 9 clusters<br>3 rounds (pre, early, late) | 8 clusters<br>3 rounds (pre, early, late) |
| | 2–3 HCP interviews & 10–30 observations & exit interviews/facility/assessment round | | | |
| Process mapping + time-flow study | 6 clusters (pulse ox arm)<br>3 rounds (pre, early, late) | 18 clusters (6/arm)<br>2 rounds (early, late) | 9 clusters<br>3 rounds (pre, early, late) | 8 clusters<br>2 rounds (pre, late) |
| | 10–30 observations for each process map and time-flow/facility/assessment round | | | |
| Healthcare Provider IDIs | 12–15 IDIs in intervention arm per period (early, late) | 12–15 IDIs per intervention arm per period (early, late) | 12–15 IDIs per period (early, late) | 12–15 IDIs per period (pre, late) |
| Caregiver IDIs | 12–15 IDIs in pulse oximetry arm/period (early, late) | 12–15 IDIs per intervention arm/period (early, late) | 12–15 IDIs per period (early, late) | 12–15 IDIs per period (pre, late) |
| Stakeholder survey | Approx. 15–20 per period (pre, early, late)/country + global | | | |
| Stakeholder KIIs | N/A | N/A | 12–15 KIIs for each round (early, late) | N/A |
| Costing study | 8 clusters per arm | 8 clusters per arm | 8 clusters<br>Pre- and post-intervention | 8 clusters<br>Pre- and post-intervention |

Abbreviations: PHC: primary health centre; CHC: community health centre; HP: health posts (poste de santé); HCs: health centres; DH: district hospital; O2: oxygen; SpO2: oxygen saturation; IM(N)CI: integrated management of (neonatal and) childhood illness; ENT: ear, nose, throat; MSK: musculoskeletal; MoH: ministry of health; DoH: department of health; SC: sub-country; CHMT: community health management team; ASHA: government community health worker; CSO: civil society organisation; CHV: community health volunteer; CHW: community health worker.

## Data collection, management & analysis

### Data collection

Data collected within the TIMCI study are described in the supplementary material. Quantitative data are collected by trained, generally non-clinical, RAs using tablet-based structured Open Data Kit (ODK) questionnaires. RCT and pre-post study Day 0 data, collected from caregivers and clinical records, include sociodemographic details, reason for attendance, prior care-seeking, assessments performed, diagnoses made, and management provided. Personally identifiable information (PII) is collected for follow-up, linking data and detecting possible duplicates. Caregiver-reported health status and care-seeking since Day 0 consultation are collected by phone on Day 7 (and 28 in the RCT only), in line with previous studies conducted by our group, and other large-scale pragmatic trials of primary care interventions [21,23,49]. Further attempts are made on at least 3 subsequent days if the initial attempt fails and the number is valid. In Tanzania, community follow-up via community health worker phone or in-person by RAs is conducted for caregivers unreachable by phone. Visit information is recorded for children who attend any study facility during the follow-up period. Where indicated, hospitalisation data (basic clinical, admission and outcome data) are collected periodically from clinical records.

Additional data are collected for SPA studies during and after the clinical consultation. Trained clinical observers collect standardised data on assessment, diagnosis and management, including IMCI, pulse oximetry and CDSA adherence (when applicable). Observers do not intervene, except (provided they have sufficient experience and expertise) if they witness or suspect an imminent error 'highly likely to result in direct, severe or irreversible harm', which could be mitigated by their intervention [50]. The exit interview RA collects information on caregiver experience of care, and additional sociodemographic details and post-consultation plans.

SPA healthcare provider interviews gather information on qualifications, training and experience, and facility assessments on infrastructure, staffing, services, consumables, recording and reporting with a focus on factors relevant to child health, pulse oximetry, and digital health.

Process mapping data, based on non-participant observation in different areas of the facilities, individual patient shadowing, and input from facility staff, are collected by RAs trained for qualitative research and recorded as notes according to a structured template, along with drawn maps. Time data are collected using ODK forms, with automated timing of steps, by RAs who follow individual children from facility arrival to exit.

Qualitative data including sociodemographic data and voice recordings from IDIs and KIIs are collected by trained RAs using a semi-structured interview guide, with open-ended questions and probes. Online survey data is collected via ODK and a link sent via email directly to respondents.

Cost data are collected by or under the supervision of health economists from government databases, facility records, information provided by medical and non-medical personnel, supplemented by data from other sub-studies and the literature where necessary. This follows an activity-based approach, with three cost centres: training for staff involved in delivering the programme, delivery of the intervention (with annualised capital costs), and selected out-of-pocket costs for referred patients.

De-identified CDSA data and health management information system (HMIS) data are extracted for all TIMCI facilities (in Kenya and Senegal, this includes data from all intervention facilities).

### Data management

Data management is standardised globally via Standard Operating Procedures (SOPs) and implemented and supervised by data managers in each country.

Research and CDSA data are centralised on dedicated secured servers hosted and maintained in each country. Data synchronisation occurs on a daily basis. To maintain privacy and data security, exported data are segregated into de-identified study databases and encrypted PII databases. The latter is required for updating follow-up participant logs, reconciling participant records and identifying duplicates, and will be destroyed after study completion and data validation.

Quality procedures are maintained throughout the entire lifecycle of quantitative research data. To maintain data integrity, an audit trail is established from the moment data are entered until they are exported to final study databases. To ensure data accuracy and completeness, ODK questionnaires incorporate offline validation checks that detect and prompt RAs to correct any erroneous data at the point of entry and prevent the finalisation of questionnaires with missing entries. Automated data quality findings and study conduct indicators are generated and communicated to study teams on a daily basis to proactively identify any problems with the data collection.

CDSA consultation data are reconciled with RCT and pre-post records using fuzzy PII matching approaches. All non-reconciled CDSA consultation data are fully anonymised.

Qualitative data are fully de-identified before coding; audio files (collected on encrypted devices) are destroyed after quality checking of transcription and translation and the remaining raw material is destroyed after validation of the final de-identified material.

### Data analysis

Intention-to-treat analyses are planned on individual and combined cross-country data. Baseline characteristics and outcomes will be described by study arm (RCT) and pre-post periods (pre-post study), with summary statistics. Primary outcomes will be assessed using a random effects logistic regression model with the cluster (facility) included as a random effect. Modelling of secondary outcomes will be performed in a similar way if numbers allow. Binary outcomes will be reported with odds ratios, risk differences and 95% confidence intervals (CI), continuous outcomes with adjusted mean differences and 95% CIs.

Models will be adjusted for stratification factors and baseline variables randomly imbalanced across arms (RCT) and for pre-specified potential confounding baseline characteristics (pre-post). Only individual-level baseline variables will be used for models adjustment.

RCT primary outcomes will be evaluated with a hierarchical fallback procedure which uses a weighted Bonferroni calculation, recycling unspent significant levels to test pre-specified subsequent hypotheses [51–53]. For each intervention arm, the trial will be interpreted as positive if either primary outcome is positive compared to control, with no indication of harm from the non-significant outcome. The primary outcomes for the pre-post study will be assessed independently with no adjustment planned.

Primary outcomes subgroup analyses are planned to assess the effect modification of age, sex, and clinical presentation for both RCT and pre-post study, and of diagnosis, referral and antimicrobial prescriptions for the RCT only. Sensitivity analyses are planned for only the first disease episode of each child during the study period and in case of substantial missing data (best/worst case scenario, complete cases, multiple imputation with chained equations) [54,55]. Machine learning methods will be used to identify prognostic features associated with outcomes.

Descriptive analyses will be conducted for the SPA, including adherence to key practices and time-flow study (visit, consultation and other care process steps), with comparison between non-intervention (pre-, control) and intervention facilities, and between early and late intervention periods. Effect modifiers will be explored. CDSA data will be analysed descriptively, including a detailed description on the use of pulse oximetry and prevalence of hypoxaemia in consultations, with univariate and multivariate analyses to assess factors associated with hypoxaemia. Aggregated routine HMIS data will be used to monitor trends over time in all facilities and administrative regions where the project is running.

Qualitative data will be transcribed and translated, with quality assurance of a random sample. After familiarisation with the data, two qualitative researchers will independently code the same random sample of 10–15% of the transcripts using the same code tree following Gale et al.'s framework analysis [56]. Interrater reliability will be assessed with the Kappa-Cohen value; coding differences will be discussed and a joint solution developed. The finalised code tree will be used to code all data. Finally, tables of coded data will be reviewed by a team of qualitative researchers to jointly analyse the data identifying patterns, similarities, and differences, as well as change over time. Secondary analysis of data may be conducted for comparison across countries and to contextualise quantitative and observational data. Process evaluation will draw on data from the various sub-studies to describe the context, implementation process and mechanisms of impact.

Cost data will be used to derive total and unit costs of the programme from a health system perspective. Total annual costs (from the first 3 years of the intervention) and incremental annual costs (for maintaining the programme) will be estimated. Costs incurred by patients and their families will be briefly described. Costs associated with changing the oxygen saturation referral threshold will be estimated. When available, effectiveness data will be used to model cost per DALY averted.

### RCT monitoring

All studies are conducted in accordance with the protocol and applicable international and national regulatory requirements. The RCT is monitored in accordance with the International Conference on Harmonisation Good Clinical Practice through independent remote and on-site monitoring, with audits triggered in case of trial conduct concerns. An independent Data Monitoring Committee (DMC) reviews open and closed interim analysis reports (conducted 3 months after the RCT start, to assess recruitment rate, follow-up and sample size assumptions to determine need for adjustment, but with no hypothesis testing), and progress reports, to provide recommendations in order to safeguard participants and ensure the integrity and relevance of results. The DMC Charter can be found in the trial registry.

We collect data on deaths and secondary hospitalisations as part of the primary outcome, but do not otherwise collect individual adverse event reports. Given the low-risk nature of the intervention, the pragmatic nature of the trial and that deaths and hospitalisations are, unfortunately, expected in this population, the DMC and monitors review summarised rates of deaths and hospitalisations, prompting more in-depth evaluation if required.

### *Patient and public involvement*

The research questions, study design and outcome measures were developed through consultation and engagement with MoHs, community members through civil society organisations (CSOs) and community advisory boards, WHO experts, and an International Advisory Group. Informed consent mechanisms and content were reviewed with community advisory boards and/or participants and refined as necessary based on piloting. During the pilot, communities and CSOs were further consulted to determine the best approaches to recruitment within health facilities, and to gather feedback on the burden of time for involvement in research, after which questionnaires were revised and reduced. In Tanzania, the CSOs were engaged to understand the best approaches to community-based follow-up. Communities and other stakeholders will be engaged in dissemination and have been involved in the development of the dissemination plan.

## Dissemination

The final, anonymised RCT and pre-post datasets will be made available on an open access data sharing platform after the end of the study, in order to promote transparency and facilitate global cooperation in child health research (see data sharing plan in study registries).

Findings from the study will be shared with caregivers through community engagement mechanisms, and with healthcare providers, local, sub-national and national stakeholders (including MoHs, with whom the project is conducted in close collaboration) through a series of engagement and dissemination meetings in each country. At the global level, engagement is conducted with technical partners including WHO and UNICEF. Through the project's 'observer country' network, findings are also shared with MoHs in other countries to inform decisions about implementation of interventions. Results will be shared with the scientific community through presentations at conferences and open-access peer-reviewed journal publications, among others.

## Conclusion

This multi-country study represents the largest scale evaluation to date of pulse oximetry and CDSAs to support healthcare providers in assessing and managing sick children in primary care in resource-constrained settings. The mixed-method design will provide comprehensive insights into the health and quality of care impact, diagnostic and medicine stewardship, acceptability, feasibility, cost, and cost-effectiveness of these interventions. Although the pragmatic nature of the study increases the potential for lower intervention fidelity relative to a tightly controlled study, it will better reflect the expected implementation of the intervention in real-world settings and thus provide critical insights into the scalability of these tools. The anticipated results will inform decision-making on pulse oximetry and CDSAs and, more broadly, contribute insights into disease burden and care pathways to inform future strategies addressing preventable morbidity and mortality among sick children attending primary care in resource-constrained settings.

## Acknowledgments

The authors would like to thank Ministries of Health for their engagement in the design of the project and study; Institutional Review Boards and Ethics Committees for their review and approval; Shamim Qazi, Yasir Bin Nisar and Wilson Were for their inputs on WHO evidence-generation priorities; and the Scientific Merit Reviewers for their thoughtful and valuable review.

In addition, the authors would like to recognise the important contribution of the Burnet Institute and PATH's Myanmar team, who were involved in the early stages of protocol development and adaptation for Myanmar, where the study was unfortunately unable to be conducted due to the challenging political situation in the country.

## Disclosure statement

No potential conflict of interest was reported by the author(s).

## Funding information

This work is supported by Unitaid as part of the Tools for Integrated Management of Childhood Illness (TIMCI) project under grant number n°2019-35-TIMCI to PATH

## Authors' contributions

KW, VD, KK, MR, HLS, HM, IM, ON, SA, JM, SH led the overall TIMCI project proposal for funding acquisition. The study design was developed collaboratively during a week-long in-person workshop, with representatives from Ifakara Health Institute, King George's Medical University, Universite Cheikh Anta Diop, University of Nairobi, University of Waterloo, Indian Council of Medical Research, Ministère de la Santé et de l'Action Sociale Sénégal, Ministry of Health and Social Welfare of Tanzania, Ministry of Health Kenya, and PATH headquarter and country teams, facilitated by Swiss Tropical and Public Health Institute (Swiss TPH) investigators.

## Ethics and consent

Following two independent scientific merit reviews, the protocols (global and national adaptations) and subsequent amendments were submitted to and approved by the WHO Ethics Review Committee (Ref ERC.0003405, v2.4, 2 February 2023, RCT and sub-studies, India and

Tanzania; and Ref ERC.0003406, v2.5, 5 April 2023, pre-post study and sub-studies, Kenya & Senegal); the King George's Medical University Internal Ethics Committee Ref. ECR/262/Inst/UP/2013/RR-16 and the Indian Council of Medical Research Ref 2020–9753 (India); the Ifakara Health Institute Institutional Review Board Ref IHI/IRB/AMM/01–2023 and the National Institute for Medical Research Ref NIMR/HQ/R.8c/Vol. I/2265 (Tanzania); the Kenyatta National Hospital Ethic Review Committee Ref P333/06/2020, KNH/ERC/R/235 (Kenya); and the Comité National d'Ethique pour la Recherche en Santé ref SEN20/50 (Senegal). Approvals were also sought from relevant national and regional authorities and facilities prior to the start of the study. All information and consent procedures are conducted in accordance with international and national regulatory and ethical requirements.

Written informed consent is sought from caregivers of children eligible for all quantitative studies on Day 0 at study facilities. If the caregiver is illiterate, information is read aloud and an impartial witness (present during consent) signs and, except in Senegal, the caregiver provides a thumbprint. For children requiring urgent clinical care, informed consent is only conducted if the child is first stabilised. Continued consent is checked orally at follow-up; oral consent is sought for process mapping.

Oral consent to approach caregivers for qualitative studies is obtained, with written informed consent in person prior to participation. Written informed consent is sought from healthcare providers (and other participants as relevant) for the SPA, IDIs, KIIs and costing studies, with continued consent assessed orally at each observation. Consent for survey participation is provided online.

Participation for all studies is voluntary; no incentives to participate are provided, and withdrawal is possible up to completion of the study and anonymisation. Caregivers are informed that non-participation will not affect the medical care their child will receive.

PII is collected solely for the purpose of the study and will be destroyed in accordance with SOPs. Data are handled confidentially and are only accessible to authorised personnel requiring the data to fulfil study duties.

## Roles and responsibilities

The funder of the TIMCI project is Unitaid; PATH is the grant recipient, with country teams leading implementation activities in close collaboration with the Ministries of Health. As the Sponsor of the study, PATH delegated Sponsor responsibilities to Swiss TPH, who leads the research at the global level. The economic analysis is led by the University of Waterloo. The development, adaptation and maintenance of the clinical decision support software are led by Unisanté, University of Lausanne. Research in individual countries is led by Ifakara Health Institute in Tanzania, King George's Medical University in India, Université Cheikh Anta Diop in Senegal, and University of Nairobi in Kenya.

Research governance structures include a Research Steering Committee (RSC), chaired by an independent expert, and working/management groups within countries and cross-country according to the study, and an independent Data Monitoring Committee (DMC) for the randomised controlled trial. The overall TIMCI project also draws on the guidance of an International Advisory Group (IAG), comprised of international – experts, a shared IAG with the Améliorer l'Identification des Détresses Respiratoires chez l'Enfant (AIRE) project, a parallel initiative funded by Unitaid and led by the Alliance for International Medical Action (ALIMA). The funder, Unitaid, does not have any role in the study design, conduct or analysis, nor in the decision to submit for publication.

Sponsor contact PATH: Mike Ruffo (mruffo@path.org); Sponsor contact Swiss TPH: Kaspar Wyss (kaspar.wyss@swisstph.ch).

## ORCID

Fenella Beynon http://orcid.org/0000-0002-4758-8847
Hélène Langet http://orcid.org/0000-0002-6758-2397
Leah F. Bohle http://orcid.org/0009-0004-9324-4959
Shally Awasthi http://orcid.org/0000-0003-1254-9802
Ousmane Ndiaye http://orcid.org/0000-0003-3049-5203
James Machoki M'Imunya http://orcid.org/0000-0002-8792-6812
Honorati Masanja http://orcid.org/0000-0002-3860-7348
Susan Horton http://orcid.org/0000-0002-9243-4767
Silvia Cicconi http://orcid.org/0000-0001-5507-6203
Mira Emmanuel-Fabula http://orcid.org/0000-0003-3117-767X
Papa Moctar Faye http://orcid.org/0000-0002-1493-0597
Tracy R. Glass http://orcid.org/0000-0001-7075-9803
Kristina Keitel http://orcid.org/0000-0002-9663-3843
Divas Kumar http://orcid.org/0000-0002-4522-728X
Gillian A. Levine http://orcid.org/0000-0002-9884-9592
Lena Matata http://orcid.org/0000-0001-6784-0264
Deusdedit Mjungu http://orcid.org/0000-0002-0417-3027
Francis Njiri http://orcid.org/0000-0002-1292-2809
Fabian Schär http://orcid.org/0000-0002-1182-051X
Helen L. Storey http://orcid.org/0000-0001-6263-2749
Kaspar Wyss http://orcid.org/0000-0003-0156-5989
Valérie D'Acremont http://orcid.org/0000-0002-4881-7787

## References

[1] UN IGME. Levels & trends in child mortality report 2022: estimates developed by the united nations inter-agency group for child mortality estimation. New York: UNICEF; 2023.

[2] Costello AM, Dalglish SL, on behalf of the Strategic Review Study Team. Towards a grand convergence for child survival and health: a strategic review of options for the future building on lessons learnt from IMNCI. Geneva: WHO; 2016.

[3] World Health Organization. Integrated management of childhood illness: chart booklet. Geneva: WHO; 2014.

[4] Graham HR, Kamuntu Y, Miller J, Barrett A, Kunihira B, Engol S, et al. Hypoxaemia prevalence and management among children and adults presenting to primary care facilities in Uganda: a prospective cohort study. PLoS Glob Public Health. 2022;2: e0000352. doi: 10.1371/journal.pgph.0000352

[5] Colbourn T, King C, Beard J, Phiri T, Mdala M, Zadutsa B, et al. Predictive value of pulse oximetry for mortality in infants and children presenting to primary care with clinical pneumonia in rural

Malawi: a data linkage study. PLoS Med. 2020 Oct;17:e1003300. doi: 10.1371/journal.pmed.1003300
[6] Tesfaye SH, Gebeyehu Y, Loha E, Johansson KA, Lindtjørn B. Pulse oximeter with integrated management of childhood illness for diagnosis of severe childhood pneumonia at rural health institutions in Southern Ethiopia: results from a cluster-randomised controlled trial. BMJ Open. 2020 Jun 21;10:e036814. doi: 10.1136/bmjopen-2020-036814
[7] Lazzerini M, Sonego M, Pellegrin MC, Bhutta ZA. Hypoxaemia as a mortality risk factor in acute lower respiratory infections in children in low and middle-income countries: systematic review and meta-analysis. PLoS One. 2015;10:e0136166. doi: 10.1371/journal.pone.0136166
[8] Rojas-Reyes MX, Granados Rugeles C, Charry-Anzola LP. Oxygen therapy for lower respiratory tract infections in children between 3 months and 15 years of age. Cochrane Database Syst Rev. 2014 Dec 10;2014:Cd005975. doi: 10.1002/14651858.CD005975.pub3
[9] Duke T, Subhi R, Peel D, Frey B. Pulse oximetry: technology to reduce child mortality in developing countries. Ann Trop Paediatr. 2009 Sep;29:165–175. doi: 10.1179/027249309X12467994190011
[10] Ginsburg AS, Van Cleve WC, Thompson MI, English M. Oxygen and pulse oximetry in childhood pneumonia: a survey of healthcare providers in resource-limited settings. J Trop Pediatr. 2012 Oct;58:389–393. doi: 10.1093/tropej/fmr103
[11] Floyd J, Wu L, Hay Burgess D, Izadnegahdar R, Mukanga D, Ghani AC. Evaluating the impact of pulse oximetry on childhood pneumonia mortality in resource-poor settings. Nature. 2015 Dec 3;528:S53–9. doi: 10.1038/nature16043
[12] McCollum ED, King C, Deula R, Zadutsa B, Mankhambo L, Nambiar B, et al. Pulse oximetry for children with pneumonia treated as outpatients in rural Malawi. Bull World Health Organ. 2016 Dec 1;94:893–902. doi: 10.2471/BLT.16.173401
[13] Orimadegun AE, Ogunbosi BO, Carson SS. Prevalence and predictors of hypoxaemia in respiratory and non-respiratory primary diagnoses among emergently ill children at a tertiary hospital in South Western Nigeria. Trans R Soc Trop Med Hyg. 2013 Nov;107:699–705. doi: 10.1093/trstmh/trt082
[14] Graham H, Bakare AA, Ayede AI, Oyewole OB, Gray A, Peel D, et al. Hypoxaemia in hospitalised children and neonates: a prospective cohort study in Nigerian secondary-level hospitals. EClinicalMedicine. 2019 Nov;16:51–63. doi: 10.1016/j.eclinm.2019.10.009
[15] Chew R, Zhang M, Chandna A, Lubell Y. The impact of pulse oximetry on diagnosis, management and outcomes of acute febrile illness in low-income and middle-income countries: a systematic review. BMJ Glob Health. 2021 Nov;6:e007282. doi: 10.1136/bmjgh-2021-007282
[16] Kruger C, Heinzel-Gutenbrunner M, Ali M. Adherence to the integrated management of childhood illness guidelines in Namibia, Kenya, Tanzania and Uganda: evidence from the national service provision assessment surveys. BMC Health Serv Res. 2017 Dec 13;17:822. doi: 10.1186/s12913-017-2781-3
[17] Horwood C, Voce A, Vermaak K, Rollins N, Qazi S. Experiences of training and implementation of integrated management of childhood illness (IMCI) in South Africa: a qualitative evaluation of the IMCI case management training course. BMC Pediatr. 2009 Oct 1;9:62. doi: 10.1186/1471-2431-9-62
[18] Lange S, Mwisongo A, Maestad O. Why don't clinicians adhere more consistently to guidelines for the integrated management of childhood illness (IMCI)? Soc Sci Med. 2014 Mar;104:56–63. doi: 10.1016/j.socscimed.2013.12.020
[19] Bjornstad E, Preidis GA, Lufesi N, Olson D, Kamthunzi P, Hosseinipour MC, et al. Determining the quality of IMCI pneumonia care in Malawian children. Paediatr Int Child Health. 2014 Feb;34:29–36.
[20] World Health Organization. WHO guideline: recommendations on digital interventions for health system strengthening. Geneva: WHO; 2019.
[21] Shao AF, Rambaud-Althaus C, Samaka J, Faustine AF, Perri-Moore S, Swai N, et al. New algorithm for managing childhood illness using mobile technology (ALMANACH): a controlled non-inferiority study on clinical outcome and antibiotic use in Tanzania. PLoS One. 2015;10:e0132316. doi: 10.1371/journal.pone.0132316
[22] Keitel K, Kagoro F, Samaka J, Masimba J, Said Z, Temba H, et al. A novel electronic algorithm using host biomarker point-of-care tests for the management of febrile illnesses in Tanzanian children (e-POCT): a randomized, controlled non-inferiority trial. PLoS Med. 2017 Oct;14:e1002411. doi: 10.1371/journal.pmed.1002411
[23] Schmitz T, Beynon F, Musard C, Kwiatkowski M, Landi M, Ishaya D, et al. Effectiveness of an electronic clinical decision support system in improving the management of childhood illness in primary care in rural Nigeria: an observational study. BMJ Open. 2022 Jul 21;12:e055315. doi: 10.1136/bmjopen-2021-055315
[24] Sarrassat S, Lewis JJ, Some AS, Somda S, Cousens S, Blanchet K. An integrated eDiagnosis approach (IeDA) versus standard IMCI for assessing and managing childhood illness in Burkina Faso: a stepped-wedge cluster randomised trial. BMC Health Serv Res. 2021 Apr 16;21:354. doi: 10.1186/s12913-021-06317-3
[25] Soremekun S, Kallander K, Lingam R, Branco AC, Batura N, Strachan DL, et al. Improving outcomes for children with malaria, diarrhoea and pneumonia in Mozambique: a cluster randomised controlled trial of the inSCALE technology innovation. PLoS Digital Health. 2023 Jun;2:e0000235.
[26] Agarwal S, Glenton C, Tamrat T, Henschke N, Maayan N, Fonhus MS, et al. Decision-support tools via mobile devices to improve quality of care in primary healthcare settings. Cochrane Database Syst Rev. 2021 Jul 27;7:CD012944.
[27] Odendaal WA, Anstey Watkins J, Leon N, Goudge J, Griffiths F, Tomlinson M, et al. Health workers' perceptions and experiences of using mHealth technologies to deliver primary healthcare services: a qualitative evidence synthesis. Cochrane Database Syst Rev. 2020 Mar 26;3:CD011942. doi: 10.1002/14651858.CD011942.pub2
[28] World Health Organization. Standards for improving the quality of care for children and young adolescents in health facilities. Geneva: WHO; 2018.
[29] Sekhon M, Cartwright M, Francis JJ. Acceptability of healthcare interventions: an overview of reviews and development of a theoretical framework. BMC Health Serv Res. 2017 Jan 26;17:88. doi: 10.1186/s12913-017-2031-8

[30] Michie S, van Stralen MM, West R. The behaviour change wheel: a new method for characterising and designing behaviour change interventions. Implement Sci. 2011 Apr 23;6:42.
[31] De Silva MJ, Breuer E, Lee L, Asher L, Chowdhary N, Lund C, et al. Theory of change: a theory-driven approach to enhance the medical research council's framework for complex interventions. Trials. 2014 Jul 5;15:267. doi: 10.1186/1745-6215-15-267
[32] Taplin DH, Clark H, Collins E, Colby DC. Theory of change technical papers: a series of papers to support development of theories of change based on practice in the field. New York: Center for Human Environment; 2013.
[33] Pawson R, Tilley N. Realist evaluation. London: British Cabinet Office; 2004.
[34] Moore GF, Audrey S, Barker M, Bond L, Bonell C, Hardeman W, et al. Process evaluation of complex interventions: medical research council guidance. BMJ. 2015 Mar 19;350:h1258. doi: 10.1136/bmj.h1258
[35] Chan AW, Tetzlaff JM, Gotzsche PC, Altman DG, Mann H, Berlin JA, et al. SPIRIT 2013 explanation and elaboration: guidance for protocols of clinical trials. BMJ. 2013 Jan 8;346:e7586. doi: 10.1136/bmj.e7586
[36] The DHS Program. Service provision assessment questionnaires. 2012 [cited 2019]. Available from: https://dhsprogram.com/methodology/Survey-Types/SPA.cfm
[37] India State-Level Disease Burden Initiative Child Mortality C. Subnational mapping of under-5 and neonatal mortality trends in India: the global burden of disease study 2000–17. Lancet. 2020 May 23;395:1640–1658.
[38] UNICEF. UNICEF country data profiles. 2022 [cited 2023]. Available from: https://data.unicef.org/country/
[39] Ministry of Health (MoH) [Tanzania Mainland], Ministry of Health (MoH) [Zanzibar], National Bureau of Statistics (NBS), Office of the Chief Government Statistician (OCGS), ICF. Tanzania demographic and health survey and malaria indicator survey 2022 key indicators report. Dodoma (Tanzania): MoH, NBS, OCGS, and ICF; 2023.
[40] Acare Technology Co. Ltd. Acare AH-MX. [cited 2022]. Available from: https://www.acaretech.com/product_872390.html
[41] Uwemedimo OT, Lewis TP, Essien EA, Chan GJ, Nsona H, Kruk ME, et al. Distribution and determinants of pneumonia diagnosis using integrated management of childhood illness guidelines: a nationally representative study in Malawi. BMJ Glob Health. 2018;3:e000506. doi: 10.1136/bmjgh-2017-000506
[42] Emdin CA, Mir F, Sultana S, Kazi AM, Zaidi AK, Dimitris MC, et al. Utility and feasibility of integrating pulse oximetry into the routine assessment of young infants at primary care clinics in Karachi, Pakistan: a cross-sectional study. BMC Pediatr. 2015 Sep 30;15:141.
[43] Boyd N, King C, Walker IA, Zadutsa B, Bernstein M, Ahmed S, et al. Usability testing of a reusable pulse oximeter probe developed for health-care workers caring for children < 5 years old in low-resource settings. Am J Trop Med Hyg. 2018 Oct;99:1096–1104.
[44] World Health Organization. Oxygen therapy for children. Geneva: WHO; 2016.
[45] Tan R, Cobuccio L, Beynon F, Levine GA, Vaezipour N, Luwanda LB, et al. ePOCT+ and the medAL-suite: development of an electronic clinical decision support algorithm and digital platform for pediatric outpatients in low- and middle-income countries. PLoS Digital Health. 2023 Jan;2: e0000170.
[46] The dynamic study - digital tools supporting the project: medAL software suite. [cited 2022]. Available from: https://dynamic-study.com/
[47] Ilboudo TP, Chou YJ, Huang N. Compliance with referral for curative care in rural Burkina Faso. Health Policy Plan. 2012 May;27:256–264. doi: 10.1093/heapol/czr041
[48] Pagel C, Prost A, Lewycka S, Das S, Colbourn T, Mahapatra R, et al. Intracluster correlation coefficients and coefficients of variation for perinatal outcomes from five cluster-randomised controlled trials in low and middle-income countries: results and methodological implications. Trials. 2011 Jun 14;12:151. doi: 10.1186/1745-6215-12-151
[49] Semrau KEA, Hirschhorn LR, Marx Delaney M, Singh VP, Saurastri R, Sharma N, et al. Outcomes of a coaching-based WHO safe childbirth checklist program in India. N Engl J Med. 2017 Dec 14;377:2313–2324. doi: 10.1056/NEJMoa1701075
[50] World Health Organization. Ethical issues in patient safety research. Geneva: WHO; 2013.
[51] Wiens BL. A fixed sequence Bonferroni procedure for testing multiple endpoints. Pharm Stat. 2003;2:211–215. doi: 10.1002/pst.64
[52] Wiens BL, Dmitrienko A. The fallback procedure for evaluating a single family of hypotheses. J Biopharm Stat. 2005;15:929–942. doi: 10.1080/10543400500265660
[53] Alosh M, Bretz F, Huque M. Advanced multiplicity adjustment methods in clinical trials. Stat Med. 2014 Feb 20;33:693–713. doi: 10.1002/sim.5974
[54] Jakobsen JC, Gluud C, Wetterslev J, Winkel P. When and how should multiple imputation be used for handling missing data in randomised clinical trials - a practical guide with flowcharts. BMC Med Res Methodol. 2017 Dec 6;17:162.
[55] Sullivan TR, White IR, Salter AB, Ryan P, Lee KJ. Should multiple imputation be the method of choice for handling missing data in randomized trials? Stat Methods Med Res. 2018 Sep;27:2610–2626. doi: 10.1177/0962280216683570
[56] Gale NK, Heath G, Cameron E, Rashid S, Redwood S. Using the framework method for the analysis of qualitative data in multi-disciplinary health research. BMC Med Res Methodol. 2013 Sep 18;13:117. doi: 10.1186/1471-2288-13-117
