# Supplementary material for: Effectiveness of introducing pulse oximetry and clinical decision support algorithms for the management of sick children in primary care in Kenya and Senegal on referral and antibiotic prescription: the TIMCI quasi-experimental pre-post study
Source: eClinicalMedicine. 2025 May 12;83:103196. doi: 10.1016/j.eclinm.2025.103196 (PMC12140026; doi:10.1016/j.eclinm.2025.103196)
Supplement: TIMCI_quasi-experimental pre-post study_SAP_v1.1 [file mmc10.pdf]

# STATISTICAL ANALYSIS PLAN

## *TIMCI: Tool for Integrated Management of Childhood Illness*

## *Cross-country quasi-experimental pre-post study*

| | |
|---|---|
| **Date prepared** | 16.06.2023 |
| **SAP Version** | 1.1 |
| **Protocol Version** | 2.5 |
| **Study registration number (registry)** | NCT05065320 (clinicaltrials.gov) |

Swiss TPH
Swiss Tropical and Public Health Institute
Schweizerisches Tropen- und Public Health-Institut
Institut Tropical et de Santé Publique Suisse
*Associated Institute of the University of Basel*

# ABBREVIATIONS

| | |
|---|---|
| **CI** | Confidence interval |
| **CDSA** | Clinical Decision Support Algorithm |
| **CG** | Caregiver |
| **HC** | Health Center |
| **HCP** | Health Care Provider |
| **ICC** | Intracluster Correlation Coefficient |
| **ID** | Identification |
| **IQR** | Interquartile Range |
| **IMCI** | Integrated Management of Childhood Illness |
| **LMIC** | Low – and middle – income countries |
| **PHC** | Primary Healthcare Centre |
| **RA** | Research Assistant |
| **SAP** | Statistical Analysis Plan |
| **SD** | Standard Deviation |
| **SPA** | Service Provision Assessment |
| **Swiss TPH** | Swiss Tropical and Public Health Institute |
| **TIMCI** | Tool for Integrated Management of Childhood Illness |
| **UCAD** | Université Cheikh Anta Diop de Dakar |
| **UoN** | University of Nairobi |
| **WHO** | World Health Organization |

# TABLE OF CONTENTS

**TABLE OF CONTENTS** **3**
**1 Administrative Information** **5**
1.1 Document Scope 5
1.2 Document History 5
1.3 Roles and Responsibilities 6
1.4 Signatures 6
**2 Introduction** **7**
2.1 Background and Rationale 7
2.2 Objectives 7
**3 Study methods** **8**
3.1 Study Design 8
3.1.1 Overview 8
3.1.2 Study procedures 8
3.1.3 Study intervention 9
3.1.4 Outcomes 9
3.2 Randomisation 10
3.3 Blinding 10
3.4 Sample Size 10
3.5 Framework 10
3.6 Statistical interim Analysis and stopping Guidance 11
3.7 Timing for Analysis 11
3.8 Timing for Baseline and Outcome Assessments 11
**4 Statistical Principles** **11**
4.1 Confidence Intervals and p-Values 11
4.2 Intervention adherence and Protocol Deviations 11
4.3 Analysis Populations 12
**5 Study Population** **12**
5.1 Screening 12
5.2 Eligibility 12
5.3 Recruitment 13
5.4 Withdrawal/Follow-up 13
5.5 Baseline Participant Characteristics 13
**6 Analysis** **14**
6.1 Outcomes Definitions 14
6.1.1 General definitions 14
6.1.2 Health, hospitalisation and referral outcomes 15
6.1.3 Antibiotic-related outcomes 17
6.1.4 Antimalarial-related outcomes 17
6.1.5 Hypoxaemia-related outcomes – in post-intervention only 18

6.1.6 Follow-up outcomes 19
6.2 Analysis Methods 20
6.2.1 Analytical Methods 20
6.2.2 Adjustment for Covariates 21
6.2.3 Test of Assumptions, Actions to be taken 21
6.2.4 Pre-planned Sensitivity Analyses 21
6.2.5 Pre-specified Subgroups Analysis 22
6.3 Missing Data 22
6.4 Additional analyses 22
6.5 Harms 23
6.6 Statistical Software 23
**7 References 23**
7.1 SOPs, study-specific Documents 23
7.2 External references 23
**8 APPENDIX A 24**
Figure 1) Recommended standardised age disaggregation groups for data analysis by life stage [3]. 24

# 1 ADMINISTRATIVE INFORMATION

## 1.1 Document Scope

This document provides a detailed description of the methodologies that will be followed, as closely as possible, when analysing and reporting results from TIMCI quasi-experimental pre-post study. The planned analysis detailed in this document is in compliance with that briefly specified in the TIMCI protocol, except if otherwise detailed.

The purpose of this Statistical Analysis Plan is to:

- Ensure that the analysis is appropriate for the aims of the study, reflects good statistical practice in general, and minimises bias.
- Ensure that the analyses performed are consistent with the conditions of the protocol.
- Explain in detail how the data will be handled, covariates derived and analysed to ensure transparency and reproducibility, including enabling others to perform the actual analysis in the event of sickness or other absence.
- Protect the project by helping it keep to timelines and within scope.

Additional exploratory or auxiliary analyses of data not specified in the protocol or this document are permitted but fall outside the scope of this Statistical Analysis Plan. Where analyses are presented which are not included in the SAP, these will be clearly indicated as such along with a justification as to their inclusion.

The analysis strategy will be made available if required by journals or referees when the main papers are submitted for publication. Additional analyses suggested by reviewers or editors will, if considered appropriate, be performed in accordance with the analysis plan, but if reported, the source of such post-hoc analyses shall be declared.

This document is written following the guidelines outlined in Gamble et al. [1]

## 1.2 Document History

| Statistical Analysis Plan Version | Protocol Version | Section number(s) changed | Description of changes | Date | By whom |
|---|---|---|---|---|---|
| 1.0 | 2.5 | - | New document | 05/04/2023 | Silvia Cicconi |
| 1.1 | 2.5 | 1.3 | Correction of affiliation | 16/06/2023 | Silvia Cicconi |

## 1.3 Roles and Responsibilities

| Name | Affiliation | Role in the study |
|---|---|---|
| **Ms. Silvia Cicconi** | Swiss TPH | Trial Statistician |
| **Dr. Tracy Glass** | Swiss TPH | Senior Statistician |
| **Dr. Fenella Beynon** | Swiss TPH | Clinical Research Scientist |
| **Dr. Hélène Langet** | Swiss TPH | Data Manager |
| **Prof. Kaspar Wyss** | Swiss TPH | PI |
| **Prof. Valerie D'Acremont** | Swiss TPH | PI |
| **Prof. James Machoki M'Imunya** | UoN | Kenya PI |
| **Mr. Francis Njiri John** | UoN | Kenya Study Coordinator |
| **Mr. Kevin N. G Ngari** | UoN | Kenya Data Manager |
| **Dr. Rose Jepchumba Kosgei** | UoN | Kenya Clinical Epidemiology Lead |
| **Prof. Ousmane Ndiaye** | UCAD | Senegal PI |
| **Prof. Papa Moctar Faye** | UCAD | Senegal Study Coordinator/Clinical Researcher |
| **Dr. Aliou Thiongane** | UCAD | Senegal Clinical Researcher |
| **Dr. Jean Augustin Tine** | UCAD | Senegal Biostatistician |

## 1.4 Signatures

| Name | Responsibility | Signature | Date |
|---|---|---|---|
| Silvia Cicconi | Author | DocuSigned by: 757324FFACB944F... | 16-Jun-2023 \| 14:18:23 CEST |
| Tracy Glass | Statistical reviewer | DocuSigned by: 38DF88AA385F454... | 19-Jun-2023 \| 11:49:31 CEST |
| Fenella Beynon | Clinical reviewer | DocuSigned by: Fenella Beynon CF29BE6036654B1... | 17-Jun-2023 \| 09:01:09 CEST |

| | | | |
|---|---|---|---|
| Kaspar Wyss | Reviewer | DocuSigned by: Kaspar Wyss 2AAD51657B2149B... | 16-Jun-2023 \| 02:41:31 PDT |
| Valerie D'Acremont | Reviewer | DocuSigned by: Valerie D'Acremont EBC5406609F2400... | 16-juin-2023 \| 14:25:02 CEST |
| James Machoki M'Imunya | Reviewer | Mmachoki | 16-Jun-2023 |
| Ousmane Ndiaye | Reviewer | DocuSigned by: Ousmane Ndiaye 4D3F677C263A477... | 16-juin-2023 \| 12:30:13 CEST |

# 2 INTRODUCTION

## 2.1 Background and Rationale

Despite progress in reducing child mortality in the last few decades, 5.2 million children under five years of age died of preventable causes. Strengthening systems to identify and appropriately treat sick children, alongside health prevention and promotion activities, is critical to reduce under-five mortality. The Integrated Management of Childhood Illness (IMCI) guidelines, launched in 1995 and now adopted by over 100 countries, responded to the need to systematise the implementation of evidence-based health interventions for children under five in primary care and the community [2]. Yet despite the intention of IMCI to have high sensitivity for detection of severe disease, non-adherence by health workers and intrinsic problems of guidelines based on clinical signs alone are causes of poor identification and management of sick children. The Tools for the Integrated Management of Childhood Illness (TIMCI) project seeks to address these issues by introducing pulse oximetry and clinical decision support algorithms (CDSAs) to strengthen guideline implementation and accuracy in order to improve quality of care and reduce morbidity & mortality of children under five years of age.

Please refer to the section Background and rationale of the Protocol for more information.

## 2.2 Objectives

The overall goal of the TIMCI project is to reduce morbidity and mortality in sick children attending primary care facilities, while supporting the rational and efficient use of diagnostics and medicines by health care providers (HCP). The implementation research component of the project seeks to generate evidence on the health impact, operational priorities, cost and cost-effectiveness of introducing pulse oximetry embedded into a CDSA, into primary care in

LMICs, for children 0 – 59 months of age, to facilitate national and international decision-making on scale-up.

# 3 STUDY METHODS

## 3.1 Study Design

### 3.1.1 Overview

The TIMCI study is an international project involving diverse health systems and communities in India, Kenya, Senegal and Tanzania. The study setting includes facilities providing primary care services, including outpatient settings within larger health centres in addition to the more traditionally-labelled primary care facilities.

As part of the evaluation in Kenya and Senegal, a quasi-experimental pre-post study will compare clinical care of children attending primary care facilities in a pre-intervention (baseline period) with those attending facilities following introduction of the intervention (intervention period). More details of intervention can be found in Section 3.1.3 and the Intervention section of the Protocol.

The assessment of the health impact of the intervention will be complemented by embedded mixed methods sub-studies to evaluate other key components and gain a deeper understanding of the implementation mechanisms and context. These studies include a modified service provision assessments (SPAs) and qualitative studies. Data from these studies will be triangulated in order to generate in-depth insights into implementation. Please refer to the Study Design section of the Protocols for more details.

### 3.1.2 Study procedures

Research assistants (RAs) will screen and recruit participants in the waiting area prior to consultation (Day 0). Following children's caregiver informed consent, the RA will record participant name, contact details, sociodemographic details and reason for attendance prior to consultation. In case of critical and emergency cases, children will be seen immediately by the HCP. The clinical consultation will be conducted by HCPs, who in the post-intervention phase will have been advised to use the devices.

After consultation, the RA will extract information from clinical records, the caregiver and/or medical records and will document them in the database. This information includes final diagnosis, oxygen saturation and results of laboratory investigations, prescribed medication, referral or follow-up advice and usage of pulse oximeter and/or CDSA.

Follow-up at Day 7 will be conducted by phone by an RA. If phone follow-up fails, three attempts will be made on the subsequent three days to reach the caregiver. Community mechanisms to facilitate follow-up (such as arranging a call with a community health worker) will be used, with consent, for caregivers without access to a phone. A structured questionnaire will be administered to assess the primary and secondary outcomes, including details of any government or private hospital admission. In case of non-recovery, if the child is still at home, the study team will advise the family to return to the PHC clinic for a follow-up consultation. Refer to "Participant timeline" section of the Protocol for additional details.

### 3.1.3 Study intervention

- Pulse oximetry embedded into CDSA
  - Pulse oximeters, with guidance (integrated into CDSA), training and mentorship
  - Tablet-based CDSAs, with guidance, training and mentorship
  - Refresher training on latest national / IMCI guidelines integrated with pulse oximetry and CDSA training

### 3.1.4 Outcomes

This section provides an overview of the outcomes as listed in the protocol.

**Primary outcomes**

- Proportion of children referred by a primary healthcare provider to a higher level of care at Day 0 consultation
- Proportion of children prescribed an antibiotic at Day 0

**Secondary outcomes**

- Proportion of children with a severe complication by Day 7
- Proportion of children admitted to hospital within 24 hours of the Day 0 consultation and as a result of a referral (this is a proxy for 'appropriate referral' of children)
- Proportion of children cured at Day 7 follow-up
- Proportion of children who completed referral as reported at Day 7 follow-up

**Other outcomes of interest – hypoxaemia (in post-intervention only)**

- Proportion of children with severe ($SpO_2$ <90%), moderate ($SpO_2$ 90 – 91%) and mild ($SpO_2$ 92 – 93%) hypoxaemia, adjusted for sites at high altitude
- Proportion of children with hypoxaemia (according to differing cut-offs) with severe complication
- Proportion of children with severe hypoxaemia not meeting any other clinical criteria for severe disease
- Proportion of children referred with hypoxaemia who receive oxygen at hospital

**Other outcomes of interest – referral and follow-up**

- Proportion of children with non-severe disease referred to a higher level of care on Day 0
- Average length of stay (in days) of children admitted to hospital
- Proportion of children attending scheduled follow-up at the same facility by Day 7
- Proportion of children presenting for unscheduled follow-up to any health facility by Day 7

**Other outcomes of interest – antimicrobial prescription**

- Proportion of children prescribed a diagnosis-appropriate antibiotic
- Proportion of febrile children tested for malaria at Day 0
- Proportion of malaria positive children prescribed an antimalarial
- Proportion of malaria negative children prescribed an antimalarial
- Proportion of untested children prescribed an antimalarial

**Other factors for exploratory analyses**

- Additional socio-demographic characteristics, urban / rural location, distance from health facility, key household indicators such as maternal age and education
- Main complaint / symptom categories (including cough / difficulty breathing, fever, diarrhoea and other) and time since onset of symptoms
- Alternative hypoxaemia cut-offs
- Health care provider severity classification
- Facility and health system factors – type of facility, health care provider qualification and previous training

These exploratory analyses follow outside the scope of this statistical analysis plan and they will not detailed further here but in a separate document.

## 3.2 Randomisation

This is not a randomised study so no randomisation was performed.

## 3.3 Blinding

This is an open-label study.

## 3.4 Sample Size

The sample size for the quasi-experimental pre-post study is calculated separately for each country, based on comparison between pre- and post-intervention periods. The following assumptions are used:

- power 80%
- alpha level of 0.05
- pre-intervention referral to a higher level of care 3%, with a minimum detectable difference of 50%
- pre-intervention antibiotic prescription of 60%
- intra-cluster correlation coefficient (ICC) of 0.005 for referral and 0.05 for antimicrobial prescription
- Kenya – estimated average cluster size of 230 children / month (assuming a cluster ratio of health centres : dispensaries 1:2, with 100 / month in dispensaries and 500 / month in health centres
- Senegal – estimated cluster size of 170 children per month

For the referral primary outcome, we therefore initially estimated requiring the following sample size:

- Kenya – 17 facilities, recruiting an average of 690 children per quarter per facility, equating to 11,730 children per quarter
- Senegal – 18 facilities, recruiting an average of 510 children per quarter per facility, equating to 9,180 children per quarter

For the antibiotic prescription primary outcome, this would enable a minimum detectable reduction of 18% in Kenya and Senegal.

Following lower than anticipated recruitment in the pre-intervention period, sample size calculation was revised. As a result, two additional facilities were added per country and the pre-intervention period was extended from an initially anticipated 3 months to a 6 – 8 month period. A decision was also taken to target a post-intervention sample size of at least the same as the pre-intervention sample size, as well as a minimum duration of 9 months post-intervention to allow for stabilisation over time and some seasonal overlap of pre- and post-intervention periods.

## 3.5 Framework

The quasi-experimental pre-post study is based on the hypothesis that the intervention will result in a reduction in adverse health outcomes through better treatment and referral

decisions, and an improvement in quality of care through the rational use of essential medicines and improvements in the health worker and caregiver experience.

## 3.6 Statistical interim Analysis and stopping Guidance

No interim analysis is planned for this study.

## 3.7 Timing for Analysis

The final analysis will take place once all children have received a minimum of 7 days of follow-up. A pooled cross-country analysis will be conducted as well as individual country analyses.

## 3.8 Timing for Baseline and Outcome Assessments

Baseline is defined as the date of enrolment (Day 0) and time will be measured from this point.

Follow-up will occur 7 days after Day 0. As defined in the protocol, a window of + 3 days applies to Day 7 follow-up. Refer to “Participants timeline” section of the protocol for more details.

The components (i.e. referral and antibiotic prescription) required for the analysis of the primary outcomes will be assessed at Day 0.

# 4 STATISTICAL PRINCIPLES

## 4.1 Confidence Intervals and p-Values

Results estimates of primary and secondary outcomes will be presented along with the associated two-sided 95% confidence intervals.

## 4.2 Intervention adherence and Protocol Deviations

While in Kenya pulse oximeter was indicated for all children, in Senegal, pulse oximeter was indicated for all young infants and for older children with cough/difficult breathing or red/yellow IMCI classification. Cough/difficulty breathing and red/yellow IMCI classification are defined two ways, based on signs and symptoms as reported by the caregiver pre-consultation or on diagnosis made by the healthcare provider during the consultation. These definitions are detailed in a separate document and both will be used, separately, to assess intervention adherence.

Adherence will be evaluated on the basis of pulse oximeter and CDSA use when indicated.

- Pulse oximetry adherence will be evaluated primarily using information on SpO2 values from clinical records. Pulse oximetry will be considered used if the RA has found any SpO2 recording (value readable or unreadable). Pulse oximetry will be considered not used if the RA did not find any recorded SpO2. Adherence will be additionally evaluated based on pulse oximetry use as reported by the caregivers.

- CDSA adherence will be evaluated by comparing the number of children enrolled and the number of children recorded into the CDSA database. These data will be used to explore further and in more details adherence to the CDSA, but outside the scope of this SAP and it will be detailed in a separate document.

Adherence will be reported overall and weekly by facility.

The following information will be summarised by arm to evaluate deviations to the study protocol:

- Number and percentage of caregivers for which no Day 7 follow-up was attempted.

More general deviations will be recorded by the study team in a study deviation log and categorised into minor and major.

## 4.3 Analysis Populations

All analyses will follow the intention to treat (ITT) principle, irrespective of any protocol violations.

No safety set will be defined but safety information will be reported overall and by use of intervention as defined in section 4.2.

# 5 STUDY POPULATION

## 5.1 Screening

No specific screening data will be captured, aside from those required to assess children's eligibility for the study (see section 5.2). The number of potential participants screened for the study will be presented in a flowchart for pre and post intervention period.

## 5.2 Eligibility

The following are inclusion and exclusion criteria that determine facility and child eligibility:

Facility inclusion:

- Consenting government-designated healthcare facilities within the selected geographical areas of each country
- Providing curative primary care services for children 0 – 59 months of age
- Oxygen available or referral mechanism in place with oxygen available at a higher level facility
- Electricity available (from any source with continuous or intermittent supply)

Facility exclusion:

- Attending to fewer than 20 sick children per month (based on the prior 12 month average)
- Already using pulse oximetry as a routine part of outpatient-based consultations of children 0 – 59 months
- Selected to be part of a major child health programmatic or research intervention during the study period likely to significantly affect the primary outcome
- Inaccessible to the study team (e.g. due to weather conditions or security issues) for significant parts of the year.

Individual child inclusion:

- Children 0 – 59 months for whom caregivers provide consent
- Consulting for an illness, or reported to be unwell when attending for a routine visit (e.g. vaccination, growth or chronic disease monitoring)

Individual child exclusion:

- Children in the immediate post-natal period or first day of life
- Attending for a consultation related to trauma only (including new and follow-up presentations for burns, injuries, wounds)
- Admitted within an inpatient part of the facility (including neonates delivered at the facility admitted with their mother)
- Enrolled in the study within the preceding 28 days at any study facility

## 5.3 Recruitment

The number of participants enrolled will be presented in a flowchart for pre and post intervention, together with the number of potential participants not recruited due to the child's eligibility failure, caregiver's refusal to consent or caregiver being underage. Reasons for eligibility failure will be summarised.

Further enrolment figures will be presented by facility type, location and district and a graph will be produced showing monthly and cumulative recruitment.

Additionally, the percentage of eligible children (no. eligible/no. screened) and the percentage of recruited children (no. recruited/no. eligible) will be provided, overall and pre and post intervention.

## 5.4 Withdrawal/Follow-up

The number of children for which follow-up at Day 7 was conducted will be presented in a flowchart for pre and post intervention. Reasons for withdrawals will be provided if available.

## 5.5 Baseline Participant Characteristics

Socio-demographic data of children enrolled and their caregivers will be summarised. Additional characteristics of interest, such as danger signs and symptoms as reported by the caregiver, will be presented as part of baseline summaries. Baseline data will be presented separately pre and post intervention using median and associated interquartile range (IQR) if continuous and as counts and percentages if categorical. Summary statistics will be of non-missing values, with the number (%) of missing values given if data are not complete. Assessment will be made for baseline changes between two time periods by visual inspection only and no formal testing will be performed.

For the baseline summaries, age of children will be reported as a continuous variable in months but also disaggregated in age groups. In the database, the age is estimated based on recommended standardised age disaggregation groups [2] with a different level of accuracy depending on the information provided by the caregiver. Based on age estimates, children are further categorised into young infants (<60 days) and children 60days-59 months. More details are provided in Appendix A.

# 6 ANALYSIS

Analyses will be performed and reported overall and by country.

Outcomes will be presented separately for young infants (<60 days) and older children (60 days-59 months), the malaria-related outcomes will be presented only on older children.

## 6.1 Outcomes Definitions

This section presents general definitions, applicable to multiple outcomes, and a detailed definition of each primary and secondary outcome.

Outcomes definitions are grouped by topic rather than primary and secondary outcomes.

### 6.1.1 General definitions

**Day 7 assessment**

Day 7 assessment refers to information reported by the caregiver after Day 0 and recorded in Day 7 form. Data collected as part of unscheduled visits are also integrated in these forms. If multiple successful follow-up assessments for the same child are recorded within the Day7 database, the most recent follow-up assessment will be used. To limit potential recall bias, assessments conducted far beyond the expected follow-up time will be dropped, i.e. 30 days from Day 0.

**Death**

A child will be considered deceased if, from Day 7 assessment, his/her status at the time of follow-up is “deceased”. In case of implausible death date (i.e. prior to enrolment date), death will be assumed to be within 7 days of Day0.

**Hospitalisation**

A child will be considered hospitalised if, from Day 7 assessment, he/she went to a hospital, was admitted overnight or if his/her status at the time of follow-up is “hospitalised”. In case of implausible hospitalisation date (i.e. prior to enrolment date), hospitalisation will be assumed to be within 7 days but after 24hrs of Day0.

**Hospitalisation within 24hrs of Day0**

For the purpose of primary analysis, a child will be considered admitted to hospital within 24hrs of Day0 consultation if the date of hospitalisation is the same as Day0 date or is one day after Day0 date.

**Referral**

A child will be considered to be referred (either to a hospital or to an inpatient part of a larger primary healthcare facility) if, at Day0, the research assistant confirms that an urgent referral advice was recorded for the child. If urgency is unknown , referral will be assumed to be urgent.

**Antibiotics**

Antibiotics will be defined in accordance with the WHO antibiotic point prevalence survey (PPS) methodology [4, 5, 6] and therefore only antibacterials for systemic use will be taken into account.

**SpO2 values**

SpO2 should be recorded during the Day0. For the outcomes, the following measures will be taken for spurious and missing SpO2 values:

- If SpO2 value is not available, the child will be assumed normoxaemic
- If 70 ≤ SpO2 < 90%, the child will be assumed (with accuracy) to have severe hypoxaemia
- If 40 ≤ SpO2 < 70,% the child will be assumed (with low accuracy) to have severe hypoxaemia
- If SpO2 < 40%, the value will be considered spurious (e.g. due to incorrect dataentry) and treated as not available, i.e.the child will be assumed normoxaemic.

Additionally, a description of SpO2 values distribution will be provided based on the following cut-offs:

- 95% ≤ SpO2 < 100%
- 94% ≤ SpO2 < 95%
- 92% ≤ SpO2 < 94%
- 90% ≤ SpO2 < 92%
- 70% ≤ SpO2 < 90% (severe hypoxaemia, accuracy)
- 40% ≤ SpO2 < 70% (severe hypoxaemia, but low accuracy)
- Spurious (0% ≤ SpO2 < 40%)
- Missing

**Mild hypoxaemia**

92% ≤ SpO2 < 94% (Kenya)

**Moderate hypoxaemia**

90% ≤ SpO2 < 92% (Kenya)
92% ≤ SpO2 < 95% (Senegal)

**Severe hypoxaemia**

SpO2 < 90% (Kenya)
SpO2 < 92% (Senegal)

### 6.1.2 Health, hospitalisation and referral outcomes

#### 6.1.2.1 Proportion of children with a severe complication by Day 7

Severe complication is defined as death or secondary hospitalisation. Secondary hospitalisation refers to any delayed hospitalisation (occurring at any point greater than 24hrs after the Day 0 consultation) and any hospitalisation occurring without a referral. Information on death and hospitalisation will be taken from Day7 follow-up assessment.

Death will be counted in the numerator if death date is within 7 days of Day0 (7th day included). Hospitalisation will be counted in the numerator if the child was not referred at Day0 or hospital admission date is within 7 days of Day0 (7th day included) but after 24hrs of Day0.

The denominator will be all children recruited. Children for whom no death and hospitalisation data are available or withdrew prematurely from the study will be assumed to be alive and not hospitalised by Day7.

#### 6.1.2.2 Proportion of children admitted to hospital within 24 hours of Day 0 consultation and as a result of a referral

Information on hospitalisation will be taken from Day7 follow-up assessment.

Hospitalisation will be counted in the numerator only if the child received a referral advice (as per definition above) at Day0 and hospital admission date is within the end of the following day. The denominator will be all children recruited.

Additionally, the proportion will be calculated and reported using only children who were referred as denominator.

#### 6.1.2.3 Average length of stay (in days) of children admitted to hospital

Length of stay is defined as hospitalisation duration in days as reported by the caregiver at Day 7. If a child is hospitalised twice, the first hospitalisation will be used and second hospitalisations will be reported separately. The denominator will be all hospitalised children.

#### 6.1.2.4 Proportion of children referred by a primary healthcare provider to a higher level of care at Day 0 consultation

All children who received a referral advice (as per definition above) at Day0 will be counted in the numerator. The denominator will be all children recruited.

#### 6.1.2.5 Proportion of children who completed referral as reported at Day 7 follow-up

A referral will be considered completed if a child attended a hospital, whether he/she was admitted or not. Children will be counted in the numerator if they received a referral advice (as per definition above) and their caregiver reported to have sought care in a hospital and the follow-up date is within 7 days of Day 0, considering a + 3 days window (10$^{th}$ day included). The denominator will be all children recruited.
Additionally, the proportion will be calculated and reported using only children who were referred as denominator.

Children for whom no Day7 data are available or withdrew prematurely from the study will be assumed not attending a hospital by Day7.

#### 6.1.2.6 Proportion of children with non-severe disease referred to a higher level of care on Day 0

Children with non-severe disease who received a referral advice (as per definition above) at Day0 will be counted in the numerator. The denominator will be all children recruited. Non-severe disease will be defined in a separate document.

#### 6.1.2.7 Proportion of children cured at Day 7 follow-up

A child is defined as cured if he/she recovers from illness. A child will be counted in the numerator as cured if his/her status at the time of Day 7 follow-up is at home "well, completely recovered" and the follow-up date is within 7 days of Day 0, considering a + 3 days window (10$^{th}$ day included). The denominator will be all children recruited.

Children for whom no Day7 data are available or withdrew prematurely from the study will be assumed cured by Day7.

### 6.1.3 Antibiotic-related outcomes

#### 6.1.3.1 Proportion of children prescribed an antibiotic at Day 0

Information on antibiotics prescription are recorded by the RA on Day 0, after the clinical consultation. The data collection tool is structured in a way to facilitate the reporting of antibiotics, starting with single-drug questions for the most common/important ones to drop-down lists for other antibiotics/treatments and free text. All options will contribute to determine whether an antibiotic prescription was given. A child will be considered prescribed with an antibiotic if:

- “Yes” was selected for at least one single-drug question
- “No” was selected for all single-drug questions but one or more antibiotics were selected from the antibiotics/treatments drop-down list
- “No” was selected for all single-drug questions, antibiotics were not selected from the other antibiotics/treatments drop-down list but antibiotics were listed in the free text field (conditional to feasibility of assessing free text)

All children recruited will be counted in the denominator.

#### 6.1.3.2 Proportion of children prescribed a diagnosis-appropriate antibiotic

Appropriateness of antibiotic prescription in relation to diagnosis will be evaluated as:

- Proportion of children who received a diagnosis for which a systemic antibiotic was indicated and a systemic antibiotic was prescribed
- Proportion of children who received a diagnosis for which a systemic antibiotic was not indicated and systemic antibiotic was prescribed
- Proportion of children who received first-line (or second-line) antibiotics according to recommendations for IMCI diagnoses  for which specific antibiotic(s)are indicated

Only children with a diagnosis for which a systemic antibiotic was indicated will be counted in the denominator for the first proportion, only children with a diagnosis for which a systemic antibiotic was not indicated will be counted in the denominator of the second proportion, and only children who received an IMCI diagnosis requiring specific antibiotics will be counted in the denominator of the third proportion.

Diagnoses will be classified according to whether systemic antibiotics are indicated, based on IMCI and other relevant national guidelines as used for the CDSA, using the structured data entry and free text if feasible. Diagnosis classification will be defined in a separate document.

### 6.1.4 Antimalarial-related outcomes

Although the following outcomes need to share the same definition for a cross-country analysis, it is acknowledge that malaria testing strategies are different across countries. In Senegal all febrile children (≥60 days) should be tested for malaria. In Kenya febrile children should be tested only in high risk areas (or seasons), or in low risk areas if there is history of travel to a high risk area or when no other obvious cause of fever is detected. This difference will need be taken into account when reporting these outcomes. Untested febrile children will need to be described more in detail for Kenya.

#### 6.1.4.1 Proportion of febrile children tested for malaria at Day 0

A child will be considered to be febrile if, at Day 0, history of fever was reported by the caregiver before the consultation or temperature was recorded to be above or equal 37.5 C° or fever was recorded elsewhere in the registry (e.g. diagnosis).

A child will be considered tested for malaria if a malaria test was ordered during the consultation and a test result is available (i.e. positive, negative, invalid/indeterminate, reported but handwriting not readable).

Only febrile children will be counted in the denominator.

#### 6.1.4.2 Proportion of malaria positive children prescribed an antimalarial

A malaria test will be considered positive if a malaria test was ordered during the consultation and was recorded as positive. If the test result is invalid/indeterminate, reported but handwriting not readable or not sure/not reported, the test will not be considered positive.

The child will be considered prescribed with an antimalarial if:

- One or more antimalarial drugs were selected from the antimalarial/treatment drop-down list
- Antimalarial drugs were not selected from the antimalarial/treatment drop-down list but antimalarial drugs were listed in the free text field (conditional to feasibility of assessing free text)

Only children with a positive malaria test result will be counted in the denominator.

#### 6.1.4.3 Proportion of malaria negative children prescribed an antimalarial

A malaria test will be considered negative if a malaria test was ordered during the consultation and the result was negative. If the test result is invalid/indeterminate, or handwriting not readable or not sure/not reported, the test will not be considered negative.

Antimalarial drug prescription will be assessed as described in the point above.

Only children with a negative malaria test result will be counted in the denominator.

#### 6.1.4.4 Proportion of untested children prescribed an antimalarial

A child will considered untested for malaria if, a malaria test was not ordered during the consultation or a malaria test was ordered during the consultation but a test result is not available.

Antimalarial drug prescription will be assessed as described in the point above.

Only children untested for malaria will be counted in the denominator.

### 6.1.5 Hypoxaemia-related outcomes – in post-intervention only

#### 6.1.5.1 Proportion of children with severe, moderate and mild hypoxaemia, adjusted for sites at high altitude

Hypoxaemic children will be summarised disaggregated by groups based on the following SpO2 values ranges: SpO2 < 90%, 90% ≤ SpO2 < 92% and 92% ≤ SpO2 < 94%. All children recruited will be in the denominator.

All facilities in Senegal are in low altitude area and therefore no adjustment is required. Only few facilities from one district in Kenya were above 2000m but cut-off for referral was not adjusted and therefore no adjustment will be applied.

#### 6.1.5.2 Proportion of children with hypoxaemia (according to differing cut-offs) with severe complication

Children with severe complication will be summarized disaggregated by groups based on the following SpO2 values ranges: SpO2 < 90%, 90% ≤ SpO2 < 92% and 92% ≤ SpO2 < 94%, spurious values and missing values. Each SpO2 group will be the denominator of each proportion.

#### 6.1.5.3 Proportion of children with severe hypoxaemia not meeting any other clinical criteria for severe disease

Children with severe hypoxaemia (according to country's specific cut-off) not meeting any other clinical criteria for severe disease will be counted in the numerator. All children recruited will be in the denominator.

Severe disease will be defined in a separate document.

#### 6.1.5.4 Proportion of children referred with hypoxaemia who receive oxygen at hospital

Children with hypoxaemia (according to country's specific cut-off) who received a referral advice at Day0 and oxygen at hospital (according to hospital records) will be counted in the numerator. All children recruited will be in the denominator.

Furthermore, children with hypoxaemia who were referred and children with hypoxaemia who were referred and admitted to hospital will be summarised using all children recruited as denominator.

Additionally, the proportions will be calculated and reported using only children with hypoxaemia as denominator.

### 6.1.6 Follow-up outcomes

#### 6.1.6.1 Proportion of children attending scheduled follow-up at the same facility by Day 7

Attendance of scheduled follow-up at the same facility will be evaluated from:

- Scheduled and unscheduled visits form
  A child will be counted in the numerator if:
    - a follow-up visit advice is recorded at Day 0 and
    - a follow-up visit for the child is found in the scheduled/unscheduled visits form and
    - the follow-up visit is within 7 days of Day 0 (7th day included) and conducted at the enrollment facility

  All children recruited will be in the denominator.

  Additionally, the proportion will be calculated and reported using only children for whom a follow-up visit advice is recorded at Day0 as denominator.

  Children for whom a scheduled follow-up visit is not found in the scheduled/unscheduled visit form will be assumed to not have attended scheduled follow-up at the same facility by Day 7.

- Day 7 follow-up form
  A child will be counted in the numerator if:
    - a follow-up visit advice is recorded at Day 0 and
    - the caregiver reports visiting a government / public facility (non hospital) between child's enrollment and Day 7 follow-up visit date and
    - Day 7 follow-up date is within 7 days of Day 0, considering a + 3 days window ($10^{th}$ day included).

  All children recruited will be in the denominator.

  Additionally, the proportion will be calculated and reported using only children for whom a follow-up visit advice is recorded at Day0 as denominator.

Children for whom no Day7 data are available or withdrew prematurely from the study will be assumed to not have attended scheduled follow-up at the same facility / government or public facility by Day 7.

#### 6.1.6.2 Proportion of children presenting for unscheduled follow-up to any health facility by Day 7

Attendance of unscheduled follow-up at any health facility will be evaluated from:

- Scheduled and unscheduled visits form
  A child will be counted in the numerator if:
  - a follow-up visit advice is not recorded at Day 0 and
  - a follow-up visit for the child is found in the scheduled/unscheduled visits form and
  - the follow-up visit is within 7 days of Day 0 (7th day included)

  All children recruited will be in the denominator.

  Additionally, the proportion will be calculated and reported using only children for whom a follow-up visit advice is not recorded at Day0 as denominator.

- Day 7 follow-up form
  A child will be counted in the numerator if:
  - a follow-up visit advice is not recorded at Day 0 and
  - the caregiver reports visiting a health facility (any, non-hospital) between child's enrollment and Day 7 follow-up visit date and
  - Day 7 follow-up date is within 7 days of Day 0, considering a + 3 days window ($10^{th}$ day included).

  All children recruited will be in the denominator.

  Additionally, the proportion will be calculated and reported using only children for whom a follow-up visit advice is not recorded at Day0 as denominator.

  Children for whom no Day7 data are available or withdrew prematurely from the study will be assumed to not have attended any health facility by Day 7.

## 6.2 Analysis Methods

### 6.2.1 Analytical Methods

For the primary analysis, pre-intervention and post-intervention periods will be defined as follow:

*Pre-intervention period*: from the date of data collection start to the date healthcare providers left the facilities for training on the intervention

*Post-intervention period*: from the date healthcare providers returned to the facilities after training and the devices was delivered to the facilities to the end of data collection

As training was staggered for the facilities, the definitions above will be applied at the facility level.

Outcomes will be described for each time period using summary statistics. In particular, counts and percentages will be used for binary variables and reported with one decimal place; mean and standard deviation (SD) or median (IQR) for continuous variables depending on their

distribution. Mean and median will be displayed with one decimal place and SD and IQR will be presented with two decimal places.

The two primary outcomes will be assessed using a random effects logistic regression model with the cluster included as a random effect. If convergence issues arise due to sparse data, clusters may be dropped or combined to higher grouping level (e.g. district).

Results will be reported with odds ratios, risk differences and their associated 95% confidence intervals (CI).

Secondary outcomes and other binary outcomes of interest will be evaluated in the same way.

Other continuous outcomes of interest will be assessed using random effects linear regression model with the cluster included as a random effect. Results will be reported in terms of adjusted mean differences with associated 95% CIs.

Modelling of secondary outcomes will be performed if numbers allow.

Further exploratory analysis, including interrupted time series analysis to assess change over time and data linked between the quasi-experimental pre-post study and the SPA and time-flow studies. These fall outside the scope of this SAP and will be detailed in a separate document.

### 6.2.2 Adjustment for Covariates

All models will be adjusted for the following potential confounders: age, sex, travel time to facility, illness duration, clinical presentations, previous care and previous treatment. Further sensitivity analysis may be performed adjusting for additional baseline variables.

### 6.2.3 Test of Assumptions, Actions to be taken

Continuous variables will be inspected using histograms: 1) to assess for outliers which may be queried for accuracy, and 2) to assess whether appropriate transformations are required for analyses.

As a multilevel logistic regression model is estimated using quadrature (rather likelihood methods), a quadrature check to test for adequate model fit will be performed. In case of unreliable model fit, generalized estimating equations will be used to fit the model which will provide population-averaged odds ratios and 95% confidence intervals.

### 6.2.4 Pre-planned Sensitivity Analyses

Sensitivity analyses on the primary outcomes are planned and will be conducted:

- On first encounters, which will include only the first enrolment episode of each child during the study period (as some children may attend the facility on more than one occasion). Separate presentations are identifiable from Day0 form as the caregiver is asked whether the child attended previously and within 28 days. This is the minimum number of days required for the disease episode to be considered a new one and not a repeat visit.
- Looking at different referral definitions[1]:
    - A child will be considered to be referred if, at Day0, the caregiver confirms that the child was urgently referred *or* the research assistant confirms that an urgent referral advice was recorded for the child. If urgency is unknown from both sources, referral will be assumed to be urgent.

[1] Urgency, as reported by the caregiver, was introduced only in the post-intervention period in Kenya. For the pre-intervention period, urgent referrals will be defined based on the registry alone, as described for the primary analysis.

- A child will be considered to be referred if, at Day0, caregiver confirms that the child was urgently referred *and* the research assistant confirms that an urgent referral advice was recorded for the child.

Additionally, a set of sensitivity analyses shall be carried out based on different definitions of pre and post-intervention periods:

- Pre-intervention and post-intervention defined based on periods of the year that match in the pre and post intervention.
- Pre-intervention defined as primary analysis and post-intervention from country-specific relevant dates (i.e. formal initiation after the strike in Senegal and intensive supervision in Kenya) to the end of data collection.

Sensitivity analyses on the secondary outcomes related to hospitalisation will be conducted looking at different hospitalisation appropriateness (based on different definitions of primary and secondary hospitalisations). Same day (Day0), 3 days and 7 days from Day0 will be used as cut-offs for different primary and secondary hospitalisation definitions.

### 6.2.5 Pre-specified Subgroups Analysis

Subgroup analysis of the primary outcomes are planned to assess effect modifiers, specifically:

- Age (under 2 months, 2 – 12 months, 13 – 59 months) – as recorded at Day0
- Sex (male, female) – as recorded at Day0
- Presentation with cough or difficulty breathing (yes, no) – as recorded at Day0
- Location (urban/rural)

Effect modification will be assessed by incorporating an interaction between the time period and the potential effect modifier in the model, acknowledging that power will be low. Stratified model results will only be presented if the interaction term is significant, defined as p-value<0.2.

## 6.3 Missing Data

Missing values will be primarily due to children lost to follow-up and not reached at Day 7.

Primary outcomes are based on information collected at Day0 through a structured data collection process that prevents missing values due to compulsory data entry. Therefore, missing data on the primary outcomes are not anticipated.

Analysis on secondary outcomes that rely on Day 7 follow-up data will be performed assuming the best case scenario (alive and not hospitalised) for children lost to follow-up or withdrawn.

Additionally, missing data will be described by pre and post intervention period and by relevant indicators, such as referral and severe disease to visually inspect for imbalances.

## 6.4 Additional analyses

Primary outcomes will be further presented by adherence and indication groups (e.g. children for which pulse oximetry was indicated and was used, children for which pulse oximetry was indicated but was not used, etc.).

## 6.5 Harms

Safety of the interventions will be evaluated in terms of deaths and secondary hospitalisations that occurred throughout the course of the study. Deaths, secondary hospitalisations and both combined will be reported as counts and percentages. Summary statistics will be presented separately for each intervention as outlined in Section 4.3.

## 6.6 Statistical Software

Statistical analysis will be performed using one of the standard statistical packages such as R v4.0.3 or higher versions [7]. The software and the version used for performing the analysis will be stated in the final statistical report.

# 7 REFERENCES

## 7.1 SOPs, study-specific Documents

A. TIMCI Data Management Plan.

## 7.2 External references


1. Gamble C, Krishan A, Stocken D, Lewis S, Juszczak E, Doré C, et al. Guidelines for the Content of Statistical Analysis Plans in Clinical Trials. JAMA. 2017 19;318(23):2337–43.

2. Costello, A., Dalglish, S. & on behalf of the Strategic Review Study Team. Towards a Grand Convergence for child survival and health: A strategic review of options for the future building on lessons learnt from IMNCI. (2016).

3. Diaz T, Strong KL, Cao B, Guthold R, Moran AC, Moller AB, et al. A call for standardised age-disaggregated health data. The Lancet Healthy Longevity [Internet]. 2021 Jul;2(7):e436–43. Available from: https://linkinghub.elsevier.com/retrieve/pii/S266675682100115X

4. Geneva: World Health Organization; 2018. Licence: CC BY-NC-SA 3.0 IGO.

5. https://www.who.int/publications/i/item/2021-aware-classification

6. Pauwels et al, Hospital antibiotic prescribing patterns in adult patients according to the WHO Access, Watch and Reserve classification (AWaRe): results from a worldwide point prevalence survey in 69 countries, *Journal of Antimicrobial Chemotherapy*, Volume 76, Issue 6, June 2021, Pages 1614–1624.

7. R Core Team (2020). R: A language and environment for statistical computing. R Foundation for Statistical Computing, Vienna, Austria. URL https://www.R-project.org/.

# 8 APPENDIX A

The age of the child is expressed differently for *young infants* (i.e. children up to 59 days) and for children between 60 days and 59 months (sometimes more simply referred to as "*2-59 months*")

- It is grouped in three categories ([1-6 days][2], [7-27 days], [28-59 days]) for young infants;
- It is expressed in months for children from 60 days to 59 months.

*Figure 1) Recommended standardised age disaggregation groups for data analysis by life stage [3].*

| | Recommended age grouping | Disease burden and health risk* | Examples of key prevention and health promotion interventions | Living conditions and societal factors |
|---|---|---|---|---|
| Early neonates | 0–6 days | A third of all neonatal deaths occur on the day of birth, and nearly 75% occur within the first week of life | Immediate breastfeeding; vaccination (eg, for BCG and hepatitis B) and screening for genetic, endocrine, and metabolic disorders at birth or within the first 24 h of life | Early neonates might be in facility care immediately after birth, especially if premature and low-weight or ill, but are most commonly cared for at home by family |
| Late neonates | 7–27 days | The first 27 days of life (neonatal or newborn period) are the most crucial for survival; neonates accounted for 2·5 million deaths (47% of all deaths under the age of 5 years) in 2019;[31] causes of death in this age group differ from those in early neonatal and post-neonatal infants and a large proportion is due to congenital anomalies | Ensure neonates have received vaccines at birth, check weight, assess for birth defects, and promote the continuation of exclusive breastfeeding | Commonly cared for at home by family or caretakers |
| Post-neonatal infants | 28–364 days | The first year of life after the neonatal phase is the second riskiest period for child survival; about 24% of all deaths under the age of 5, in 2019, occurred in this age group,[31] with pneumonia, diarrhoea, and malaria as leading causes of death | Completion of common vaccines schedule (diphtheria, tetanus, pertussis, *Haemophilus influenzae* type b, hepatitis B, poliovirus, pneumococcal, rotavirus, measles-rubella, and two doses of seasonal influenza);[32] continuation of breastfeeding with weaning and introduction of complementary foods when appropriate; long-lasting insecticidal nets and intermittent preventive treatment for infants in malaria-endemic countries | Commonly cared for at home by family, but can begin to go to day care |
| Young children | 1–4 years | This group has the greatest reductions in mortality of all age groups to date, but mortality in this group remains fairly high in many countries; environmental exposures during the first 3 years of life can affect a child's developmental trajectory and lead to an increased risk of physical and psychological illness, affecting health and wellbeing in later life[27–29] | Diphtheria, tetanus, pertussis, and measles-rubella boosters and seasonal influenza vaccine;[32] completion of weaning; long-lasting insecticidal nets and seasonal malaria chemoprevention in malaria endemic countries; early childhood programmes; environmental and policy interventions (eg, clean water and sanitation, fluoridation, safe playgrounds, and appropriate car seats) | Children can begin to attend preschool or day care and often play with other children |

The age of the child is available in the TIMCI database with different levels of accuracy depending on the information primarily shared by the caregiver. It can be either calculated as number of days between date of consultation and date of birth or estimated by categories.

## Calculated Age

The date of creation of the ODK form is considered to be the date of consultation. The date of birth can be entered with three different levels of accuracy:

- **DD/MM/YYYY**
  If the exact date of birth is known, the age in month is derived from the age in days through a division by 30.44 $age_{\text{month}} = \frac{age_{\text{days}}}{30.44}$
  The age is converted to the corresponding categories for young infants to keep the age granularity necessary while avoiding being able to indirectly recalculate the date of birth from the date of consultation and age in days:

[2] The first day of life (or day of birth) is an exclusion criterion for TIMCI.

$$\begin{cases} 1 \leq if\ age_{\text{days}} < 7 & [1-6\text{d}] \\ 7 \leq if\ age_{\text{days}} < 28 & [7-27\text{d}] \\ 28 \leq if\ age_{\text{days}} < 60 & [28-59\text{d}] \end{cases}$$

- **MM/YYYY and YYYY**
  When the date of birth is not exactly known with a day accuracy, the age can only be known within a range of maximum and minimum ages.
  The following approach is used:
  - Young infants: those children who have their minimal age *(*variable *a3-a3_a_6a* in the Day 0 ODK form*)* in the interval [1-59 days]
  - Children from 60 days to 59 months: those children who have their minimal age *(*variable *a3-a3_a_6a* in the Day 0 ODK form*)* in the interval [60 days - 59 months]

  When only the year of birth is known, it is attempted to reduce the possible age range by clarifying the season of the birth of the child:

  - January - March
  - April - June
  - July - September
  - October – December
  - Unknown (code: 98), the age range remains the one determined by the year of birth and the date of consultation

### Estimated age

If the caregiver is not able to provide any elements of the date of birth, an available alternative is to select the category corresponding to the estimated age of the child.

The available categories that are defined in the data collection tool are as follow:

- Under 1 year
  - Under 1 month
    - Less than 1 day (first day of life) ⇒ children excluded from the study
    - 1-6 days
    - 7-28 days
  - 1 month
  - 2 months
  - 3 months
  - 4 months
  - 5 months
  - 6 months
  - 7 months
  - 8 months
  - 9 months
  - 10 months
  - 11 months
- From 1 to under 2 years
  - 1 year and 0-2 months (12-14 months)
  - 1 year and 3-5 months (15-17 months)
  - 1 year and 6-8 months (18-20 months)

    - 1 year and 9-11 months (21-23 months)
- From 2 to under 3 years
- From 3 to under 4 years
- From 4 to under 5 years
