# Supplementary material for: Effectiveness of introducing pulse oximetry and clinical decision support algorithms for the management of sick children in primary care in Kenya and Senegal on referral and antibiotic prescription: the TIMCI quasi-experimental pre-post study
Source: eClinicalMedicine. 2025 May 12;83:103196. doi: 10.1016/j.eclinm.2025.103196 (PMC12140026; doi:10.1016/j.eclinm.2025.103196)

WHO ERC
Review Summary

Protocol ID: ERC.0003406
Country: Kenya, Myanmar and Senegal
Protocol Title: Tools for the Integrated Management of Childhood Illness: Evaluation of pulse oximetry and clinical decision support algorithms in primary care. Longitudinal observational study, with embedded mixed methods studies, cost and modelled cost-effectiveness in Kenya, Myanmar and Senegal
Version: 2.3 **Dated:** 06/08/2020
WHO Responsible Staff Member: Pablo VEGA
Responsible Unit: UTD/UT/UTC/UTD
Meeting Date: 13/08/2020

Dear Dr. Pablo VEGA,
Please find the review summary of the Protocol "Tools for the Integrated Management of Childhood Illness: Evaluation of pulse oximetry and clinical decision support algorithms in primary care. Pragmatic cluster randomised controlled trial, with embedded mixed methods, cost and cost-effectiveness studies in India and Tanzania", which was submitted to the Secretariat on 22/07/2020. This proposal underwent regular review.

The outcome of the review is provided below. When responding, please submit the following:

1. A cover memorandum that addresses your responses, POINT BY POINT, to each of the queries in sections A and B.
Section C contains Suggestions to improve the proposal but there is no obligation to follow them.

2. An Amended protocol including the responses in bold, highlighted or in track changes. Please ensure that tracking formatting changes is switched off or that all formatting changes have been accepted and that no comments which the team may exchange during the editing are included in the track changes version. The protocol should include all relevant documentation (ICF, study instruments, peer review, etc.) even if already submitted.

Please note that comments in the introductory paragraph are meant for the WHO Responsible Staff Member, though you may decide to share them with the PI.

PLEASE RESPOND TO THIS REVIEW SUMMARY WITHIN A 3 MONTH PERIOD, OR PROVIDE THE ERC SECRETARIAT A VALID JUSTIFICATION FOR THE DELAY.

The ERC considered this an important study which will provide information of significance for how childhood illness is managed. The ERC appreciated the level of detail provided and the complexity of the study due to its scale and embedded mixed methods sub-studies. However, there are some points which require further elaboration. The ERC's main concerns relate to recruitment, confidentiality, the consent documents and risks to health care workers. Please find specific comments below.

A. Amendments (Response and change required)

This section includes queries and comments on your protocol, study instruments or the informed consent form for which the ERC requires your response and where relevant, appropriate amendments to the protocol, study instruments or the informed consent.

1. Protocol

1.1. Please provide an amended proposal specifying the version number and/or date on each page.

In relation to fairness:

1.2. In terms of recruitment of caregivers, it is unclear in the protocol how prospective participants for each part of the study will be selected, when and how they will be approached, who they will be approached by and when consent will take place. This is compounded by the number of sub-studies and consent forms.

1.2.1. Please elaborate how caregivers will be recruited for each aspect of the study including the mixed method studies. Caregivers of patients should be approached first by health care providers, yet under "6.7 Recruitment", when describing recruitment for qualitative studies, it appears participants will be approached by a qualitative researcher. Please clarify.

1.2.2. Please specify the selection criteria and sampling for the observed consultations, the walking interviews, in-depth interviews (IDIs), focused group discussions (FGDs) and creative FGDs as this is unclear in the protocol.

1.2.3. It is noted that the consent documents for the main study inform participants of follow-up calls to check on their child's wellbeing, and that they may be approached by a research assistant about a walking interview after their consultation. However, it is unclear whether, when or how participants are informed they may be contacted by the study team to participate in IDIs, FGDs and creative FGDs. This information should be described in the protocol.

1.2.4. With regards to the recruitment of health care provider (HCP) participants, please specify how HCPs will be identified and approached to participate in the study, including who by.

In addition, the study team's response to the ERC secretariat's memo requesting clarification on this point says "Healthcare providers will be purposively selected from facilities with divergent implementation." (TIMCI WHO ERC Response Letter 6th August 2020 page 3 of 5.) Please explain what is meant by *divergent implementation* in this context.

1.3. On the selection of health care facilities, in the TIMCI WHO ERC Response Letter 6th August 2020 (p.2), the research team wrote that "Individual facility assessments will be conducted prior to project start to confirm appropriate capacity exists for inclusion in the project." Please describe the selection criteria for health facilities, including how capacity is assessed.

1.4. In terms of dissemination, the protocol and consent documents indicate a summary report will be available from healthcare facilities. Please specify how results will be communicated to participating health facilities and study participants as well how policymakers will be informed.

In relation to potential risks and benefits:

1.5. The ERC considered the use of walking interviews intrusive and burdensome on participants who will have sick children with them and also noted privacy concerns in the tracking of the journey. With reference to these points, please provide the rationale for the walking interviews to map care seeking pathways in terms of the study outcomes and specify how participants' privacy and safety will be protected.

1.6. The exit interview is long, particularly since caregivers will be leaving the health centre with sick children. Please address and clarify when and how the interview will take place.

1.7. On duration of data storage, it is noted that shared copies of the de-identified study data will be transferred overseas to Swiss TPH and UoW and kept for 25 years. 25 years seems excessive; please amend or provide strong justification.

1.8. With regards to observation of HCP adherence to ICMI guidelines in their clinical care, the ERC judged there to be risk to participants in terms of potential repercussions following assessments depending on how this is reported. Please provide detail with particular attention to the implications of poor competence and how observed deviations from ICMI guidelines will be handled including whether training/re-training/supervision will be provided. Please advise whether these will be addressed by researchers at the time and whether HCPs must follow directives from researchers.

1.9. If a marked difference is noticed between arms with pulse oximetry and routine care, indicating that routine care is inadequate and missing a lot of severe cases which require referral, please describe how this will be handled. In particular, please elaborate on:

1.9.1. The actions that will be taken,

1.9.2. Any plans to scale up the intervention if a big difference is found, including how governments will be empowered to make this routine practice and whether pulse oximeters will be available at an affordable price in case of scale up.

1.10. It is unclear where IDIs and FGDs will take place. Please address.

1.11. Under 9.3.2 it is stated "No information on individual decisions to accept or decline participation will be shared, neither with supervisors nor with health authorities.". Please advise whether HCPs will have time off to participate.

1.12. In the context of COVID-19, please discuss the implications for the recruitment of participants, the feasibility of fieldwork and what adaptations will be made to ensure that participants who agree to participate are safe (e.g. whether masks and hand sanitizer will be provided to those participating in FGDs).

2. Study Instruments

2.1. For interview and FGD scripts, please include information advising the participant that they can stop the interview and/or decline to answer particular questions without penalty. Please also state that the recording will be destroyed as soon as it has been transcribed.

2.2. In IDI and FGD scripts, it is noted that the pulse oximeters and CDSA are described as “new devices to treat sick children”, but this is misleading as these devices are not treatments, they are an element of diagnostics. Please revise the language used to reflect this and avoid any misunderstanding.

3. Informed Consent Forms

3.1. As per 1.7 above, please amend all informed consent documents to include information on data storage, particularly that data will be transferred overseas and the duration of storage. Please ensure this information is consistent with the protocol, noting the ERC’s concern that retention of data for 25 years is excessive and seems unjustified.

3.2. Consent documents do not explain how participants may withdraw from the study, whether they can withdraw consent for the use of their data and who to contact if they wish to do so. Please ensure this information is clearly stated in all forms.

3.3. To be consistent with the protocol, please amend the consent form for the creative FGDs to state that the participants can refuse to be videoed or photographed but still participate in the activity.

3.4. In the consent document for the walking interview, please explain to the participant the reason for wanting to track the journey by recording data (e.g. GPS) with a device or mobile phone.

3.4.1. Please clearly state that the participant can stop the interview at any time and advise how the data will be handled in case of withdrawal.

3.5. Some inconsistencies were noticed in the consent documents. For example, in the information leaflet and consent form for HCPs (p. 2) under “Study approval”, it says “but will not be able to identify you or your child.” Please review informed consent forms for clarity and consistency.

B. Clarifications (Response required but change may not be required)

This section includes queries on your protocol, study instruments or the informed consent form for which the ERC requires to make changes.

1. Protocol

1.1. Please clarify the role of ministries of health in the study, specifically in terms of implementing results.

C. Suggestions

This section consists of suggestions for alternative scientific or technical approaches or methods for conducting the research but which do not raise critical, ethical issues. These are meant to be helpful to investigators and are presented as suggestions for you to consider incorporating into a revised protocol. No response from you is required for any comment in this section. If, however, you do make changes to the protocol as a result of these suggestions, please submit the revised protocol to the ERC.

1. In the FGDs for HCPs, there do not seem to be any questions about reliability and trustworthiness of the devices, which would be useful information to collect.
2. Under the costing questions, it may be useful to ask which HCPs are routinely expected to use the devices.

Protocol ID: ERC.0003406 **Meeting Date**: 13/08/2020

Based on the above comments, the Committee has the following recommendation(s) for this proposal:

[ ] The proposal is ***Approved as submitted.*** No modifications are required.

[**X**] The proposal is ***Conditionally Approved; requires amendments and/or clarifications.*** Final approval is contingent upon an adequate response by the Principal Investigator, to the satisfaction of the reviewers or the Chair on behalf of the ERC.

[ ] The proposal is ***Not approved; requires additional information and/or rewriting.*** A revised version of the proposal should be re-submitted by the WHO responsible staff member as a new submission to the ERC for re-review by Committee.

[ ] The proposal is ***Rejected***. The proposal is ethically unacceptable, for the reasons stated above. The Principal Investigator may submit a new proposal that takes into consideration the ethical issues raised by the Committee. If you do not agree with the Committee's assessment, please feel free to submit an appeal to the Chair of the ERC, through the Secretariat.

NOTE: Final Approval of the Proposal is contingent upon submission of the following:

[ ] Local ethics approval(s) [ ] Other relevant documents

The ERC would like to receive a copy of the recommendations of the local ethics committee when available.

IMPORTANT

1. *Any changes to the proposal or to the attachments (informed consent/study instruments etc.) should be approved by ERC before being implemented.*
2. *The approval for this proposal is valid for a period of* ***one year only.***
3. *Please resubmit this proposal for a Continuing Review at least 2 months before the next re-approval period.*

Chairperson …………………………………. **Date**. 20.08.2020

Name: Peter Olumese

**FINAL APPROVAL**	**FOR THE SECRETARIAT**
Amendments and Clarifications to the proposal have been reviewed. The protocol (Version: 0.2.4 Date:19.11.2020) and informed consent Forms (Dated: 19.11.2020) submitted on 10.12.2020 are approved by the ERC.<br><br>Please share the protocol and consent documents approved by the ERC with the local ethics committees.<br><br>**Chairperson** ………………………………….<br>**PO/AL**<br>**Name** ………………………………….<br><br>**Date** 21.01.2021……………………….	Amendments and Clarifications to be reviewed:<br>[ ] Electronically by ERC<br>[ ] by Primary reviewers<br>[ ] by Secretariat<br><br>Amendments approved / Clarifications accepted on<br>Local ERC approval(s) obtained on 09.09.2020 (Senegal), 06.10.2020 (Kenya).<br><br>Relevant Documents submitted on<br><br>Comments: pending submission of ethics apporval from Myanmar.

Tools for the Integrated Management of Childhood Illness

Evaluation of pulse oximetry and clinical decision support algorithms in primary care

Longitudinal observational study, with embedded mixed methods studies, cost and modelled cost-effectiveness in Kenya, Myanmar and Senegal

**Version / date**	**Description**	**Prepared by**
Kenya, Myanmar & Senegal specific protocol		
Versions 0.1.1 to 0.1.5	Draft versions (internal only)	Swiss TPH, PATH, UoW, KGMU, UoN, Burnett, UCAD, IHI
Version 0.1.6, 1st Apr 2020	Protocol shared with PATH for independent Scientific Merit Review submission	Swiss TPH
Version 0.1.8 20th May 2020	Protocol shared with PATH in response to independent Scientific Merit Review	Swiss TPH, PATH, UoW, UoN, Burnet, UCAD,
Version 0.1.9, 3rd June 2020	Protocol shared with PATH in response to independent Scientific Merit Review (with track changes)	Swiss TPH
Version 0.2.0, 3rd June 2020	Protocol approved by independent Scientific Merit Review (without track changes)	Swiss TPH
Version 0.2.1, 3rd July 2020	Protocol shared with PATH, updating setting & sample size for Myanmar (with track changes)	Swiss TPH
Version 0.2.2, 3rd July 2020	Protocol approved by independent Scientific Merit Review (without track changes)	Swiss TPH
Version 0.2.3, 6th August 2020	Protocol shared with WHO ERC in response to Secretariat Memorandum	Swiss TPH, PATH

The information contained in this document is confidential. It is intended solely for the Investigators, potential Investigators, consultants, or applicable Independent Ethics Committees and Regulatory Authorities. It is

understood that this information will not be disclosed to others without prior written authorization from the Sponsor, except where required by applicable local laws.

Contents

Abbreviations.......6

1 Summary.......8

2 General information.......13

2.1 Trial registration.......13

2.2 WHO Trial Registration Data Set.......13

2.3 Funding.......13

2.4 Roles & responsibilities.......13

2.4.1 Protocol development: Swiss TPH.......15

2.4.2 Project and research governance.......17

3 Background & rationale.......19

3.1 Rationale for pulse oximetry.......19

3.2 Rationale for clinical decision support algorithm.......21

3.3 The TIMCI project.......22

4 Goal & objectives.......23

5 Study design.......25

5.1 Pilot phase.......26

5.2 Longitudinal observational study.......26

5.3 Service provision assessment.......27

5.4 Facility-based process mapping and time-flow study.......27

5.5 Healthcare providers' acceptability and perceptions of the intervention.......28

5.6 Caregiver perceptions of the intervention and health-seeking behaviour.......28

5.7 Stakeholder perceptions and project data review.......29

5.8 Cost and modelled cost-effectiveness study.......30

6 Participants, interventions, and outcomes.......30

6.1 Study setting.......30

6.1.1 Kenya.......31

6.1.2 Myanmar.......31

6.1.3 Senegal.......32

6.2 Eligibility criteria.......32

6.2.1 Longitudinal observational study and pilot.......32

6.2.2 Other mixed methods studies.......33

6.2.3 Cost and modelled cost-effectiveness study.......34

6.3 Interventions.......34

6.4 Outcomes.......38

6.4.1 Longitudinal observational study 38
6.4.2 Other mixed methods studies 40
6.4.3 Cost and modelled cost-effectiveness study 41
6.5 Participant timeline 41
6.5.1 Longitudinal observational study 41
6.5.2 Other mixed methods studies 46
6.5.3 Cost and modelled cost-effectiveness study 47
6.6 Sample size 47
6.6.1 Longitudinal observational study 47
6.6.2 Mixed methods studies 48
6.6.3 Cost and modelled cost-effectiveness study 50
6.7 Recruitment 50
7 Data collection, management and analysis 52
7.1 Data collection methods 53
7.1.1 Longitudinal observational study 53
7.1.2 Routine data 55
7.1.3 Other mixed methods studies 55
7.1.4 Cost and modelled cost-effectiveness study 57
7.2 Data management 60
7.2.1 Data flow, storage and transfer 60
7.2.2 Data entry, handling and validation 62
7.2.3 Data privacy protection 64
7.2.4 Data quality assurance and control 65
7.3 Statistical methods 66
7.3.1 Longitudinal observational study 66
7.3.2 Routine data 67
7.3.3 Other mixed methods studies 67
7.3.4 Cost and modelled cost-effectiveness study 69
8 Monitoring 70
9 Ethics and dissemination 70
9.1 Research ethics approval 70
9.1.1 Regulatory status of the intervention 71
9.2 Protocol amendments 71
9.3 Consent or assent 72
9.3.1 Longitudinal observational study 72

9.3.2 Other mixed methods studies ... 72
9.3.3 Cost and modelled cost-effectiveness study ... 73
9.4 Confidentiality ... 73
9.4.1 Longitudinal observational study ... 73
9.4.2 Other mixed methods studies ... 73
9.4.3 Linking between longitudinal observational study and mixed methods studies ... 74
9.4.4 Cost and modelled cost-effectiveness study ... 74
9.5 Risks and benefits to participants ... 74
9.6 Access to data ... 75
9.7 Dissemination policy ... 75
10 References ... 77
11 Appendices ... 83
11.1 Informed consent forms appended to the protocol ... 83
Study instruments appended to the protocol ... 83
11.2 83
11.3 Budget and collaboration appended to the protocol ... 83
11.4 TIMCI Preliminary Theory of Change ... 84

Abbreviations

(co)-PI	(co)-Principal Investigator
(e)CRF	(electronic) Case Report Form
BI	Burnet Institute
CDSA	Clinical Decision Support Algorithm
CE	Conformité Européenne
CG	Caregiver
CHC	Community Health Centre
CS	Case Studies
CSO	Civil Society Organization
DALY	Disablity-Adjusted Life Years
DB	Database
DHIS	District Health Information System
DMC	Data Monitoring Committee
DMP	Data Management Plan
EDC	Electronic Data Capture
ePOCT	electronic Point of Care Tool
ERC	Ethical Review Committee
FGD	Focus Group Discussion
GPS	Global Positioning System
HC	Health Centre
HCP	Health Care Provider
HMIS	Health Management Information System
HP	Health Post
HQ	Headquarters
IAG	International Advisory Group
ICC	Intracluster Correlation Coefficient
ICD-10	International Statistical Classification of Diseases and Related Health Problems
ID	Identification
IDI	In-Depth Interview
IHI	Ifakara Health Institute
IMCI	Integrated Management of Childhood Ilnesses
IQR	Interquartile Range
KGMU	King George's Medical University
KII	Key Informant Interview
LMIC	Low- and middle-income countries
M&E	Monitoring and Evaluation
MedAL-C	Medical Algorithm – Creator
MedAL-R	Medical Algorithm – Reader
MedDRA	Medical Dictionary for Regulatory Activities
MEP	Monitoring and Evaluation Plan
MIS	Management Information System
MOH	Ministry Of Health
PHC	Primary Healthcare Center
PII	Personal Identifiable Information
RA	Research Assistant
RHC	Rural Health Center
SAP	Statistical Analysis Plan

SNOMED CT	Systemized Nomenclature of Medicine - Clinical Terms
SOP	Standard Operating Procedure
SPA	Service Provision Assessment
SRA	Stringent Regulatory Authority
Swiss TPH	Swiss Tropical and Public Health Institute
TIMCI	Tools for integrated management of childhood illness
ToC	Theory of Change
UCAD	Université Cheikh Anta Diop de Dakar
UoN	University of Nairobi
US FDA	United States Food and Drug Administration
VPN	Virtual Private Network
WHO	World Health Organisation

1 Summary

Background / rationale

Achieving global targets for child mortality reduction in low- and middle-income countries (LMICs) requires significant improvements in the detection and management of severely ill children. Existing guidelines are inconsistently implemented by healthcare providers and, as they are based on clinical signs, are inadequate for the detection of hypoxaemia, a strong predictor of mortality.

The overall goal of the Tools for the Management of Childhood Illness (TIMCI) project is to reduce morbidity and mortality in sick children attending primary care facilities, while supporting the rational and efficient use of diagnostics and medicines by healthcare providers. The evaluation component of the project seeks to generate evidence on the health impact, operational priorities, cost and modelled cost-effectiveness of introducing pulse oximetry, embedded into a Clinical Decision Support Algorithm (CDSA), at primary care level in LMICs, for children 0 – 59 months of age, to facilitate national and international decision-making on scale-up.

Study design

Health impact will be assessed through a quasi-experimental longitudinal study, comparing clinical care of children in a three-month pre-intervention period (Q1) with four sequential three-month periods over the following year (Q2-Q5). The intervention package will be piloted and refined in parallel with the pre-intervention period of the longitudinal study.

This will be complemented by embedded mixed methods sub-studies to evaluate key components of quality of care and gain a deeper understanding of the implementation processes, mechanisms and context following a realist approach. These studies include modified Service Provision Assessments (SPAs), a facility-based process mapping and time-flow study, qualitative studies with caregivers, healthcare providers and key stakeholders, a survey sent to selected key stakeholders on a quarterly basis, a desk-based project document and monitoring and evaluation (M&E) review, and a cost and modelled cost-effectiveness study.

Study setting

The study setting encompasses facility-based primary care level in Kenya (Kakamega, Kitui and Uasin Gishu), Myanmar (Ayeyarwady and Southern Shan) and Senegal (Thiès). Caregivers often attend different types of facilities to meet their primary care needs; the study setting therefore includes a diverse range of facilities providing primary care services, including outpatient settings within larger health centres in addition to the more traditionally-labelled primary care facilities. This includes Level 2 (dispensaries) and Level 3 (health-centres / sub-county hospitals) in Kenya, sub-centers and rural health centers (RHCs) in Myanmar, and health posts in Senegal.

Key Eligibility Criteria

Consenting government-designated healthcare facilities providing outpatient curative primary care services for children 0 – 59 months of age will be included. Facilities will be excluded if they use pulse oximetry as a routine part of outpatient consultations of children prior to the start of the intervention.

Children 0 – 59 months, for whom caregivers provide consent, will be eligible if attending any study facility with an illness, excluding those admitted as inpatients within the facility, or attending for a consultation related to trauma only.

Caregivers of enrolled children, and healthcare providers at study sites involved in the care of children 0 – 59 months of age, and stakeholders involved in or affected by the intervention, will be eligible for inclusion in the applicable studies.

Interventions

The TIMCI project will introduce pulse oximetry, incorporated into a CDSA, into primary care facilities, supported by targeted training and embedded into monitoring, supportive supervision and maintenance systems.

The pulse oximeters provided in the study will be UNICEF-approved handheld pulse oximeters with paediatric and neonatal probes. The criteria for pulse oximetry use, on which healthcare providers will be trained, are:

- All children under 2 months of age
- Children 2 – 59 months of age presenting with cough / difficulty breathing
- Children 2 – 59 months of age with Integrated Management of Childhood Illness (IMCI) signs of moderate / severe disease (IMCI 'yellow' or 'red' classification)

Healthcare providers will be advised to use a cut-off of SpO2 <90% for referral, unless specific national guidance states otherwise (for example in Senegal, where it is likely that a <92% SpO2 cut-off may be instituted). Different SpO2 cut-offs for referral for sites at high altitude will be finalised with the International Advisory Group (IAG) and Ministries of Health (MoHs).

The above guidance will be incorporated into a tablet-based CDSA, which guides healthcare providers through medical consultations with an algorithm called electronic point-of-care tool (ePoct+). The clinical algorithm will be adapted to reflect national guidelines for the management of children 0 – 59 months of age in primary care (based on IMCI and additional relevant case management guidelines), and will be validated through consultation of international and national experts in each country.

Outcomes

Two primary outcomes will be assessed between the pre-intervention (Q1) and the last intervention period (Q5) of the longitudinal study:

- Proportion of children referred by a primary care healthcare provider to a higher level of care (either to a hospital or to an inpatient part of a larger primary healthcare facility) at Day 0 consultation
- Proportion of children prescribed an antibiotic at Day 0 primary care consultation

Important secondary outcomes include:

- Proportion of children with a severe complication (death or secondary hospitalisation) by Day 7
- Proportion of children admitted to hospital within 24 hours of the Day 0 primary care consultation and as a result of a referral (used as a proxy for 'appropriate referral')
- Proportion of children completing referral to a higher level of care within 24 hours, of all children referred at Day 0 consultation
- Proportion of children cured (defined as caregiver reported recovery from illness) by Day 7
- Proportion of children with hypoxaemia (according to differing cut-offs) with severe complication

Sub-group analyses will include age (under 2 months, 2 – 12 months, 13 – 59 months), sex, and clinical presentation (with cough / difficulty breathing, fever, other). Exploratory interrupted time series analysis across the 5 quarters of the longitudinal study will be conducted, along with other exploratory analyses of individual and health system factors associated with health and patient-centred outcomes.

Mixed methods sub-studies will mostly be exploratory, to evaluate quality of care outcomes and explore perceptions, values, attitudes and beliefs. Process evaluation criteria will be evaluated according to the Medical Research Council criteria.

Cost and modelled cost-effectiveness will assess:

- Full implementation cost of the intervention per focal country (cost to health system as well as direct costs paid by the household to the health system)
- Cost per child assessed using pulse oximetry devices and CDSA per country compared to without these supports.
- Modelled cost per DALY averted attributed to the introduction of pulse oximetry and CDSAs

Sample size

The sample size for the longitudinal observational study is calculated separately for each country, based on comparison between Q1 and Q5, however the same sample size will also be collected in each of the intervening quarters (Q2, Q3 and Q4) where logistically feasible, for the purposes of secondary analysis over time. In order to detect a difference in referral from primary care from 3% to at least 4.5%, with 80% power, we estimate a sample size in each country as follows:

- Kenya – 17 facilities, recruiting an average of 690 children per quarter per facility, equating to 11,730 children per quarter
- Myanmar – 20 facilities, recruiting an average of 338 children per quarter per facility, equating to 6,760 children per quarter
- Senegal – 18 facilities, recruiting an average of 510 children per quarter per facility, equating to 9,180 children per quarter

For the antibiotic prescription primary outcome, this would enable a minimum detectable reduction of 18% in Kenya and Senegal and of 16% in Myanmar.

Service provision assessments and process mapping will be conducted in 8 – 10 longitudinal study facilities per country, stratified by rural / urban location and facility type, at 5 time points (once per quarter in each facility). At each facility at each time point, 10 – 30 children per facility will be included, resulting in an estimated sample size of 800 – 1000 clinical observations, time-flow observations and exit interviews per country over the study period.

Final sample size for qualitative studies will be determined by reaching thematic saturation. For those countries choosing to conduct qualitative data collection pre-intervention an estimated 10-20 IDIs with caregivers and health care providers and 10-20 KIIs with stakeholders will be conducted. Per country, an estimated total of 35-45 in-depth interviews (IDIs), 6 – 8 walking interviews and 6 focus group discussions (FGDs) will be conducted with caregivers, spread across the period of implementation. Similarly, 35 – 45 IDIs and 6 FGDs will be conducted with healthcare providers, and approximately 45 key informant interviews with stakeholders.

Ethics Review

Following independent scientific merit review, the protocol will be submitted to all relevant research ethics committees / institutional review boards in each country and the WHO ethical review committee.

Estimated study timeline

Pending all relevant ethical approvals, first enrolment is anticipated to be in January 2021, with completion of data collection in March 2022.

Depending on the evolution of the Covid-19 pandemic, we will keep implementation, research processes and timelines under review and inform the necessary bodies and institutions accordingly of any changes.

2 General information

2.1 Trial registration

Intended registry: clinicaltrials.gov

2.2 WHO Trial Registration Data Set

To be uploaded once approved

2.3 Funding

The TIMCI project and associated research described in this protocol are funded by UNITAID. PATH (Seattle) is the primary recipient of the funds and manages the project, with distribution of funds for project implementation to PATH Country offices. All research partners are sub-contracted by and accountable to PATH. PATH is responsible for all procurement related to implementation (including medical supplies and consumables). Pulse oximeters used within the study will be procured through UNICEF.

2.4 Roles & responsibilities

PATH headquarters (Seattle) lead the project, including strategic direction and decision-making, project oversight and compliance, global communications, risk monitoring and mitigation, quality/change management, as well as serving as liaison with UNITAID, the International Advisory Group and other relevant global partners, such as WHO and UNICEF. Additional details on project governance are described below. PATH retain overall responsibility for the project, but have sub-contracted (**Fig. 1**): Swiss TPH to coordinate research design and analysis, which includes collaboration with UniSanté, who lead the design and development of the clinical decision support algorithm; University of Waterloo (UoW) to conduct cost-effectiveness analysis; and the following research partners leading country research activities: King George's Medical University (KGMU, India); University of Nairobi (UoN, Kenya); Burnet Institute (BI, Myanmar); Université Cheikh Anta Diop de Dakar (UCAD, Senegal); and Ifakara Health Institute (IHI, Tanzania). As this protocol relates only to research activities in Kenya Myanmar and Senegal, other country research leads will not be further detailed in this document.

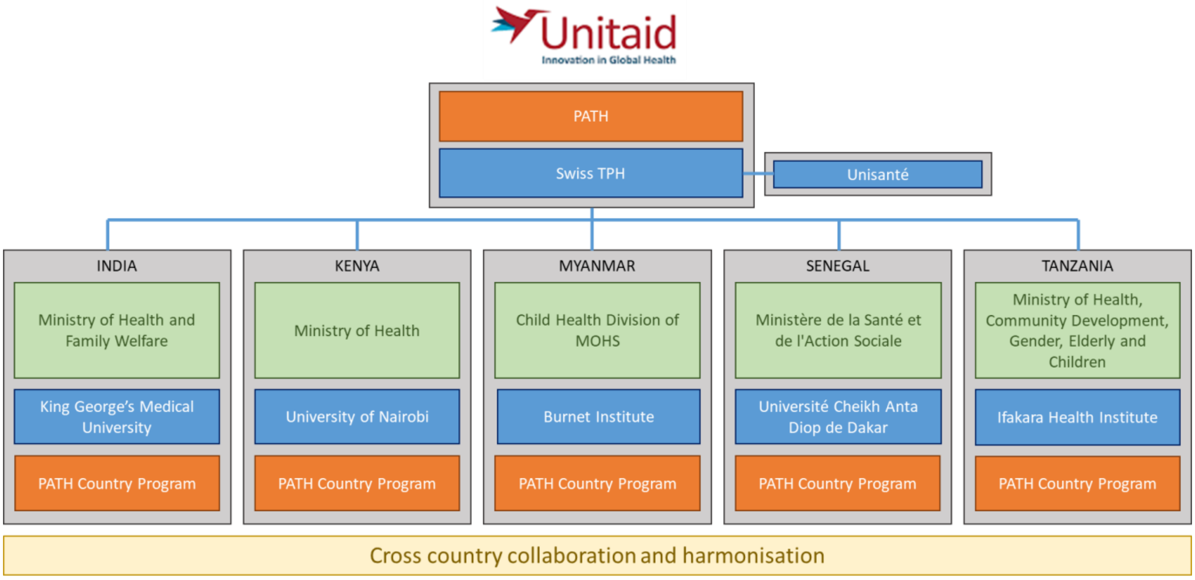

Fig. 1. TIMCI project partners

PATH is the Sponsor of the research but is delegating certain responsibilities to Swiss TPH and country research partners, outlined in **Tab. 1**. Through that delegation, Swiss TPH in collaboration with the research partners in each country will be responsible for the initiation, and management of the research. Each of the three study countries has a separate tri-partite agreement between the country research partner, PATH and Swiss TPH. Within this agreement, Swiss TPH has the lead in development and design of the research. The respective in-country research partner will be responsible for the adaption of the design to the country specific context, in close collaboration with Swiss TPH and PATH.

Tab. 1. TIMCI Sponsor & research responsibilities

	**PATH**	**Swiss TPH**	**Research Partners**
Investigator selection	Lead		
Medical expertise	Review	Lead	Participates
Research design	Review	Lead	Participates
Submission to WHO ethical review	Lead	Review	Review
Submission to country ethical review	Review	Review	Lead
Preparation of study sites	Review	Participates	Lead
Research management		Lead	Participates
Research conduct		Participates	Lead
Data collection		Participates	Lead
Quality assurance & control	Review	Lead	Lead
Data analysis (country specific)		Participates	Lead
Data analysis (cross-country)		Lead	Participates
Dissemination	Review	Lead	Lead
Compensation	Review	Lead	Participates
Financing	Lead		

2.4.1 Protocol development: Swiss TPH

Swiss TPH leads the study design and protocol development in close collaboration with all other partners. The University of Waterloo leads the development and conduct of the cost-effectiveness component of the research, in close collaboration with Swiss TPH, PATH and the country research partners. The longitudinal study with mixed methods sub-studies and cost-effectiveness research will be conducted in three countries, Kenya, Myanmar and Senegal. In each country, Swiss TPH and PATH collaborate with research institutions, all of which have extensive experience in implementing and conducting large-scale projects in their respective study settings. PATH central contributes to the protocol development, ensuring alignment of the proposed research with the intervention and the training packages, which are led by PATH. In both study countries, the local PATH interlink with the research partners **Tab. 2** outlines the investigators and contributors to the protocol.

Tab. 2. TIMCI investigators and contributors for Kenya, Myanmar and Senegal study

	Name	Role	Input
Swiss TPH	Prof. Kaspar Wyss	Co-PI	Oversight & overall responsibility for research design for SwissTPH
	Prof. Valérie d'Acremont	Co-PI	Oversight & overall responsibility for research design for SwissTPH
	Dr. Fenella Beynon	Clinical Research Scientist, Co-I	Quantitative study design and analysis, clinical input for algorithm
	Dr. Hélène Langet	Data Manager, Co-I	Study design, data management oversight, technical input for algorithm, link CDSA into research design
	Dr. Leah F. Bohle	Social Scientist, Co-I	Qualitative study design & analysis
	Dr. Fabian Schär	Project Manager, Co-I	Study design, Ethics approval, timelines and link with research partners
	Dr. Tracy Glass	Biostatistician	Input into study design, data management and analysis
	Dr. Kristina Keitel	Senior Clinical Scientist	Input in development of clinical content for algorithm, input on study design
	Dr. Elisabeth Reus	Clinical Operations	Development of monitoring plan, ethics input
Unisanté	Vincent Faivre	Deputy head of IT department	Software development
	Alan Vonlanthen	Project Manager	UniSanté Project Management, IT department
UoW	Prof. Susan Horton	Cost-effectiveness PI	Oversight and analysis of cost-effectiveness study
	Dr. Nnachebe Michael Onah	Cost-effectiveness Co-I	Design and conduct of cost-effectiveness study in collaboration with country research partners
UoN	Prof. James Machoki M'Imunya	National PI	Overall administrative and scientific lead for Kenya
	Mr. Francis Njiri John	Project Coordinator	Data collection & Management, Ethics approval, timelines and link with partners
	Dr. Peter Mwiti Arimi	Clinical Epidemiology Lead	Clinical Expertise, Study design, Research Implementation
	Dr. Anthony Runo Gichangi	Biostatistician	Data Management, statistical analysis and dissemination

	Dr. Mercy Mugo	Cost-Effectiveness Lead	Design and conduct of cost-effectiveness study
	Dr. Mariah Ngutu	Operations Research Lead	Design, conduct and analysis for the Mixed-methods studies including Observational, Qualitative and Time-flow studies
Burnet	Dr. Myint Myint Than	National PI	Overall lead for Myanmar
	Dr. Michelle Scoullar	Technical Lead, Co-PI	Technical and scientific lead for Myanmar
	Dr Thin Mar Win	R&D Manager, Co-PI	Research implementation and cost effectiveness data collection
	Dr Phone Myint Win	Burnet Myanmar Country Representative	Overall project management lead for Myanmar
	Dr. Aye Aye Myint	Senior Program Manager	Lead Research Implementation
	Ms. Yasmin Mohamed	Technical Focal Point & Project Manager	Project Management Burnet and technical input
	Dr. Anna Bowring	Technical Specialist	Qualitative Lead
UCAD	Prof. Ousmane Ndiaye	National PI	Overall lead for Senegal
	Prof. Papa Moctar Faye	Research Coordinator	
	Dr. Aliou Thiongane	Research Coordinator	
	Dr. Marième Sougou	Consultant - Epidemiologist	
	Dr. Jean Augustin Tine	Consultant - Biostatistician	
PATH	Mr. Mike Ruffo	TIMCI Project Director	Oversight & overall responsibility for TIMCI
	Ms. Mira Emmanuel-Fabula	TIMCI Project Manager	Global Project Management PATH
	Dr. Helen Storey	Senior Research Officer	Lead research PATH on Output 2
	Dr. Manjari Quintanar Solares	Senior Program Officer	Training Lead
	Ms Tara Herrick	Senior Market Analytics Officer	Cost-Effectiveness Lead
	Mr Zachary Clemence	Market Dynamics Senior Associate	Implementation development
	Ms Tanya Lalwani	Senior Program Officer	Monitoring & Evaluation Lead
	Dr. Yi Yi Cho	PATH-Myanmar	TIMCI Country Lead
	Dr. James Mukabi	PATH-Kenya	PATH-Tanzania Country Representative
	Dr. Dan Otieno	PATH-Kenya	TIMCI Country Lead
	Dr. Maymouna Ba	PATH-Senegal	TIMCI Country Lead

2.4.2 Project and research governance

The TIMCI project has several different working groups for governance of implementation and research activities, described in **Tab 3** below. As shown in **Fig. 2**, the research governance for the longitudinal study is shared with the RCT (conducted in India and Tanzania) through a Research Steering Committee (RSC). The RSC is chaired by an independent expert, and will include two sub-groups, which act as management groups, one for the RCT and one for the longitudinal observational study. The Management groups will facilitate the day-to-day running of the RCT and escalate issues to the RSC. Thematic Working Groups will consist of thematic experts from all study countries.

Tab 3. TIMCI project and research governance

**Project Governance**		
Group	Membership	Function
International Advisory Group	Unitaid*, ALIMA, WHO, UNICEF, USAID, experts in paediatrics and IMCI, CHAI<br><br>*Unitaid is an observer in this group	Strategy and guidance focused; shared with ALIMA – recipient of parallel UNITAID award
Country Implementation Working Groups	PATH in-country project lead, leaders of Swiss TPH and research/implementation partners for research activities, key implementation and/or management staff, MOH representatives	Project management focused at country level; ensures close country-level collaboration across multiple organizations and implementation partners; important link to MOH.
Global Implementation Team	PATH country- and HQ-based leadership, Swiss TPH, Country Implementation Working Group leaders	Pan-country strategic and management function; forum to build relationships between countries, share learnings, and ensure alignment.
Consortium Executive Board	PATH and Swiss TPH executive-level leaders, Program Director, Senior Project Manager	Strategic advisory and dispute-resolution committee for PATH and Swiss TPH.
Country-Level Technical Working Groups	Depends upon country; link with most applicable existing group, such as a child health TWGs. Key influencers with MOH, policy, operations	Existing working groups that will provide essential review and oversight in each country; builds and maintains linkages with country operators.
Manufacturers Advisory Group	Influential organizations involved with product development for pulse oximetry, multimodal devices, O2, and possibly clinical or procurement functions	Inform development of TPP, design manufacturer incentive programs, and ensure alignment with market forces.
**Research Governance**		
Research Steering Committee	Independent external chair, with representation from Swiss TPH, and research partners	Regular review of research progress in all five countries with particular focus on trial and longitudinal study
Subgroup one: RCT management group	Swiss TPH, KGMU, IHI	Internal working group for management of RCT study activities
Subgroup two: LS management group	Swiss TPH, UoN, BI and UCAD	Internal working group for management of LS study activities
Thematic Working Groups	Thematic experts Swiss TPH, BI, UoN, UCAD, and IHI, KGMU	Exchange on content-specific topics across all research partners

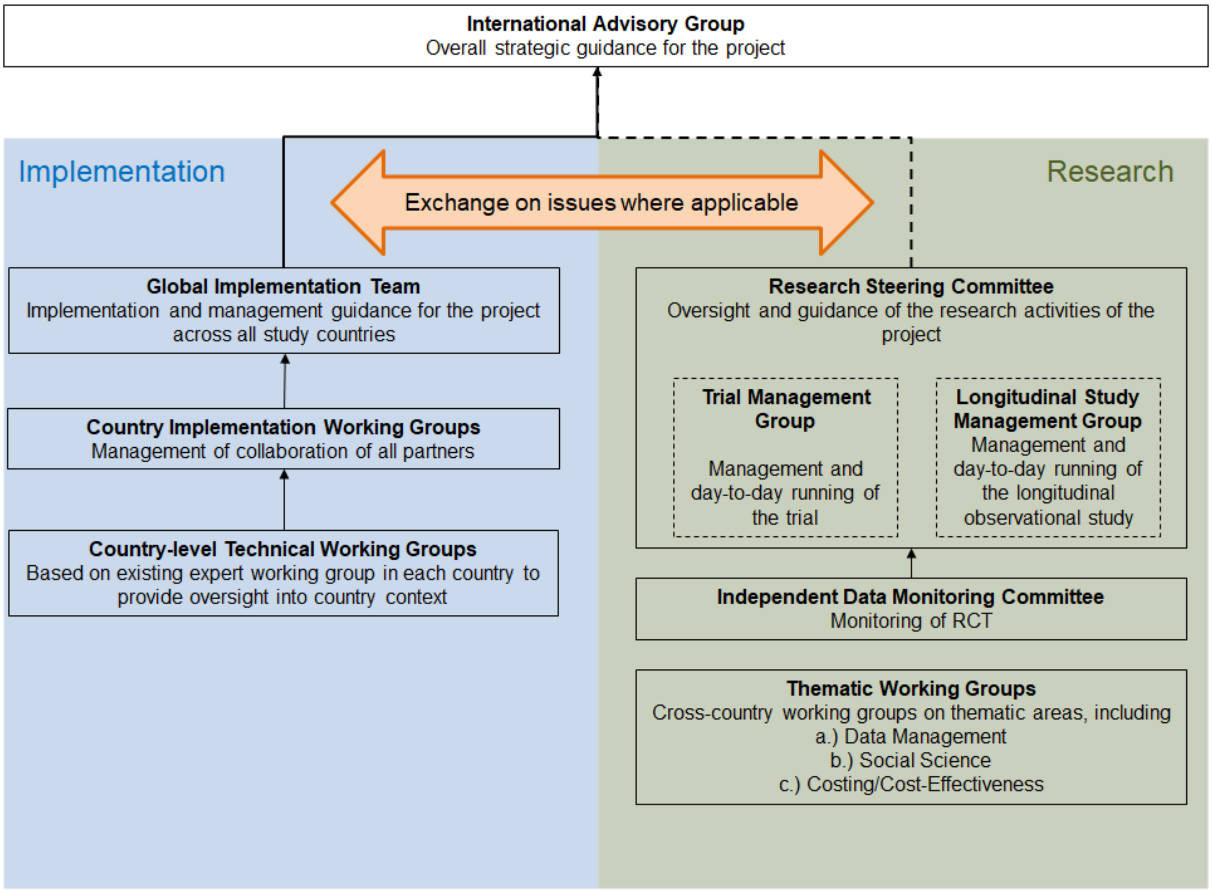

Fig. 2. TIMCI governance overview

3 Background & rationale

Despite progress in reducing child mortality in the last few decades, an estimated 5.3 million children under five years of age died of preventable causes in 2018.[1] If we are to achieve the Sustainable Development Goal 3.2 by 2030, all countries must reduce under-five mortality to at least as low as 25 per 1,000 live births and neonatal mortality to at least as low as 12 per 1,000 live births.[2] Strengthening systems to identify and appropriately treat sick children, alongside health prevention and promotion activities, is critical to achieving this goal.

The Integrated Management of Childhood Illness (IMCI) guidelines, launched in 1995 and now adopted by over 100 countries, responded to the need to systematise the implementation of evidence-based health interventions for children under five in primary care and the community.[3] The case management component provides a simple, structured approach to assessment, classification and treatment of the sick child, with a particular focus on severe illness.[4]

Yet despite the intention of IMCI to have high sensitivity for detection of severe disease, many studies have demonstrated poor identification and management of severely ill children due to both non-adherence by health workers[5–9] and intrinsic problems of guidelines based on clinical signs alone[10–14]. The Tools for the Integrated Management of Childhood Illness (TIMCI) project seeks to address these issues by introducing clinical decision support algorithms and pulse oximetry to strengthen guideline implementation and accuracy in order to improve quality of care and reduce morbidity & mortality.

3.1 Rationale for pulse oximetry

Hypoxaemia, a reduction in blood oxygenation, has a myriad of causes in children – both respiratory and non-respiratory, but regardless of the cause, is strongly associated with mortality.[15] A meta-analysis of 13 studies on 13,928 children found a 5.5 fold increased risk of death among children with acute lower respiratory infection (ALRI) with oxygen saturation (SpO2) below 90%.[15] The association of hypoxaemia with mortality in non-respiratory conditions is less extensively documented, but a recent large study in Nigeria found adjusted odds of death in hypoxaemic children to be 7.1 in ALRI, and range from 5.9 to 6.6 in the next most common causes of hypoxaemia (malaria, acute febrile encephalopathy, sepsis, seizures).[16]

The prevalence of hypoxaemia is estimated to be around 13% among children with WHO defined suspected pneumonia.[17] Prevalence is also high among sick neonates (18 – 23%), malaria (3 – 17%), meningitis (3 – 15%) and malnutrition (2 – 8%).[ibid] Studies have further demonstrated the importance of hypoxaemia in children with asthma, sepsis, malaria, sickle cell disease, tetanus and a variety of neonatal illnesses.[16,18–22] However, the relative contribution of different diseases to the burden of hypoxaemia is highly variable in different settings[17], and likely to reflect the local burden of disease. For example, in the aforementioned study of 23,926 hospitalised children in Nigeria, among all children (28 days – 14 years) with hypoxaemia, 37% had ALRI, 33% malaria, 32% acute febrile encephalopathy, 27% sepsis and 18% seizures. In

countries facing a lower burden of malaria, such as India (2.2% of under 5 deaths are attributed to malaria, compared to 13.2% in Nigeria), whilst systematic data on hypoxaemia burden is not available, this distribution is likely to be rather different.[23]

Given the prevalence of hypoxaemia and its role in mortality, a number of studies have sought to evaluate the predictive power of clinical signs to detect hypoxaemia and guide oxygen therapy. But no single sign or combination of signs has reliably been found to detect hypoxaemia with adequate sensitivity or specificity.[24] Using clinical signs alone thus leads to a failure to identify all children who need oxygen and unnecessary administration of oxygen (which is often in scarce supply) for children who do not need it.[24]

Pulse oximeters provide a non-invasive, accurate and easy to use method of evaluating hypoxaemia. A number of observational studies at hospital level have shown significant reductions in child mortality, referrals and triage time following the introduction of pulse oximetry and associated interventions.[13,25,26] Furthermore, a recent modelling study estimated that implementing pulse oximetry could avert up to 148,000 deaths per year of children under-5 in fifteen high-burden pneumonia countries, far more than the potential deaths averted by IMCI alone.[27] In addition to the potential impact on child morbidity and mortality, there are potential resource savings through the reduction in unnecessary oxygen use – up to two thirds of children eligible for oxygen based on WHO criteria are not hypoxic.[14]

Whilst most data and guidance relating to pulse oximetry pertains to hospital settings, there is emerging evidence on the benefit in primary care. In Malawi, provision of pulse oximetry to rural health centres and community health workers resulted in increased referral rates among children with WHO-defined severe pneumonia.[13] They also found that pulse oximetry enabled the identification of hypoxaemic children who otherwise would have been categorised as having non-severe pneumonia. Whilst their study was implemented before the 2014 modification to WHO IMCI guidelines, retrospectively applying the criteria to their dataset revealed that 69% of hypoxaemic children would not have been categorised as requiring referral. Pulse oximetry therefore provides the opportunity to improve the sensitivity of IMCI guidelines for severe disease. Implementation of pulse oximetry could also be a strategy to rule-out severe disease and therefore reduce unnecessary hospital referrals and admissions, though the exact oxygen saturation cut-off requires further validation.[28]

This potential to improve the detection and referral of children with severe disease has led to strengthened interest in the potential to rollout pulse oximetry to primary care. However, before significant investment is made in this area, it is critical to understand what health impact pulse oximetry has, for which children in which contexts, and with what consequences – such as over-referral to already stretched hospital resources. Impact is likely to depend on a number of factors, including burden of hypoxaemia in primary care, choice of criteria for pulse oximetry use (to optimise detection of hypoxaemic children whilst avoiding false positives and over-burdening clinic staff) and pre-existing quality of care.

3.2 Rationale for clinical decision support algorithm

Whilst the introduction of pulse oximetry can improve the identification of sick children in primary care, it does not provide a comprehensive approach to improve adherence to evidence-based guidelines. Observational studies have indicated that IMCI-trained healthcare providers only correctly classify children according to IMCI guidelines 33 – 87% of the time.[5] This lack of correct classification likely stems from incomplete assessments. Pooled data from the Service Provision Assessment surveys in Namibia, Kenya, Tanzania and Uganda found health workers conducted a complete danger signs assessment in less than 33% of 6856 children assessed.[6] In Malawi, fewer than 1% of children had an assessment including all 16 elements of the history and examination.[9] In the same study, respiratory rate was only checked in 16% of children presenting with cough or difficulty breathing.

Failure to assess key signs can result in under-classification of severity and inadequate management of children with severe disease, leading to a higher mortality risk. For example, in the above study in Malawi, clinical officers did not refer 41% of children with severe / very severe pneumonia, and requested oxygen for only 23% of those that met criteria.[9] Similarly, in a study in South Africa, only 47% of children with severe disease were correctly identified by a health worker.[7]

Even when health workers do recognise severe disease, they do not necessarily treat and refer according to guidelines. In a study in Tanzania, only 38% of children with severe disease were referred by IMCI-trained health workers, despite 95% having been recognised as having severe pneumonia, severe malaria or both.[8] The authors surveyed health workers and found that 91% believed that referral was not a necessary component of management for certain severe conditions.

Electronic clinical decision support algorithms (CDSAs) provide an electronic step-by-step guide through a clinical consultation for healthcare providers, supporting the integrated disease management approach promoted by WHO. Recent studies of two such tools, based on IMCI and national guidelines, have demonstrated potential for significant improvements in health outcomes and large reductions in unnecessary antimicrobial prescribing.[29,30] The use of one such tool by healthcare providers in consultations for all acutely unwell children was found to improve cure rate at day 7 (97% compared to 92% in routine care) whilst reducing antibiotic prescriptions from 84% to 15%.[29] Another tool, ePOCT, used for children with fever and integrating point of care diagnostic tests, reduced clinical failure rate to 2% (compared to 5% in routine care) and antibiotic prescriptions to 11% (from 95%).[31]

The mechanisms by which CDSAs impact health worker practice may stem from the fact that the electronic algorithms, by design, provide support in decision making for any combination of clinical findings, whereas paper-based IMCI or national guidelines are, by necessity, reductionist.[10] In a mixed-methods exploration of the reasons for non-adherence to IMCI guidelines by health workers, Lange and colleagues described "cognitive overload" as a contributory factor to non-adherence.[32] In such circumstances, support to the complex clinical

decision making process with CDSAs can facilitate health workers to provide higher quality care. This is likely to be particularly true in the case of introducing novel technology such as pulse oximetry, which adds an additional level of complexity to the integrated clinical assessment of children.

Implementing pulse oximetry embedded into a CDSA has the potential to improve the detection of children with severe disease and improve the adherence of healthcare providers to evidence-based, integrated disease management guidelines. We hypothesise that these interventions will result in a reduction in adverse health outcomes through better treatment and referral decisions, and an improvement in quality of care through the rational use of essential medicines and improvements in the health worker and caregiver experience.

3.3 The TIMCI project

The Tools for Integrated Management of Childhood Illness (TIMCI) project builds on this hypothesis to form part of the response to the need for better mechanisms to detect and manage children with severe illness in LMICs. It is a 4 year UNITAID-funded project, focused on the interventions of pulse oximetry and CDSAs in primary care, organised into four main outputs: (1) introduction of pulse oximetry and CDSAs in five countries (India, Kenya, Myanmar, Senegal and Tanzania); (2) evidence generation for pulse oximetry and CDSA introduction; (3) market strengthening for adapted multi-modal devices; and (4) creating conditions for national and global scale-up and sustainable use of these tools.

This protocol focuses on the evidence generation component of the TIMCI project in Kenya, Myanmar and Senegal (a separate protocol has been developed for India and Tanzania to reflect a different study design).

To our knowledge, no large scale studies have systematically evaluated the health impact, cost and optimal implementation approaches of pulse oximetry, alone or embedded into a CDSA, for children with a broad range of clinical diagnoses in primary care.

4 Goal & objectives

The overall goal of the TIMCI project is to reduce morbidity and mortality in sick children attending primary care facilities, while supporting the rational and efficient use of diagnostics and medicines by healthcare providers. The evaluation component of the project in Kenya, Myanmar and Senegal seeks to generate evidence on the health impact, operational priorities, cost and modelled cost-effectiveness of introducing pulse oximetry, embedded into a CDSA, into primary care in LMICs, for children 0 – 59 months of age, to facilitate national and international decision-making on scale-up. Specific objectives, according to these themes, include:

Health and quality of care impact

To evaluate the impact on health and quality of care outcomes of sick children 0 – 59 months of age of introducing pulse oximetry, embedded into a CDSA, for facility-based primary care, compared to routine care, including on:

- Referral and hospitalisations
- Antimicrobial prescription
- Adherence by primary healthcare providers to key components guidelines

To assess the prevalence of severe, moderate and mild hypoxaemia among sick children attending primary care, and understand the association of different degrees of hypoxaemia with adverse outcomes, according to socio-demographic and clinical features.

Implementation and process

- To understand the facilitators and barriers to uptake, and acceptability of the intervention and behaviour change by health care providers, caregivers and their children, and a variety of stakeholders at community, health facility, national and international levels
- To evaluate characteristics of children, caregivers, healthcare providers and health facilities associated with uptake and impact of the interventions
- To understand how the interventions affect patient flow and patient-provider interactions, including change in care processes, consultation time, and communication between caregivers and healthcare providers

Cost and modelled cost-effectiveness

- To determine the cost and estimate any cost savings and/or additional costs of introducing pulse oximetry and CDSAs into primary health care

- To model cost-effectiveness of the interventions compared to routine care and other interventions aimed at improving the management of severe illness in children 0 – 59 months of age

5 Study design

Health impact will be assessed through a longitudinal study, comparing clinical care of children in a three-month pre-intervention period (baseline) with four sequential three-month periods over the following year (intervention). The intervention package includes pulse oximetry embedded into a CDSA, with training, supervision, monitoring and community engagement, further described in Section 6.3. This intervention package will be piloted and refined in parallel to the pre-intervention period of the longitudinal study.

This assessment of the health impact of the intervention will be complemented by embedded mixed methods sub-studies to evaluate other key components of quality of care[33] and gain a deeper understanding of the implementation mechanisms and context. These studies include modified Service Provision Assessments (SPAs), a facility-based process mapping and time-flow study, qualitative studies with caregivers, healthcare providers and key stakeholders, a regular survey sent to selected key stakeholders, a desk-based document and monitoring and evaluation (M&E) review, and an economic evaluation (**Fig. 3**, **Tab. 8**).

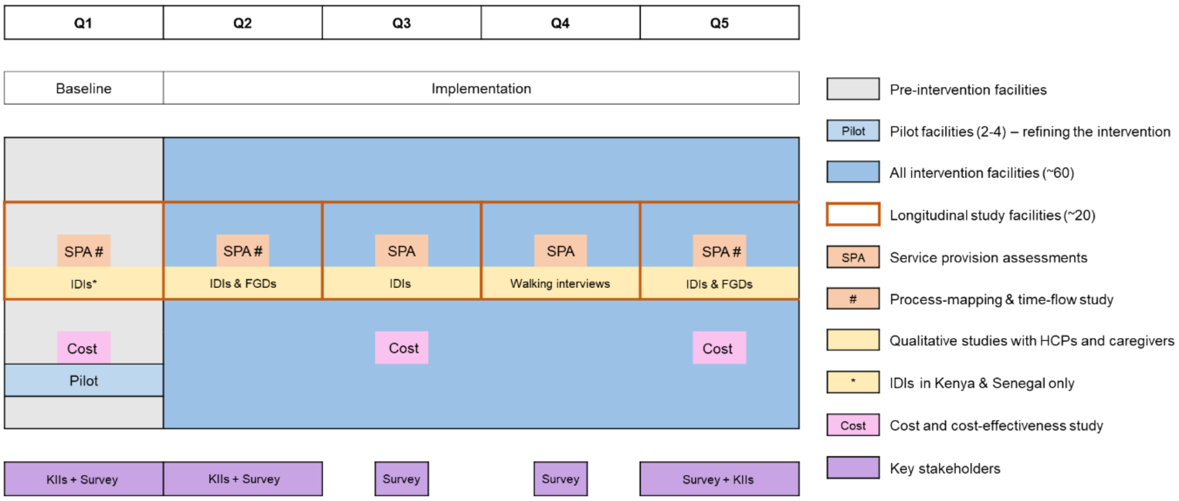

Fig. 3: Timeline of TIMCI research in Kenya, Myanmar and Senegal. **Longitudinal study:** comparing clinical care of children in a three-month pre-intervention period ('baseline') with four sequential three-month periods over the following year ('implementation'). **Pilot** – 2 to 4 facilities in parallel to longitudinal baseline, to refine the intervention. The longitudinal study dataset will be collected in these facilities, along with SPA and process mapping, caregiver IDIs and HCP IDIs. **SPAs** – cross-sectional quarterly assessments in 8-10 facilities comprising of observations of consultations of sick children, exit interviews with caregivers, interviews with HCPs and facility assessments. **Process-mapping and time-flow** – during baseline, early and late implementation. **Qualitative studies with caregivers and with HCPs** – at pilot facilities (IDIs only) and across the implementation period to understand change over time. In Kenya and Senegal, IDIs will also take place in the baseline/pre-intervention period. **Key stakeholders** – KIIs during baseline / pilot, early and late implementation and quarterly surveys. **All intervention facilities** – PATH M&E data, ePoct+ clinical consultation data, Health Management Information System data. **Cost and modelled cost-effectiveness:** cost data will be collected prior to intervention and on start-up costs, and again during implementation with final data collection to capture any missing information. **Abbreviations**: **SPA** – service provision assessments; **IDIs** – in-depth interviews; **FGDs** – focus group discussions; **KIIs** – key informant interviews; **HCPs** – healthcare providers

The intervention will be implemented in approximately 60 health facilities providing primary care services. Longitudinal, mixed-method and costing studies will be conducted in only a sub-

sample of these facilities, with triangulation across studies to generate in-depth insights into implementation. Routine data from non-study facilities will be extracted for analysis of the wider programme, including from the health management information system (HMIS), anonymised routine clinical data from the CDSA, and PATH project data.

This mixed methods design draws on principles of realist evaluation (focusing on processes, and mechanisms in their respective contexts leading to outcomes)[34], the MRC guidance on Process Evaluation for Complex Interventions[35], the Theoretical Framework of Acceptability of Health Care Interventions[36], and the COM-B model of behaviour change[37]. It is further informed by the project's Theory of Change (ToC, appendix), developed in line with the approach taken by de Silva and colleagues[38] and the Centre for Theory of Change[39]. The ToC components will be tested and revised to incorporate unanticipated pathways and assumptions and provide feedback to strengthen the intervention.

5.1 Pilot phase

The pilot phase will occur over an estimated 3-month period in small number of facilities in each country whilst the pre-intervention assessment is occurring in the longitudinal study facilities. It will serve to refine the intervention package if necessary, including the approach to training, supervision, caregiver engagement and guidance on where to use the devices within the care process. This refined package will then be implemented in the remaining facilities involved in the TIMCI project.

The longitudinal study dataset will be collected from these pilot facilities and will be used for exploratory analysis in comparison with data from the main longitudinal study. We will also collect SPA, process mapping and time-flow data and qualitative data (from interviews with caregivers, healthcare providers and key stakeholders; possibly informal group discussions, feedback from post-training assessments by PATH). These studies will aid understanding of implementation processes, mechanisms and context. Emphasis will be placed on exploring acceptability, and barriers and facilitators to implementation to inform fine-tuning and adaptations of the intervention.

Concurrently, we will pilot the research instruments and processes and refine if necessary, including eligibility screening, participant information and consent, and data collection tools.

5.2 Longitudinal observational study

A quasi-experimental longitudinal observational study, comparing clinical care of children in a three-month pre-intervention period with four sequential three-month periods from intervention start. The last post-intervention three-month period will correspond to the same period of the year as the pre-intervention three-month period, to allow for comparison between two similar seasonal time points within the 15-month study period. Interrupted time

series analysis will be conducted across the five three-month periods to assess change over time.

5.3 Service provision assessment

This periodic assessment of service delivery will follow the approach of the Demographic and Health Surveys (DHS) Program Service Provision Assessment (SPA) (facility assessment, healthcare provider interview, sick child observation protocol and caregiver interview),[40–42] modified to focus on child health, pulse oximetry and CDSA use and incorporate additional quantitative and qualitative questions covering factors associated with child mortality and health-seeking behaviour, as well as patient-centred outcomes including experience of care.

We will conduct cross-sectional surveys at a stratified sample of facilities at baseline and quarterly over the following year in order to assess differences over the course of implementation and with varying burden of disease in different seasons. Stratification is based on rural / urban location, facility type, and, where applicable, district and altitude. On the days of the surveys, we will conduct an abbreviated facility assessment, administer a questionnaire to all providers consulting children under five, and a modified 'sick child' observation and exit interview on all eligible children attending the facility.

5.4 Facility-based process mapping and time-flow study

Prior to intervention, we will develop a basic process map defining the common steps in the care pathway of sick children in primary care facilities in each country (adapted to facility type). The map will be reviewed and refined through informal discussions with staff and non-participant observation at a sample of facilities (including on whether specific processes have been introduced or changed as a result of COVID-19). Time-flow data will be captured for discrete steps in the care process through observation of a sample of children attending the facility, from arrival through to consultation (including on use of the devices), to departure from the facility. Non-participant observation will focus on understanding rationale for care processes and when and why deviation from the standard pathway may occur.

This pre-intervention map will be adapted to incorporate pulse oximetry and CDSAs through discussions with staff and observations in the pilot facilities to understand the impact of the intervention on care processes (including on time) and on patient-provider interactions, and inform refinement of the intervention strategy if necessary. After roll-out of the intervention, this will be repeated both early and late in the intervention to understand changes in care processes and time with the refined intervention package, and adaptations that may have occurred as a result of provider preferences (or as a result of other changes to care processes external to the TIMCI project). This will also generate information on whether the intervention has been normalised into care processes over the longer-term in order to understand sustainability and inform the package for scale. Time-flow data will also be used in the cost and modelled cost-effectiveness study.

5.5 Healthcare providers' acceptability and perceptions of the intervention

We will conduct semi-structured in-depth interviews (IDIs) and focus group discussions (FGDs) with clinical vignettes with a purposive sample of healthcare providers working in intervention facilities. IDIs (and informal group discussions) will be conducted at the pilot facilities to inform intervention refinement. IDIs and FGDs will be conducted over the course of the intervention to understand changes in perception of the intervention over time and reasons for early and late adoption. In Kenya and Senegal only, IDIs will also take place pre-intervention for comparison. FGDs will be based around clinical vignettes to facilitate discussion of experiences with the devices (including usability, practicality and design) and on quality of care and acceptability of tools without causing unwanted disclosure, or bias due to self-stigmatization or self-censorship. IDIs, which may include vignettes, will focus particularly on the individual experience with the introduction of the intervention and over time; the acceptability of the intervention; mechanisms, processes and contexts leading to behaviour change and identifying barriers and facilitators for intervention success.

5.6 Caregiver perceptions of the intervention and health-seeking behaviour

Semi-structured IDIs, walking interviews resulting in case studies (CS) and FGDs will be conducted with a purposive sample of caregivers who have attended study facilities. IDIs and FGDs will be carried out at the household and respective community level.

IDIs (and informal group discussions where required) will be carried out with caregivers attending pilot facilities to inform intervention refinement. IDIs, walking interviews and FGDs will be conducted with caregivers attending intervention facilities over the course of the implementation period to understand changes in perception of the intervention over time and behaviour change. In Kenya and Senegal only, IDIs will also take place pre-intervention for comparison. The IDIs, walking interviews and FGDs will focus on aspects of the quality of care and acceptability frameworks, as well as health-seeking behaviour and decision making, incorporating a realist approach. Walking interviews will delve into greater detail on decision-making on and experience of referral.

Classical FGDs will be paired with focus groups incorporating method acting (i.e. adaptation of interactive performance) structured in a three-step approach including verbalized senses and experiences, embodied images and performance, using photo- and or video-recording. Method acting is particularly useful when conducting focus groups with participants who have low health literacy or in groups where hierarchy between participants predominates, and can enable empathetic associations, linking knowledge, attitudes and behaviour to personal lived experience.

Walking interviews will be conducted with a purposive sample of caregivers having received referral advice and after their visit at an intervention facility allowing for case studies. Walking

interviews will use an explorative theme catalogue focusing on the care seeking pathway and decision-making. When accompanying the caregiver, the pathway will be tracked by the data collector (e.g. by using a mobile device and GPS data). Hand-written notes and GPS coordinates from qualitative data collected will be used for mapping of care seeking pathways.

Participants with a particularly interesting or divergent view in focus group discussions with health care providers or caregivers might be approached for follow-up IDIs.

Qualitative data collection is exploratory in nature; time points in data collection are preliminary. Final time points will depend on the preliminary analysis of qualitative data and findings throughout the data collection. Therefore the studies planned may require a closer follow-up or data collection at additional time points (e.g. mid phase of the intervention).

5.7 Stakeholder perceptions and project data review

This will incorporate key-informant interviews (KIIs) with selected stakeholders at global, national, subnational and local level with perspectives on implementation, policy and research. Stakeholders will include those internal (i.e. project stakeholders, e.g. PATH) and external (i.e. non-project stakeholders e.g. Ministry of Health, community, other organisations) to the project, either directly involved in implementation or affected by implementation. This includes, but may not be limited to: selected International Advisory Group members, Unitaid and WHO representatives, PATH headquarters staff, national policy makers, members of district health management teams, health facility managers, community leaders, national and local PATH implementation staff and research staff. The KIIs will be conducted during the pilot phase, to understand perspectives at the start of implementation, and during late intervention, to gain reflections into implementation over time and to inform a package for scale. In Kenya and Senegal only, KIIs will also be conducted in the pre-intervention phase to understand health care provision prior to implementation.

These interviews will be complemented by quarterly electronic surveys of selected project informants and a structured review of key documents including PATH project reports (including on changes as a result of COVID-19), minutes and M&E data (e.g. on training evaluation or maintenance and replacement of devices) for each country. It will primarily focus on the processes, mechanisms and context (i.e. MRC process evaluation criteria) of implementation including adaptations made.

In each country, PATH project data, along with routine Health Management Information Systems (HMIS) data, and anonymised data from clinical consultations with the CDSA routinely entered by healthcare providers will be assessed for all facilities and administrative regions where the project is running (i.e. both study facilities and non-study facilities) to draw inferences on the wider project implementation.

5.8 Cost and modelled cost-effectiveness study

The economic evaluation will involve a cost analysis and modelled cost-effectiveness using direct health system costs in which financial and economic costs will be assessed. The scope of the costing will cover only intervention costs and not research-based costs, and will include costs incurred by the health system as well as estimated direct costs paid out of pocket by the households to the health system. It is important to note that this cost methodology is not comprehensive. It does not include the indirect costs incurred by the consumer (patient) e.g. the caregiver's time or transport costs. Rather, it focuses exclusively on the provider's direct costs, whether borne by the health system or paid out of pocket by the patient's household.

The approach that has been taken in applying this methodology has been to capture what is thought to be the "most likely" cost scenario, on average. In identifying this "most likely" cost scenario, effort will be made to supplement the analysis with a discussion about factors that influence the level of costs (e.g. the number of trainees per training, or the frequency of supervision) so as to provide a type of sensitivity analysis of the robustness of the estimates. Cost scenarios will also guide possible future programme scale up. The cost resources for each cost centre might differ across the three countries. For instance, at some facilities laboratory tests or some treatments may not be available, and patients have to be referred elsewhere: we will have to take account of these differences.

Research-based costs are excluded to enable the costing to provide guidance on future replication and potential scale up of the intervention. Full and incremental costing involving an activity-based and ingredient-based approach will be used. When available, effectiveness data will be combined with the cost data and cost per DALY averted will be estimated as outlined in 7.3.3. The costing and modelled cost-effectiveness will use a 12-month time frame and a 3% discount rate. Where applicable, discount rates will be adjusted to prevailing rates at country-level.

6 Participants, interventions, and outcomes

6.1 Study setting

Whilst the study is taking place in diverse health systems and communities in Africa and Asia, outlined below, the common feature is the focus on facility-based primary care. Caregivers often attend a diverse range of facilities to meet their primary care needs. The study setting therefore includes a diverse range of facilities providing primary care services, including outpatient settings within larger health centres in addition to the more traditionally-labelled primary care facilities.

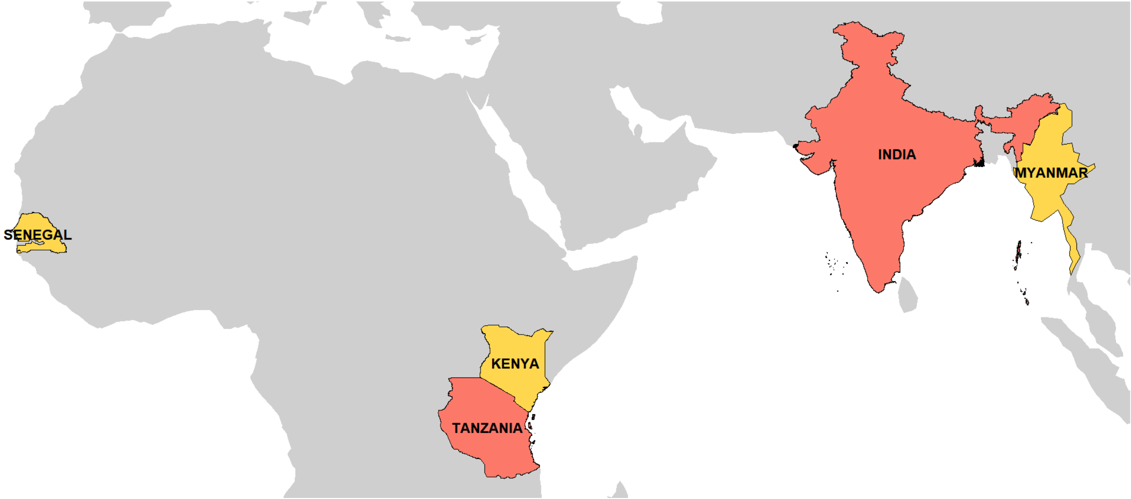

Fig. 4. Map overview of the countries that are involved in TIMCI. Countries conducting the pragmatic cluster RCT (India and Tanzania) are displayed in red, while countries conducting the longitudinal observational study (Kenya, Myanmar and Senegal) are displayed in yellow.

6.1.1 Kenya

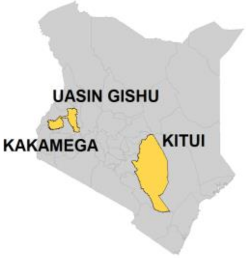

Setting

Health dispensaries (Level 2) and **health centres/sub-county hospitals (Level 3)** in three counties of Kenya (Kakamega, Kitui and Uasin Gishu). They have been selected due to the expected numbers of children presenting in the health dispensaries and health centres/sub-county hospitals, as well as absence of other interventions. Kitui altitude averages 400-1800m (Kitui County CIPD, 2018), Kakamega county 1535m (Kakamega County CIPD, 2018) and Uasin gishu from 2700 m – 1500m (Uasin Gishu CIPD, 2018).

Services & Staffing

Both health dispensaries and HCs have opening hours 8am-5pm.
The health dispensaries in the cities act like a health centre, with the difference that the dispensary does not have in-patient facilities.
HCs are small hospitals with minimal facilities, yet they offer services like the big hospitals. Health dispensaries are run by clinical officers.
HCs are run by at least one doctor, clinical officers and nurses.

Referral system & O_2 availability

Ambulances located in hospitals are usually called to refer emergency cases. However, most stable patients are given referral notes by the referring health facility and they organize their own transport to the next level of care. Neither dispensaries nor HCs have cylinder-based oxygen.

6.1.2 Myanmar

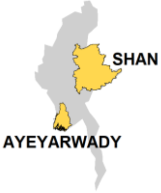

Setting

Sub-centres and **rural health centres** (RHCs) and outpatient departments of **station** and **township hospitals** in the Myaungmya district of the Ayeyarwady region and the Nyaung Shwe district in the Southern Shan region. Each RHC has four to five sub-centres. Sub-centres have a catchment population of 5,000-7,000 people and RHCs have a catchment population of 20,000 – 30,000.

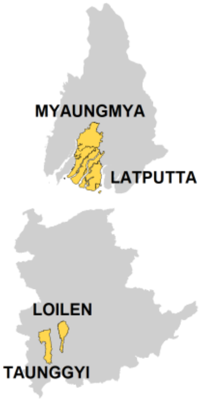

Services & Staffing

Primary health care services such as health education, immunization, nutrition, disease control, maternal health, and treatment of common illness including childhood illness. Each sub-centre is staffed by a midwife at the village level. RHCs are staffed by a health assistant, 1 LHV (lady health visitor), and 1 midwife, 1 public health supervisor (grade 1), 1 public health supervisor (grade 2).

Referral system

RHC and Sub centres usually refer patients to either station hospital or township/district hospital depending on the distance and transportation feasibility. If required, station hospital or township/district hospital refer patients to tertiary hospitals. Sometimes, RHC and sub-centers directly refer to tertiary hospital especially in Taunggyi.

O_2 availability

Neither sub-centres nor RHCs have pulse oximeter or oxygen available. Station hospitals are the lowest level with oxygen available.

6.1.3 Senegal

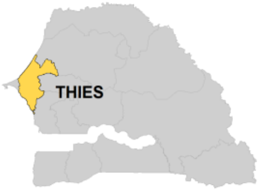

Setting

Health posts (HPs) in the Thiès region.

Services & Staffing

Health posts are open between 8 am and 4 pm. They offer primary health services like prenatal care, births, nutrition assessment, immunization, care for sick babies etc. They are staffed with 4 to 5 health care providers on average. No inpatient care for children or adults. No permanent staff for nights and weekends, but nurses may be contacted if needed.

Referral system & O_2 availability

The HPs refer to health centers and health centers refer to hospital level 1, 2 or 3. From HPs, generally only non-medicalized ambulances are available. Oxygen is available in most referral health centers but still of limited use. Most HPs do not have oxygen yet. Pulse oximeters are used in some health centers and not in HPs, except for sites enrolled in the Oxygen Access project led by Air Liquide.

6.2 Eligibility criteria

6.2.1 Longitudinal observational study and pilot

Facility inclusion:

- Consenting government-designated healthcare facilities within the selected geographical areas of each country
- Providing curative primary care services for children 0 – 59 months of age
- Oxygen available or referral mechanism in place with oxygen available at a higher level facility

Facility exclusion:

- Attending to fewer than 20 sick children per month (based on the prior 12 month average)
- Already using pulse oximetry as a routine part of outpatient-based consultations of children 0 – 59 months
- Selected to be part of a major child health programmatic or research intervention during the study period likely to significantly affect the primary outcome
- Inaccessible to the study team (e.g. due to weather conditions or security issues) for significant parts of the year.

Individual child inclusion:

- Children 0 – 59 months for whom caregivers provide consent
- Consulting for an illness, or reported to be unwell when attending for a routine visit (e.g. vaccination, growth or chronic disease monitoring)

Individual child exclusion:

- Children in the immediate post-natal period or first day of life
- Attending for a consultation related to trauma only (including new and follow-up presentations for burns, injuries, wounds)
- Admitted within an inpatient part of the facility (including neonates delivered at the facility admitted with their mother)
- Enrolled in the study within the preceding 28 days at any study facility

6.2.2 **Other mixed methods studies**

Facilities for the remaining studies will be sampled from the longitudinal study facilities (and pilot facilities for healthcare provider and caregiver IDIs). A stratified sample of facilities will be selected for the SPA and process mapping and time flow study, with stratification factors including location (rural/urban) and type of health facility (if applicable, regional differences and altitude may also be taken into account). Sub-samples for qualitative studies with healthcare providers and caregivers will be sampled from SPA facilities to allow for triangulation of data between studies, but may also be sampled from non-SPA facilities to avoid research fatigue or amplification of the Hawthorne effect.

Individual children and caregivers will be eligible for inclusion in the SPA if they attend a study facility on the day of the SPA and are enrolled into the longitudinal study. Caregivers will be eligible for inclusion in IDIs, walking interviews and FGDs if their child has been enrolled in the longitudinal study.

Individual healthcare providers will be eligible for the SPA interview if they are present on the day(s) of assessment and are involved in the care of children 0 – 59 months at that facility. Healthcare providers for IDIs and FGDs will be eligible if they provide care for children 0 – 59 months at any study facility.

Individual stakeholder eligibility: policy, implementation or research stakeholders at a national or international level with expertise in child health and / or the devices introduced; local and sub-national stakeholders either internal or external to the project, directly involved in any aspect of implementation or affected by the intervention.

6.2.3 Cost and modelled cost-effectiveness study

Medical and non-medical personnel from study facilities and the government-designated hospitals to which they refer will be approached for costing data as described in section 6.6.3.

6.3 Interventions

The intervention package included in the TIMCI project is comprised of introduction of pulse oximetry, incorporated into a CDSA, supported by targeted training. This will be embedded into monitoring and supportive supervision systems, and link with existing systems to provide device maintenance and replacement parts. Community engagement will complement the facility-based intervention, to promote health-seeking behaviour and compliance with referral advice.

Pulse oximetry

UNICEF-approved handheld pulse oximeters (Acare, AH-MX)[43] will be provided, along with a package of supplies that includes a carry case, spare set of rechargeable batteries, battery charger, and six reusable probes (two each of adult, paediatric and neonatal sizes). Handheld devices were selected as they are portable, and thus better suited for point-of-care use in PHC settings, and more affordable than benchtop devices. Handheld devices are also more appropriate for children and neonates than fingertip devices, which are typically not designed for these populations.

The criteria for pulse oximetry use, agreed with the International Advisory Group and Ministries of Health:

- All children under 2 months of age
- Children 2 – 59 months of age presenting with cough / difficulty breathing
- Children 2 – 59 months of age with IMCI signs of moderate / severe disease (IMCI 'yellow' or 'red' classification)

These criteria aim to identify children with the highest likelihood of having hypoxaemia whilst avoid unnecessary referrals of children with a low likelihood of having hypoxaemia and a false positive result, as well as avoiding over-burdening healthcare providers or contributing to clinic delays.

Young infants represent a small proportion of all clinic attendees[44], but with a relatively high prevalence of hypoxaemia and risk of severe disease and mortality.[1,7,16] Children with cough or difficulty breathing represent around 40 – 70% of children attending primary care. Children with WHO-defined suspected pneumonia (around 15 – 30% of clinic attendees)[7,44,45] are more likely to be hypoxaemic than those with cough / cold, but respiratory rate, one of the key differentiating signs is often not measured.[16,45,46] All children with cough / difficulty breathing are therefore chosen to avoid missing children inappropriately classified as not having suspected pneumonia.

Lastly, children presenting with signs of moderate or severe disease are included to ensure detection of children with illnesses such as sepsis, meningitis, malaria, malnutrition, diarrhoea, anaemia that may also present with hypoxaemia.[16,17] Children with mild disease – such as diarrhoea with no evidence of dehydration, fever with no evidence of malaria, meningitis / encephalitis, or complicated measles – are very unlikely to have hypoxaemia and pulse oximetry use will therefore not be advised in this group.

In certain circumstances, there may be a deviation from these criteria for operational reasons. Such circumstances will be agreed with local decision-makers, but, for example, may include the use of pulse oximetry during a triage step in large, high volume facilities. In such circumstances, preference may be given to using pulse oximetry on all sick children. In the event that this occurs, children found to have low oxygen saturation will be triaged for immediate assessment by a clinician, who may repeat the pulse oximetry measurement if needed.

Healthcare providers will be advised to use a cut-off of SpO2 <90% for referral, unless specific national guidance states otherwise (for example in Senegal, where it is likely that a <92% SpO2 cut-off may be instituted nationwide).[47] The message to refer children with other signs will be reinforced. Different SpO2 cut-offs for referral for sites at high altitude will be finalised with the IAG and MoHs, given that the normal range for SpO2 varies with altitude, but this is unlikely to affect cut-offs below 2500m.[48]

Healthcare providers will be advised to only take one measurement for children with severe or moderate disease and will be advised not to attempt to obtain a reading for more than 5 minutes (most readings are obtainable within this time)[13,49,50]. However, if a child with no other sign of moderate or severe disease (i.e. IMCI 'green' category) is found to have SpO2 <90% (or altitude-specific cut-off), healthcare providers will be advised to repeat the reading. In cases of conflicting readings (i.e. one <90% and one ≥90%, or altitude-specific cut-off), a third reading will be advised as confirmatory.

The above guidance will be incorporated into the CDSA, described in detail below.

Clinical Decision Support Algorithm

In addition to pulse oximetry, healthcare providers in the pulse oximeter plus Clinical Decision Support Algorithm (CDSA) will be guided throughout their medical consultations by an

algorithm called ePoct+ and developed by Swiss TPH in collaboration with Unisanté/Wavemind (Lausanne, Switzerland).

Healthcare providers will use ePoct+ for the assessment, diagnosis and management of all sick children 0 – 59 months of age (i.e. only excluding those coming for routine immunisation / growth check visits who are not reported to be unwell). In case a child comes back to the facility during the same disease episode, ePoct+ can be used to manage the child in the same way as during the baseline visit, as the algorithm takes into account duration of symptoms and previous treatments provided.

MedAL-R, the front-end application developed by Unisanté/Wavemind to implement ePoct+ is available for Android tablet or PC. The application can be deployed in health facilities in a standalone mode or a multi-device mode to facilitate the collaboration of several healthcare providers and storage of information in the patient's medical records. In the context of this study, MedAL-R will be deployed on tablets. The choice of a standalone / multi-device setting depends on discussions with the country MoHs.

The clinical algorithm will be adapted to reflect national guidelines for the management of children 0-59 months of age in primary care (based on IMCI and additional relevant case management guidelines for children 0 – 59 months). An expert group will be established and consulted in each country using a Delphi method approach and / or nominal group technique to validate the clinical algorithm.

The adaptation of ePoct+ will be done using MedAL-C, the back-end software developed by Unisanté/Wavemind to manage, reference, modify and deploy the different versions of the clinical algorithm into MedAL-R. MedAL-C allows an interactive visualization of the clinical algorithm and therefore clinicians without any IT programming skills can modify the decision trees to release a new version of the clinical algorithm. Recommendations on use of additional point of care laboratory and diagnostic test use, such as malaria or haemoglobin, will be incorporated into the algorithm if routinely available at the targeted health facilities. Before the start of the study, ePoct+ will be reviewed and validated by national key experts in child health. The clinical algorithm will not be updated for the duration of the study, except for technical reason (patches).

Training

PATH, with input from other collaborators, will develop the pulse oximetry and CDSA curricula, adapting existing materials (such as Lifebox training materials) to IMCI context using WHO and national guidelines, specifically including examples of pneumonia and possible serious bacterial infection. Materials will be pilot tested at PATH HQ, and the next iteration will be reviewed by the technical working group in each country. The feedback from countries will be incorporated into the final English version of the pulse oximetry training materials and job aids. Materials will then be translated into local languages as applicable and back translated to ensure accuracy. Training materials and job aids will be printed and

distributed to all TIMCI health facilities in draft form for the pilot phase and pending finalization after the pilot phase.

In collaboration with respective Ministries of Health (MOHs), PATH will conduct training of healthcare providers in the use of pulse oximetry and use of CDSA. To avoid creating a parallel program and improve sustainability, PATH will work with each MOH to integrate with existing training programs. WHO has updated their IMCI guidelines recently to include changes in pneumonia management and offering outpatient treatment of possible serious bacterial infection in young infants when referral is not feasible. Per WHO curricula, the trainings for these updates include clinical sessions with visits to health facilities to see real life patients, which is out of the scope of the TIMCI project. To expedite the updated IMCI training process, PATH may provide financial support to ensure that these trainings are conducted in TIMCI areas if necessary. PATH in partnership with the MOH in each country will agree on which day of the IMCI training the pulse oximetry and CDSA modules will be included. Trainings will be conducted using a "train the trainers" approach, leveraging the experience of MOH IMCI trainers. Moreover, supervisors will also be included in training sessions to equip them for their supportive supervision role.

The pulse oximetry training session will be interactive, in line with adult learning principles, and with sessions that allow hands-on practice using the device. At the end of the training session, a post-training written test and one observation of the use of the pulse oximetry per participant will be used to determine immediate acquisition of knowledge and skill. If incorrect answers or limited skills were observed, the trainer will reinforce the correct concepts and provide supportive guidance and encouragement to correct use of the device. At the end of the CDSA training session, a post-training practical test based on a set of pre-recorded cases will be used to determine immediate acquisition of knowledge and skills. Competency evaluation will be extended post-course by asking trainees to perform a certain number of consultations while using the CDSA and including a minimum number of classical IMCI conditions.

In the case of staff turnover in TIMCI facilities detected during monitoring visits, PATH in consultation with the MOH will elaborate a plan for training such new staff either at the facility or a in a group session elsewhere, depending on circumstances at the time.

Supervision

As per training, PATH will work with each MOH to integrate pulse oximetry and CDSA use into existing supervision mechanisms where possible. Supportive supervision for CDSA may additionally include personalized feedback directly informed by data recorded in the device. Intensive mentoring / supervision will be conducted following training, and then will be gradually tapered and fully transferred to government mechanisms.

Community and caregiver engagement

PATH will build on existing relationships with local civil society organisations (CSOs) and networks active in the child health space, and relevant Ministry of Health community initiatives (such as community healthcare provider networks), establishing new collaborations as needed. PATH will work closely with these CSOs and Ministries of Health to integrate information on the intervention, and content aimed at encouraging care seeking and following referral recommendations into their ongoing child health activities.

Additional information materials on pulse oximetry and CDSAs will be provided at intervention facilities, including multi-media materials or public health nurse talks, depending on preferences within each country.

6.4 Outcomes

6.4.1 **Longitudinal observational study**

Two primary outcomes will be assessed between the pre-intervention (Q1) and the last intervention period (Q5):

- Proportion of children referred by a primary care healthcare provider to a higher level of care (either to a hospital or to an inpatient part of a larger primary healthcare facility) at Day 0 consultation
- Proportion of children prescribed an antibiotic at Day 0 primary care consultation

These two outcomes are selected to reflect the intended impact on referral of children with severe disease (pulse oximetry and CDSA) and antimicrobial stewardship (CDSA). Severe complication outcomes are included in secondary outcomes rather than primary as, given the pre-post nature of the study, change in mortality / morbidity outcomes could be influenced by other events occurring between the baseline and post-intervention comparison phase.

Secondary outcomes:

- Proportion of children with a severe complication (death or secondary hospitalisation) by Day 7
    - Secondary hospitalisation refers to any delayed hospitalisation (occurring at any point greater than 24 hours after Day 0 consultation) and any hospitalisation occurring without referral. The denominator is all enrolled children
- Proportion of children admitted to hospital within 24 hours of the Day 0 primary care consultation and as a result of a referral
    - This is used as a proxy for 'appropriate referral' of children, as those with severe disease should generally be admitted to hospital. The denominator for this outcome is also all children enrolled in the study, rather than only referred children. This is because the proportion of referred children that are admitted may be high in routine care, in the context of an inappropriately low referral

rate. The aim of the intervention is therefore to increase the overall referral rate of children with severe disease. Hospital admission is chosen as the proxy of severe disease rather than using primary care classification of severe disease, as there are inadequacies in the classification of severe disease in routine practice

- Proportion of children completing referral to a higher level of care within 24 hours, of all children referred at Day 0 consultation
- Proportion of children cured (defined as caregiver reported recovery from illness) by Day 7

Sub-groups for analysis of primary and secondary outcomes:

- Age (under 2 months, 2 – 12 months, 13 – 59 months)
- Sex
- Presentation with cough / difficulty breathing

Other outcomes of interest – hypoxaemia

- Proportion of children with severe (SpO_2 <90%), moderate (SpO_2 90 – 91%) and mild hypoxaemia (SpO_2 92 – 93%), adjusted for sites at high altitude
- Proportion of children with hypoxaemia (according to differing cut-offs) with severe complication
- Proportion of children with severe hypoxaemia not meeting any other clinical criteria for severe disease
- Proportion of children referred with hypoxaemia who receive oxygen at hospital

Other outcomes of interest – referral and follow-up

- Proportion of children with non-severe disease referred to a higher level of care on Day 0
- Average length of stay (in days) of children admitted to hospital
- Proportion of children attending scheduled follow-up at the same facility by Day 7
- Proportion of children presenting for unscheduled follow-up to any health facility by Day 7

Other outcomes of interest – antimicrobial prescription

- Proportion of children prescribed a diagnosis-appropriate antibiotic
- Proportion of febrile children tested for malaria at Day 0
- Proportion of malaria positive children prescribed an antimalarial
- Proportion of malaria negative children prescribed an antimalarial
- Proportion of untested children prescribed an antimalarial

Other exploratory analyses:

- Additional sociodemographic characteristics: urban / rural location, distance from health facility, key household indicators such as maternal age and education
- Additional complaint / symptom categories (fever, diarrhoea and other) and time since onset of symptoms
- Alternative hypoxaemia cut-offs
- Healthcare provider severity classification
- Facility and health system factors – type of facility, healthcare provider qualification and previous training
- Time point (Q1, Q2, Q3, Q4, Q5) and pilot site data

6.4.2 **Other mixed methods studies**

These studies will mostly be exploratory, particularly the qualitative studies, which seek to understand perceptions, values, attitudes and beliefs. Process evaluation criteria will be evaluated according to the MRC criteria.[35]

Service provision assessments, at baseline and each quarter during implementation, will assess: adherence to IMCI (or national guidelines) and device guidelines (during intervention phase), including proportion of: caregivers asked about all three danger signs; caregivers asked about all main symptoms; children with cough or difficulty breathing in whom a respiratory rate is counted for a minute and (where applicable) in whom a pulse oximetry reading is attempted, conducted correctly, obtained at first attempt or at repeated attempts; and proportion of children in whom CDSA is used and recommendations are adhered to. Furthermore, correctness of classification and treatment (as far as is possible with available information), and other indices of quality of care will be assessed. Facility and healthcare provider factors associated with health and quality of care impact and intervention uptake will be assessed.

Facility-based process maps and associated non-participant observational data will be described and compared between baseline and early and late intervention periods. Time (in minutes) will be compared (pre, early and late intervention) for total in clinic; in consultation; and on use of devices, and any other relevant single steps of the care process. Comparison will be made using mean (if parametric) or median (if non-parametric).

Qualitative enquiry with healthcare providers and caregivers will primarily focus on service provision, the participant response to the intervention and acceptability of devices, with exploration of change in perceptions, attitudes and behaviour over time. With caregivers, we will also explore experience of care and care-seeking behaviour through IDIs and walking interviews.

The sub-study of stakeholder perceptions, surveys and project data review will primarily focus on the processes, mechanisms and context (i.e. process evaluation criteria with focus on fidelity, dose, reach, context and adaptation) of implementation including adaptations made.

6.4.3 Cost and modelled cost-effectiveness study

- Full implementation cost of the intervention per focal country (cost to health system as well as direct costs paid by the household to the health system)
- Cost per child assessed using pulse oximetry devices and CDSA per country compared to without these supports.
- Cost per DALY averted attributed to the introduction of pulse oximetry both without and with CDSAs across focal countries. This will be modelled comparing proportions referred to hospital on day zero or by day 7 or requiring secondary hospitalization by day 28 in each arm and the predicted outcomes in each case, using published literature supplemented where necessary by expert opinion. We will also utilize data on any other severe outcomes, and also use medical indicators on children who are admitted to hospital to try to assess level of severity of illness.

6.5 Participant timeline

6.5.1 Longitudinal observational study

Following informed consent and enrolment, research assistants (RAs) will record participant name, address and contact details (including personal and alternate contacts) for Day 7 follow-up, basic sociodemographic details, reason for attendance and care-seeking. This will be collected from the caregiver before consultation, except in critical / emergency cases who will be seen immediately by the healthcare provider. The clinical consultation will be conducted by healthcare providers, who in the post-intervention phase will have been advised to use the devices as described above.

After the consultation, the RA will use extract information from the clinical records from the caregiver (child health card and / or referral note), and / or from clinic ledgers or medical records. Information will be extracted and recorded on the final diagnosis, oxygen saturation and results of laboratory investigations (if performed), prescribed medication with dosage, referral or follow-up advice and the usage of the pulse oximeter device and/or CDSA (if applicable). Research assistants will seek clarification from healthcare providers in cases where the diagnosis is not clear from the records.

Follow-up at Day 7 will be conducted by phone by an RA. If phone follow-up fails, three attempts will be made on the subsequent three days to reach the caregiver. Community mechanisms to facilitate follow-up (such as arranging a call with a community health worker) will be used, with consent, for caregivers without access to a phone. A structured questionnaire will be administered to assess the primary and secondary outcomes, including details of any government or private hospital admission. In case of non-recovery, if the child is still at home, the study team will advise the family to return to the PHC clinic for a follow-up consultation.

Additionally, visit information from government-designated hospital records will be collected retrospectively for children whose caregiver reported at Day 7 phone follow-up that they attended hospital, including clinical assessment on arrival (vital signs, admitting diagnosis), admission details (duration, if oxygen was administered, intensive care admission) and admission outcome (final diagnosis, discharge against medical advice, death). The same data will be collected for children who attended a non-hospital higher-level of care (emergency or admission unit of a primary or secondary care facility).

It is expected that some participants may present at the same or another health facility enrolled in the study during the follow-up period. These visits and the findings will be recorded on the appropriate scheduled / unscheduled visit eCRFs.

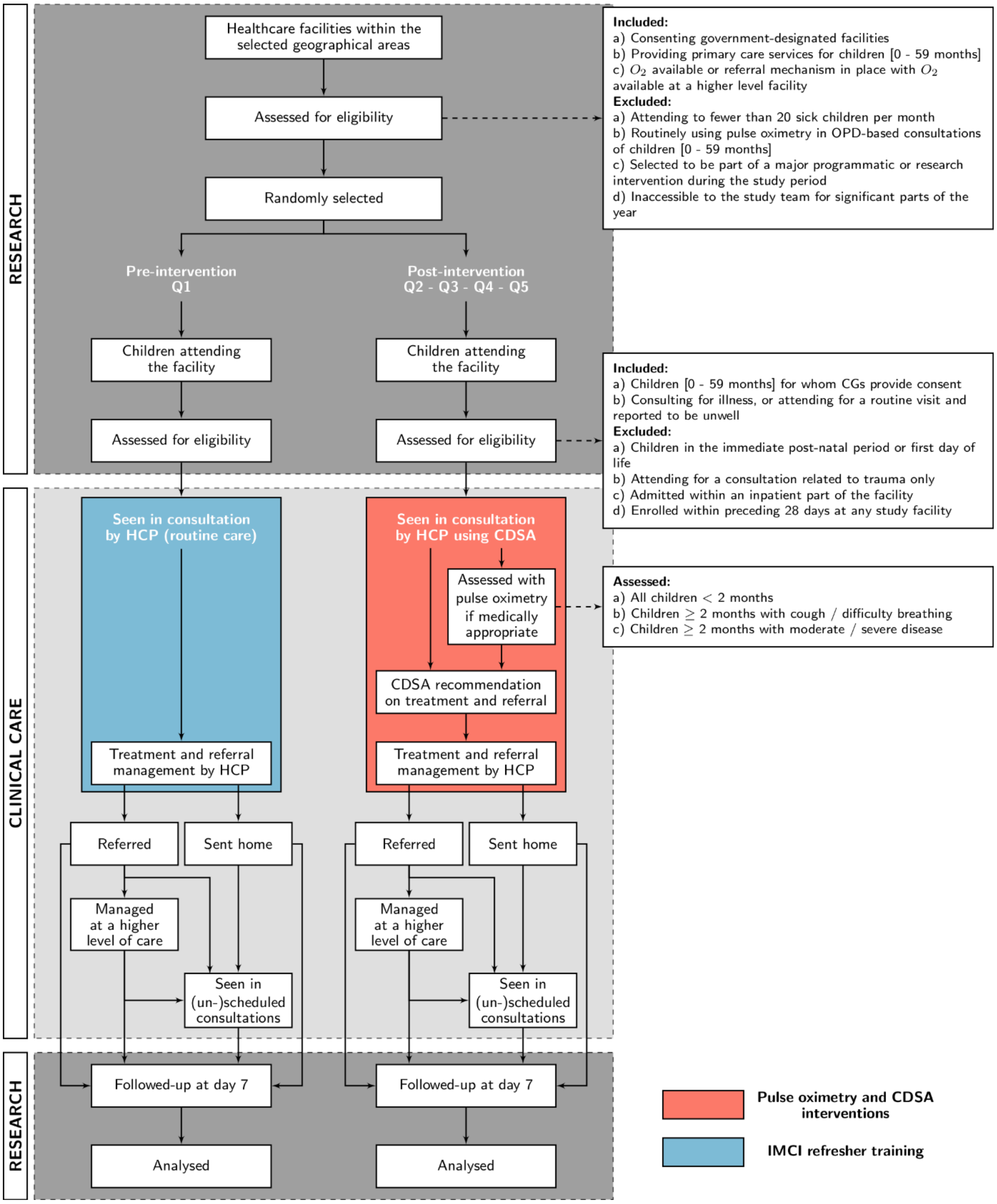

Fig. 5. Flowchart of the longitudinal observational study. CDSA = Clinical Decision Support Algorithm. CG = Caregiver. HCP = Healthcare provider. OPD = Out-patient department.

Tab. 4. Schedule of the visits and associated CRFs for the longitudinal observational study. The different types of staff collecting data are represented by the following icons:

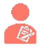 Facility-based research assistants

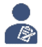 Research assistant rotating between different referral health facilities

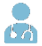 Healthcare providers as part of intervention clinical routine

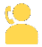 Call centre-based research assistant

			**Health facility baseline visit**		**Higher level of care visit[1,2]**	**Follow-up or unscheduled visit at enrolled health facility**		**Phone follow-up**
		**Time point**	Day 0		Day 0-7	Day 1 -7		Day 7 [7 - 10]
		**Data collection method**	Prospective data collection		Review of medical records	Prospective data collection		Phone interviews
		**Study phase**	Pre	Post[3]		Pre	Post[3]	
**Events**		**Forms**						
**Informed consent**		-	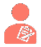	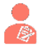				
**Screening**		TIMCI-01	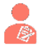	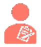				
**Identification & contact information**	Full identification	TIMCI-02a	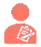	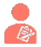				
	Contact information	TIMCI-02b	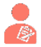	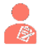				
	Identification and contact information check	TIMCI-02c				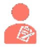	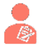	
	Referral facility identification	TIMCI-02d			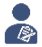			
	Follow-up identification check	TIMCI-02e						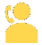
**Socio-demographics**		TIMCI-03	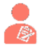	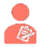				
**Visit info**	Care seeking behaviour, transport and costs for visiting health facility reported by caregiver	TIMCI-04a	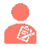	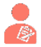				
	Visit and admission info recorded by referral facility (medical records, registries)[2]	TIMCI-04b			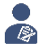			
	Patient journey reported by caregiver at follow-up	TIMCI-04c						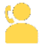
**Clinical presentation & medical history**	Clinical presentation and medical history reported by caregiver at baseline	TIMCI-05a	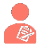	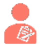		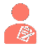	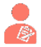	
	Medical history recorded in clinical notes and / or facility registries	TIMCI-05b	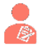	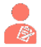		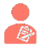	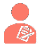	
	Chief complaints and medical history recorded in CDSA	TIMCI-05c		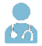			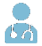	

**Basic measurements & physical exams**	Basic measurements and physical exams recorded in clinical notes and / or facility registries	TIMCI-06a	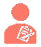	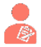		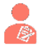	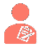	
	Basic measurements and physical exams recorded in CDSA	TIMCI-06b		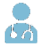			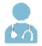	
**Respiratory rate & pulse oximetry**	Respiratory rate and pulse oximetry recorded in clinical notes and/or facility registries[2]	TIMCI-07a		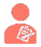			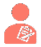	
	Respiratory rate and pulse oximetry recorded in CDSA[2]	TIMCI-07b		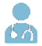			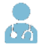	
	Respiratory rate and oxygen saturation recorded at arrival by referral facility[2]	TIMCI-07c			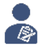			
**Laboratory investigations**	Laboratory investigations recorded in clinical notes and / or facility registries[2]	TIMCI-08a	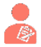	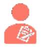		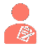	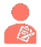	
	Laboratory investigations recorded in CDSA[2]	TIMCI-08b		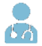			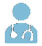	
**Diagnosis & care management**	Diagnosis and care management in clinical notes and / or facility registries (medical records, discharge summaries)	TIMCI-09a	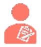	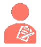		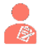	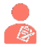	
	Diagnosis and care management recorded in CDSA	TIMCI-09b		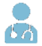			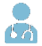	
	Diagnosis and care management recorded by referral facility (medical records, discharge summaries)	TIMCI-09c			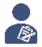			
**Follow-up at Day 7**		TIMCI-10						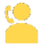

[1] Data will be collected retrospectively for visits at facilities offering a higher level of care, including district hospitals, any study facility receiving referrals or admitting patients.
[2] If appropriate for clinical management.
[3] The schedule assumes the intervention includes pulse oximetry plus CDSA.

6.5.2 Other mixed methods studies

An overview of the timeline for participants recruited to the mixed methods studies and how they are embedded into the longitudinal observational study is shown in **Fig. 6**. In the sub-sample of children (and their caregivers) selected as part of the SPA, two additional time-points of data collection will occur – during the consultation (observation), and after their clinical consultation (for the exit interview).

Walking interviews will be carried out with caregivers as they leave the facility, accompanying them on their continued care seeking path. IDIs and FGDs with caregivers will take place after the day 7 follow-up phone call. Healthcare providers recruited for qualitative interviews or FGDs will be invited for an interview/ FGD from SPA and/or non-SPA facilities. The final selection of stakeholders (outlined in Section 5.7) will be determined during implementation and based on implementation experience.

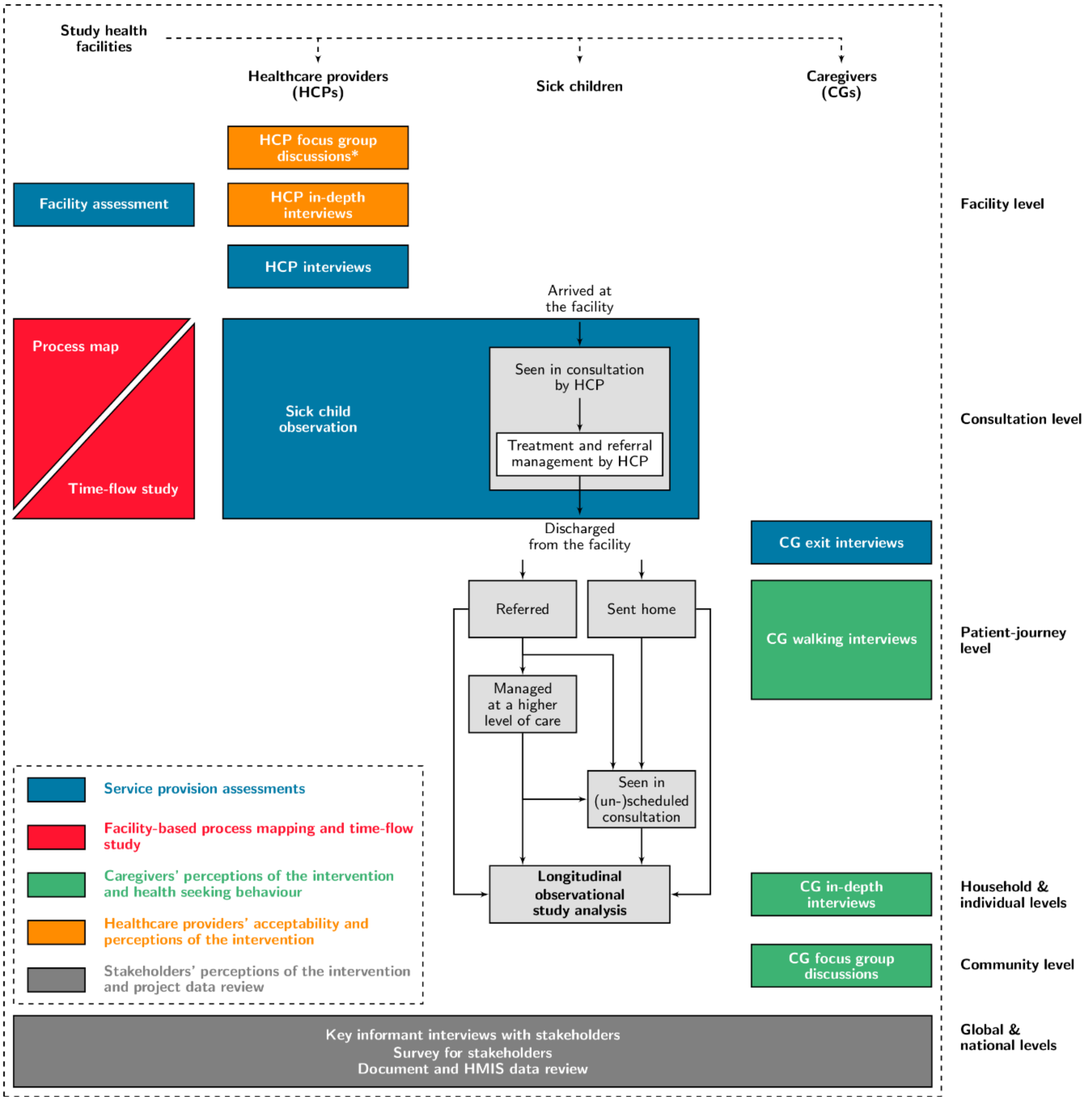

Fig. 6. Overview of the mixed methods studies and how they are embedded within the longitudinal observational study in Kenya, Myanmar and Senegal. CG = Caregiver. HCP = Healthcare provider. Healthcare provider focus group discussions will group healthcare providers from different facilities.

6.5.3 Cost and modelled cost-effectiveness study

Facility-based medical and non-medical personnel who provide information for costing will be approached at a time convenient for them so as not to interfere with clinical or health service delivery. Information will be provided about the study and consent will be sought for personal data as outlined in 9.3.3. These providers will be approached pre-intervention and then again during intervention for data collection, though different providers may be approached in case of staff rotation.

6.6 Sample size

The estimated sample sizes for each of the studies are described below, but may be revised during the course of the study. In the case of the longitudinal study, this is due to uncertainty of some of the assumptions (such as eligibility rate and outcome proportions in routine care, for which data reliability is a challenge). Final qualitative sample sizes will be determined based on reaching thematic saturation, i.e. when no new concepts are emerging.

The remaining sample sizes, for SPA, process mapping and time-flow study, have been chosen pragmatically and will vary according to country depending on the degree of difference in care processes between facility types and location, whilst allowing for exploratory analysis into more in-depth aspects of quality of care from baseline to intervention and over time within the intervention period (both due to changes in implementation uptake and seasonal variation in burden of disease).

The sample size for the pilot phase will also be pragmatically based on the degree of difference between facility types and location (and thus the potential need to understand and refine the intervention strategy according to context), but is likely to include between 2 and 6 facilities per country.

6.6.1 Longitudinal observational study

The sample size for the longitudinal observational study is calculated separately for each country, based on comparison between Q1 and Q5, however the same sample size will also be collected in each of the intervening quarters (Q2, Q3 and Q4) for the purposes of secondary analysis over time (the sample may be smaller for Q2 – Q4 in Myanmar for logistical reasons). The following assumptions are used:

- power 80%
- alpha 0.05

- pre-intervention referral to a higher level of care 3% (based on informal estimates from country research teams and SPA data from various countries), with a minimum detectable difference of 50%
    - We have chosen a large detectable difference for referral from primary care as not all children referred will complete referral, i.e. a 50% increase in referral rate may only result in a 20 – 30% total increase in completed referrals (this will be assessed as a secondary outcome and is therefore not a component of the sample size calculation)
- pre-intervention antibiotic prescription of 60% (based on informal estimates from countries, SPA data and other observational studies)
- intra-cluster correlation coefficient (ICC) of 0.005 for referral and 0.05 for antimicrobial prescription
- Kenya – estimated average cluster size of 230 children / month (assuming a cluster ratio of health centres : dispensaries 1:2, with 100 / month in dispensaries and 500 / month in health centres
- Myanmar – estimated cluster size of 96 children per month (based on informal estimates from Burnet Institute and PATH Myanmar), assuming a ratio of RHC : sub-centre of 1:1
- Senegal – estimated cluster size of 170 children per month (based on DHIS2 data on outpatient consultations in Thies in 2019)

For the referral primary outcome, we therefore require the following sample size:

- Kenya – 17 facilities, recruiting an average of 690 children per quarter per facility, equating to 11,730 children per quarter
- Myanmar – 20 facilities, recruiting an average of 338 children per quarter per facility, equating to 6,760 children per quarter
- Senegal – 18 facilities, recruiting an average of 510 children per quarter per facility, equating to 9,180 children per quarter

For the antibiotic prescription primary outcome, this would enable a minimum detectable reduction of 18% in Kenya and Senegal and of 16% in Myanmar.

6.6.2 Mixed methods studies

We will conduct the assessment in 8 – 10 longitudinal study facilities, stratified by rural / urban location and facility type, at 5 time points – pre-intervention, and each quarter over the 12 months of the post-intervention period. We will also conduct the SPA in the pilot facilities to inform intervention refinement.

At each facility at each time point, observation and exit interview assessments will be conducted over 3 – 10 working days, in order to achieve a sample size of 10 – 30 children per facility. In smaller facilities with only one provider, the target will be a minimum of 10 observations and exit interviews, whereas in larger facilities, the target will be a minimum of 20 observations and exit interviews with at least 5 for any single provider. This would result in an estimated sample size of 800 – 1000 observations over the study period. Interviews will be conducted with all consenting healthcare providers responsible for the care of sick children at the study facility during the first assessment. These will only be repeated if details have changed (e.g. training updates), or for new staff not present at the first assessment. We estimate around 20 – 40 healthcare provider interviews (depending on facility size). The full facility assessment will be done at the first visit, and only variables which change over time will be recollected at subsequent visits.

We will conduct facility-based process mapping and time-flow assessments in each of the SPA facilities. One process map (and associated notes on the care process and its rationale or deviations) will be developed per facility during baseline, which will be revisited and revised during early and late intervention phases to reflect changes over time, resulting in 3 maps per facility. Process maps will also be developed in the pilot facilities to inform intervention refinement. Each map will involve informal discussion with 2 – 4 healthcare providers in the facility, and non-participant observation over a period of one week, during which time-flow data will also be collected (for an estimated 10 – 30 children depending on facility size).

IDIs with caregivers: A purposive sample of caregivers will be selected for IDIs including those who completed referral (and arrived in the hospital within 24 hours) as well as those that did not adhere to the advice, and those that were not referred. Within these sub-categories, participants will be purposively selected with varying socio-demographic characteristics such as rural / urban location, age, and education. An estimated total of 35-45 IDIs with caregivers in each country throughout the entire project are expected. Approximately 10 – 12 in-depth interviews with caregivers will be conducted across different time points (pilot, early, mid and late intervention) within one geographical area of each country. However, if geographical variation in health-seeking behaviour and outcomes is felt to be significant, fewer time points may be selected in favour of greater geographical diversity. In Kenya and Senegal only, a further 10-12 IDIs will also take place in the pre-intervention phase. Caregivers for IDIs will be different from FGD participants.

FGDs with caregivers: One geographical area will be selected per country, in which a total of 6 FGDs (3 in urban and 3 in rural location) will be conducted, each with 8 – 12 participants. These will be divided between early and late intervention. Caregivers for FGDs will be different from IDI participants. Where possible participants will be divided by age groups and gender as well as number of children.

Walking interviews with caregivers: A purposive sample of 6 – 8 caregivers per country, having received referral advice for their sick child, will be selected towards the end of the intervention

period. Care givers may be followed through (to IDI) if of interest; otherwise caregivers for walking interviews will be different for IDIs and FGDs.

IDIs with healthcare providers: An estimated total of 35– 45 IDIs during the intervention phase per country is expected, allowing to document change over time. This will include approximately 10 – 12 IDIs with healthcare providers at different time points (pilot, early, mid, late intervention), purposively selected from facilities with divergent implementation. In Kenya and Senegal only, a further 10-12 IDIs will also take place in the pre-intervention phase. Where possible, some healthcare providers will be interviewed repeatedly over time.

A total of 6 FGDs with healthcare providers will be conducted per country, with 6 – 8 participants per group. Healthcare providers at both, SPA and non-SPA-facilities may be invited, with purposive sampling of early and late adopters at a later stage of data collection during the intervention. Where possible healthcare providers from different cadres will be separated. The FGDs with healthcare providers will be split between early and late intervention.

KIIs with stakeholders: A total of approximately 45 key-informant interviews during the pilot and intervention phase will be carried out. This will include approximately 3-6 KIIs at global and 10-12 KIIs at national level at different time points (between pilot, early and late stages) of the project. In Kenya and Senegal only, a further 10-12 IDIs will also take place in the pre-intervention phase. The final selection of type and number of different types of stakeholders (outlined in Section 5.7) for the intervention phase will be determined during implementation and based on implementation experience.

6.6.3 Cost and modelled cost-effectiveness study

Cost data will be collected from eight facilities per country (4 urban and 4 rural), and in countries where there are big size differences among health facilities, we aim to include four larger and four smaller facilities. At each facility, the most senior medical professional and the most senior administrative professional available will be interviewed to obtain the majority of the unit cost data, and the senior medical staff member to obtain information on time allocation of front-line staff (time seeing patients, time in supervision etc).

6.7 Recruitment

To reach the required sample size, and to ensure data collection across different seasons, it is estimated that recruitment will take place over 15 months. As we intend to validate eligibility and recruitment rate estimates in the pre-intervention phase, we may either recruit additional facilities or slightly increase the study duration if necessary. Participants will be recruited during working hours, Monday to Friday, though evenings and weekends may be included if high volumes of patients attend at those times (depending on country and facility type).

Child and caregiver participants will be recruited for the SPA and time-flow observations on the relevant study assessment days consecutively until the sample size requirements detailed above have been met. Children and their caregivers will be enrolled to these studies at the

same time as they are enrolled to the longitudinal study. Longer assessment periods will be required for low-volume facilities. All healthcare providers providing care for children under 5 years of age and present at facilities during SPA assessments will be invited to participated in the SPA interview.

Participants in the qualitative studies will be recruited as follows:

A qualitative researcher (possibly assisted by the research assistant based at each study facility for the longitudinal study) will approach caregivers of referred children directly after consultation for walking interviews. A range of facilities will be visited, but if recruitment is challenging due to low referral numbers, only high-volume facilities will be visited.

Caregivers for IDIs and FGDs will be selected based on pre-defined queries of the longitudinal database after Day 7 follow-up has occurred in order to identify potential participants within a selected geographical area. A list will be generated for the qualitative researcher with basic socio-demographic details (divided into referred and not referred for IDIs) of double the number of participants required (to allow for unreachable participants or refusals to participate) for the time point in question. The qualitative researcher will then contact participants by phone to arrange IDIs or FGDs. If recruitment with this strategy proves challenging, individuals may be approached directly at facilities to arrange a follow-up in the community after 7 days or at the community level (i.e. by a community health worker).

Health care providers from study facilities will be invited to participate in the studies by the qualitative researcher. Some of the same healthcare providers invited to participate in the FGDs and IDIs will be re-invited over time. A pre-defined list of variables from the longitudinal study will be used to classify healthcare providers into early and late adopters of the intervention; and change in uptake over time. This will help to inform the focus of the qualitative inquiry over the implementation period. However if the recruitment process proves challenging due to staff rotation, clinical commitments or research fatigue, a broader range of healthcare providers will be approached from both SPA and non-SPA facilities.

Recruitment of healthcare providers in the pre-intervention / pilot / early intervention phase: Where only one healthcare provider is present at the facility s/he will be invited. If more than one healthcare provider is working at the same facility a random selection will be made, based on a variety of sociodemographic factors to ensure a diverse sample (e.g. including age, gender, professional background, years of work experience).

Recruitment of healthcare providers in the mid/late intervention: Healthcare providers will be purposively selected from facilities with divergent implementation. Divergent implementation will be identified as described above.

Stakeholders will be purposively selected with a focus on a diverse sample presenting the different levels of implementation (global, national, regional and local level) as well as their level of direct involvement in the project. Stakeholder mapping will be conducted to identify potential participants internal and external to the project. All pre-selected key stakeholders will

be invited by the research assistant to participate in the study. The same respondents will be invited over time. If the recruitment process proves challenging (e.g. due to staff rotation) the same principles as with healthcare providers apply.

7 Data collection, management and analysis

The data used within the TIMCI project are described in **Tab. 5**. Primary research data will be generated in study and pilot facilities through case report forms, questionnaires, interviews, focus group discussions and surveys. In addition, relevant routine data will be extracted for all intervention facilities (i.e. including those in which primary research data will not be collected). This will include individual consultation data recorded in the CDSA for clinical purposes and aggregate facility data that are reported to the national HMIS.

Tab. 5. Summary of the data used within the TIMCI project. CDSA = Clinical Decision Support Algorithm. MedAL-R is the front-end application developed by Unisanté/Wavemind (Lausanne, Switzerland) to implement ePoct+, the clinical algorithm. EDC = Electronic Data Capture. FGD = Focus Group Discussion. HMIS = Health Management Information System. IDI = In-Depth Interview. KII = Key Informant Interview. SARA = Service Availability and Readiness Assessment.

Ref	Methodology	Data collection	Tool	Data format	Data origin	Data classification	Data reuse
**1.1**	**Routine data**	CDSA individual consultation data	MedAL-R with ePoct+	Textual, numerical, categorical	Primary	Sensitive personal	1.2<br>2.5
		Aggregate facility data	National HMIS	Textual, numerical, categorical	Secondary	Non-personal	1.2<br>2.1<br>2.5
**1.2**	**Longitudinal observational study**	Quasi-experimental longitudinal observational study	Electronic case report forms (EDC platform)	Textual, numerical, categorical	Primary	Sensitive personal	2.1<br>2.2<br>2.3<br>2.4<br>2.5
**2.1**	**Service provision assessment**	Modified demographic and health survey	Facility assessment (modified SARA questionnaire)	Textual, numerical, categorical	Primary	Non-personal	2.5
			Healthcare provider interview	Textual, numerical, categorical	Primary	Sensitive personal	2.5
			Sick child observation protocol	Textual, numerical, categorical	Primary	Sensitive personal	2.5
			Structured exit interview	Textual, numerical, categorical	Primary	Sensitive personal	2.5
**2.2**	**Facility-based process mapping and time-flow study**	Process map	Process map tool	Abstract	Primary	Non-personal	2.5
		Time-flow study	Time-flow tool	Textual, numerical, categorical	Primary	Sensitive personal	2.5<br>3.1
**2.3**	**Healthcare providers' acceptability and perceptions of the intervention**	Semi-structured in-depth interviews	IDI tool for healthcare providers	Audio, Textual, numerical, categorical	Primary	Sensitive personal	2.5
		Focus group discussions	FGD tool for healthcare providers	Audio Textual, numerical, categorical	Primary	Sensitive personal	2.5

**2.4**	**Caregivers' perceptions of the intervention and health-seeking behaviour**	Semi-structured in-depth interviews	IDI tool for caregivers	Multimedia (audio, drawings), Textual Numerica	Primary	Sensitive personal	2.5
		Focus group discussions	FGD tool for caregivers	Multimedia (audio, video-/photo-documented), Textual, numerical, categorical	Primary	Sensitive personal	2.5
		Walking interviews	Walking interview tool	Audio GPS tracking Textual, numerical, categorical	Primary	Sensitive personal	2.5
**2.5**	**Stakeholder's perceptions and project data review**	Key informant interviews	KII tool	Audio Textual, numerical, categorical	Primary	Sensitive personal	2.5
		Survey for stakeholders	Online survey tool	Textual, numerical, categorical	Primary	Sensitive personal	2.5
		Project data review	Document review matrix	Textual	Secondary	Non-personal	N/A
**3.1**	**Cost and modelled cost-effectiveness**	Cost analysis	Medical personnel questionnaire	Textual, numerical, categorical	Primary	Sensitive personal	2.5 3.1
			Non-medical personnel questionnaire	Textual, numerical, categorical	Primary	Sensitive personal	2.5 3.1
			Hospital costs	Textual, numerical, categorical	Primary	Non-personal	2.5 3.1
		Modelled cost-effectiveness			Secondary	Non-personal	2.5

7.1 Data collection methods

Quantitative research data for the longitudinal observational and mixed-method studies will be collected using a secure third-party cloud based electronic data capture (EDC) platform that meets research / ethical standards. CDSA routine individual consultation data will be extracted from MedAL-R, a software developed by Unisanté/Wavemind (Lausanne, Switzerland) to implement the clinical algorithm (ePoct+), while HMIS aggregate facility data will be extracted from the national HMIS, following MoH approval. Qualitative research data will be collected using both EDC, audio recordings, photo documentation and video recordings (in the case of method acting) and hand-written paper notes and drawings. GPS data will be collected using a mobile device.

7.1.1 Longitudinal observational study

For each participant enrolled in the longitudinal observational study, data will be captured during the pre- and post-intervention periods according to the different assessments and Case Report Forms (CRFs) described in the visit schedule displayed in **Tab. 4**. A description of the variables collected in each CRF is available in the appendix.

Health facility visits

Quantitative research assistants will use an EDC mobile application to enter the research data of enrolled participants. The quantitative research assistant will collect from the caregiver screening information, information on care seeking behaviour, transport and costs, and clinical presentation and medical history. A number of in-depth sociodemographic and household variables will also be collected either as surrogate measures of household wealth, or known to be associated with health-seeking behaviour and / or child mortality (including maternal education). Household wealth indicators were selected from DHS and other surveys in the study countries as those which best estimate wealth index that can be directly collected from the mother / caregiver and with a reasonable time vs. reliability trade-off. Data on laboratory investigations (if performed), final diagnosis, prescribed medication, referral or follow-up advice will be extracted from clinical notes and / or facility registers. Quantitative research assistants will seek clarification from healthcare providers only in cases where these variables are not clear from the records. Personally identifiable information (PII), including participant's name and date of birth, as well as caregiver's phone number and physical address, will be collected for linking data and detecting possible duplicates and carrying out the study follow-up.

During the post-intervention period, the research data of participants enrolled in the study will be linked with their CDSA consultation data. Additional information on oxygen saturation will be collected as recorded in clinical notes and / or facility registers. Pulse oximeter device savings will be cross-checked by quantitative research assistants at the end of the consultation where possible.

Follow-up

Quantitative research assistants will conduct the phone follow-up interviews using an EDC mobile application. Personally identifiable information (PII) will be used for contacting caregivers. A window of +3 days (Day 7 to Day 10) applies to Day 7.

Data that will be collected at Day 7 follow-up will include cure status (recovery from illness as reported by the caregiver), hospitalisation and death between Day 0 and Day 7. If appropriate, name of the referral facility and duration of hospitalization will be collected to facilitate the retrospective data collection at referral hospital level.

Higher level of care visit

Data collection at this level of care will be conducted retrospectively. A quantitative research assistant using the EDC mobile application will collect data from referral facility records, registries and discharge summaries for children whose caregiver reported at Day 7 phone follow-up that they attended a government-designated referral facility between Day 0 and Day 7. This includes attendance to hospital or emergency / inpatient area of a larger primary or secondary care facility (e.g., study facilities that can admit paediatric patients). Personally identifiable information will be collected to cross-check the identity of the patients. Arrival date and time, basic clinical information on arrival and, if admitted, admission and discharge diagnosis and admission duration will be collected. If available, data on oxygen saturation at

admission, oxygen administration and intensive care unit (ICU) admission and duration will also be recorded.

7.1.2 Routine data

CDSA individual consultation data

Routine individual consultation data will be recorded in the CDSA in all intervention facilities in the post-intervention period. As part of the intervention, healthcare providers will be asked to use MedAL-R during their consultations. When they do so, they will routinely enter all data necessary for the clinical management of children 0 – 59 months of age into MedAL-R. Data used within TIMCI will cover clinical presentation (including symptoms and dangers signs), medical history (including HIV and TB status if known), all physical exams and measurements relevant for clinical assessment (including pulse oximetry), and laboratory investigations. Automated recommendations from CDSA on diagnosis and child management will also be collected, as well as healthcare provider's agreement or disagreement with these recommendations and possible additional diagnoses and treatments, which will be manually entered. A description of the variables extracted from CDSA is available in the appendix.

HMIS aggregate facility data

HMIS data will include the total number of outpatient consultations – for all patients and for children 0 – 59 months of age (disaggregated by new and follow-up visit if possible) –, disease burden, and any other relevant indicators if available (such as referral rate or mortality rate), disaggregated by age and sex if routinely collected with this granularity.

7.1.3 Other mixed methods studies

Some data required for the SPA (observation and caregiver exit interview) are common with the longitudinal observational study and will not be collected again. To reconstruct the full SPA dataset, SPA observation and caregiver exit interviews will be linked with the longitudinal observational study as described in Section 9.4.3. The consultation observation will be conducted by a standardised clinical observer who will record information asked or provided by the healthcare provider and / or caregiver during the consultation on key aspects relating to adherence to IMCI (or national guidelines) and the use of pulse oximetry and CDSA (post intervention). The observer will not ask any questions directly of the caregiver, but at the end of the consultation will ask a limited number of questions of the healthcare provider to record diagnosis and management. The exit interview will be performed by a non-clinical research assistant who will record responses to questions on additional sociodemographic and clinical details and experience of care. The healthcare provider interviews will collect information from healthcare providers involved in the care of children 0 – 59 months, on qualifications, in-service training and experience related to child health and pulse oximetry and experience of the working environment. The facility assessment will focus on the basic infrastructure, staffing, services, diagnostics and medicines relevant to child health, with additional information collected on pulse oximetry, records and reporting and management. At the end of the SPA

assessment at the facility, the clinical observer and research assistant will note down additional (prompted) summary observations about the use of pulse oximetry and / or CDSA in the facility.

For the process mapping and time-flow study, pre-drawn, country-specific process maps will be reviewed and modified by research assistants with informal input from facility staff and observation. Time data will be recorded using an individual follow-through (timing one child from arrival to exit) and/or area-based (waiting area timing, consultation timing, further diagnostic timing, etc.) approach. Notes based on non-participant observation will be taken by research assistants on observations within the facility (e.g. if devices are not charged, waiting rooms are crowded, the type of interactions between patients and providers, change in provision of care (e.g. new triage system introduced)) and will not include any individual patient data.

FGDs will be facilitated by a moderator and have an assigned note taker (in charge of documentation). Prior to moving to the main topics of discussion, ice-breaker exercises will be used to establish trust with participants and to provide an overview of the topic for discussion (pulse oximetry and CDSA). The classical FGDs with healthcare providers (and possibly caregivers) will use clinical vignettes which will be presented and jointly discussed.

In the method acting FGDs, participants will first be asked key questions about their last visit with their sick child at an intervention facility (i.e. verbalized senses and experiences); the moderator will create a discussion among participants. In a next step, participants will be asked to recall their last visit and choose a pantomimic position (i.e. embodied images) when thinking about the consultation and the devices in use. The group facilitator will ask each participant to share their story on why they have chosen the specific position. As a last step, participants divided into two groups will then be asked to develop a role play of a realistic visit at the health facility with a sick child from arrival until departure incorporating interactions with different facility staff members (i.e. performance). Small scenes will be presented by each group and the scenario documented. The role plays will be jointly discussed during the development and after the performance, exploring rationale for choosing specific roles, dialogue and behaviour, with exploration of relation of these to lived experiences. The FGD will be photo-documented or video-taped (if all participants have provided consent, if some participants have not provided consent they will be taken out of shot; if none have consented to video/photo-documentation the session will only be audio-recorded and described in notes).

Qualitative data from IDIs, walking interviews and KIIs will be collected by trained qualitative research assistants using a voice recorder and a mobile phone (or similar device) for GPS tracking during walking interviews, in addition to paper-based notes and drawings and a tablet for quantitative data collection. Interviews will be conducted using a semi-structured guideline interview tool, with emphasis placed on building trust and using open-ended questions with probes. Walking interviews will be based on an explorative theme catalogue.

Key-informant interviews with stakeholders will either be conducted in-person or via telephone/Skype/Zoom. Interviews will be scheduled and interview questions shared in

advance if wished. Surveys (using an online survey tool such as Survey Monkey) for selected stakeholders will be sent out on a regular basis (quarterly) via email. Participants will be asked to answer the questions and upload additional documents. A single reminder will be sent in case no response was received. Documents will be requested from partners on a quarterly basis; a desk-review will be conducted and data entered into a document matrix.

7.1.4 Cost and modelled cost-effectiveness study

Cost data collection will occur pre- and during intervention as shown in **Fig. 3**.

Definition of costs using an activity-based approach

An activity-based approach will be explored using available cost data. Specific activities in the intervention will be allocated to different cost centres. In order to provide cost estimates that will be as managerially-, policy- and programmatically-relevant as possible, the cost analysis will adopt this approach as this separate research costs from the main programme implementation costs. The focus will be on those costs that are expected to change with the introduction of pulse oximetry and CDSAs. Costs including utilities and large equipment costs will remain the same with or without the introduction of pulse oximetry and CDSAs.

To apply the activity-based approach, we will identify specific activities that are to be costed. These will be referred to as "cost centres" (See **Tab. 6**). Cost centres are a way in which the resources used to produce the intervention activities—and the costs of those resources—are grouped. Cost centres are defined in such a way that:

- The sum of the cost centres is comprehensive—including all the resources used to produce each and every activity of the program—and together, the entire program, and
- They are mutually exclusive—thus avoiding double counting any of the resources used to provide the program.

It is important to note that this cost methodology is not comprehensive. As described in 5.8, it does not include the indirect costs incurred by the consumer (patient) e.g. the caregiver's time or transport costs. Rather, it focuses exclusively on the provider's direct costs, whether borne by the health system or paid out of pocket by the patient's household. The cost resources for each cost centre may differ between each country according to availability of services.

Tab. 6. Activity-Based Cost Centres of the TIMCI programme

**1**	Training cost for staff involved in delivering the programme:<br>- Consumable costs (including where applicable stationary, and other consumables)<br>- Personnel costs<br>- Overhead costs (including where applicable, rent for training venue, administrative costs, per diems and transport costs, and other overheads)
**2**	Delivery of intervention<br>- Annualized[1] fixed/equipment costs (including pulse oximetry device and CDSA tablet costs)<br>- Consumable costs (including medicines, diagnostic tests, and other consumables)<br>- Personnel costs
**3**	Out of pocket costs

- Any out of pocket costs paid by patients' households to the health system to access care and prescribed treatment will be collected, including resources purchased outside the facility due to unavailability in the facility. However, care not prescribed by facility healthcare professionals will not be included.

1. Since costing needs to account for both capital and recurrent costs, annualizing capital costs (i.e. calculating the annual value of a capital item) is the easiest way of combining capital and recurrent costs meaningfully. Annualizing capital cost is based on three pieces of important information: the initial purchase price of the item, its expected useful life, and a discount rate

Sources of cost data

Personnel and procurement units of the organisations involved in the TIMCI intervention, especially through different local PATH offices and sampled facilities will be sourced for the cost data. Centrally available databases will be used to estimate personnel and consumable (including medicines and diagnostics) costs where available.

Start-up costs, which will include training and community sensitisation costs, will be collected pre facility enrolment and intervention from PATH offices. After facility enrolment, cost data will be collected from non-medical administrative personnel. All personnel costs for those involved in the pre-intervention and intervention phase will also be collected since time in supervision, time of administrative support staff is as important as that of front line staff seeing patients. Personnel time, and volumes of diagnostic tests and treatments prescribed, will be obtained from the time-flow and service provision assessment studies. Data on time required for supervision and administrative tasks such as taking patient notes will be obtained from the senior medical professional at the facility, along with grades of personnel. Salaries of personnel according to grade will be sourced from centrally available databases which would include government salary publications for health facility workers (medical and non-medical) and complemented with salary costs sourced from health facility administrative databases where needed. Costs of consumables will also be sourced centrally where available and would for instance include cost of items on the National Essential Medicine List. Referral and hospitalization costs will be sourced from interviewing senior administrative personnel at referral health facilities.

Total programme cost and cost per child will be estimated. We will collect administrative data from appropriate levels in each country depending on organization of the specific health system, and will use time-flow data, and data collected elsewhere in the project before and after the intervention.

The table below lists the broad areas of cost data collection for each cost centre and the respective data sources.

Tab. 7. Sources of cost data

**Type of Cost**		**Identification**	**Measurement**		**Valuation**	
		Categories	Costing method	Sources of data	Valuation methods	Sources of data

**Recurrent** (costs incurred every year e.g. staff, maintenance, fuel)	Personnel (staff)	All staff involved (field workers and managers, administrators, support staff (drivers), etc)	Percentage of time spent on different activities within TIMCI; grade of personnel	Time data collected by operational research	Gross remuneration package costs	Centrally from government databases and complemented with provider's payroll department
	Consumables	Disposable items used per child per intervention	Quantity of consumables used per child	Medical personnel at health facility	Market prices	Centrally from government databases and complemented with provider's procurement department
	Equipment/ Capital (incurred every few years)	Pulse oximetry devices, tablets etc	Annualised cost of items	Interview, observations, and timing	Replacement prices	Provider's financial syndicate office
	Out of pocket costs	All out of pocket costs paid by patients' households to the health system for fever management	Direct fees and charges paid by households to health systems	Cost borne by households	Financial cost	Cost data collected form health system administrative records; information from interview of senior non-medical personnel

Definitions of types of costs and data to be collected

Staff time: For each activity within the TIMCI project, the responsible staff member involved with the activity will be identified. Secondly the time involved will be ascertained from the time-flow study and quantified. Supervision costs will similarly be estimated.

Consumables: The type and quantity of medicines, diagnostic tests and consumables used for each activity will be identified. In addition, unit costs of each consumable item will be obtained (for example the cost of an oxygen cylinder) and the size of unit (the time average spent on oxygen per patient).

Equipment: All equipment used for each activity will be identified and the price for which it was purchased. For the equipment costing, the lifespan for the equipment (usually between 3 – 5 years for smaller items including electronic tablets and pulse oximetry devices) will be obtained and annualized. Annualization will follow the cost recovery methodology where item cost, life span, and a defined interest rate is used to estimate the annual cost of an item. For equipment that is shared with other activities, the time spent per consultation will be obtained as a percentage of total time spent using each equipment and used to calculate the cost associated with the intervention after annualization. For donated equipment, market price information will be sought.

Equipment inventories and training records will be used to collect retrospective costs. Time spent by staff on consultations with children 0 – 59 months of age will be collected from data obtained in the time-flow study. Time costs will then be determined by combining staff annual salaries obtained from government sources and/or payroll offices with time spent in consultations with children. Where applicable, this personnel cost will be disclosed only to the principal researcher without the identity of staff involved. Consumables used will be examined to determine the unit cost of each consumable, while equipment will be annualized to determine their unit costs.

All capital and start-up costs will be annualised using an appropriate length of life and discount rate. Out of pocket payments to health systems borne by households will be estimated to capture all health system costs. These costs might vary across different settings since there might be flat rates in one setting and a varied fee schedule in another. Hence, the cost data collection for out of pocket payments will be country specific. The cost of hospital referrals where applicable, will also be estimated from interviews with senior healthcare professionals at the hospital level. Data will be captured and analysed in an excel workbook.

7.2 Data management

All data management procedures will be further described in the Data Management Plan (DMP).

7.2.1 Data flow, storage and transfer

The data management system will process and combine two main data flows: the research data flow, which will be managed by a secure third-party cloud based EDC platform, and the routine consultation data flow, which will be managed by a customized PostGreSQL Server (Unisanté/Wavemind, Lausanne, Switzerland). As illustrated in **Fig. 7**, there will be separate secured databases with restricted access rights for storing and processing CDSA consultation data, research data for each study, PII that are necessary for all research operations, and (possible) linking between the different databases.

- **Study databases** – These databases will contain de-identified quantitative research data collected in study facilities. Unless specified otherwise per country requirements in the DMP, these databases will be hosted on a device owned and maintained by the country research partner.

- **CDSA consultation database** – This database will contain routine consultation data extracted from CDSA in all intervention facilities. CDSA consultation data from study facilities will be de-identified, i.e. a link will exist between these data, the longitudinal observational study database and the identification database. There will be no such link for CDSA consultation data from non-study facilities, which will then be fully anonymised. Unless specified otherwise per country requirements in the DMP, this database will be hosted on a device owned and maintained by the country research partner.

- **Identification database** – This database will contain PII collected in study facilities. It will be kept under strict operating procedures, in particular it will not be shared or accessible outside of the country. The database will be used for generating follow-up call logs and random selection of a purposive sample of caregivers for qualitative studies. It will also be used for flagging possible duplicates in children enrolled in the longitudinal observational study.
- **Mapping database** – This database will be used for linking data from the study databases and the identification database. Each table will be maintained confidential under strict operating procedures for the duration of the study. The mapping between databases will not be shared or accessible outside of the research institution. When all data validation and regulatory requirements have been met, the mapping database will be destroyed, so that all the study databases and CDSA consultation database are ultimately fully anonymized and independent from each other.

In addition, **qualitative study repositories** with restricted access rights will store raw (audio-recordings, notes, drawings, photos, videos, GPS coordinates and document review grids) and processed (transcribed, translated and coded) labelled qualitative material. Unless specified otherwise per country requirements in the DMP, these repositories will be hosted on a device owned and maintained by the country research partner.

A data transfer agreement will be requested from the national competent authorities for providing access to and sharing a full copy of the de-identified study databases, de-identified consultation database and de-identified qualitative material with the core team at Swiss TPH. A subset of these database will be additionally shared with UoW as needed for the cost and cost-effectiveness study. Following approval from the national competent authorities, the copies will be stored on servers hosted in Switzerland / Canada. Data ownership will remain with the country research partner. After study termination or premature termination of the study, the de-identified databases and de-identified qualitative material will be archived by the country research partner in accordance with national laws. The shared copies will be archived for 25 years by Swiss TPH and UoW, unless otherwise stipulated by the applicable legislation. All repositories, databases and related software files will be backed up by the administrator in conjunction with any updates or changes.

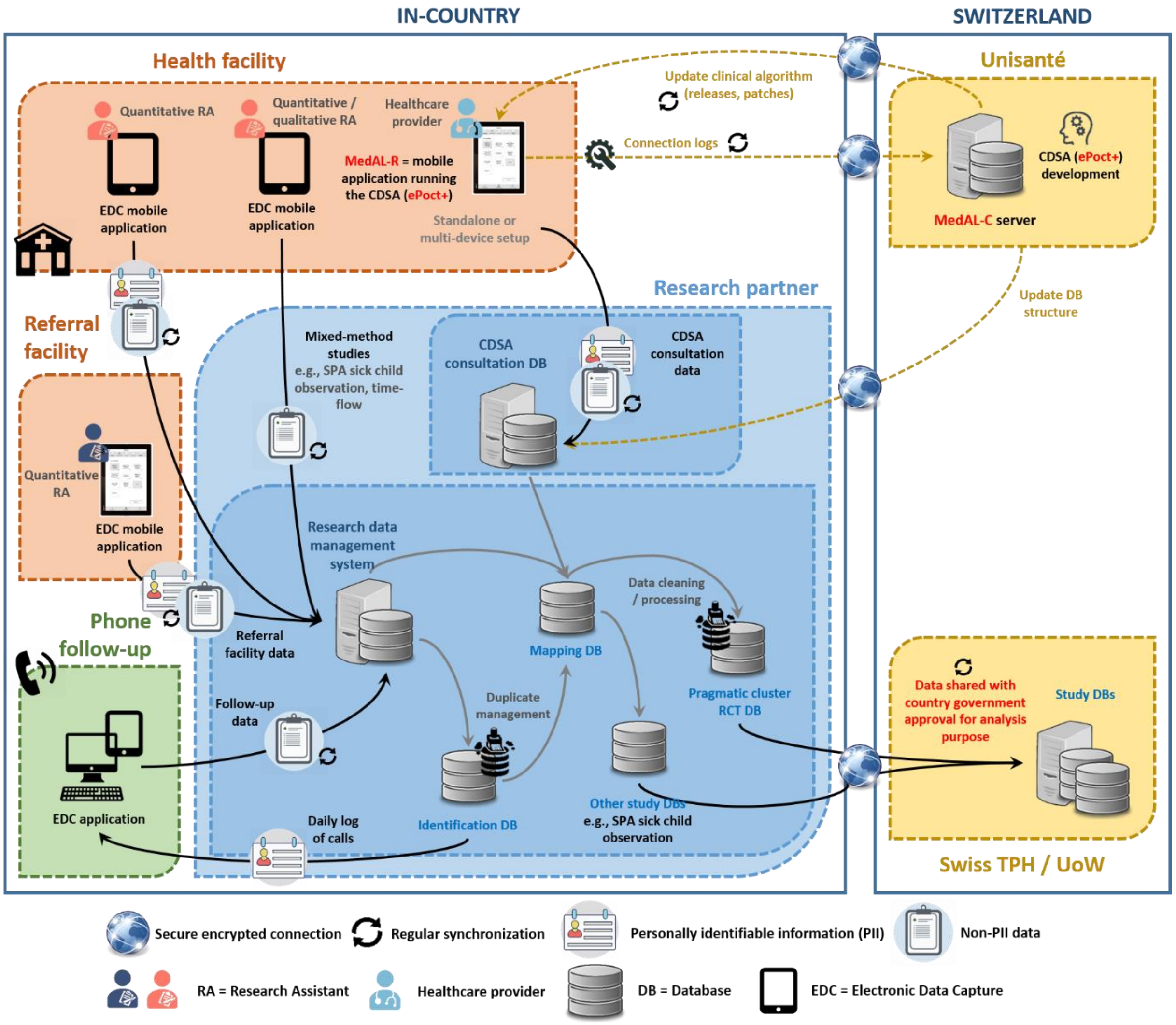

Fig. 7. Overview of the research data and consultation data flows and supporting IT architecture used within the TIMCI project. MedAL-R can receive updates directly from MedAL-C to be able to deploy patches as needed. The MedAL-C server is hosted in Switzerland and managed by the IT Unisanté team in Lausanne. This server will receive connection logs from the tablets when a network access is available.

7.2.2 Data entry, handling and validation

Quantitative research data

The EDC mobile application will perform offline validation (e.g., range and consistency) checks that immediately detect and request the correction of erroneous data entry, and prevent missing values upon compulsory data entry, guaranteeing completeness of the mandatory datasets. Standardized dictionaries such as SNOMED CT and ICD-10 will be implemented within the data collection tools (and hence available offline) in order to code clinical findings and symptoms in a consistent way. To ensure the integrity of the study-related duties and any data generated, each quantitative research assistant will log into the EDC mobile application using their secure user name and password. All passwords will be strictly personal and confidential. Electronically signed data from the mobile application will be regularly synchronized with the central server.

Procedures for cleaning and coding quantitative data will be developed by the core data manager at Swiss TPH to ensure consistency across countries. These procedures will be further described in the Data Management Plan. Data cleaning and coding activities will be supervised by the data manager in charge in each country, with possible country-specific additions. These activities will include, but will not be limited to, data de-identification, duplicate management and free text management. Data quality checks will be run on a daily basis. A follow-up log based on baseline visits and referral facility log based on Day 7 phone follow-up outputs will be generated daily. The server will certify the integrity of data by using in-built range and consistency checks to prevent errors during data manipulation.

An audit trail will be maintained for the life of the study for all quantitative research data. If a correction is required for an entry, the time and date stamps will track the person entering or updating entry and creates an electronic audit trail. There will be access control on the audit trails so that a user cannot view information about another user or site that they are not allowed to see. A synthetic visualization of the audit trail will be readily accessible by the data manager in charge in each country, so that they can check all modification to data and meta-data and who made them, as well as all successful and attempted system logins. At critical junctures of the longitudinal observational study, the research database will be reviewed and cleaned per established procedures, before being locked. Consistency between the data cleaning procedures in each country will be regularly reviewed. In addition, a dashboard will be developed for facilitating the review of key indicators to trigger in-depth data review.

CDSA individual consultation data

Each patient attending an intervention facility will be given a unique identifier in the CDSA used by this facility. Each new visit at the same facility will create a new case for the patient. Similar to EDC mobile application, MedAL-R will perform offline validation checks and relies on standardized dictionaries code clinical findings and symptoms in a consistent way. MedAL-R will allow the modification of consultation data by different healthcare providers within the health facility until the case has been closed to ensure the flexibility required by the clinical workflow. Routine consultation data from MedAL-R will be regularly synchronized with the CDSA server. Consultation data records will be electronically signed before being transferred to the central research database. A timestamp (date, time, device identifier number) will guarantee the integrity of the data associated with the electronic signature.

In non-study facilities, CDSA consultation data will be anonymised before transfer, so that no PII will leave the facility and these data can never be re-associated with the underlying individual.

Qualitative research data

Qualitative data will be voice recorded and complemented by written notes, drawings, photos and videos and GPS coordinates by a dedicated qualitative research assistant. Audio-recordings, notes, drawings, photos, videos, GPS coordinates and document review grids will be either written up in electronic files and/or directly transferred to the qualitative research

assistant's computer and uploaded to the central server. Electronic files will be stored on two separate password-protected devices according to country specific mechanisms (e.g. audio recording stored on a password protected computer and on an external drive in a password protected folder in locations where internet cannot be accessed; back-up on server as soon as internet is available and deleting from external drive).

Qualitative research data will be backed up at the first possible opportunity. Where connectivity to the central server is not possible, field teams will make a second back up on an encrypted external hard drive. Transcription and translations from voice recordings and hand-written notes will be entered into Word documents and translated into English and/or French. Hand-drawn process maps and health care seeking pathways will be digitalized.

7.2.3 Data privacy protection

Personally identifiable information will be maintained confidentially under strict SOPs, complying with all applicable international regulations. Study team members with access to PII will sign a confidentiality agreement prior to collecting or accessing any of the data. Each participant will be given a different unique alphanumeric identifier for each study in which he/she is associated. Voice recording files and written notes that refer to the same participant interview will be labelled using the same identifier. The IT infrastructure will provide password protected devices, laptops with encrypted hard drives, fileserver with customizable access rights, and documented and audited access right management. All transfer of data via the internet will be done via secure, encrypted connections.

All quantitative data collection instruments will be stored in closed, locked file cabinets with restricted access. The EDC platform will be encrypted and password protected. All study databases will be safeguarded against unauthorized access by established security procedures. A double coding procedure will ensure that participant re-identification is not feasible. The name of the facility of enrolment, geographic information and some elements of dates related to facility visits will also be de-identified. To further prevent re-identification of individuals (patients, caregivers and healthcare providers), where appropriate, data will be de-identified so that their level of granularity that does not allow indirect identification of an individual. In addition, the data imported from another study datasets will be processed (e.g., aggregated data, categorization of numeric values) so that a unique combination of facility and individual characteristics does not allow indirect identification of an individual.

Voice recordings, photos and videos, as well as GPS mapping, will be deleted from the devices after transcription, translation and digitalization. Transcriptions and translations of audio data and typed-up field notes will be stored on the central database in password protected folders accessible only to authorized qualitative researchers. Hand-written notes and drawings will be stored in locked filing cabinets in an area with restricted access. During the coding process qualitative data uploaded into the coding software will be manually de-identified (i.e. replacing names, locations, other personal identifiers) ensuring that only de-identified interview material is stored in the programme. Photos and videos for the method acting FGDs will be stored on

the central database in password protected folders. Audio track will be removed after transcription has been conducted, possibly using subtitles. For the purpose of method acting analysis, access to non-de-identified videos and photos will be requested from the national competent authorities for the international team of qualitative researchers involved in TIMCI (including the lead social scientist at Swiss TPH). Following approval from the national competent authorities, remote viewing will be made possible through a secured connection, with access rights, and for a limited period of time only. All raw qualitative material will be destroyed after validation of the final database of consolidated material.

7.2.4 Data quality assurance and control

Quality assurance activities across all studies will be more intense during study start-up to ensure quality data practices are instituted from the start of the trial. Further details will be given in the DMP and SOPs.

All research assistants will be trained to provide information and seek written consent using the information and consent form. Separate trainings will cover all aspects of quantitative data collection and all aspects of qualitative data collection. A competency assessment using scenarios will be done before commencing field work.

Data reconciliation between the different visits of the same child will be one of the most crucial data quality activities in the longitudinal observational study. Specific procedures will be developed for this purpose. To identify repeat visits of children that may re-attend the study facility where they enrolled, or any other study facility during the follow-up period, participants will be given a study card (or sticker) with a unique ID number. Retrospective search for data at referral level will be restricted to facilities within the geographical area where the study is running and will be targeted to include only children reported to have attended hospital by the caregiver at follow-up and those lost to follow-up. The name of the referral facility will be requested during the follow-up call. If available, it should further restrict the scope of the search and the probability of negative matches when cross-checking the participant identity. Children who re-attend any study facility more than 28 days after their previous enrolment will be enrolled for the new disease episode and given a new ID number. If available, the old ID number will be recorded to facilitate linking of the different disease episodes. Data to be potentially reconciled will be flagged by automated cross-check of a set of personal identifiers. Given that personal identifiers may not be identically recorded at each visit, fuzzy matching methods will be used to calculate a score of similarity between records and likelihood that they relate to the same child. Reconciliation of records with high similarity scores will be manually validated.

The validity of quantitative data will be ensured through data definitions and field restrictions in the electronic systems. Acceptable error rates in critical quantitative data will be established during the pilot phase and documented in the DMP. Pre-defined automated high-frequency checks will be performed including checks of completeness and indicators of other irregularities such as completion time. Reports will be shared with individual quantitative research assistants and their supervisors. In addition to reviewing automatically generated

reports for their supervisees, supervisors will perform random accuracy checks of research data compared to available source data. In the longitudinal observational study, quantitative research assistants responsible for the Day 7 call will be audited through random checks of phone logs and repeat phone calls. After confirmation that the study databases contain all expected data, an internal review will check their accuracy and quality. Once all the required data quality assurance and control have been completed and passed, the study databases will be “locked”.

During the pilot phase, qualitative tools will be translated and back translated, and tested using cognitive interviewing to ensure questions asked are understood as intended and felt to be appropriate. Regular quality checks of transcriptions and translations will be conducted by the senior social scientist in each country.

7.3 Statistical methods

Statistical methods will be described in detail in the Statistical Analysis Plan (SAP). We will conduct all analyses described below per country, in addition to a pooled cross-country analysis. Quantitative analyses will be conducted using Stata (or alternative statistical analysis software, depending on investigator for certain country-specific or sub-study specific analyses).

7.3.1 Longitudinal observational study

Analysis will include all eligible, consenting and enrolled children. Baseline characteristics will be described by country and across all study time points with summary statistics such as median and interquartile range or number and percentage. Outcomes will be described for each time period using summary statistics and results from the pre-intervention period (Q1) and the last intervention period (Q5) will be compared. The two primary outcomes will be assessed using a random effects logistic regression models with the cluster (facility) included as a random effect. All models will be adjusted for individual-level baseline characteristics such as gender and age whose distribution at the facility may have changed between the time periods. Results will be reported with odds ratios and 95% confidence intervals (CI). Binary secondary outcomes will be evaluated in the same way. Continuous secondary outcomes will be assessed using random effects linear regression models, reporting adjusted mean differences between time periods with 95% CIs.

We will conduct planned subgroup analysis of the primary outcomes to assess for potential effect modification in variables such as clinical presentation (particularly cough / difficulty breathing) and diagnosis (particularly severity classification). If these variables are found to be balanced between the two time periods, effect modification will be assessed by incorporating an interaction between the time period and the variable in the model, acknowledging that power will be low. Additional individual or facility level characteristics to be considered in the model will be further detailed in the statistical analysis plan (SAP).

We will conduct the primary analysis on all visits (for visits by the same child, this implies visits greater than 28 days apart as per the exclusion criteria). However, we will conduct a sensitivity

analysis excluding repeat visits of an individual child within the same quarter, to address the possibility that outcomes of children attending for more than one disease episode are correlated and impact the interpretation of the study. Due to the challenge in correctly identifying all repeated visits, the fairly low likelihood of repeat visits (based on available literature) and the acuity of the majority of sick child visits, we do not expect these analyses to produce very different results. We will conduct sensitivity analyses for measured and unmeasured confounders over time to assess robustness of the findings, which will be detailed in the SAP.

An exploratory interrupted time series analysis will be conducted to assess change over time, comparing outcomes for each of the quarters (Q1 – 5). Further exploratory analysis, including of data linked between the longitudinal observational study and the SPA and time-flow studies will be detailed in the SAP.

7.3.2 Routine data

Relevant aggregated data from routine HMIS will be used to monitor trends over time in all facilities and administrative regions where the project is running (i.e. both study facilities and non-study facilities) pre- and post-intervention.

Routine CDSA clinical data will be used to conduct descriptive analysis on the use of pulse oximetry and prevalence of hypoxaemia in non-longitudinal study facilities post-intervention. Univariate and multivariate analyses will assess factors associated with hypoxaemia such as age, sex and clinical presentation. This data will also be used to estimate health outcomes in facilities not involved in the longitudinal study, building a model from the data collected in facilities that are involved in the study. This estimate will then be gauged against HMIS data to identify indicators for longer term monitoring of the intervention.

7.3.3 Other mixed methods studies

Quantitative

Descriptive analysis will be conducted of the data obtained from the SPA studies by country, facility type and location and child and healthcare provider characteristics. Indices of adherence to key practices will be described and compared between and time points (pre-intervention and the 4 post intervention time points). Time-flow data will also be compared between pre-intervention and each of the post-intervention periods. Effect modifiers will be explored.

Monitoring and evaluation data from non-longitudinal study intervention facilities will be analysed along with aggregated HMIS data, with comparison to longitudinal study facilities to draw inferences on the impact and uptake of the wider programme of implementation.

Qualitative analyses

Quality assurance steps for transcription and translation will be built into the process.

Qualitative data will be transcribed verbatim using modified transcription rules by Kallmeyer and Schütze[51] and translated into English and/or French. A random sample of transcripts and translations will be quality checked by listening to audio-recordings and back translations and measures taken accordingly. After familiarization with all interviews/FGDs, an experienced qualitative researcher will code the transcripts using a qualitative coding software. A random sample of a total of 10-15% of the qualitative interviews will be coded using the framework analysis by Gale et al.[52] following an inductive and deductive approach; in-vivo codes will be created where applicable. The final draft code tree will be exported, codings deleted and the same interviews coded by a second and independent qualitative researcher for inter-rater reliability check. The Kappa-Cohen value will be calculated. Differences in coding will be discussed and a joint solution developed. A Kappa-Cohen value above 80% will allow using the code tree after minor adaptations based on joint agreements by the two coders and for final coding. The finalized code tree will be used to code the entire material. Finally, each code and each sub-code with its coded material will be exported into excel tables. A team of qualitative researchers will review the tables and jointly analyse the data identifying patterns, similarities, and differences as well as change over time. Secondary analysis of data (i.e. including comparison of final code trees) from all study countries may be conducted jointly and findings compared across countries.

Document review

The document review data will be entered into a grid with predefined categories in line with process evaluation criteria. Data will be triangulated against qualitative and quantitative data collected.

Process evaluation

The process evaluation will draw on data from the various sub-studies to describe the context, implementation process and mechanisms of impact as outlined in **Fig 8**.

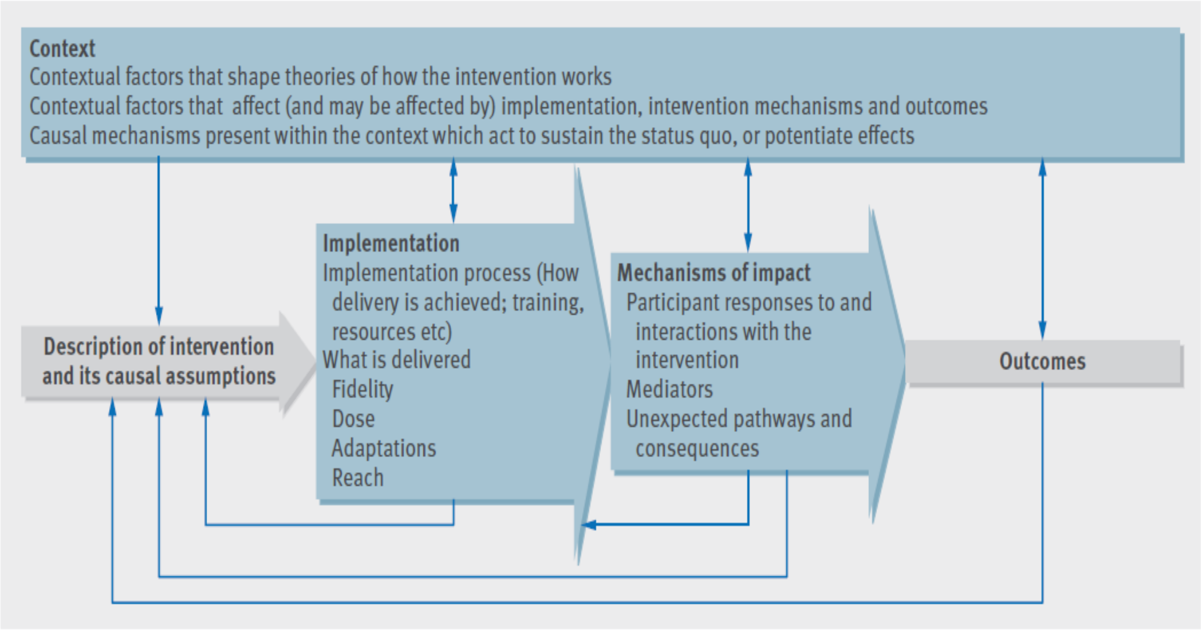

Fig 8. Overview of MRC process evaluation of complex interventions (Image credit Moore et al)[35]

7.3.4 Cost and modelled cost-effectiveness study

When all the cost components have been collected, the data will be aggregated or collated in Excel. Once the summary is completed, a cost profile of the programme activities will be derived and after the total costs of the programme have been calculated, the unit costs will then be derived (i.e. total costs divided by the sample size). These profiles are useful in highlighting major cost components (and thus identifying potential areas where improvements in efficiency may have significant impact on costs). For this evaluation, analysis will focus on full costing and incremental costing approach. Total, average and incremental costs per year will be estimated. Costs associated with increasing the oxygen saturation referral threshold will be estimated based on the additional proportion of children meeting referral criteria.

When available, effectiveness data will be combined with the cost data and cost per DALY averted will be estimated. Effectiveness will be modelled using the information on severe outcomes such as mortality, and secondary hospitalization, adjusting as far as possible the level of severity of illness of the child at presentation.

At primary care facilities, these will include measurements (when taken by healthcare providers) such as anthropometry, respiratory rate, temperature and routine investigations where available (e.g. haemoglobin, CRP), and oxygen saturation (post-intervention/in intervention arms). We will also use diagnosis and severity classification recorded by primary care providers.

At hospital level, we will also utilize the same measurement data, and other vital sign data (such as a paediatric early warning score if recorded), decision to admit, intensive care and / or ventilation, provisional and final diagnosis, admission outcome (discharge, death, left against medical advice) and length of hospital stay. We will then use these indicators, along with mortality from phone call follow-up data by Day 28, to predict probability of mortality based

on available literature. The DALY calculation will be based primarily on probability of mortality over a relatively short horizon (to be determined based on a review of literature, likely between three and six months to reflect the time windows used in post hospital discharge mortality studies). The types of illnesses considered do not typically lead to long-term disability, and the duration of illness is too short to have a big impact on DALY calculations. We will not use age weights: we will use GBD (2017) DALYs, [53] and we will not use country-specific disutility data, both since we are unsure these exist for all the countries, and they render cross-country comparisons more difficult.

Sensitivity analysis

As not all data will be available precisely, we will explore possible assumptions using sensitivity analysis which will assess how the estimates would react to percentages changes in the value of the assumptions. It can help the reviewer to determine which parameters are the key drivers of a model's results. Guidelines and standard textbook recommend that economic evaluations should include sensitivity analysis.[54] We will undertake one-way sensitivity analysis of key parameters using the major outcome and cost parameters. Typically. Such analyses are more sensitive to the assumptions about outcomes, and for this we have to rely on the best judgement of the epidemiologists.

8 Monitoring

All studies will be conducted in accordance with the protocol and international and applicable national regulatory requirements. The Clinical Operations Unit within the Department of Medicine at Swiss TPH will provide training of trainers on ethics and monitoring for research partners if required. Country research partners will be responsible for setting up and carrying out the monitoring plan, in consultation with Swiss TPH. This will include regular monitoring of screening data, consent, recruitment rates and missing data on primary and important secondary endpoints. Outliers on key indicators will trigger site visits.

9 Ethics and dissemination

9.1 Research ethics approval

Following two independent scientific merit reviews, the protocol will be submitted to all relevant research ethics committees / institutional review boards in each country. Receipts of submission to each local ethical review board will be submitted with the master protocols to the WHO Research Ethics Review Committee (ERC).

For Kenya, this is the University of Nairobi-Kenyatta National Hospital ERC (KNH-UoN ERC)

For Myanmar, the protocol will be submitted first to the International Relations Department (IRD) to obtain the Union Minister's (Ministry of Health and Sports) approval to conduct research and a decision on which IRB to submit the protocol to. For example, this will be

either a.) Myanmar Department of Medical Research (DMR) or b.) University of Public Health ERC (UoPH), finalised depending on MoHS decision.

For Senegal, this is the Comité National d'Ethique por la Recherche en Santé (CNERS)

9.1.1 Regulatory status of the intervention

Pulse oximetry

The pulse oximetry device selected for the TIMCI project is the Acare AH-MX device. This was selected as it is available through the UNICEF catalogue, which infers a level of quality and device appropriateness. The competitive procurement process established by UNICEF incorporates pulse oximetry specifications developed through a collaborative process involving global and national level experts. Devices in the catalogue have regulatory approval from a stringent regulatory authority (SRA) such as the United States Food and Drug Administration (US FDA) or Conformité Européenne (CE) mark.

Country level regulatory approval will vary. It is possible that the devices will have approval in some of the countries. If the device does not have pre-existing regulatory approval in a project country, the project team will work with the MOH to receive a waiver to use the device for the study purposes. If appropriate, the study team will continue to work with the manufacturer and MOH in each country to receive a permanent regulatory approval to facilitate scale of that device.

Clinical Decision Support Algorithm

The regulatory classification of clinical decision support software is currently subject to debate at the US FDA, depending on how and in what context the software is used. In this study, the CDSA is intended to provide decision support for the diagnosis and treatment of childhood illness. It will collate and analyse patient-specific information to suggest possible diagnoses and recommend treatment plans or diagnostic tests to healthcare providers (HCPs). These suggestions and recommendations will be evidence-based and will support the decision-making of the HCP when considering treatment options or diagnostic tests for a patient. The HCP may then use this information to make a decision about the care of the patient, along with other information and factors of which he/she is aware. In addition, the CDSA presents a low risk profile since it will support the implementation of and strengthen the adherence to standard clinical guidelines. These functions do not meet the definition of a medical device per country regulatory authorities and the CDSA is therefore regarded as a non-regulated device.

9.2 Protocol amendments

Any important protocol modifications will be submitted to relevant research ethics committees, trial registries and study sites including participants where appropriate.

9.3 Consent or assent

9.3.1 Longitudinal observational study

Information and consent procedures will be conducted in concordance with national regulatory and ethical requirements (for example on the criteria applied to determine capacity of a caregiver to provide consent). Prior to study start, information and consent mechanisms and content will be reviewed and refined with community advisory boards and piloted with participants and modifying if necessary to ensure appropriate.

As the intervention is at the facility level, approvals from relevant authorities will be sought before engaging with facility management and staff prior to study start.

Informed consent from caregivers will be sought for the collection of data and follow-up of individual children in longitudinal observational study. Information will be provided in the waiting area of facilities in accordance with country-specific mechanisms, which may include written, pictorial, video or spoken information. Potentially eligible children will be identified at registration (based on age) during working hours and will be screened by a research assistant in the waiting area for eligibility. Consecutive caregivers of eligible children will be provided with more detailed information on the study and provided with an opportunity to ask questions. Written informed consent will be obtained for those that agree to participate. In the case of an illiterate caregiver, an impartial witness will be asked to sign on their behalf, along with a thumbprint of the caregiver. Caregivers will be informed that they can withdraw at any point up to study completion (and anonymization) and that non-participation or withdrawal will not affect their care in any way. If the clinical condition of the child requires immediate treatment, this will supersede the written consent process, which will be conducted once the child is stabilized, and if this is still not possible, they will not be included in the study.

9.3.2 Other mixed methods studies

Additional consent from caregivers, healthcare providers and stakeholders will be sought for the embedded mixed methods studies. For the SPA, this will be conducted at the health facility as per the longitudinal study.

The qualitative research assistant (possibly with the help of an assistant such as a community health worker) will invite caregivers for participation providing verbal information and asking for verbal consent. Prior to the start of the IDI or FGD participants will receive written information and asked to provide written consent. For method acting FGDs, explicit consent will be sought for photo- and video-recording. Participants will have the option to take part in the FGD but withhold consent for video-/photo-recording. In this instance, the respective participants will be kept out of shot.

Caregivers for walking interviews will be recruited by the research assistant permanently based in the health facility and as part of the routine longitudinal observational study recruitment process including information and written consent (with procedures for illiterate participants

as per the longitudinal observational study). Recruitment for walking interviews will only be conducted on days during which the qualitative research assistant is present. The research assistant will inform the qualitative data collector in case a caregiver has provided consent. Caregivers who have received referral advice (excluding those with a child with a life threatening emergency condition requiring immediate action) will be approached by the qualitative research assistant prior to leaving the facility and additional verbal consent will be sought.

For healthcare providers written information will be provided and written consent sought. No information on individual decisions to accept or decline participation will be shared, neither with supervisors nor with health authorities. For key stakeholders, written information will be provided and verbal consent sought prior to an interview via telephone; or written consent if an interview is conducted person-by-person.

9.3.3 Cost and modelled cost-effectiveness study

We will obtain consent from healthcare providers (including medical and non-medical personnel) participating in the costing study. An information sheet on the study will be provided prior to data collection, discussed with target providers from sampled health facility before written consent is obtained. Data relevant to the costing study collected as part of other studies will be extracted and provided as anonymised, summary data for the purposes of the costing study.

9.4 Confidentiality

Participant identifiable data will only be collected for the purpose of study conduct (participant follow-up, duplicate checking, as well as qualitative analysis) and will be destroyed in accordance with standard operating procedures.

9.4.1 Longitudinal observational study

Study and participant data will be handled confidentially and will only be accessible to authorised personnel who require the data to fulfil their duties within the scope of the study. All personnel who have access to data forms will be asked to sign confidentiality agreements. We will make sure consent forms make provision for future sharing of data.

A unique identifier number will be assigned to each participant (including healthcare providers) and facilities.

9.4.2 Other mixed methods studies

Qualitative data will be audio recorded using an encrypted device after which it will be transcribed and translated verbatim (personal identifiers not removed) in each country with staff signing a confidentiality clause. During walking interviews audio-recordings will only be done during the bilateral interview between the caregiver and data collector (but e.g. switched off when the caregiver has a conversation with a third person). Hand-written notes of relevant

conversations and observations will be taken in addition. Audio data will be fully de-identified when uploaded to and read through in the qualitative coding programme and according to SOPs including use of pseudonyms. GPS tracking data of the walking interviews, as well as photos and videos of the method acting FGDs will be additionally analysed. Audio tracks from videos will be removed after transcription has been conducted and replaced by transcripts and/or sub-titles. Only the qualitative data collectors facilitating the FGD and the authorised qualitative researchers (including qualitative researchers in country, in other TIMCI countries, and the Swiss TPH lead social scientist) will view the non-de-identified acting method videos/photos for the purpose of primary analysis, after signing a confidentiality agreement and agreeing with the Code of Conduct. All raw qualitative material will be destroyed after validation of the final database of consolidated qualitative material. Further information on data management can be found in Section 7.2.

9.4.3 Linking between longitudinal observational study and mixed methods studies

Data collected during the mixed-method studies will be linked with a subset of data collected during the longitudinal observational study. The privacy protection of enrolled participants will be ensured following a multiple coding procedure: each participant will be given a different unique alphanumeric identifier for each study in which he/she is associated. These identifiers will be linked with the longitudinal observational study identifier by the clinical management system. This means that the knowledge of both codes and their mutual link is needed to access the full dataset about a participant. The link between two study identifiers will be maintained confidential information under strict operating procedures (in a central, secured database with restricted access rights) for the duration of the study. In particular, this information will not be shared or accessible outside of the research institution. When all data validation and regulatory requirements have been met, the link between study identifiers will be destroyed, so that all study databases are ultimately fully independent.

9.4.4 Cost and modelled cost-effectiveness study

Confidentiality of personnel cost of staff participating in the study will be ensured by protecting the identity of staff whose grade level will be collected as part of the study. Information on staff salaries collected will be stored in a restricted cabinet with only access to the principal investigator. All data collected will be stored with other intervention data in the research server with limited access. We will assign unique identifiers to health facilities and maintain a link between facility name and identifier stored in the research server. However, for the analyses which will be performed in Waterloo, Canada, only de-identified data will be used.

9.5 Risks and benefits to participants

No major risks are foreseen given the low risk nature of the intervention. The only potential risk envisaged is the time involved in participation in the study, which will be clearly outlined during the informed consent process. The potential risk of delaying care of sick children as a

result of recruitment to the study will be mitigated by training of research assistants and sensitization of healthcare providers to ensure that they are aware that no research activity should take priority over clinical care. Scenarios in which they should not attempt to recruit participants will be provided during training to ensure this is clearly understood.

No participants will receive incentives, but participants, particularly in studies requiring greater time commitment, may receive a small reimbursement (monetary/non-monetary) for their time (e.g. to compensate for travel costs or lost time) and according to country specific standards.

In order to avoid the risk of diverting healthcare providers from clinical duties, all data collection from healthcare providers will be scheduled at appropriate times.

Whilst there are no direct benefits to participants, this study could benefit them and their community in future as a result of the understanding generated by this study.

9.6 Access to data

Access to study data will be governed by a data sharing agreement between each country research partner, Swiss TPH, UoW and PATH, in accordance with country-specific regulations. The final, anonymised datasets will be made available on an open access data sharing platform after the end of the study, in order to promote transparency and facilitate global cooperation in child health research.

9.7 Dissemination policy

The protocol will be published after all ethical approvals have been received.

Findings from the study will be shared through community engagement mechanisms described in section 6.3. Findings will also be shared with the healthcare providers at facilities involved in the study, and supervisors / other stakeholders within district health management teams. Advocacy work through various mechanisms at national and international level based on the findings of the study will be conducted as part of Output 4 of the TIMCI project.

Results will be shared with the wider scientific community through presentations at national and international conferences, and through open-access peer-reviewed journal publications.

Tab. 8. Multi-method intervention evaluation overview

	Sub-study title	Objectives	Design	Tools
Impact of intervention on QoC (health outcomes, key practices)	Pragmatic cluster randomised controlled trial (India & Tanzania)	Large-scale evaluation of impact of pulse oximetry and CDSAs on health outcomes (death, hospitalisations, cure rate) and healthcare provider practice (antimicrobial prescriptions, referral decisions)	3-arm parallel cluster RCT comparing:<br>• Pulse ox + CDSA<br>• Pulse ox + paper job aid<br>• Routine care (refresher training)	D0 – clinical/sociodemographic data at facility<br>D7 – primary outcome<br>D28 – secondary outcome
	Longitudinal observational study (Kenya, Myanmar, Senegal)	To evaluate impact of pulse oximetry / CDSAs on quality of care (referral, antimicrobial prescriptions with exploratory assessment of outcomes	Quasi-experimental pre-post:<br>• 3 mo baseline<br>• 1 year intervention (early/late)<br>• 3 mo comparison Q1 and Q5	**D0 – clinical/sociodemographic data at facility**<br>D7 - outcome / health-seeking
	Service provision assessments	To evaluate the effect of the intervention on adherence to IMCI guidelines, including the correct classification and management of children under five years & to evaluate health system factors associated with impact	Periodic assessment (in each phase) using Service Provision Assessment methodology – i.e. collecting data at facilities for (1-5) days per facility per quarter	Facility assessment<br>HCP interview<br>Sick child observation<br>Exit interview
Evaluation of implementation (incorporating process evaluation, acceptability framework, realist approach)	Facility-based process mapping and time-flow study	To understand change in care processes including time taken for consultation and use of devices	Baseline and intervention process mapping with group discussion at sample of facilities, with observation and timing of key steps	Process map<br>Time-flow study
	Healthcare provider perceptions of the intervention	To understand acceptability, usability of devices by HCPs and gain insight on perceived facilitators and barriers to successful implementation	Qualitative work with HCPs from a sample of facilities in pilot, early and late intervention	IDIs<br>FGDs
	Caregiver perceptions of the intervention and health-seeking behaviour	To understand knowledge, attitudes and behaviour (i.e. experience of care, care seeking pathways, perception of devices, adherence to advice (in last consultation), child health literacy) related to the health care received	Community follow-up of individual caregivers, purposively selected (e.g. referred / unreferred, adverse / non-adverse outcomes) during intervention period	IDIs, Case Studies
		To explore knowledge, attitudes, values, beliefs related to the devices, experiences with clinical care with a focus on aspects of quality of care (i.e. people-centred outcomes, provision of care and experience of care).	FGD of purposively selected caregivers (urban / rural, type of facility, location) grouped by age/sex; During pilot, early and late intervention phases	FGDs
	Stakeholder perceptions	To understand health care provision pre-intervention; the intervention mechanisms / decision-making, identification of facilitators and barriers to implementation, best practices, inform package for scale	Baseline & late intervention	KIIs with govt & project stakeholders
	Project data review		Late intervention	Desk review incl. project data e.g. on devices, training

10 References

1. United Nations Inter-agency Group for Child Mortality Estimation (UN IGME). *Levels & Trends in Child Mortality: Report 2019, Estimates developed by the United Nations Inter-agency Group for Child Mortality Estimation*. (2019).
2. UN General Assembly Resolution. *Transforming our world: the 2030 Agenda for Sustainable Development. A/RES/70/1*.
3. Costello, A., Dalglish, S. & on behalf of the Strategic Review Study Team. *Towards a Grand Convergence for child survival and health: A strategic review of options for the future building on lessons learnt from IMNCI*. (2016).
4. World Health Organization (WHO). *Integrated Management of Childhood Illness Chartbook*. (2014).
5. Nguyen, D. T. K., Leung, K. K., McIntyre, L., Ghali, W. A. & Sauve, R. Does Integrated Management of Childhood Illness (IMCI) Training Improve the Skills of Health Workers? A Systematic Review and Meta-Analysis. *PLoS ONE* **8**, e66030 (2013).
6. Krüger, C., Heinzel-Gutenbrunner, M. & Ali, M. Adherence to the integrated management of childhood illness guidelines in Namibia, Kenya, Tanzania and Uganda: evidence from the national service provision assessment surveys. *BMC Health Serv Res* **17**, 822 (2017).
7. Horwood, C. *et al.* An Evaluation of the Quality of IMCI Assessments among IMCI Trained Health Workers in South Africa. *PLoS ONE* **4**, e5937 (2009).
8. Walter, N. D. *et al.* Why first-level health workers fail to follow guidelines for managing severe disease in children in the Coast Region, the United Republic of Tanzania. *Bull World Health Organ* **87**, 99–107 (2009).
9. Bjornstad, E. *et al.* Determining the quality of IMCI pneumonia care in Malawian children. *Paediatrics and International Child Health* **34**, 29–36 (2014).
10. Keitel, K., Kilowoko, M., Kyungu, E., Genton, B. & D'Acremont, V. Performance of prediction rules and guidelines in detecting serious bacterial infections among Tanzanian febrile children. *BMC Infectious Diseases* **19**, 769 (2019).

11. Agweyu, A. *et al.* Appropriateness of clinical severity classification of new WHO childhood pneumonia guidance: a multi-hospital, retrospective, cohort study. *The Lancet Global Health* **6**, e74–e83 (2018).
12. Chisti, M. J. *et al.* Clinical predictors and outcome of hypoxaemia among under-five diarrhoeal children with or without pneumonia in an urban hospital, Dhaka, Bangladesh: **Clinical predictors and outcome of hypoxemia**. *Tropical Medicine & International Health* **17**, 106–111 (2012).
13. McCollum, E. D. *et al.* Pulse oximetry for children with pneumonia treated as outpatients in rural Malawi. *Bull. World Health Organ.* **94**, 893–902 (2016).
14. McCollum, E. D., Bjornstad, E., Preidis, G. A., Hosseinipour, M. C. & Lufesi, N. Multicenter study of hypoxemia prevalence and quality of oxygen treatment for hospitalized Malawian children. *Transactions of the Royal Society of Tropical Medicine and Hygiene* **107**, 285–292 (2013).
15. Lazzerini, M., Sonego, M. & Pellegrin, M. C. Hypoxaemia as a Mortality Risk Factor in Acute Lower Respiratory Infections in Children in Low and Middle-Income Countries: Systematic Review and Meta-Analysis. *PLoS ONE* **10**, e0136166 (2015).
16. Graham, H. *et al.* Hypoxaemia in hospitalised children and neonates: A prospective cohort study in Nigerian secondary-level hospitals. *EClinicalMedicine* **0**, (2019).
17. Subhi, R. *et al.* The prevalence of hypoxaemia among ill children in developing countries: a systematic review. *The Lancet Infectious Diseases* **9**, 219–227 (2009).
18. Orimadegun, A. E., Ogunbosi, B. O. & Carson, S. S. Prevalence and predictors of hypoxaemia in respiratory and non-respiratory primary diagnoses among emergently ill children at a tertiary hospital in south western Nigeria. *Transactions of The Royal Society of Tropical Medicine and Hygiene* **107**, 699–705 (2013).
19. Chinawa, J., Ubesie, A., Chukwu, B., Ikefuna, A. & Emodi, I. Prevalence of hypoxemia among children with sickle cell anemia during steady state and crises: A cross-sectional study. *Niger J Clin Pract* **16**, 91 (2013).

20. Morgan, M. C. *et al.* Pulse oximetry values of neonates admitted for care and receiving routine oxygen therapy at a resource-limited hospital in Kenya. *Journal of Paediatrics and Child Health* **54**, 260–266 (2018).
21. Adebola, O., Babatunde, O. & Bose, O. Hypoxemia predicts death from severe falciparum malaria among children under 5 years of age in Nigeria: The need for pulse oximetry in case management. *Afr H. Sci.* **14**, 397 (2014).
22. Nantanda, R., Tumwine, J. K., Ndeezi, G. & Ostergaard, M. S. Asthma and Pneumonia among Children Less Than Five Years with Acute Respiratory Symptoms in Mulago Hospital, Uganda: Evidence of Under-Diagnosis of Asthma. *PLOS ONE* **8**, e81562 (2013).
23. Institute for Health Metrics and Evaluation (IHME). *GBD Compare*. http://vizhub.healthdata.org/gbd- compare (2015).
24. Rojas-Reyes, M. X., Granados Rugeles, C. & Charry-Anzola, L. P. Oxygen therapy for lower respiratory tract infections in children between 3 months and 15 years of age. *Cochrane Database of Systematic Reviews* (2014) doi:10.1002/14651858.CD005975.pub3.
25. Enoch, A. J., English, M. & Shepperd, S. Does pulse oximeter use impact health outcomes? A systematic review. *Arch Dis Child* **101**, 694–700 (2016).
26. Duke, T. *et al.* Improved oxygen systems for childhood pneumonia: a multihospital effectiveness study in Papua New Guinea. *The Lancet* **372**, 1328–1333 (2008).
27. Floyd, J. *et al.* Evaluating the impact of pulse oximetry on childhood pneumonia mortality in resource-poor settings. *Nature* **528**, S53–S59 (2015).
28. Blanc, J. *et al.* Retrospective study on the usefulness of pulse oximetry for the identification of young children with severe illnesses and severe pneumonia in a rural outpatient clinic of Papua New Guinea. *PLoS ONE* **14**, e0213937 (2019).
29. Shao, A. F. *et al.* New Algorithm for Managing Childhood Illness Using Mobile Technology (ALMANACH): A Controlled Non-Inferiority Study on Clinical Outcome and Antibiotic Use in Tanzania. *PLOS ONE* **10**, e0132316 (2015).

30. Keitel, K. *et al.* A novel electronic algorithm using host biomarker point-of-care tests for the management of febrile illnesses in Tanzanian children (e-POCT): A randomized, controlled non-inferiority trial. *PLoS Med* **14**, (2017).

31. Keitel, K. *et al.* A novel electronic algorithm using host biomarker point-of-care tests for the management of febrile illnesses in Tanzanian children (e-POCT): A randomized, controlled non-inferiority trial. *PLoS Med* **14**, (2017).

32. Lange, S., Mwisongo, A. & Mæstad, O. Why don't clinicians adhere more consistently to guidelines for the Integrated Management of Childhood Illness (IMCI)? *Social Science & Medicine* **104**, 56–63 (2014).

33. WHO. *Standards for improving the quality of care for children and young adolescents in health facilities*. (2018).

34. Pawson, R. & Tilley, N. Realist Evaluation. (2004).

35. Moore, G. F. *et al.* Process evaluation of complex interventions: Medical Research Council guidance. *BMJ* **350**, (2015).

36. Sekhon, M., Cartwright, M. & Francis, J. J. Acceptability of healthcare interventions: an overview of reviews and development of a theoretical framework. *BMC Health Services Research* **17**, 88 (2017).

37. Michie, S., van Stralen, M. M. & West, R. The behaviour change wheel: A new method for characterising and designing behaviour change interventions. *Implementation Sci* **6**, 42 (2011).

38. De Silva, M. J. *et al.* Theory of Change: a theory-driven approach to enhance the Medical Research Council's framework for complex interventions. *Trials* **15**, 267 (2014).

39. Taplin, Dana, Clark, H., Collins, E. & Colby, D. C. *Theory of Change Technical Papers: A Series of Papers to Support Development of Theories of Change Based on Practice in the Field*. (2013).

40. DHS Service Provision Assessment Tools. https://dhsprogram.com/pubs/pdf/SPAQ2/SICK_CHILD_OBSERVATION_PROTOCOL_06012012.pdf.

41. Sheffel, A., Karp, C. & Creanga, A. A. Use of Service Provision Assessments and Service Availability and Readiness Assessments for monitoring quality of maternal and newborn health services in low-income and middle-income countries. *BMJ Glob Health* **3**, e001011 (2018).

42. World Health Organization. *Service Availability and Readiness Assessment (SARA) -an annual monitoring system for service delivery: Reference Manual, Version 2.2*. https://apps.who.int/iris/bitstream/handle/10665/149025/WHO_HIS_HSI_2014.5_eng.pdf?sequence=1 (2015).
43. Acare Technology Co., Ltd. -AH-MX. *Acare Technology Co., Ltd.* http://www.acaretech.com/product_872390.html.
44. Uwemedimo, O. T. *et al.* Distribution and determinants of pneumonia diagnosis using Integrated Management of Childhood Illness guidelines: a nationally representative study in Malawi. *BMJ Global Health* **3**, e000506 (2018).
45. Ministry of Health and Social Welfare (MoHSW) [Tanzania Mainland], Ministry of Health (MoH) [Zanzibar], National Bureau of Statistics (NBS), Office of the Chief Government Statistician (OCGS) & ICF International. *Tanzania Service Provision Assessment Survey 2014-2015*. https://dhsprogram.com/pubs/pdf/spa22/spa22.pdf (2016).
46. Krüger, C., Heinzel-Gutenbrunner, M. & Ali, M. Adherence to the integrated management of childhood illness guidelines in Namibia, Kenya, Tanzania and Uganda: evidence from the national service provision assessment surveys. *BMC Health Serv Res* **17**, 822 (2017).
47. *Oxygen therapy for children*. (World Health Organization, 2016).
48. Rojas-Camayo, J. *et al.* Reference values for oxygen saturation from sea level to the highest human habitation in the Andes in acclimatised persons. *Thorax* **73**, 776–778 (2018).
49. Emdin, C. A. *et al.* Utility and feasibility of integrating pulse oximetry into the routine assessment of young infants at primary care clinics in Karachi, Pakistan: a cross-sectional study. *BMC Pediatr* **15**, 141 (2015).
50. Boyd, N. *et al.* Usability Testing of a Reusable Pulse Oximeter Probe Developed for Health-Care Workers Caring for Children < 5 Years Old in Low-Resource Settings. *The American Journal of Tropical Medicine and Hygiene* **99**, 1096–1104 (2018).
51. Kallmeyer, W. & Schütze, F. Konversationsanalyse. in *Studium Linguistik* (Scriptor Verlag, 1976).

52. Gale, N. K., Heath, G., Cameron, E., Rashid, S. & Redwood, S. Using the framework method for the analysis of qualitative data in multi-disciplinary health research. *BMC Med Res Methodol* **13**, 117 (2013).

53. James, S. L. *et al.* Global, regional, and national incidence, prevalence, and years lived with disability for 354 diseases and injuries for 195 countries and territories, 1990–2017: a systematic analysis for the Global Burden of Disease Study 2017. *The Lancet* **392**, 1789–1858 (2018).

54. Drummond, M. F., Sculpher, M. J., Claxton, K., Stoddart, G. L. & Torrance, G. W. *Methods for the economic evaluation of health care programmes*. (Oxford university press, 2015).

11 Appendices

11.1 Informed consent forms appended to the protocol

- 01 TIMCI_ICF_LS_V0.3.1_03Jun2020
- 02 TIMCI ICF LS CG SPA V0.2.1, 03Jun2020
- 03 TIMCI ICF LS CG TF V0.2.1, 03Jun2020
- 04 TIMCI ICF LS CG Walking Interview V0.2.2, 03Jun2020
- 05.1 TIMCI ICF LS CG IDI intervention V0.2.2, 03Jun2020
- 05.2 TIMCI ICF LS CG IDI non-intervention V0.2.2, 03Jun2020
- 06 TIMCI ICF LS CG FGD V0.2.2, 03Jun2020
- 07 TIMCI ICF LS CG method actingV0.2.2, 03Jun2020
- 08 TIMCI ICF LS HCP SPA V0.2.1 03Jun2020
- 09.1 TIMCI ICF LS HCP IDI V0.2.2, 03Jun2020
- 09.2 TIMCI ICF LS HCP IDI pre-intervention V0.2.2, 03Jun2020
- 10 TIMCI ICF LS HCP FGD V0.2.2, 03Jun2020
- TIMCI ICF SH KII Intervention V0.2.2, 03Jun2020
- TIMCI ICF SH survey V0.2.2, 03Jun2020
- TIMCI ICF LS costing V0.2, 04Jun2020

11.2 Study instruments appended to the protocol

- 01 TIMCI LS CRF description
- 02 TIMCI LS SPA Exit Interview instrument
- 03 TIMCI LS SPA Facility Assessment instrument
- 04 TIMCI LS SPA HealthCare Provider Interview
- 05 TIMCI LS SPA Sick Child Observation instrument
- 06 TIMCI LS process map timeflow tool
- 07 TIMCI LS healthcare provider IDI
- 08 TIMCI LS healthcare provider FGD
- 09 TIMCI LS caregiver IDI
- 10 TIMCI LS caregiver walking interview
- 11 TIMCI LS caregiver FGD
- 10 TIMCI LS stakeholder KII
- 13 TIMCI LS document review matrix
- 14 TIMCI LS stakeholder survey
- 15 TIMCI LS medical personnel questionnaire
- 16 TIMCI LS non-medical personnel questionnaire
- 17 TIMCI LS hospital costs
- 18 TIMCI LS ICF costing
- 19 TIMCI LS training community sensitization costs

11.3 Budget and collaboration appended to the protocol

11.4 TIMCI Preliminary Theory of Change

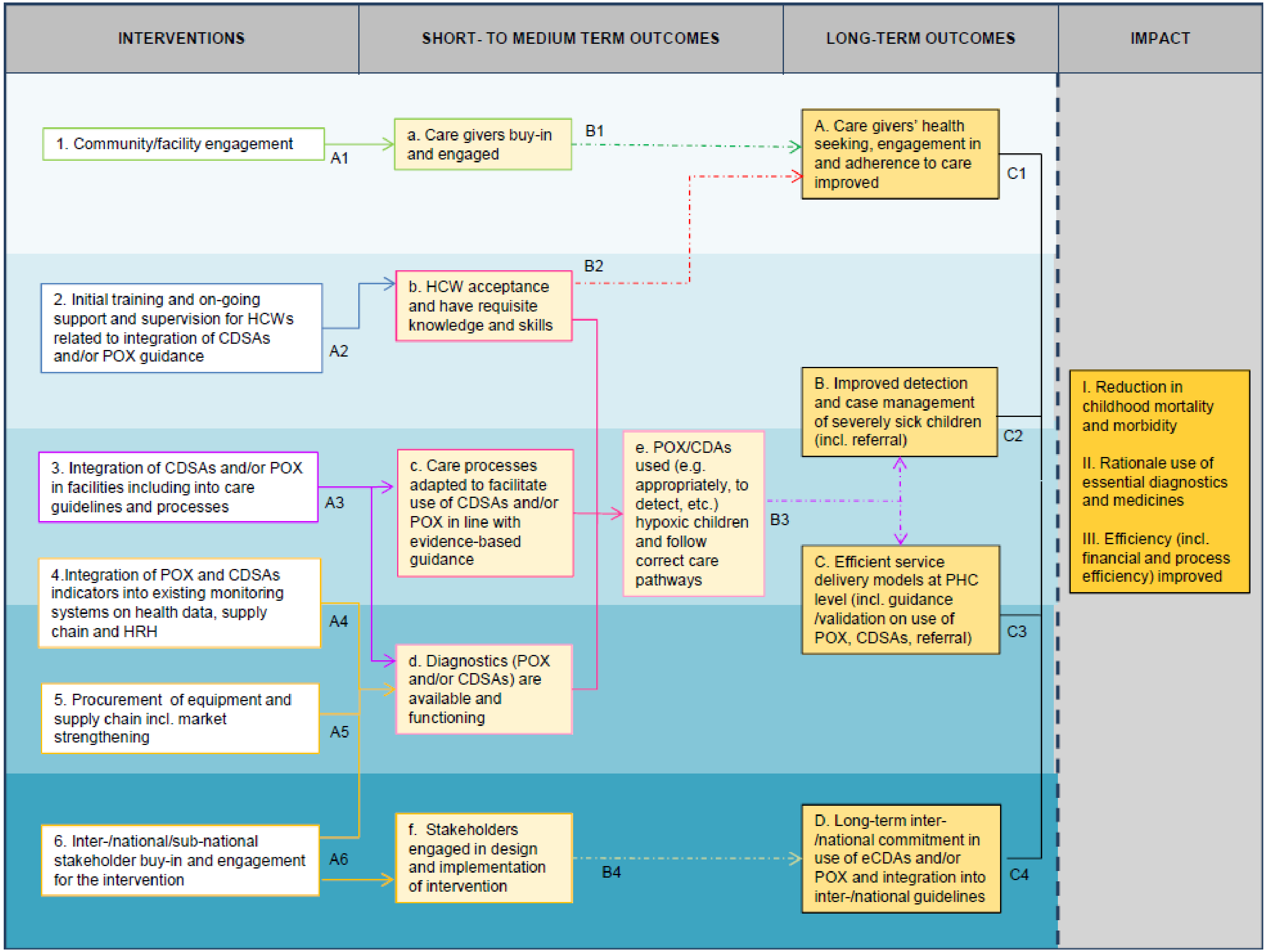

Supplement: TIMCI_WHOERC.0003406_v2.3 [file mmc11.pdf]
